# Differences between predicted outer membrane proteins of genotype 1 and 2 *Mannheimia haemolytica*

**DOI:** 10.1186/s12866-020-01932-2

**Published:** 2020-08-12

**Authors:** Michael L. Clawson, Gennie Schuller, Aaron M. Dickey, James L. Bono, Robert W. Murray, Michael T. Sweeney, Michael D. Apley, Keith D. DeDonder, Sarah F. Capik, Robert L. Larson, Brian V. Lubbers, Brad J. White, Jochen Blom, Carol G. Chitko-McKown, Dayna M. Brichta-Harhay, Timothy P. L. Smith

**Affiliations:** 1 United States Department of Agriculture, Genetics, Breeding, and Animal Health Research Unit, Agricultural Research Service, U.S. Meat Animal Research Center, Clay Center, NE USA; 2 Zoetis, Kalamazoo, MI USA; 3 Kansas State University, Manhattan, KS USA; 4 Veterinary and Biomedical Research Center, Inc, Manhattan, KS USA; 5 Texas A&M AgriLife Research, Texas A&M University System, Amarillo, TX USA; 6 Department of Veterinary Pathobiology, Texas A&M College of Veterinary Medicine and Biomedical Sciences, Texas A&M University, College Station, TX USA; 7 Justus-Liebig-University Giessen, Giessen, Hesse Germany

**Keywords:** *Mannheimia haemolytica*, Bovine respiratory disease, Genomics, Genotypes, Adhesins, Peptidase S6, Porin, Ligand-Gated Channel

## Abstract

**Background:**

*Mannheimia haemolytica* strains isolated from North American cattle have been classified into two genotypes (1 and 2). Although members of both genotypes have been isolated from the upper and lower respiratory tracts of cattle with or without bovine respiratory disease (BRD), genotype 2 strains are much more frequently isolated from diseased lungs than genotype 1 strains. The mechanisms behind the increased association of genotype 2 *M. haemolytica* with BRD are not fully understood. To address that, and to search for interventions against genotype 2 *M. haemolytica*, complete, closed chromosome assemblies for 35 genotype 1 and 34 genotype 2 strains were generated and compared. Searches were conducted for the pan genome, core genes shared between the genotypes, and for genes specific to either genotype. Additionally, genes encoding outer membrane proteins (OMPs) specific to genotype 2 *M. haemolytica* were identified, and the diversity of their protein isoforms was characterized with predominantly unassembled, short-read genomic sequences for up to 1075 additional strains.

**Results:**

The pan genome of the 69 sequenced *M. haemolytica* strains consisted of 3111 genes, of which 1880 comprised a shared core between the genotypes. A core of 112 and 179 genes or gene variants were specific to genotype 1 and 2, respectively. Seven genes encoding predicted OMPs; a peptidase S6, a ligand-gated channel, an autotransporter outer membrane beta-barrel domain-containing protein (AOMB-BD-CP), a porin, and three different trimeric autotransporter adhesins were specific to genotype 2 as their genotype 1 homologs were either pseudogenes, or not detected. The AOMB-BD-CP gene, however, appeared to be truncated across all examined genotype 2 strains and to likely encode dysfunctional protein. Homologous gene sequences from additional *M. haemolytica* strains confirmed the specificity of the remaining six genotype 2 OMP genes and revealed they encoded low isoform diversity at the population level.

**Conclusion:**

Genotype 2 *M. haemolytica* possess genes encoding conserved OMPs not found intact in more commensally prone genotype 1 strains. Some of the genotype 2 specific genes identified in this study are likely to have important biological roles in the pathogenicity of genotype 2 *M. haemolytica*, which is the primary bacterial cause of BRD.

## Background

Bovine respiratory disease (BRD) affects cattle worldwide and is a serious animal health and well-being concern [1–4]. Additionally, global monetary losses to cattle agriculture from BRD are estimated to exceed three billion dollars annually, with close to a billion lost in the United States alone [5, 6]. Multiple environmental and host factors cause or contribute to BRD, as well as viral, bacterial, and eukaryotic pathogens [7–9].

*Mannheimia haemolytica* causes a necrotizing, fibrinous, pneumonia or pleuropneumonia in cattle, and is the predominant bacterial pathogen isolated from BRD cases [3, 10]. An opportunistic pathogen, *M. haemolytica* is often found in the upper respiratory tract of cattle without signs of BRD, and can invade the lower respiratory tract and cause disease when animals are stressed and/or immunocompromised [8, 11]. *M. haemolytica* is a member of the *Pasteurellaceae,* and consists of Gram-negative, non-motile, non-spore-forming, facultative anaerobic rod or coccobacilli bacteria [3, 8, 12].

The propensity for *M. haemolytica* to cause pneumonia varies at the subspecies level by both capsular serotypes and whole genome-based genotypes. There are 12 capsular serotypes of *M. haemolytica*, of which serotypes 1, 2, and 6 are most commonly found in cattle [11–14]. Strains of all three serotypes can be isolated from the upper or lower respiratory tract of cattle with or without BRD, however, serotype 2 is more commonly isolated from the upper respiratory tract of cattle without signs of BRD, and serotypes 1 and 6 are more commonly isolated from the lower respiratory tract of cattle with BRD [11, 13, 15]. There are also two major genotypes of *M. haemolytica* frequently found in North American cattle [16]. Of them, genotype 1 is more commonly isolated from the upper respiratory tract of cattle without signs of BRD, and genotype 2 is more commonly isolated from the lungs of cattle with BRD [16]. Relationships between serotypes and genotypes have yet to be established.

Disproportionate representation of *M. haemolytica* substrains in the diseased lungs of cattle is connected to disproportionate proliferation in the nasopharynx during times of stress [11, 13, 17, 18]. Serotype 1 *M. haemolytica*, for example, can undergo expansive growth when cattle are stressed, thereby overwhelming serotype 2 *M. haemolytica* within the nasopharynx, and establishing an infection of the lungs through the inhalation of infected droplets [11, 17, 18]. The extent of proteins and/or their isoforms that are specific to *M. haemolytica* substrains that preferentially proliferate in the nasopharynx and infect the lungs of cattle is not fully understood. Identification of these proteins, especially outer membrane proteins (OMPs), may indicate why some substrains of *M. haemolytica* associate with BRD more strongly than others.

The primary aim of this study was to bioinformatically identify OMPs that are specific to genotype 2 *M. haemolytica* and characterize the extent of their diversity. In support of that we had four goals. The first was to sequence, assemble, and close the chromosomes of 69 strains that collectively represent a broad spectrum of genotype 1 and 2 genetic diversity. The second was to search the chromosomes for genes with specificity to either of the genotypes. The third was to analyze proteins with genotype 2 specificity for beta-barrel topology which is indicative of outer membrane localization in Gram negative bacteria [19]. The fourth was to characterize the isoform diversity of predicted outer membrane proteins with genotype 2 specificity within the 69 closed chromosomes sequenced in this study, and up to 1075 additional *M. haemolytica* strains, of which 1062 had been previously sequenced with short read technology and incompletely assembled. Additionally, we sought to compare strain genotypes with molecular serotypes to address long standing questions regarding how they may be related.

## Results

### Strain sequencing and whole genome phylogenetics

The genomes of 69 *M. haemolytica* strains representing genotypes 1 (*n* = 35) and 2 (*n* = 34) were sequenced to closed, circularized, error-corrected chromosomes with an average coverage of 27-fold (range = 7.9–48). These strains had previously been sequenced with short-read technology, which facilitated their characterization by concatenated nucleotide polymorphism alleles into genotypes and subtypes, but precluded de novo assembly of their chromosome [16]. The strains collectively represented all genotypes and subtypes characterized to date (1b = 6, 1c = 5, 1e = 5, 1f = 10, 1i = 5, 2b = 16, 2c = 3, 2d = 8, 2e = 5). Additionally, four genotype 1 and two genotype 2 strains with ambiguous subtype assignments were also sequenced (Additional file 1: Table S1).

**Fig. 1:**
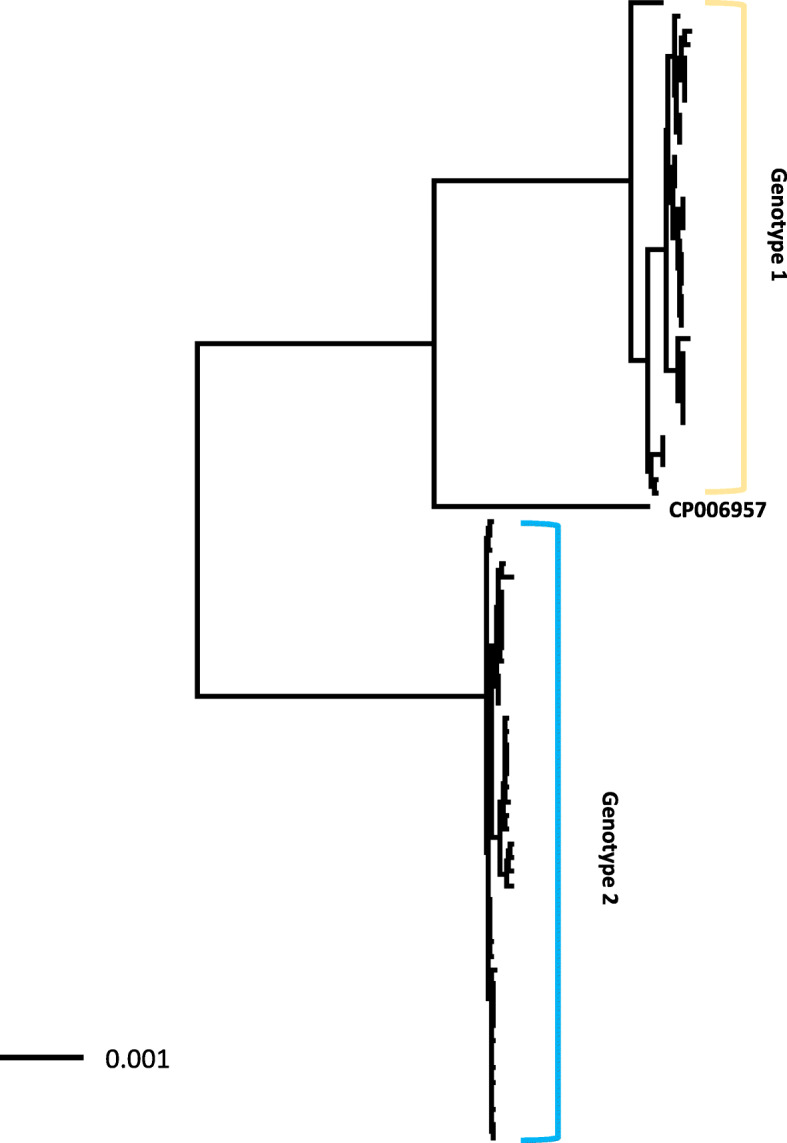
EDGAR generated phylogenetic tree of closed chromosomes from 82 *M. haemolytica* strains. The tree was constructed with DNA shared between the 69 strains sequenced in this study and 13 additional strains with closed genomes available in GenBank. The one strain available in GenBank (CP006957) that placed on a long branch between genotype 1 and 2 clusters is denoted. The scale bar represents substitutions per site

A phylogenetic tree constructed in EDGAR from conserved core sequence shared between the 69 genomes sequenced in this study, and 13 additional closed, circularized *M. haemolytica* genomes available in GenBank, contained two clades that corresponded with genotypes 1 and 2 (Fig. 1). The tree additionally contained a single outer taxonomic unit that placed between the two genotypes that was represented by a previously sequenced *M. haemolytica* strain (USDA-ARS-USMARC-184, GenBank #CP006957). This strain may represent a third *M. haemolytica* genotype, or a very deep branch within genotype 1.

**Fig. 2:**
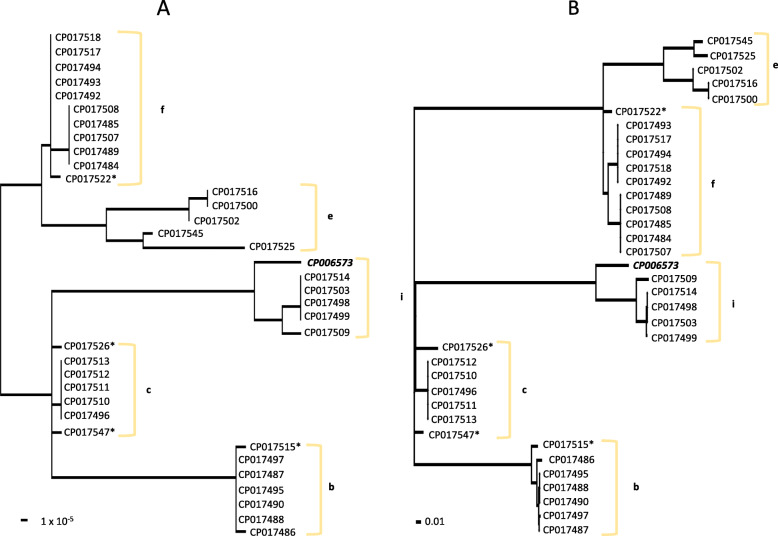
EDGAR (**a**) and Parsnp (**b**) generated phylogenetic trees of genotype 1 *M. haemolytica* strains. Both trees were constructed with DNA shared between 35 genotype 1 *M. haemolytica* strains sequenced in this study and one genotype 1 strain with a closed genome available in GenBank. Lower case letters represent subtypes. The label for the single genotype 1 strain from GenBank is bold and italicized. Asterisks depict strains that were not subtyped with statistical confidence in a previous study [16]. The scale bar represents substitutions per site. Both trees are midpoint rooted. Sequence between and including direct repeats flanking the insertion of ICE*Mh1* were masked from the core genome sequences used to generate the Parsnp tree, which equaled 81% of a reference genome (GenBank# CP017502)

**Fig. 3:**
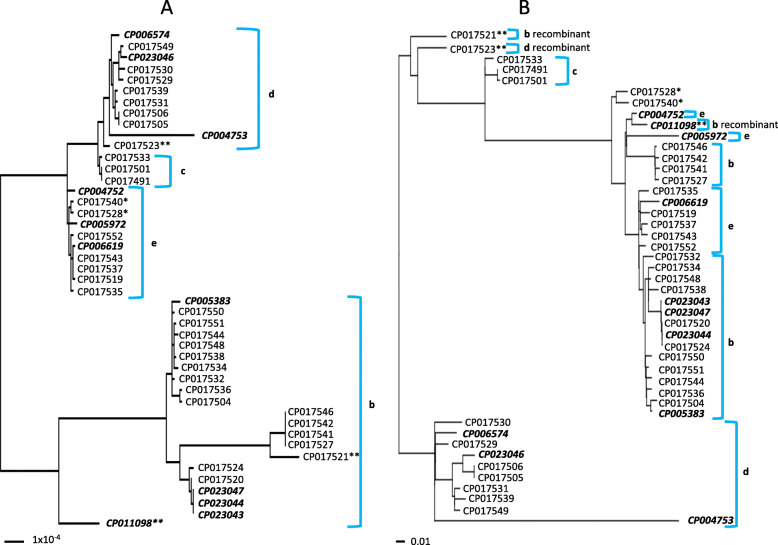
EDGAR (**a**) and Parsnp (**b**) generated phylogenetic trees of genotype 2 *M. haemolytica* strains. Both trees were constructed with DNA shared between 34 genotype 2 *M. haemolytica* strains sequenced in this study and 11 genotype 2 strains (bold and italicized) with a closed genome available in GenBank. Lower case letters represent subtypes. Single asterisks depict strains that were not subtyped with statistical confidence in a previous study and double asterisks depict those previously identified as recombinant [16]. Sequence between and including direct repeats flanking the insertion of ICE*Mh1* was masked from the core genome sequences used to generate the Parsnp tree, which equaled 76% of a reference genome (GenBank# CP004752)

The five subtypes of genotype 1 clustered separately from each other in trees generated from both EDGAR and Parsnp (Fig. 2). However, the EDGAR and Parsnp trees did not agree in their placement of genotype 2 subtypes. All four genotype 2 subtypes clustered separately from each other in the tree generated by EDGAR, with considerable diversity within the 2b cluster (Fig. 3). However, subtypes b and e placed together in the Parsnp tree (Fig. 3). A major difference between the EDGAR and Parsnp generated trees was that sequence corresponding to an integrative and conjugative element (ICE) that is known to reside in genotype 2 *M. haemolytica* (ICE*Mh1*) was excluded from the Parsnp tree build. This suggests that the classification system of genotype 2 subtypes is impacted by the inclusion or exclusion of ICE variation.

### Comparison of molecular serotypes with whole genome phylogenetics

All genotype 1 strains with closed genomes (1b = 6, 1c = 5, 1e = 5, 1f = 10, 1i = 6, and the four with ambiguous subtypes prior to this study) molecularly serotyped as serotype 2, as did the one strain that placed as an intermediate between genotypes 1 and 2 (Additional file 1: Table S1). Ninety five percent of the 2b strains either sequenced in this study or that were available in GenBank (*n* = 20/21) molecularly serotyped as serotype 1, as did 100% of the 2e strains (*n* = 10), (Additional file 1: Table S1). One 2b strain (GenBank# CP017521) molecularly serotyped as serotype 6 (Additional file 1: Table S1). This strain was previously flagged as having recombinant sequence from genotype 2 other than subtype 2b [16]. All 2c strains (*n* = 3) and the 2d strains (*n* = 11) with closed genomes serotyped as serotype 6 (Additional file 1: Table S1). Thus, there appears to be a strong correlation between genotypes and subtypes with serotypes, although a larger collection of strains would be needed for that determination.

### Identification of pan and core genomes, and proteins with specificity to genotype 2 *M. haemolytica* containing amphipathic beta-strands typical of outer membrane proteins

**Fig. 4:**
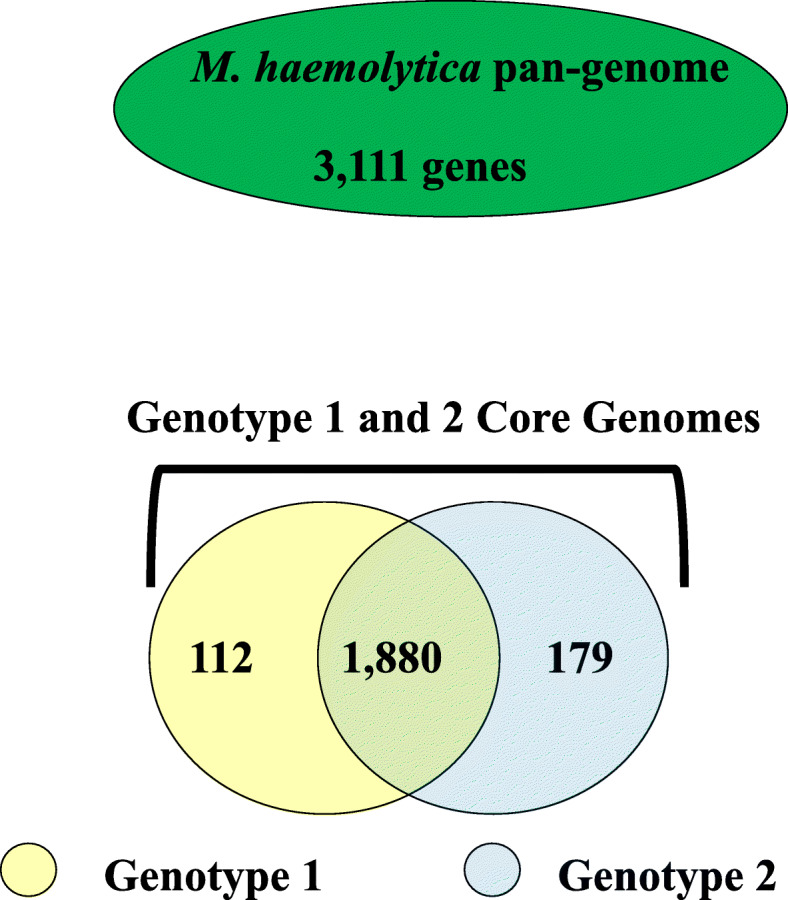
*M. haemolytica* pan and core genomes. These were determined from the 69 genomes sequenced and assembled in this study. The pan genome is the total number of different genes observed across the strains. Genotype 1 core genes are those observed in all of the genotype 1 strains and none of the genotype 2 strains. Genotype 2 core genes are those observed in all of the genotype 2 strains and none of the genotype 1 strains. Core genes shared between genotype 1 and 2 were observed in all 69 strains

The pan-genome of the 69 *M. haemolytica* sequenced chromosomes consisted of 3111 genes (Fig. 4, Additional file 2: Table S2), of which 1880 comprised a shared common core between genotypes 1 and 2 (Additional file 3: Table S3). Among the remaining 1231 pan-genome genes, there were 112 and 179 with specificity to genotype 1 and 2, respectively, as defined by presence in all members of one genotype and absence in all members of the other (Additional file 4: Table S4 and Additional file 5: Table S5). Of the 179 genes with specificity to genotype 2, four were predicted to encode proteins containing amphipathic beta-strands by all four OMP prediction software programs. The NCBI annotations for those proteins were; peptidase S6, an autotransporter outer membrane beta-barrel domain-containing protein (AOMB-BD-CP), a ligand-gated channel, and a porin (Additional file 5: Table S5). These four proteins were investigated further for the extent of their specificity to genotype 2 *M. haemolytica* and overall diversity*,* as was a class of trimeric autotransporter adhesins with members also predicted to locate to the outer membrane. Although the adhesins were not flagged by EDGAR as specific to genotype 2 *M. haemolytica*, significant differences were observed between them in genotype 1 and 2 *M. haemolytica* strains based on annotation searches and initial sequence alignments.

### Peptidase S6

Differing numbers of peptidase S6 genes were annotated in five strains that each represented a different subtype of genotype 1 (genotype 1 subtype representative strains), and four strains that each represented a different subtype of genotype 2 (genotype 2 subtype representative strains). The five genotype 1 subtype representative strains all had three annotated peptidase S6 genes and one peptidase S6 pseudogene. Conversely, the four genotype 2 subtype representative strains had two annotated peptidase S6 genes, and two annotated S6 family IgA-specific metalloendopeptidase genes, which encoded peptides with regions of distinct homology with genotype 1 and/or 2 peptidase S6 isoforms. The genotype 1 peptidase S6 pseudogene was the homolog of the genotype 2 specific peptidase S6 as predicted by EDGAR. To confirm that, and to identify the genetic determinants conferring that specificity, protein sequences of the two S6 family IgA-specific metalloendopeptidase genes and the two peptidase S6 genes of the four genotype 2 subtype representative strains were aligned together with those of the three peptidase S6 genes and one peptidase S6 pseudogene of the five genotype 1 representative strains (Additional file 6: Figure S1).

The isoforms of the genotype 1 peptidase S6 pseudogene were closest in homology to those encoded by the peptidase S6 gene identified as specific for genotype 2. The pseudogene encoding the isoforms did not contain a stop codon and appeared to have been prematurely truncated by the NCBI annotation pipeline (Additional file 6: Figure S1).However, manual translation of immediate flanking three prime sequence of the pseudogene isoforms identified a stop codon after an additional 19 amino acids, with virtually no homology to the genotype 2 specific peptidase S6 gene (Additional file 6: Figure S1). Thus, although both genotype 1 and 2 *M. haemolytica* encode peptidase S6 proteins, one is specific to genotype 2 *M. haemolytica* as it has been knocked out in genotype 1 *M. haemolytica*.

The diversity of protein isoforms encoded by the genotype 2 specific peptidase S6 gene and its genotype 1 pseudogene homolog is low. Between the genomes of 1144 *M. haemolytica* strains (69 sequenced in this study, 13 sequenced and assembled previously, and 1062 sequenced previously with short read technology and incompletely assembled), four isoforms were observed in genotype 1, five were observed in genotype 2, and an additional isoform was observed in USDA-ARS-USMARC-184 (Additional file 1: Table S1 and Additional file 7: Figure S2). All four isoforms observed between the genotype 1 strains were knockouts with the same premature stop codon. None of the five isoforms observed in the genotype 2 strains, or the isoform of USDA-ARS-USMARC-184 contained a premature stop codon homologous to the genotype 1 strains. Just one genotype 2 strain contained a premature stop codon, but this was located closer to the carboxyl end of the predicted protein than the conserved stop codon observed in of all genotype 1 strains (Additional file 1: Table S1 and Additional file 7: Figure S2).

### AOMB-BD-CP

Two AOMB-BD-CP genes were annotated in the five genotype 1 subtype representative strains, versus three in the four genotype 2 representative strains. Two AOMB-BD-CP genes, while distinct from each other, encoded isoforms with greater than 99% identity across the genotypes (Additional file 8: Figure S3). The third AOMB-BD-CP gene was tagged by EDGAR as specific to genotype 2 *M. haemolytica* and was not observed in the five genotype 1 subtype representative strains. Identical isoforms were encoded by the genotype 2 specific AOMB-BD-CP gene in all four genotype 2 subtype representative strains (Additional file 8: Figure S3).

Although genotype 2 strains have an unique AOMB-BD-CP gene, corresponding isoform sequence from the four genotype 2 subtype representative strains was found to have 54% identity with the carboxyl side of a S6 family IgA-specific metalloendopeptidase isoform of the same four strains, as well as a peptidase S6 isoform of the five genotype 1 subtype representative strains (Additional file 9: Figure S4). Notably, the S6 family IgA-specific metalloendopeptidase and peptidase S6 isoforms contain 766 and 1270 amino acids preceding the start amino acid of the genotype 2 specific AOMB-BD-CP gene isoform, respectively (Additional file 9: Figure S4). This indicates that the genotype 2 specific AOMB-BD-CP gene could be a truncated peptidase S6 or S6 family IgA-specific metalloendopeptidase gene, or incorrectly annotated.

The genotype 2 specific AOMB-BD-CP gene is preceded by 105 bp of intergenic sequence and a serine O-acetyltransferase gene in the four genotype 2 subtype representatives. Thus, incorrect annotation of the genotype 2 specific AOMB-BD-CP gene, where a large section of the actual start of the gene may have been deleted is not apparent. Additionally, translated sequence of the four genotype 2 subtype representatives immediately upstream of the annotated start codon of the genotype 2 specific AOMB-BD-CP gene introduced a stop codon 36 theoretical amino acid positions from the encoded start site (Additional file 9: Figure S4). This also does not support the notion that a large portion of the gene has been incorrectly excluded from the 5′ end. No leader peptide sequence was detected in the genotype 2 specific AOMB-BD-CP isoform of the four genotype 2 subtype representatives. These results indicate that the AOMB-BD-CP gene specific to genotype 2 *M. haemolytica* is likely a severely truncated version of a S6 family IgA-specific metalloendopeptidase gene, peptidase S6 gene, or other related gene. Consequently, this gene was not investigated further in this study.

### Porin

Three genes encoding an aquaporin, an aquaporin Z, and a maltoporin, plus one porin pseudogene were annotated in the genotype 1 subtype representative strain genomes. Conversely, four genes encoding an aquaporin, an aquaporin Z, a maltoporin, and a porin were all annotated in the genotype 2 subtype representative genomes. The gene annotated simply as a porin was flagged by EDGAR as being specific for genotype 2 *M. haemolytica* with the porin pseudogene as its homolog. Annotation within the genotype 1 porin pseudogene indicated it contained an internal stop codon. To examine the location of the stop codon, and the extent of isoform sequence similarity encoded by all of the porin genes and pseudogene, the isoforms of all four types of porin encoding genes and the pseudogene were aligned for the genotype 1 and 2 subtype representative strains (Additional file 10: Figure S5). The peptides of the genotype 1 porin pseudogene and the genotype 2 porin gene shared 98% identity (Additional file 10: Figure S5). However, an identical stop codon within the porin pseudogene of all five of the genotype 1 strains resulted in a truncated isoform missing more than 50% of the amino acids found in the genotype 2 homolog (Additional file 10: Figure S5).

**Fig. 5:**
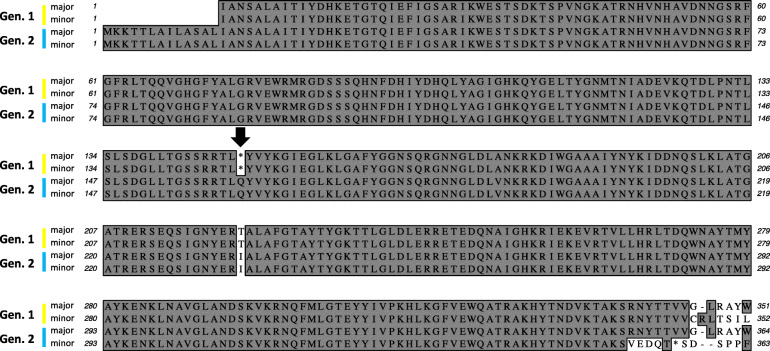
*M. haemolytica* porin gene and pseudogene isoform diversity. The porin gene is specific to genotype 2 *M. haemolytica* as all observed genotype 1 strains have the pseudogene. Only two isoforms, designated major and minor in the figure, were observed for each genotype. The major isoform of each genotype was observed in over 99% of genotype members. The black arrow highlights the premature stop codon within the major and minor isoforms encoded by the genotype 1 pseudogene

Overall diversity of the porin pseudogene and gene isoforms was low in both genotype 1 and 2 strains, respectively. Within 1144 *M. haemolytica* strains of known genotypes, only two isoforms of the porin pseudogene were observed in genotype 1 strains and shared over 98% identity. Over 99% of genotype 1 strains had the same, “major” isoform of the truncated protein (Fig. 5 and Additional file 1: Table S1) which was also observed in strain USDA-ARS-USMARC-184. Similarly, only two isoforms of the porin gene were observed in genotype 2 strains that shared over 96% identity (Fig. 5). Over 99% of genotype 2 strains had the same, “major” isoform of the protein. However, two genotype 2 *M. haemolytica* strain genomes available in GenBank prior to this study (CP005972, CP006574) were negative for the genotype 2 specific porin gene by annotation and blast searches. Thus, although a full length porin gene was found to be specific for genotype *2 M. haemolytica,* with a single isoform predominating at a population level, a low frequency of genotype *2 M. haemolytica* strains do not appear to harbor the gene.

### Ligand-Gated Channel

A ligand-gated channel gene and pseudogene were each annotated in the genotype 1 subtype representative strain genomes versus two ligand-gated channel genes in the genotype 2 subtype strain representative genomes. One of the two ligand-gated channel genes was flagged by EDGAR as being specific for genotype 2 *M. haemolytica,* with the genotype 1 pseudogene as its homolog. Alignments of predicted proteins encoded by the genes and pseudogene of the representative strains revealed two distinct ligand-gated channel homologs, with greater than 78% similarity between all of the isoforms encoded by the same homolog, and less then 30% similarity of isoforms encoded by different homologs (Additional file 11: Figure S6). The homolog flagged as specific to genotype 2 *M. haemolytica* encoded identical predicted protein isoforms across the genotype 2 subtype strain representative genomes. The counterpart genotype 1 pseudogene encoded a single protein isoform with a premature stop codon across the genotype 1 subtype strain representative genomes. The stop codon occurred at amino acid position 82 of the protein which was predicted to exceed 700 amino acids. The genotype 2 homolog isoform was found to contain motifs for the TonB-hemin, CirA PRK13483 superfamily, and OM_channel superfamilies through Blast searches, indicating it has a role in iron, or iron containing compound transport across the outer membrane.

Overall diversity of the genotype 2 specific ligand-gated channel gene was low. Within 1142 *M. haemolytica* strains, all but one genotype 2 genome had the same isoform sequence, with the minor isoform differing by one amino acid (Additional file 12: Figure S7). Similarly, all but one genotype 1 strain also had the same exact isoform sequence with the premature stop codon at position 82. The isoform of the remaining genotype 1 strain contained a different stop codon observed at amino acid position 446 of the protein, with exceedingly low downstream homology to any of the ligand gated channel isoforms observed in either genotype 1 or 2 *M. haemolytica* (Additional file 1: Table S1 and Additional file 12: Figure S7). Consequently, not a single genotype 1 strain was identified in this study with an intact version of the ligand-gated channel gene flagged as specific to genotype 2 *M. haemolytica*. Strain USDA-ARS-USMARC-183 had a unique sequence that was identical to the predominant genotype observed in genotype 1 except at the stop codon site, where it had the same glutamine residue observed in all genotype 2 strains.

### Adhesins

**Fig. 6:**
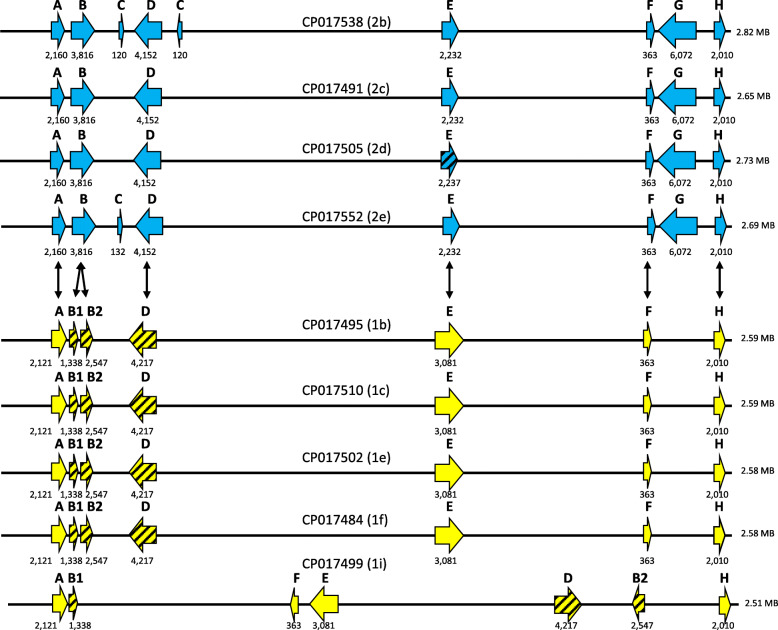
Map of adhesin genes and pseudogenes annotated in genotypes 1 and 2 *M. haemolytica*. The map is approximate and not to scale. Adhesin genes are represented with horizontal arrows that are solid yellow for genotype 1 strains and solid blue for genotype 2 strains. Pseudogenes are represented with striped horizontal arrows that are similarly color coded. Numbers below the genes and pseudogenes represent their nucleotide size. Black vertical arrows indicate homology of adhesin genes and/or pseudogenes between genotype 1 and 2 strains. The adhesin B gene of genotype 2 strains is split into two pseudogenes in genotype 1 strains. The adhesin B1 and B2 pseudogenes of the 1i strain (GenBank# CP017499) have greater genome separation from each other versus the other four genotype 1 strains due to an apparent genome inversion. The adhesin D gene was annotated to a similar size in genotype 1 and 2 strains by the NCBI prokaryotic genome annotation pipeline, however the D pseudogene in genotype 1 strains encodes multiple stop codons. The adhesin G gene of genotype 2 strains was not annotated or observed in genotype 1 strains. The adhesin E gene of all four genotype 2 strains is truncated in comparison to genotype 1 strains

Four adhesin genes and three adhesin pseudogenes were annotated in all five of the genotype 1 subtype representative genomes. Seven to nine adhesin genes were annotated in three of the four genotype 2 subtype representative genomes and six adhesin genes plus one pseudogene were annotated in remaining representative genome (Fig. 6). Three adhesin genes showed specificity to genotype 2 *M. haemolytica* (B*,* D*,* and G, Fig. 6). All three of the genes encode YadA domains, indicating that they are all trimeric autotransporter adhesins [20, 21].

The adhesin B gene was comprised of 3816 nucleotides in all four of the genotype 2 subtype representative genomes, but was split into two pseudogenes (B1: 1338 nucleotides and B2: 2547 nucleotides) in all five of the genotype 1 subtype representative genomes (Fig. 6, also see Additional file 13: Figure S8 for an alignment of genotype 2 adhesin B isoforms with genotype 1 adhesin B1 isoforms, and Additional file 14: Figure S9 for an alignment of genotype 2 adhesin B isoforms with genotype 1 adhesin B2 isoforms). This same arrangement of an intact genotype 2 adhesin B gene versus incomplete genotype 1 B1 and B2 pseudogenes was observed in the additional 30 genotype 1 and 29 of the additional 30 genotype 2 strains sequenced in this study (Additional file 1: Table S1). One genotype 2 strain, (GenBank# CP017521), had an adhesin B gene split into two pseudogenes similar to genotype 1 strains (Additional file 1: Table S1).

An adhesin D gene of 4152 nucleotides was annotated in the genomes of all four genotype 2 subtype strain representatives. A similar sized adhesin D pseudogene of 4217 nucleotides was annotated in the genomes of all five genotype 1 subtype strain representatives (Fig. 6). All five genotype 1 adhesin D pseudogenes contained a 65 bp deletion compared to their genotype 2 homologs, which resulted in a disruption of amino acid homology at amino acid position 199, along with multiple stop codons starting at amino acid position 208 of a 1405 amino acid protein (Additional file 15: Figure S10). The additional 30 genotype 2 strains sequenced in this study also had an adhesin D gene, whereas 29 of 30 sequenced genotype 1 strains had the pseudogene (Additional file 1: Table S1). The one genotype 1 exception was a strain (GenBank# CP017526) that has not phylogenetically placed within subtype c with strong support (Fig. 2, Additional file 1: Table S1, see also Additional file 16: Figure S11 for the adhesin D isoform sequences).

An adhesin G gene of 6072 nucleotides was annotated in all four genotype 2 subtype representative strains. In contrast, this gene was neither annotated nor detected in the genomes of the five genotype 1 subtype representative strains. An adhesin G gene was also not observed in the genomes of the 30 additional genotype 1 strains sequenced in this study, whereas all of the additional 30 genotype 2 strains sequenced in this study did have the adhesin G gene (Additional file 1: Table S1).

Isoform diversity encoded by adhesin genes B, D, and G was low within a particularly diverse collection of genotype 2 strains (Zoetis collection, described in Methods). Of 290 strains 98.6% (*n* = 286) had an identical adhesin B isoform (Additional file 1: Table S1 and Additional file 17: Figure S12). A total of 5 full length genotype 2 adhesin B isoforms were observed in this study with 5 variable sites identified throughout the protein. Of 284 strains scored for the adhesin D gene, 98.2% (*n* = 279) had one of three isoform sequences (Additional file 1: Table S1 and Additional file 16: Figure S11) that shared 99.85% identity. Notably, one genotype 2 strain did have an adhesin D isoform that contained multiple stop codons, starting at amino acid position 70 of a 1371 amino acid protein. Lastly, of 290 strains scored for the adhesin G gene, 83.8% (*n* = 243) had the exact same isoform sequence (Additional file 1: Table S1, Additional file 18: Figure S13). The second most frequent adhesin G isoform (4.48%) had a premature stop codon at amino acid position 1446 of a 1993 protein (Additional file 18: Figure S13). Many of the rarer adhesin G isoforms were defined by large blocks of amino acid deletions (Additional file 18: Figure S13).

**Fig. 7:**
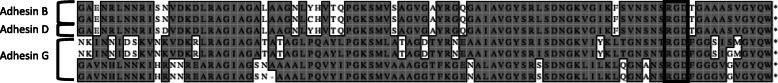
Conserved RGD motif in C-terminus of adhesin isoforms. Alignment of all genotype 2 adhesin B, D, and G unique isoforms at the last 91 or 92 amino acids of the isoforms. A genotype 1 strain (GenBank# CP0175216) is also represented in this alignment. The conserved RGD motif is highlighted within a black rectangle

Typical of trimeric autotransporter adhesins, isoforms encoded by the adhesin B, D, and G genes showed greater conservation near the C-terminal domain of the protein, which is involved in translocation and serves as the membrane anchor (Additional file 19: Figure S14), [22]. Notably, all of the adhesin B, D, and G isoforms that did not contain premature stop codons were found to have an Arginine, Glycine, and Aspartate (RGD) motif near the C-terminus (Fig. 7 and Additional file 19: Figure S14). This motif has a central role in cell attachment, although it must be surface exposed and unencumbered by flanking amino acids [23]. While the three adhesins specific to genotype 2 *M. haemolytica* all had this motif, RGD was not observed in adhesin A, C, E, F or H isoforms in any of the genotype 1 or 2 *M. haemolytica* strains sequenced in this study. It additionally was not encoded by genotype 1 B1 pseudogene adhesins. The motif was, however, encoded by the B2 pseudogene adhesin isoforms of all 35 genotype 1 strains sequenced in this study. Given that the B2 pseudogene adhesin is predicted as being severely truncated and non-functional, the RGD motif was not found in any full length adhesin isoforms other than those of B, D, and G.

## Discussion

The two major genotypes of *M. haemolytica* and/or their subtypes had not been connected to serotypes prior to this study. A comparison of genotypes and serotypes in the isolates sequenced for the present study revealed a perfect correlation of serotype 2 with genotype 1. The relationship of genotype 2 with serotypes was more complex, with genotype 2 subtypes c and d completely correlated with serotype 6, subtype e completely correlated with serotype 1, and subtype b imperfectly (one exception) correlated with serotype 1.

Although the overall correspondence of genotypes and serotypes was strong, the genotyping/subtyping classification system could be improved upon in the future. Genotype 2 subtypes b and e strains placed on separate branches of a phylogenetic tree when core genomes were used for its construction (Fig. 3). However, these two subtypes collapsed together in a tree where ICE*Mh1*, an ICE known for containing antimicrobial resistance genes [24], was removed from the core genome sequences used for the build (Fig. 3). This is because the principal variation that separates the two subtypes in Fig. 3 is within core genes of the ICE itself, indicating that different backbones, or phylotypes of the ICE are circulating within *M. haemolytica*. Although virtually all genotype 2 *M. haemolytica* examined to date have a version of this ICE (with or without antimicrobial resistance genes), it would be preferable to treat it as a separate character in a classification scheme that is independent from core genome variation, to account for the fact that it is a mobile element. To that end, the one 2b strain sequenced in this study that molecularly serotyped as an A6 (GenBank# CP017521), clustered between subtypes c and d in the phylogenetic tree build without ICE sequence (Fig. 3).

The rationale behind our focus on putative OMPs with specificity to genotype 2 *M. haemolytica* versus 1 was first, the majority of respiratory disease caused by *M. haemolytica* in cattle has thus far been genotype 2, and second, the overall diversity of genotype 1 strains in cattle has not been characterized as it has for genotype 2 strains [16]. Given that the genome of one serotype 2 strain isolated in 2008 from a *Bos taurus* in Kansas, U.S., (GenBank# CP006957) formed a very deep branch between genotype 1 and 2 clades (Fig. 1), we potentially have only scratched the surface of genotype 1 and/or serotype 2 genetic diversity [25]. Strains of *M. haemolytica* characterized as genotype 1, serotype 2, or both have been found in the diseased lungs of cattle [15, 16]. Additionally, serotype 2 *M. haemolytica* causes pneumonic pasteurellosis in sheep [3], however, the extent of pathogenicity within the genotype 1 clade of *M. haemolytica* has not been determined. If all or some subtypes of genotype 1 are shown to be pathogenic to cattle, the results of this study could be expanded by examining OMPs shared by pathogenic genotype 1 and 2 subtypes.

Peptidase S6 is an enzyme that cleaves IgA [26]. Given that IgA is the predominant antibody found on mucosal surfaces of most mammals [27], pathogens with an ability to neutralize IgA have a distinct advantage in invading mucosal surfaces. While a peptidase S6 annotated gene was found to be specific to genotype 2 *M. haemolytica*, the exact function of this gene is unknown. The protein sequence of this gene has a domain placing it within the peptidase S6 superfamily, which consists in part of immunoglobulin A1 proteins. Humans and other primates have two IgA heavy constant region (Cα) genes and thus encode IgA subclasses IgA1 and IgA2, whereas cattle have just one (Cα) gene and do not encode IgA1 [27]. Thus, the true target of this enzyme could be bovine IgA2 or a non-IgA molecule. In addition to the peptidase S6 superfamily domain, this protein also has a pertactin-like passenger domain, meaning the passenger portion of the protein may be cleaved from the C-terminal portion embedded in the outer membrane, and either remain in contact with the C-terminal portion covalently, or be released as a free extracellular protein. While the function remains to be proven, this protein is highly conserved and specific to the genotype 2 *M. haemolytica* in this study.

Porins are the most abundant proteins found on the outer membrane of gram negative bacteria [28]. They facilitate the exchange of nutrients over the outer membrane and can have important roles in pathogenesis [28, 29]. *M. haemolytcia ompA* encodes a porin protein that has been well researched for both its role in *M. haemolytica* pathogenicity, and for its potential as a vaccine candidate (see [30] for review of *M haemolytica* OmpA). This protein has adhesive properties, as it binds to cell-surface fibronectin [31] and lactoferrin [32]. Vaccination of cattle with either live *M. haemolytica* or recombinant *M. haemolytica* OmpA has generated a high OmpA antibody response [30]. More recently, a different porin was identified that reacted strongly with anti-serotype 1, 2, and 6 bovine sera [33]. The porin gene identified in this study as specific to genotype 2 *M. haemolytica* is neither *ompA,* nor the recently identified porin described above. The gene is highly conserved in genotype 2 *M. haemolytica* given that one porin isoform sequence predominated across almost all of the genotype 2 strains examined in this study.

Iron acquisition of *M. haemolytica* is an active area of research and does not occur through siderophores (see Confer and Ayalew 2018 for a review of this area of research). Transferrin is a source of iron for *M. haemolytica,* and three outer membrane proteins of *M. haemolytica* involved in transferrin binding have been identified; a 70 kDa protein encoded by *tbpA*, a 105 kDa protein encoded by *tbpB*, and a 77 kDa protein for which we could not locate a reference gene sequence [34–36]. *M. haemolytica* also has two putative hemoglobin receptors encoded by *hmbR1* and *hmbR2* [37]. The ligand-gated channel gene specific to genotype *2 M. haemolytica* that encodes motifs suggesting iron, or iron containing compound transport across the outer membrane, does not match the gene sequences of *tbpA*, *tbpB*, *hmbR1*, or *hmbR2*. The projected size of the encoded protein based on 660 amino acids is 72.6 kDa, which does not entirely rule out the 77 kDa protein previously identified to bind transferrin, particularly if posttranslational modifications occur to the protein. Similar to the porin outer membrane protein specific to genotype 2, a single isoform of the ligand-gated channel gene predominated within the genotype 2 strains examined in this study.

Adhesion to epithelial cells is a vitally important component to *M. haemolytica* pathogenesis and a number of adhesin proteins have been identified for this species (reviewed in Confer and Ayalew 2018). YadA is a model representative of trimeric autotransporter adhesins [22], and YadA domains are used to identify trimeric autotransporter adhesins, as was done with the three adhesins identified in this study that have specificity to genotype *2 M. haemolytica*. YadA is a homotrimeric protein found in *Yersenia enterocolitica* and *Yersenia pseudotuberculosis* [38]. The individual protein strands consist of an N-terminal passenger domain and a C-terminal translocation unit/membrane anchor [38]. Three copies of the protein are transported across the outer membrane by type Vc secretion to create the functional homotrimeric adhesin, which has a lollipop-like structure with a globular head, extended stalk, and membrane anchor [22, 38]. YadA binds to collagen, fibronectin, and laminin components of the extracellular matrix, mucus, epithelial cells, macrophages, and to itself [38].

Two of the adhesin genes described in this study have been previously described as *ahsA* and *ahsB* in serotype 1 *M. haemolytica* strain SH1217 [39], (*ahsA* = adhesin genes A and *ahsB* = adhesin gene B). Of these two genes, *ahsA* was found in both genotypes 1 and 2 *M. haemolytica* in this study, whereas intact *ahsB* was found only in genotype 2 *M. haemolytica.* These two genes, which reside in close proximity to each other are expressed on a continuous transcript [39]. The encoded protein of *ahsA* was found to contain multiple collagen-binding motifs, as well as two neck motifs, and to be directly involved with collagen binding [39]. A chimeric protein consisting of the C-terminal domain of AhsB and the signal sequence of AhsA was shown to form trimers under standard denaturing conditions, and AhsB was proposed to be the cognate transporter for AhsA [39].

Adhesin B, D, and G genes show specificity for genotype 2 *M. haemolytica* in three distinct ways. The B gene is physically disrupted in genotype 1 strains with an insertional sequence, the D gene is disrupted in genotype 1 strains with a 65 bp deletion that induces a frame shift and multiple downstream stop codons, and the G gene resides on a genomic segment not found in genotype 1 strains. Similar to the other outer membrane proteins identified in this study with specificity to genotype 2 *M. haemolytica*, the isoform diversity of these proteins is quite low. However, they do not all appear essential to genotype 2 *M. haemolytica* viability, as a minor isoform of adhesin G in genotype 2 *M. haemolytica* contained a premature stop codon.

An RGD motif has been identified in extracellular matrix proteins including fibronectin, vitronectin, osteopontin, and lamin, and it is this motif that is involved with integrin binding as part of an adhesion and cell signaling process [40]. It is not known if this motif is looped out of the genotype 2 *M. haemolytica* outer membrane as part of the mature homotrimeric adhesin protein or has any role in epithelial cell adhesion involving B, D, or G proteins. It is also not known what the binding targets are for these proteins. Very recently, a report by Cozens et al reported that A1 strains of *M. haemolytica*, and not A2 strains, invade bovine bronchial epithelial cells through transcytosis, undergo rapid intracellular replication, and spread to adjacent cells, and that this mechanism might explain the proliferation of pathogenic *M. haemolytica* strains in animals versus commensal strains during times of stress [41]. It will be interesting to learn if the adhesins identified in this study, that are specific to pathogenic genotype 2 *M. haemolytica*, play a role in this newly identified process.

## Conclusions

In silico analyses of 69 *M. haemolytica* genomes sequenced in this study and additional sequences available in GenBank have identified seven OMP genes with high or complete specificity to genotype 2 *M. haemolytica* versus genotype 1. One of them, *AOMB-BD-CP*, appears to be truncated and to encode dysfunctional protein. The remaining six are highly conserved in genotype 2 *M. haemolytica* and collectively encode a peptidase S6, a porin, a ligated-gated channel protein, and three trimeric autotransporter adhesins. The expression of these genes, and the biological conditions that affect their expression, as well as the cell localization and abundance of their protein products, and the confirmed function of the protein products will be addressed in future studies. These genes, and the proteins they encode, may have important roles affecting the increased propensity of genotype 2 *M. haemolytica* to cause respiratory disease in cattle.

## Methods

### Strains and available sequences used in study

A total of 1144 *M. haemolytica* strains and/or their genomic sequences were used in this study. Sixty-nine strains were selected for long-read sequencing and de novo assembly of their chromosomes because they collectively represented all genotypes and subtypes characterized to date. Four genotype 1 and two genotype 2 strains with ambiguous subtype assignments were also sequenced to further resolve their phylogenetic placements. Some, or all of another 1062 *M. haemolytica* strains*,* which were also obtained from North American cattle, were used to obtain population-level sequence information on select genes or pseudogenes encoding a peptidase S6, porin, ligand-gated channel, and adhesins. The 1062 strains were previously sequenced with short-read technology and characterized into genotypes and subtypes without de novo assembly of their chromosome [16]. Additionally, 13 closed, circularized *M. haemolytica* chromosomes available in GenBank were also used to augment population level gene sequence information (See Additional file 1: Table S1 for information on all strains used in this study).

Of the 1144 strains involved in this study, 1131 were part of two collections (Zoetis and KSU-USMARC), and placed in one of four groups (lung clinical strains 1 (*n* = 155), lung clinical strains 2 (*n* = 162), nasopharyngeal non-clinical strains (*n* = 35), and clinical and non-clinical strains (*n* = 780), (Additional file 1: Table S1), [16]. The two groups of lung clinical strains 1 and 2 consisted of strains isolated from the lungs of clinical BRD cases from 35 U.S. States and 5 Canadian Provinces between 2002 and 2011 [16]. Members of the nasopharyngeal non-clinical group were isolated from the nasopharynx of cattle without signs of BRD from three US states in 2013 [16]. Members of the clinical and non-clinical group were isolated from either the lungs or nasopharynx of cattle with or without BRD, with close epidemiological relatedness between many members of the group [16]. The thirteen strains with publicly available closed chromosomes prior to this study all originated from cattle either with or without BRD, from five different US states and one unknown location (Additional file 1: Table S1). Twelve of them were not part of the Zoetis or KSU-USMARC collections.

### Strain culture, DNA purification, library construction and sequencing

The 69 strains selected for DNA sequencing were each streaked from frozen stocks onto chocolate agar plates containing 1% bovine hemoglobin and growth/nutrient supplements (Hardy Diagnostics, Santa Maria, CA, USA). They were passaged twice on chocolate agar at 37 °C with 5% CO_2_ for full revival from their frozen state. A single colony of each strain was either: 1) inoculated into 2 mL of Brain Heart Infusion (BHI) broth (pH 7.3), briefly cultured for several hours at 37 °C with shaking, transferred to 25–50 mL BHI broth and cultured with shaking to mid-logarithmic growth, or 2) inoculated directly into 25–50 mL of BHI broth and cultured with shaking to mid-logarithmic growth. A GENESYS 20 spectrophotometer (Thermo Fisher, Waltham, MA, USA) was used to assess growth. DNAs were extracted and purified from the cultured strains using Qiagen 100/G gravity-flow anion-exchange columns (Qiagen, Valencia, CA, USA) according to the manufacturer’s instructions with the following modifications: Bacteria pellets were suspended in buffer B1 containing 21 mM – 43 mM EDTA (pH 8.0) along with RnaseA (0.2 mg/mL) and initially incubated at 70 °C for 10 min [42]. The strains were then equilibrated in Buffer B1 at 37 °C and incubated for 10 min with lysozyme (2.28 mg/mL), followed by an additional incubation at 37 °C with proteinase K (0.57 mg/mL for 30 min. Additionally, for some strains, 4.7 mL of QBT buffer was added to the lysed cell suspensions immediately prior to column loading. DNA concentrations were determined with a Promega Quantus Fluorometer and QuantiFluor Dyes per the manufacturer’s instructions (Promega, Madison, WI, USA). In preparation of library construction, strain DNAs (~ 10 μg) were sheared to an approximate size of 20 kbp in a g-tube (Covaris, Woburn, MA, USA). Single molecule real-time DNA libraries (SMRT Bell 1.0, 10–20 kbp) were constructed from the DNAs of each strain according to the manufacturer’s instructions (Pacific Biosystems, Menio Park, CA, USA), and sequenced on a PacBio RS II sequencer with P6/C4 chemistry and movie run times of either 4 or 6 h. Subsequent to library construction for some strains, DNA fragments exceeding 20 kbp were purified from smaller fragments using a BluePippin according to the manufacturer’s instructions (Sage Science, Beverly, MA, USA).

### Whole chromosome assembly and annotation

Whole chromosome assembly was initiated with RS HGAP Assembly 3, which was part of the SMRT Analysis software package (version 2.3.0, Pacific Biosystems, Menio Park, CA, USA). An estimated chromosome size of 2.5 Mbp was used for the construction based on available *M. haemolytica* genomes in GenBank [13, 43], and to maximize usage of longer reads in the assembly. Resulting contigs that were close to, if not greater than 2.5 Mbp indicated that the entire chromosome was likely covered by sequence with redundancy at the unclosed ends. The contigs were annotated using Do It Yourself Annotation (DIYA) software [44] and imported into the Geneious software package (version R9 or R9.1), [45]. Redundant annotation coupled with identical sequence at contig ends were used to identify end overlaps which were edited accordingly. Geneious was also used to join some contigs representing segments of strain chromosomes where overlap of annotation and sequence between contig ends supported the connection. The predicted origin of replication was identified using Ori-Finder [46], and the contig sequences were edited to start at the predicted origin of replication in Geneious. The edited contigs were then polished and error corrected with PacBio sequence reads through the RS Resequencing.1 component of SMRT Analysis. Resulting error-corrected contigs were re-annotated with DIYA and imported in Geneious. There, they underwent additional error-correction through the mapping of previously run MiSeq Illumina library sequences for the corresponding strain [16]. This facilitated the identification and correction of homopolymer errors which can occur with the PacBio platform [47]. Fasta files of the finished chromosomes were submitted to GenBank for final annotation though the NCBI prokaryotic genome annotation pipeline [48].

### Whole genome analyses

Phylogenetic trees of core chromosome DNA shared between strains were generated in EDGAR 2.0 (http://edgar.computational.bio) [49] and with Parsnp (Harvest version 1.1.1) [50]. To construct the trees in EDGAR, core coding regions were computed and aligned using MUSCLE [51, 52]. Non-matching regions of the alignments were removed, and the remaining regions were concatenated into new alignments which were subsequently used as input in PHYLIP [53]. For tree construction in Parsnp, the software identified locally colinear blocks of multi-maximal unique matches which were used to anchor multiple alignments [50]. MUSCLE was used for sequence alignments [51, 52], with the final one containing all polymorphism, indel, and structural variation within the core genome. Polymorphisms passing filtering criteria for quality, possible recombination [54], and other parameters were run through FastTree 2 [55] for the generation of approximately maximum likelihood phylogenetic trees.

A phylogenetic tree was constructed in EDGAR from 1688 genes and 1,588,530 nucleotide residues of core chromosome DNA sequence shared between all 69 sequenced strains, and the additional 13 fully assembled *M. haemolytica* strain chromosomes available in GenBank (Additional file 1: Table S1). Trees were also generated in EDGAR between the 35 genotype 1 sequenced strains, plus one additional fully assembled genotype 1 *M. haemolytica* strain chromosome available in GenBank (Additional file 1: Table S1), based on a core of 2059 genes and 1,915,615 nucleotide residues, and between the 34 genotype 2 sequenced strains, plus 11 additional fully assembled genotype *2 M. haemolytica* chromosomes available in GenBank, based on a core of 1917 genes and 1,798,710 nucleotide residues. The 13 previously sequenced strains available in GenBank were placed into or between genotypes and subtypes based on their chromosome position in the trees, and from the identification of subtype specific single nucleotide polymorphisms [16]. Similarly, the four genotype 1 and two genotype 2 strains included in this study with ambiguous subtype assignments [16], were placed into subtypes based on their chromosome position in the trees.

Two phylogenetic trees were constructed in Parsnp and viewed in Gingr. The first consisted of the 35 genotype 1 *M. haemolytica* sequenced strains, plus the one additional fully assembled genotype 1 chromosome available in GenBank (Additional file 1: Table S1). The genome of a genotype 1 strain sequenced in this study (GenBank# CP017502) was used as a reference in the build. Prior to construction of the tree, 19,337 bases of sequence contained between direct repeats were blocked from the build. The repeats comprise the integration site of ICE*Mh1* which is a member of highly related ICEs known to reside in genotype 2 *M. haemolytica* [16]. The tree was constructed with core genome equaling 81% of the GenBank# CP017502 genome.

The second tree consisted of the 34 genotype 2 *M. haemolytica* strains sequenced in this study plus the 11 additional fully assembled genotype 2 chromosomes available in GenBank (Additional file 1: Table S1). For this tree, the genome of a genotype 2 strain available in GenBank (#CP004752) was used as a reference. Prior to construction of the tree, 55,115 bases of ICE sequence contained between direct repeats were blocked from the build. The tree was constructed with core genome equaling 76% of the GenBank# CP004752 genome.

In addition to constructing phylogenetic trees, EDGAR was used to calculate a pan genome of the 69 strains sequenced in this study, as well as a set of genes with specificity to: 1) all 69 strains, 2) all 35 genotype 1 strains, and 3) all 34 genotype 2 strains. Previously sequenced and annotated *M. haemolytica* strain chromosomes available in GenBank prior to this study were not included for those analyses to avoid disparate results that were the artificial consequence of different annotation methods or reference databases.

The 69 strains sequenced in this study, along with the 13 *M. haemolytica* strains with fully assembled chromosomes available in GenBank were all molecularly serotyped through in silico use of a recently developed multiplex PCR assay [14]. This assay amplifies unique *M. haemolytica* gene segments associated with capsular polysaccharide synthesis. The resulting amplicons are used to molecularly assign *M. haemolytica* to serotypes 1, 2, or 6. The 82 genomes were queried for all primer sequences used in the assay. Those genomes with sequence regions that matched primer pairs were then checked for strand orientation and anticipated amplicon size to determine the serotype score from the assay.

### Identification of OMP encoding genes with specificity to genotype 2 *M. haemolytica*

Within the set of genes identified by EDGAR as specific to all 34 genotype 2 strains sequenced in this study, searches were conducted to identify genes encoding OMPs. Translated protein sequences of the entire set of genes were used from the genotype 2 subtype representative strains: 2b (GenBank# CP017538), 2c (GenBank# CP017491), 2d (GenBank# CP017505), and 2e (GenBank# CP017552). The proteins were analyzed with four software programs that identified amphipathic beta-strands typical of OMPs, including HHomp (http://toolkit.tuebingen.mpg.de/hhomp), [56], PRED-TMBB (http://bioinformatics.biol.uoa.gr/PRED-TMBB), [57], PRED-TMBB2 (http://www.compgen.org/tools/PRED-TMBB2), [58], and Boctopus 2 (http://boctopus.bioinfo.se) [59]. The software PRED-TAT [60], which was part of the PRED-TMBB2 software package, was also used to predict signal peptide regions within protein sequences. Additionally, a number of related adhesins were found to encode variants specific to genotype 2 strains through annotation searches, genome inspections and/or alignments of the 69 strains sequenced in this study using Geneious. The genotype 2 adhesin proteins were run through the four OMP prediction programs and PRED-TAT.

### Identification of determinants conferring genotype 2 *M. haemolytica* OMP specificity

Genotype 1 subtype representative strains (*n* = 5) and the genotype 2 subtype representative strains described above (*n* = 4) were used to 1) confirm EDGAR identifications of genotype 2 specific genes that were additionally predicted to encoded OMPs by all four prediction software programs, 2) identify the probable determinants that conferred genotype 2 OMP gene specificity, and 3) identify unique regions within genotype 2 specific OMPs that were not shared by other proteins with similar function. The five genotype 1 subtype representative strains included; 1b (GenBank# CP017495), 1c (GenBank# CP017510), 1e (GenBank# CP017502), 1f (GenBank# CP017484), and 1i (GenBank# CP017499). For each of the genotype 1 and 2 subtype representative strains, protein sequences of all genes within their genome annotated identically as one identified as specific to genotype 2 *M. haemolytica,* that was also predicted to encode an outer membrane protein, were aligned. Additionally, the proteins of genes with similar annotations were also included in the alignments in some cases. Genome alignments or sequence searches of the genotype 1 subtype representative strains were also used to confirm that an intact homolog of a genotype 2 specific gene was not detectable within their genomes.

### Assessment of protein isoform diversity of genotype 2 specific adhesins, peptidase S6, porin, and a signal-ligand

Previously sequenced Illumina genomic DNA libraries of up to 1062 *M. haemolytica* strains (NCBI Sequence Read Archive # SRP078075) were individually mapped to reference genomes generated in this study to obtain adhesin, peptidase S6, porin and signal-ligand gene sequences of the strains (Additional file 1: Table S1). The libraries and reference genomes were matched by genotypes and subtypes where necessary to improve mapping quality. The reads were mapped in Geneious, visually checked, and corresponding gene sequences were extracted and translated into protein sequences. Adhesin gene sequences were manually extracted and translated into protein sequence only from the 69 genomes sequenced in this study, the 13 available fully sequenced *M. haemolytica* chromosomes available in GenBank, and from the mapped MiSeq library reads of 302 genotype 2 strains comprising the Zoetis collection, and additional genotype 2 strains of the KSU-USMARC collection. Alignments of genes variants or protein isoforms were made in either MacVector 15.5.3, or Geneious.

## Supplementary information

**Additional file 1: Table S1.** Strains and/or strain sequences used in this study and information on their genotype, subtype, molecular serotype, and isoform sequence encoded by the genes found to be specific for genotype 2 *M. haemolytica.* Strains and/or strain sequences used in this study and information on their genotype, subtype, molecular serotype, and isoform sequence encoded by the genes found to be specific for genotype 2 *M. haemolytica.*

**Additional file 2: Table S2.** Pan genome of 69 *M. haemolytica* strains. This table lists all the genes comprising the pan genome of the 69 strains along with their locus tag and product for each strain that has them.

**Additional file 3: Table S3.** The core genome of 69 *M. haemolytica* strains. This table lists all the genes comprising the core genome of the 69 strains along with their locus tag and product for each strain.

**Additional file 4: Table S4.** Genes with specificity to genotype 1 *M. haemolytica*. This table lists all the genes observed only in genotype 1 strains along with their locus tag and product for each strain.

**Additional file 5: Table S5.** Genes specific to genotype 2 *M. haemolytica* and outer membrane protein predictions. This table lists all of the genes observed only in genotype 2 strains as identified by EDGAR. It also gives outer membrane localization/Beta barrel probability scores for Hhomp, PRED-TMBB, PRED-TMBB2 and Boctopus2, as well as predicted signal peptide detection for strains with the following GenBank numbers: CP017538 (2b), CP017491 (2c), CP017505 (2d), CP017552 (2e).

**Additional file 6: Figure S1.** Alignment of genotypes 1 and 2 peptidase S6 and S6 family IgA-specific metalloendopeptidase gene and pseudogene encoded proteins. Represented in the alignment are four peptidase S6 proteins from five genotype 1 strains that are each of a different subtype, two peptidase S6 proteins from four genotype 2 strains that are each of a different subtype, and two proteins from the same four genotype 2 strains that originated from genes annotated as S6 family IgA-specific metalloendopeptidase proteins. Areas of 51% chemical identity or greater are indicated with grey boxes within the alignment. The peptidase S6 proteins originating from a gene flagged for specificity to genotype 2 *M. haemolytica* by EDGAR software is annotated as “Genotype 2 specific*”. The most closely related genotype 1 protein to the genotype 2 specific peptidase S6, encoded by a pseudogene, is annotated within the alignment. Regarding the closely related genotype 1 protein, the end of the peptide, which does not contain a corresponding gene sequence stop codon, is denoted with an arrow in the alignment. Extended translation to a stop codon is shown with sequence above the alignment.

**Additional file 7: Figure S2.** Alignment of all detected isoforms of the peptidase S6 gene specific to genotype 2 *M. haemolytica* and those encoded by a homologous pseudogene in genotype 1 *M. haemolytica.* The alignment shows all five isoforms of the peptidase S6 specific to genotype 2 *M. haemolytica* that were detected, and all four of the peptidase S6 homologs in genotype 1 *M. haemolytica* which all contained a premature stop codon. Isoform numbers in this figure correspond to the isoform numbers listed in Additional file 1: Table S1. *184 iso 1 is an abbreviation for USDA-ARS-USMARC-184 isoform 1. Areas of 51% chemical identity or greater are indicated with grey boxes.

**Additional file 8: Figure S3.**. Alignment of autotransporter outer membrane beta-barrel domain-containing protein (AOMB-BP-CP) isoforms. The alignment contains all annotated AOMB-BP-CP proteins from five genotype 1 and four genotype 2 *M. haemolytica* strains that are each of a different subtype. The AOMB-BP-CP proteins flagged by EDGAR as specific to genotype 2 *M. haemolytica* are indicated with an asterisk. Areas of 51% chemical identity or greater are indicated with grey boxes.

**Additional file 9: Figure S4.** Alignment of genotype 2 AOMB-BP-CP proteins flagged as specific to genotype 2 *M. haemolytica* with genotype 1 peptidase S6 protein and genotype 2 S6 family IgA-specific metalloendopeptidase protein. The alignment contains peptidase S6 protein sequence from five genotype 1 strains of different subtypes, S6 family IgA-specific metalloendopeptidase protein sequence from four genotype 2 strains of different subtypes, and AOMB-BP-CP protein sequence from four genotype 2 strains of different subtypes. The AOMB-BP-CP proteins flagged by EDGAR as specific to genotype 2 *M. haemolytica* are denoted by an asterisk. The alignment additionally contains translated nucleotides immediately upstream of, in-frame, and concatenated to the AOMB-BP-CP protein sequence for each of the genotype 2 strains representing different subtypes. The black arrow highlights the location of a stop codon in the concatenated sequences. Areas of greater than 51% chemical identity are indicated with grey boxes.

**Additional file 10: Figure S5.** Alignment of all annotated porin-like proteins in five genotype 1 and four genotype 2 *M. haemolytica* strains that are each of a different subtype. The proteins flagged by EDGAR as specific to genotype 2 *M. haemolytica* are indicated within the alignment with an asterisk. The site of premature stop codons within proteins encoded by a pseudogene homolog of the genotype 2 specific porin is highlighted with an arrow. Areas of 51% chemical identity or greater are indicated with grey boxes.

**Additional file 11: Figure S6.** Alignment of ligand-gated channel proteins in five genotype 1 and four genotype 2 *M. haemolytica* strains that are each of a different subtype. Within the alignment, proteins encoded by the ligand-gated channel gene flagged by EDGAR as specific to genotype 2 *M. haemolytica* are labelled with an asterisk. The stop codon site within the genotype 1 proteins that are encoded by a pseudogene is highlighted with an arrow. Areas of 51% chemical identity or greater are indicated with grey boxes within the alignment.

**Additional file 12: Figure S7.** Alignment of all detected isoforms of the ligand-gated channel specific to genotype 2 *M. haemolytica* and those of the homologous pseudogene in genotype 1 *M. haemolytica.* Within the alignment, sequences corresponding to the major isoform of genotype 1 and the major isoform of genotype 2 are highlighted with brackets. *184 isoform 1 is an abbreviation for USDA-ARS-USMARC-184 isoform 1 (ligand-gated channel). Areas of 51% chemical identity or greater are indicated with grey boxes within the alignment.

**Additional file 13: Figure S8.** Alignment of genotype 1 pseudogene adhesin B1 and genotype 2 adhesin B isoform sequences. Represented within the alignment are pseudogene B1 adhesin isoforms from five genotype 1 strains that are each of a different genotype, and B adhesin isoforms from four genotype 2 strains that are also each of a different subtype. Areas of 51% chemical identity or greater are indicated with grey boxes.

**Additional file 14: Figure S9.** Alignment of pseudogene adhesin B2 isoforms from five genotype 1 strains that are each of a different genotype and adhesin B isoforms from four genotype 2 strains that are also each of a different subtype. The alignment contains pseudogene adhesin B2 isoforms from five genotype 1 strains that are each of a different genotype and adhesin B isoforms from four genotype 2 strains that are also each of a different subtype. Areas of 51% chemical identity or greater are indicated with grey boxes.

**Additional file 15: Figure S10.** Alignment adhesin D isoforms from five genotype 1 strains and four genotype 2 strains that are each of a different subtype. The alignment contains adhesin D isoforms from five genotype 1 strains and four genotype 2 strains that are each of a different subtype. Areas of 51% chemical identity or greater are indicated with grey boxes.

**Additional file 16: Figure S11.** Alignment of adhesin D isoforms observed in genotype 2 *M. haemolytica* strains and one genotype 1 strain. The alignment contains adhesin D isoforms observed in genotype 2 *M. haemolytica* strains and one genotype 1 strain. Areas of 51% chemical identity or greater are indicated with grey boxes.

**Additional file 17: Figure S12.** Alignment of adhesin B isoforms observed in genotype 2 *M. haemolytica*. The alignment contains adhesin B isoforms observed in genotype 2 *M. haemolytica*. Within it, the arrow points to the site where a single genotype 2 strain (GenBank# CP017521) has a stop codon consistent with genotype 1 strains. Prior to the stop codon, the adhesin B sequence for GenBank# CP017521 is an exact match with Gen 2 isoform 1. Areas of 51% chemical identity or greater are indicated with grey boxes.

**Additional file 18: Figure S13.** Alignment of adhesin G isoforms observed in genotype 2 *M. haemolytica* strains. The alignment contains adhesin G isoforms observed in genotype 2 *M. haemolytica* strains. Areas of 51% chemical identity or greater are indicated with grey boxes.

**Additional file 19: Figure S14.** Alignment of all unique genotype 2 adhesin B, D, and G isoforms observed in this study. The alignment contains all unique genotype 2 adhesin B, D, and G isoforms observed in this study. The one genotype 2 “like” adhesin G isoform observed in a genotype 1 strain (GenBank# CP0175216) is also included in the alignment. The conserved RGD motif is highlighted with a bracket. Areas of 51% chemical identity or greater are indicated with grey boxes.

## Data Availability

The genomes sequenced in this study have been placed in GenBank (CP017484-CP017552). All additionally used GenBank and/or Sequence Read Archive accession files are listed in Supplementary Table #1.

## Abbreviations

AOMB-BD-CP: Autotransporter outer membrane beta-barrel domain-containing protein
ARS: Agricultural Research Service
BHI: Brain Heart Infusion
BRD: Bovine respiratory disease
DIYA: Do It Yourself Annotation
ICE: Integrative and conjugative element
ICE*Mh1*: ICE identified in *M. haemolytica*
*M. haemolytica*: *Mannheimia haemolytica*
OMPS: Outer membrane proteins
RGD: Arginine, Glycine, Aspartate
SMRT: Single molecule real-time
USDA: United States Department of Agriculture
