# Supplementary material for: Differences between predicted outer membrane proteins of genotype 1 and 2 Mannheimia haemolytica
Source: BMC Microbiol. 2020 Aug 12;20:250. doi: 10.1186/s12866-020-01932-2 (PMC7424683; doi:10.1186/s12866-020-01932-2)
Supplement: Supplementary file 6 — Additional file 6: Figure S1. Alignment of genotypes 1 and 2 peptidase S6 and S6 family IgA-specific metalloendopeptidase gene and pseudogene encoded proteins. Represented in the alignment are four peptidase S6 proteins from five genotype 1 strains that are each of a different subtype, two peptidase S6 proteins from four genotype 2 strains that are each of a different subtype, and two proteins from the same four genotype 2 strains that originated from genes annotated as S6 family IgA-specific metalloendopeptidase proteins. Areas of 51% chemical identity or greater are indicated with grey boxes within the alignment. The peptidase S6 proteins originating from a gene flagged for specificity to genotype 2 M. haemolytica by EDGAR software is annotated as “Genotype 2 specific*”. The most closely related genotype 1 protein to the genotype 2 specific peptidase S6, encoded by a pseudogene, is annotated within the alignment. Regarding the closely related genotype 1 protein, the end of the peptide, which does not contain a corresponding gene sequence stop codon, is denoted with an arrow in the alignment. Extended translation to a stop codon is shown with sequence above the alignment. [file 12866_2020_1932_MOESM6_ESM.pdf]

Fig S1

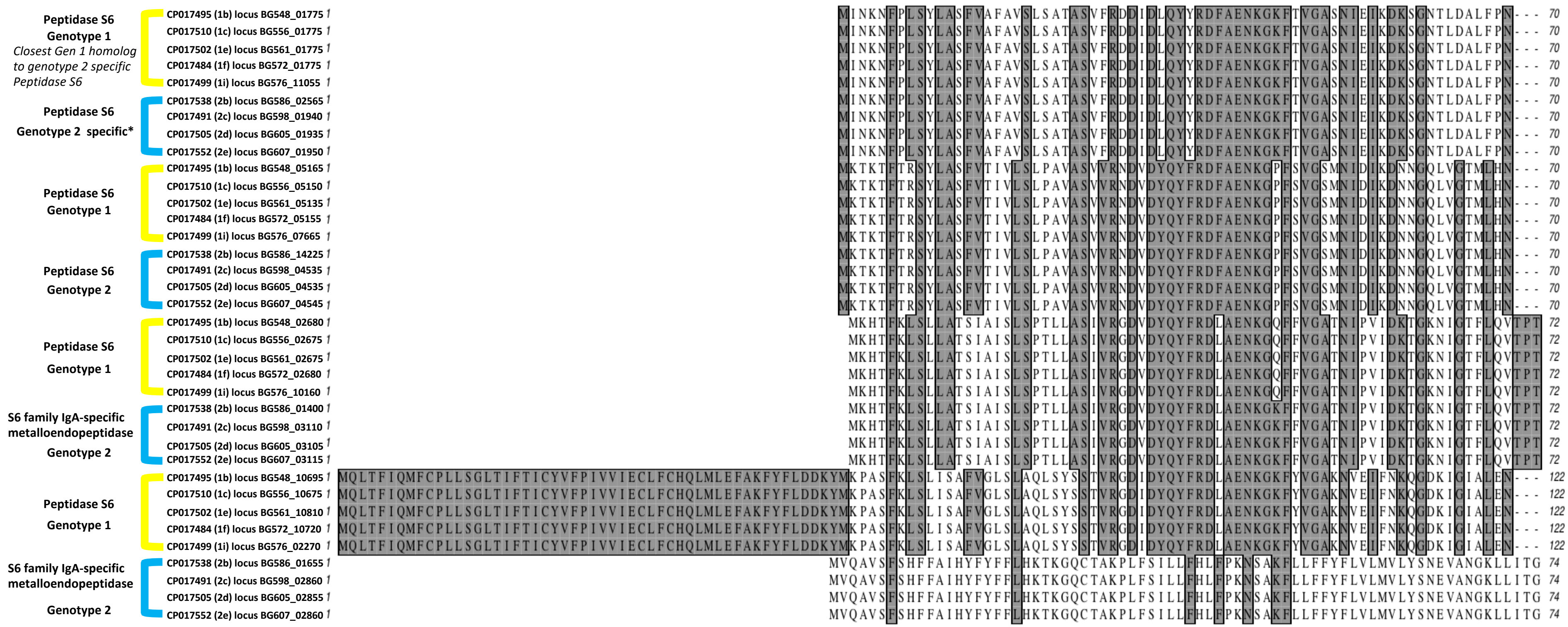

Peptidase S6 Genotype 1
Closest Gen 1 homolog to genotype 2 specific Peptidase S6
Peptidase S6 Genotype 2 specific*
Peptidase S6 Genotype 1
Peptidase S6 Genotype 2
Peptidase S6 Genotype 1
S6 family IgA-specific metalloendopeptidase Genotype 2
Peptidase S6 Genotype 1
S6 family IgA-specific metalloendopeptidase Genotype 2
CP017495 (1b) locus BG548_01775 MINKNFPLSYLASFVAFAVSLSATASVFRDDIDLQYYRDFAENKGKFTVGASNIEIKDKSGNTLDALFPN--- 70
CP017510 (1c) locus BG556_01775 MINKNFPLSYLASFVAFAVSLSATASVFRDDIDLQYYRDFAENKGKFTVGASNIEIKDKSGNTLDALFPN--- 70
CP017502 (1e) locus BG561_01775 MINKNFPLSYLASFVAFAVSLSATASVFRDDIDLQYYRDFAENKGKFTVGASNIEIKDKSGNTLDALFPN--- 70
CP017484 (1f) locus BG572_01775 MINKNFPLSYLASFVAFAVSLSATASVFRDDIDLQYYRDFAENKGKFTVGASNIEIKDKSGNTLDALFPN--- 70
CP017499 (1i) locus BG576_11055 MINKNFPLSYLASFVAFAVSLSATASVFRDDIDLQYYRDFAENKGKFTVGASNIEIKDKSGNTLDALFPN--- 70
CP017538 (2b) locus BG586_02565 MINKNFPLSYLASFVAFAVSLSATASVFRDDIDLQYYRDFAENKGKFTVGASNIEIKDKSGNTLDALFPN--- 70
CP017491 (2c) locus BG598_01940 MINKNFPLSYLASFVAFAVSLSATASVFRDDIDLQYYRDFAENKGKFTVGASNIEIKDKSGNTLDALFPN--- 70
CP017505 (2d) locus BG605_01935 MINKNFPLSYLASFVAFAVSLSATASVFRDDIDLQYYRDFAENKGKFTVGASNIEIKDKSGNTLDALFPN--- 70
CP017552 (2e) locus BG607_01950 MINKNFPLSYLASFVAFAVSLSATASVFRDDIDLQYYRDFAENKGKFTVGASNIEIKDKSGNTLDALFPN--- 70
CP017495 (1b) locus BG548_05165 MKTKTFTRSYLASFVTIVLSLPAVASVVRNDVDYQYFRDFAENKGPFSVGSMNIDIKDNNGQLVGTMLHN--- 70
CP017510 (1c) locus BG556_05150 MKTKTFTRSYLASFVTIVLSLPAVASVVRNDVDYQYFRDFAENKGPFSVGSMNIDIKDNNGQLVGTMLHN--- 70
CP017502 (1e) locus BG561_05135 MKTKTFTRSYLASFVTIVLSLPAVASVVRNDVDYQYFRDFAENKGPFSVGSMNIDIKDNNGQLVGTMLHN--- 70
CP017484 (1f) locus BG572_05155 MKTKTFTRSYLASFVTIVLSLPAVASVVRNDVDYQYFRDFAENKGPFSVGSMNIDIKDNNGQLVGTMLHN--- 70
CP017499 (1i) locus BG576_07665 MKTKTFTRSYLASFVTIVLSLPAVASVVRNDVDYQYFRDFAENKGPFSVGSMNIDIKDNNGQLVGTMLHN--- 70
CP017538 (2b) locus BG586_14225 MKTKTFTRSYLASFVTIVLSLPAVASVVRNDVDYQYFRDFAENKGPFSVGSMNIDIKDNNGQLVGTMLHN--- 70
CP017491 (2c) locus BG598_04535 MKTKTFTRSYLASFVTIVLSLPAVASVVRNDVDYQYFRDFAENKGPFSVGSMNIDIKDNNGQLVGTMLHN--- 70
CP017505 (2d) locus BG605_04535 MKTKTFTRSYLASFVTIVLSLPAVASVVRNDVDYQYFRDFAENKGPFSVGSMNIDIKDNNGQLVGTMLHN--- 70
CP017552 (2e) locus BG607_04545 MKTKTFTRSYLASFVTIVLSLPAVASVVRNDVDYQYFRDFAENKGPFSVGSMNIDIKDNNGQLVGTMLHN--- 70
CP017495 (1b) locus BG548_02680 MKHTFKLSLLATSIAISLSPTLLASIVRGDVDYQYFRDLAENKGQFFVGATNIPVIDKTGKNIGTFLQVTPT 72
CP017510 (1c) locus BG556_02675 MKHTFKLSLLATSIAISLSPTLLASIVRGDVDYQYFRDLAENKGQFFVGATNIPVIDKTGKNIGTFLQVTPT 72
CP017502 (1e) locus BG561_02675 MKHTFKLSLLATSIAISLSPTLLASIVRGDVDYQYFRDLAENKGQFFVGATNIPVIDKTGKNIGTFLQVTPT 72
CP017484 (1f) locus BG572_02680 MKHTFKLSLLATSIAISLSPTLLASIVRGDVDYQYFRDLAENKGQFFVGATNIPVIDKTGKNIGTFLQVTPT 72
CP017499 (1i) locus BG576_10160 MKHTFKLSLLATSIAISLSPTLLASIVRGDVDYQYFRDLAENKGQFFVGATNIPVIDKTGKNIGTFLQVTPT 72
CP017538 (2b) locus BG586_01400 MKHTFKLSLLATSIAISLSPTLLASIVRGDVDYQYFRDLAENKGKFFVGATNIPVIDKTGKNIGTFLQVTPT 72
CP017491 (2c) locus BG598_03110 MKHTFKLSLLATSIAISLSPTLLASIVRGDVDYQYFRDLAENKGKFFVGATNIPVIDKTGKNIGTFLQVTPT 72
CP017505 (2d) locus BG605_03105 MKHTFKLSLLATSIAISLSPTLLASIVRGDVDYQYFRDLAENKGKFFVGATNIPVIDKTGKNIGTFLQVTPT 72
CP017552 (2e) locus BG607_03115 MKHTFKLSLLATSIAISLSPTLLASIVRGDVDYQYFRDLAENKGKFFVGATNIPVIDKTGKNIGTFLQVTPT 72
CP017495 (1b) locus BG548_10695 MQLTFIQMFCPLLSGLTIFTICYVFPIVVIECLFCHQLMLEFAKFYFLDDKYMKPASFKLSLISAFVGLSLAQLSYSSTVRGDIDYQYFRDLAENKGKFYVGAKNVEIFNKQGDKIGIALEN--- 122
CP017510 (1c) locus BG556_10675 MQLTFIQMFCPLLSGLTIFTICYVFPIVVIECLFCHQLMLEFAKFYFLDDKYMKPASFKLSLISAFVGLSLAQLSYSSTVRGDIDYQYFRDLAENKGKFYVGAKNVEIFNKQGDKIGIALEN--- 122
CP017502 (1e) locus BG561_10810 MQLTFIQMFCPLLSGLTIFTICYVFPIVVIECLFCHQLMLEFAKFYFLDDKYMKPASFKLSLISAFVGLSLAQLSYSSTVRGDIDYQYFRDLAENKGKFYVGAKNVEIFNKQGDKIGIALEN--- 122
CP017484 (1f) locus BG572_10720 MQLTFIQMFCPLLSGLTIFTICYVFPIVVIECLFCHQLMLEFAKFYFLDDKYMKPASFKLSLISAFVGLSLAQLSYSSTVRGDIDYQYFRDLAENKGKFYVGAKNVEIFNKQGDKIGIALEN--- 122
CP017499 (1i) locus BG576_02270 MQLTFIQMFCPLLSGLTIFTICYVFPIVVIECLFCHQLMLEFAKFYFLDDKYMKPASFKLSLISAFVGLSLAQLSYSSTVRGDIDYQYFRDLAENKGKFYVGAKNVEIFNKQGDKIGIALEN--- 122
CP017538 (2b) locus BG586_01655 MVQAVSFSHFFAIHYFYFFLHKTKGQCTAKPLFSILLFHLFPKNSAKFLLFFYFLVLMVLYSNEVANGKLLITG 74
CP017491 (2c) locus BG598_02860 MVQAVSFSHFFAIHYFYFFLHKTKGQCTAKPLFSILLFHLFPKNSAKFLLFFYFLVLMVLYSNEVANGKLLITG 74
CP017505 (2d) locus BG605_02855 MVQAVSFSHFFAIHYFYFFLHKTKGQCTAKPLFSILLFHLFPKNSAKFLLFFYFLVLMVLYSNEVANGKLLITG 74
CP017552 (2e) locus BG607_02860 MVQAVSFSHFFAIHYFYFFLHKTKGQCTAKPLFSILLFHLFPKNSAKFLLFFYFLVLMVLYSNEVANGKLLITG 74

Fig S1 continued

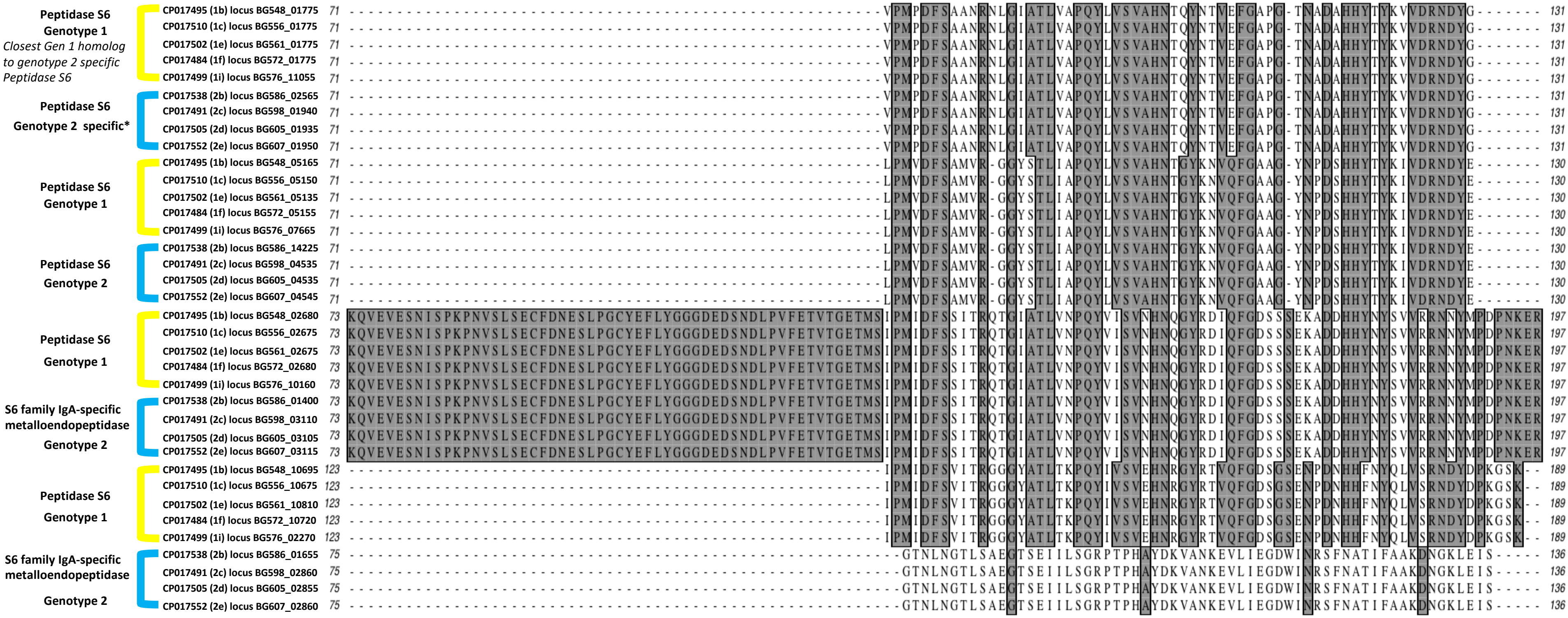

Fig S1 continued

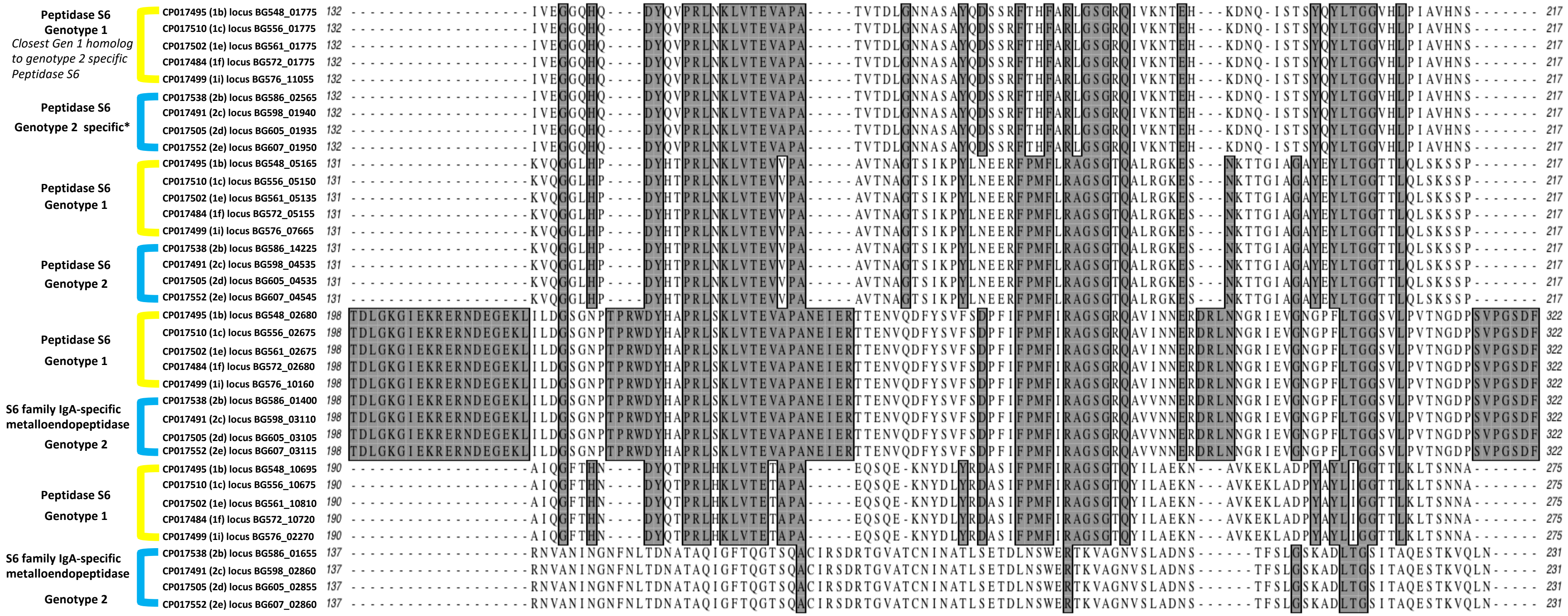

Fig S1 continued

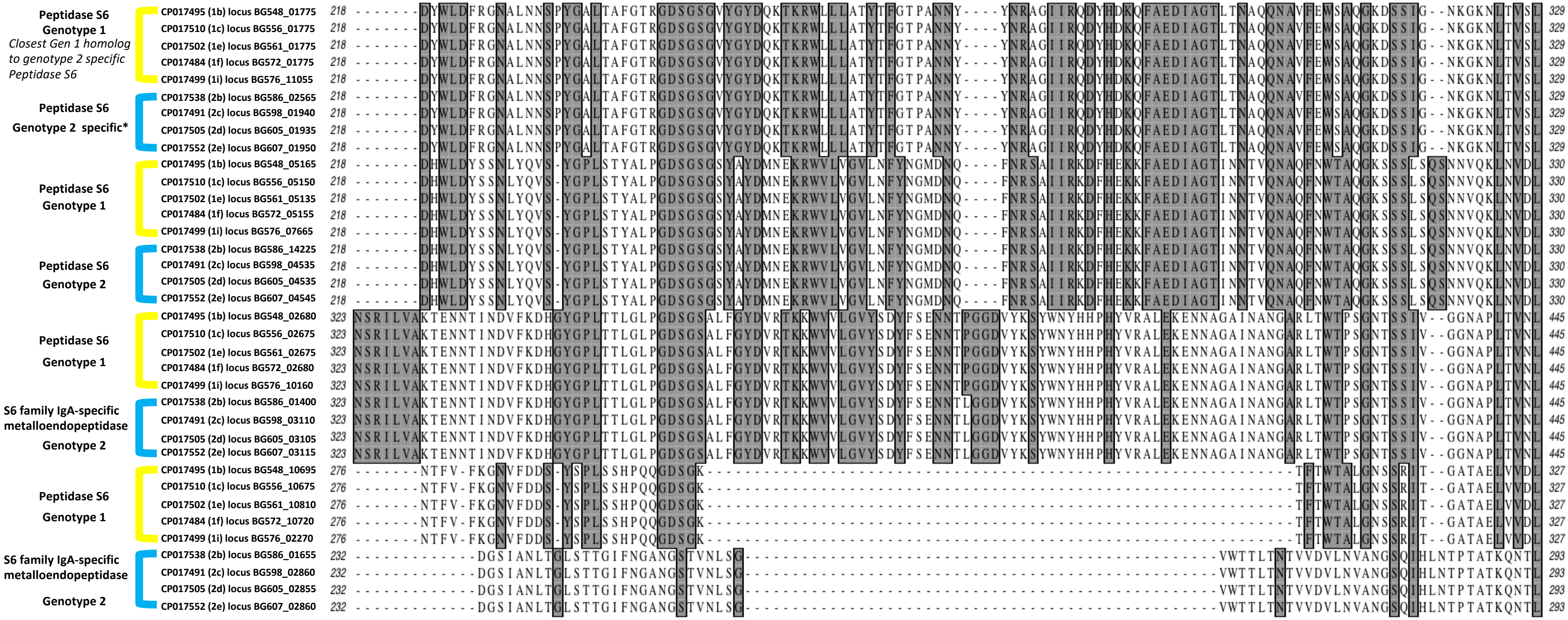

Fig S1 continued

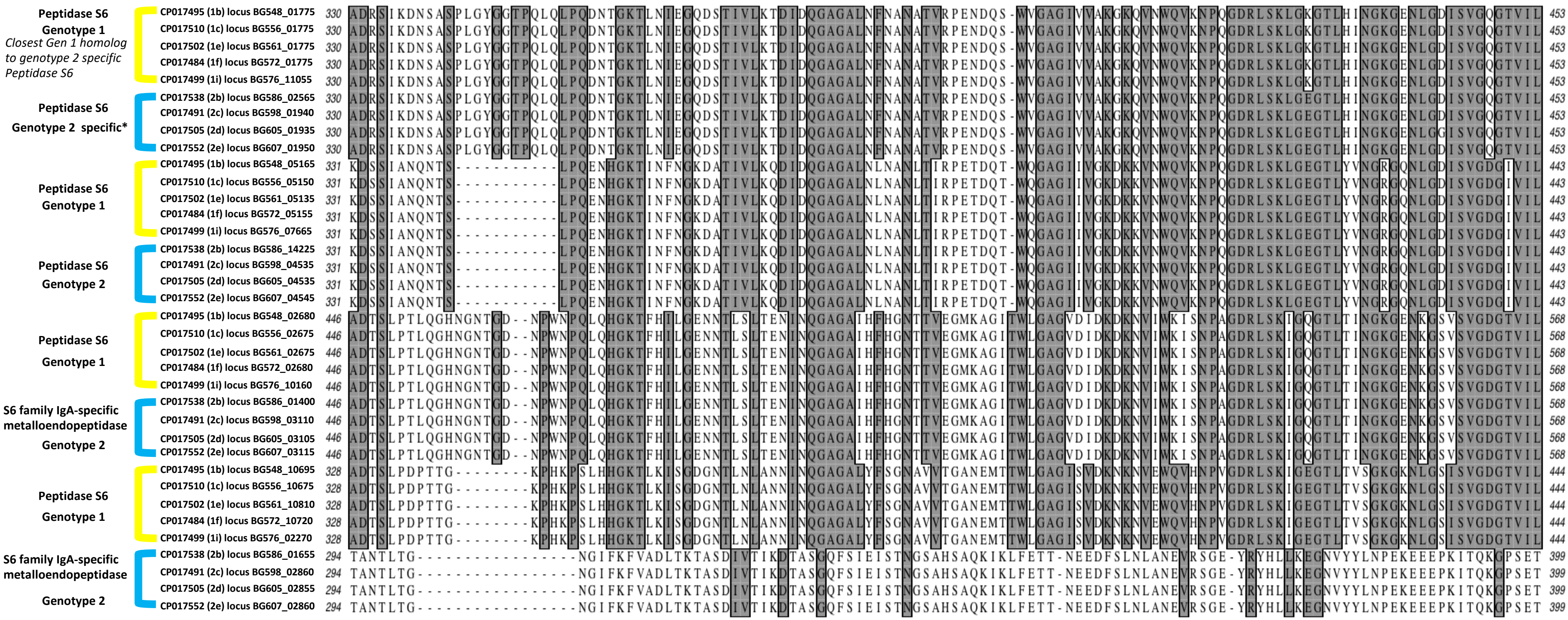

Fig S1 continued

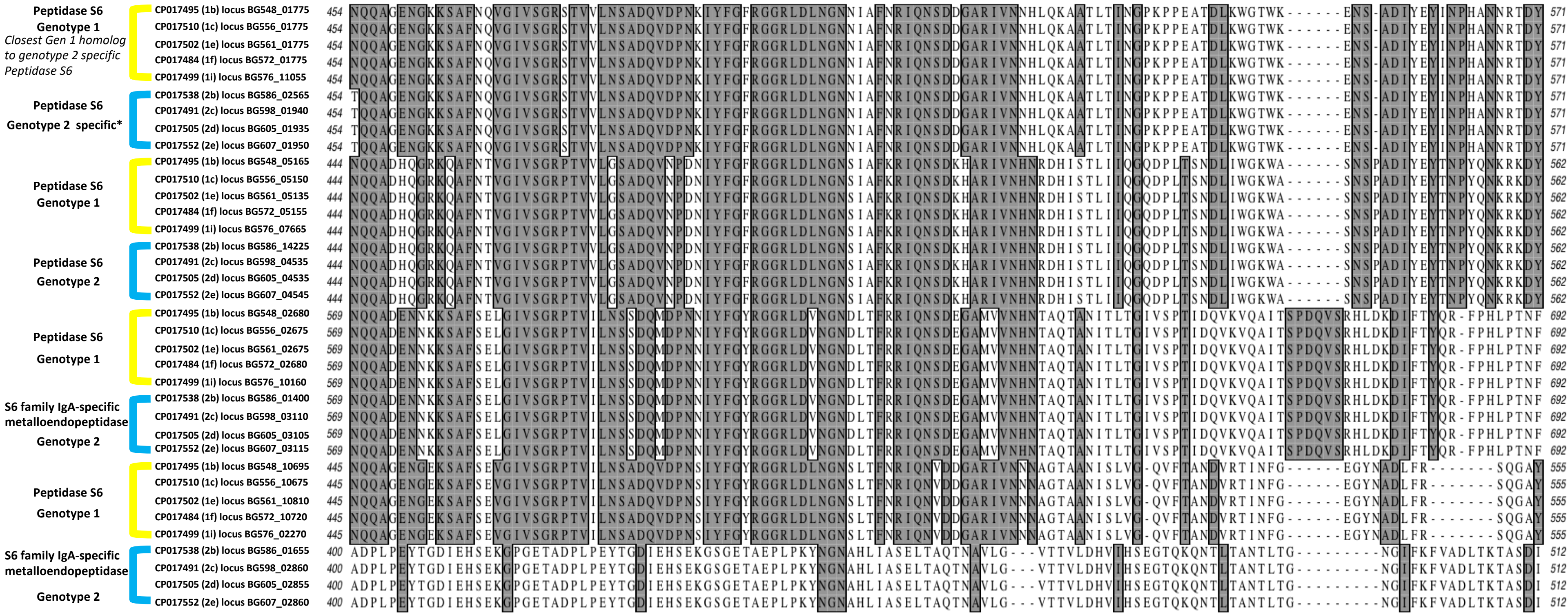

Fig S1 continued

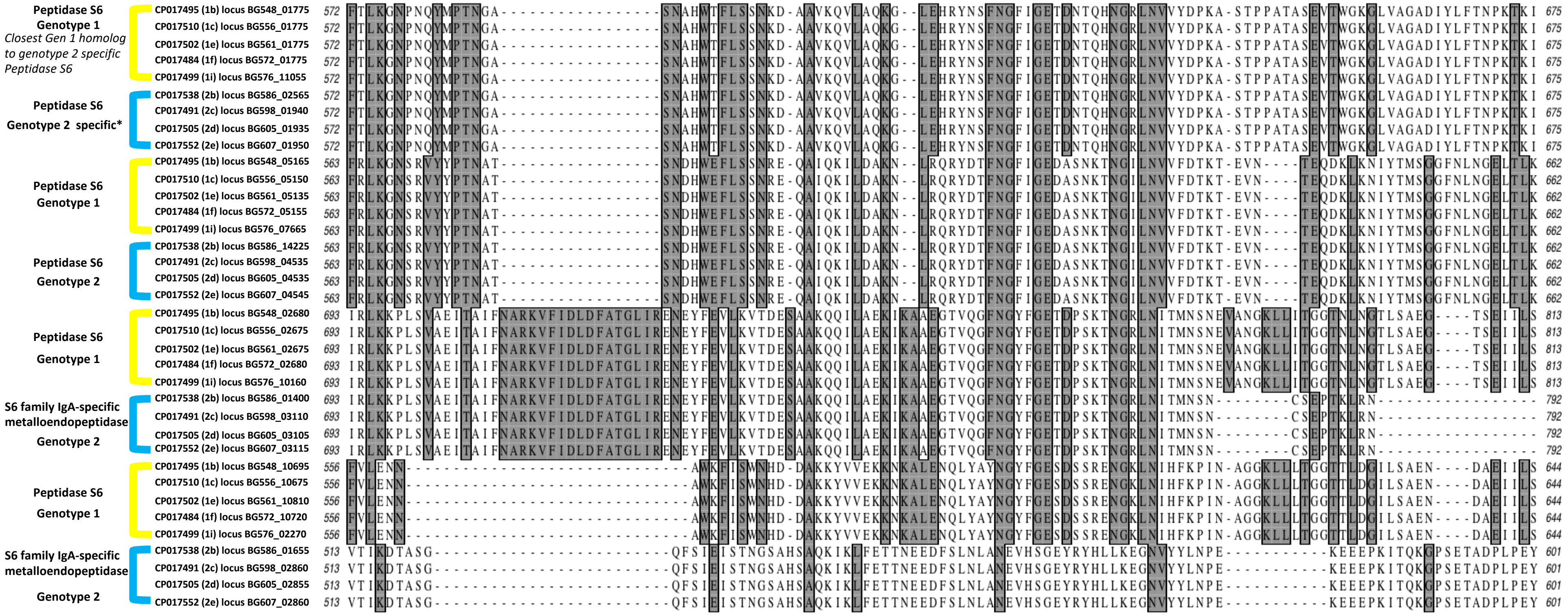

Fig S1 continued

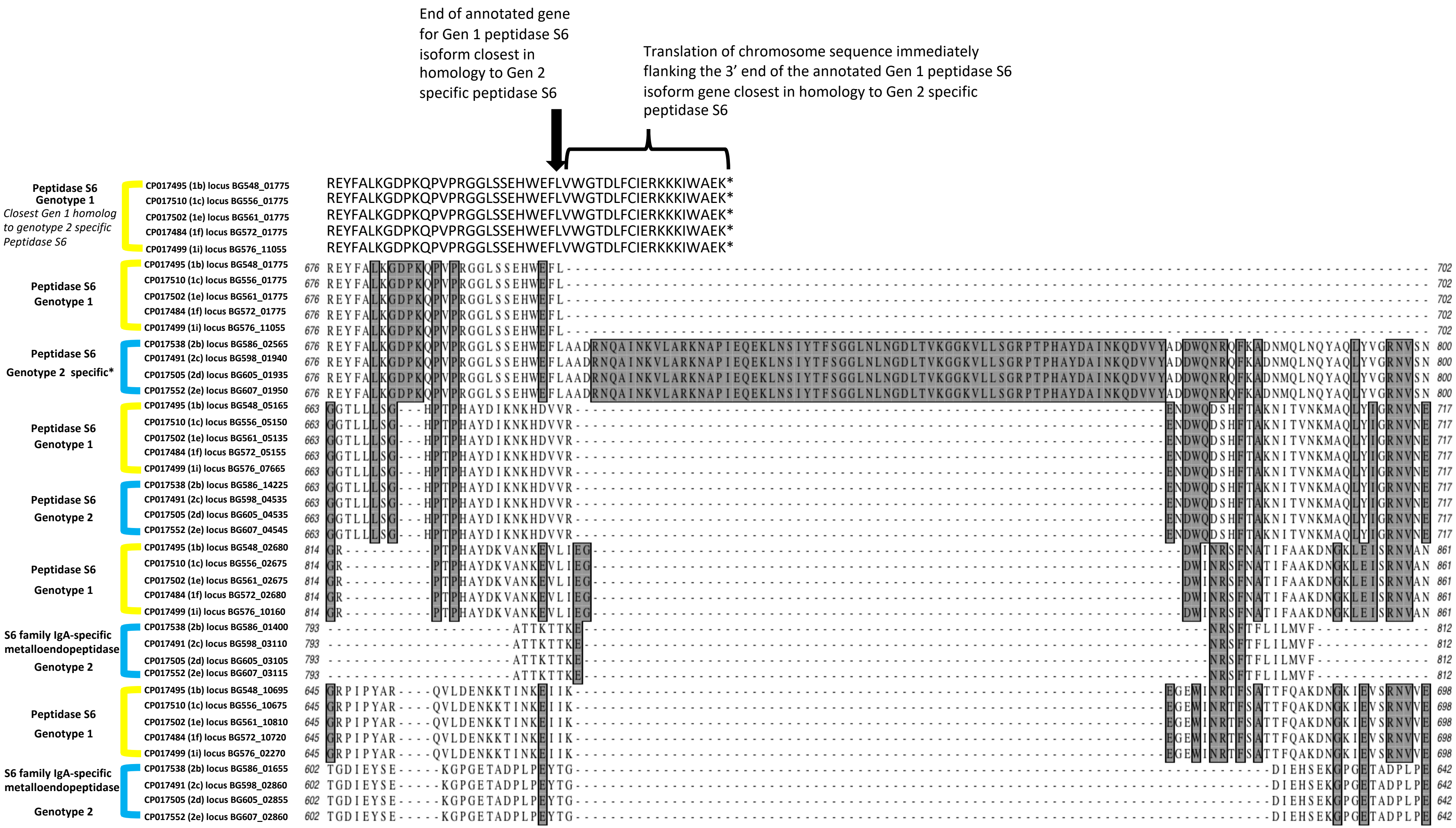

Fig S1 continued

Group	Sequence	Start	Alignment	End
Peptidase S6 Genotype 1 *Closest Gen 1 homolog to genotype 2 specific Peptidase S6*	CP017495 (1b) locus BG548_01775	703	--	702
	CP017510 (1c) locus BG556_01775	703	--	702
	CP017502 (1e) locus BG561_01775	703	--	702
	CP017484 (1f) locus BG572_01775	703	--	702
	CP017499 (1i) locus BG576_11055	703	--	702
Peptidase S6 Genotype 2 specific*	CP017538 (2b) locus BG586_02565	801	LQANLSANDHAQLHLGFINEQTPSCYYSEYTGKTSCDTQAVVSNEIFATLPTTQINGDVSLAEQSQLHIG-KANLIGTIQAAATTSIRLANQASWTNTGDSRTGNLVAENGSTINLNEKFVTGEI	924
	CP017491 (2c) locus BG598_01940	801	LQANLSANDHAQLHLGFINEQTPSCYYSEYTGKTSCDTQAVVSNEIFATLPTTQINGDVSLAEQSQLHIG-KANLIGTIQAAATTSIRLANQASWTNTGDSRTGNLVAENGSTINLNEKFVTGEI	924
	CP017505 (2d) locus BG605_01935	801	LQANLSANDHAQLHLGFINEQTPSCYYSEYTGKTSCDTQAVVSNEIFATLPTTQINGDVSLAEQSQLHIG-KANLIGTIQAAATTSIRLANQASWTNTGDSRTGNLVAENGSTINLNEKFVTGEI	924
	CP017552 (2e) locus BG607_01950	801	LQANLSANDHAQLHLGFINEQTPSCYYSEYTGKTSCDTQAVVSNEIFATLPTTQINGDVSLAEQSQLHIG-KANLIGTIQAAATTSIRLANQASWTNTGDSRTGNLVAENGSTINLNEKFVTGEI	924
Peptidase S6 Genotype 1	CP017495 (1b) locus BG548_05165	718	VNSHFTATDKAKLNLGFINRSTPSCYDSEYTGTTHCEVQAVISDNIFANLATTAIKGNVKLQNHSQLNLG-KANLTGSVQADQTTHITLANHSHWLNNGTSQIGHLTMEKGSILSLNDKFATTEI	841
	CP017510 (1c) locus BG556_05150	718	VNSHFTATDKAKLNLGFINRSTPSCYDSEYTGTTHCEVQAVISDNIFANLATTAIKGNVKLQNHSQLNLG-KANLTGSVQADQTTHITLANHSHWLNNGTSQIGHLTMEKGSILSLNDKFATTEI	841
	CP017502 (1e) locus BG561_05135	718	VNSHFTATDKAKLNLGFINRSTPSCYDSEYTGTTHCEVQAVISDNIFANLATTAIKGNVKLQNHSQLNLG-KANLTGSVQADQTTHITLANHSHWLNNGTSQIGHLTMEKGSILSLNDKFATTEI	841
	CP017484 (1f) locus BG572_05155	718	VNSHFTATDKAKLNLGFINRSTPSCYDSEYTGTTHCEVQAVISDNIFANLATTAIKGNVKLQNHSQLNLG-KANLTGSVQADQTTHITLANHSHWLNNGTSQIGHLTMEKGSILSLNDKFATTEI	841
	CP017499 (1i) locus BG576_07665	718	VNSHFTATDKAKLNLGFINRSTPSCYDSEYTGTTHCEVQAVISDNIFANLATTAIKGNVKLQNHSQLNLG-KANLTGSVQADQTTHITLANHSHWLNNGTSQIGHLTMEKGSILSLNDKFATTEI	841
Peptidase S6 Genotype 2	CP017538 (2b) locus BG586_14225	718	VNSHFTATDKAKLNLGFINRSTPSCYDSEYTGTTHCEVQAVISDNIFANLATTAIKGNVKLQNHSQLNLG-KANLTGSVQADQTTHITLANHSHWLNNGTSQIGHLTMEKGSILSLNDKFATTEI	841
	CP017491 (2c) locus BG598_04535	718	VNSHFTATDKAKLNLGFINRSTPSCYDSEYTGTTHCEVQAVISDNIFANLATTAIKGNVKLQNHSQLNLG-KANLTGSVQADQTTHITLANHSHWLNNGTSQIGHLTMEKGSILSLNDKFATTEI	841
	CP017505 (2d) locus BG605_04535	718	VNSHFTATDKAKLNLGFINRSTPSCYDSEYTGTTHCEVQAVISDNIFANLATTAIKGNVKLQNHSQLNLG-KANLTGSVQADQTTHITLANHSHWLNNGTSQIGHLTMEKGSILSLNDKFATTEI	841
	CP017552 (2e) locus BG607_04545	718	VNSHFTATDKAKLNLGFINRSTPSCYDSEYTGTTHCEVQAVISDNIFANLATTAIKGNVKLQNHSQLNLG-KANLTGSVQADQTTHITLANHSHWLNNGTSQIGHLTMEKGSILSLNDKFATTEI	841
Peptidase S6 Genotype 1	CP017495 (1b) locus BG548_02680	862	INGNFNLTDNATAQIGFTQGTSQACIRSDRTGVATCNINATLSETDLNSWERTKVAGNVSLADNSTFSLGSKADLTGSITAQESTKVQLNDGSIANLTGLSTTGIFNGANGSTVNLSGVWTTLTN	986
	CP017510 (1c) locus BG556_02675	862	INGNFNLTDNATAQIGFTQGTSQACIRSDRTGVATCNINATLSETDLNSWERTKVAGNVSLADNSTFSLGSKADLTGSITAQESTKVQLNDGSIANLTGLSTTGIFNGANGSTVNLSGVWTTLTN	986
	CP017502 (1e) locus BG561_02675	862	INGNFNLTDNATAQIGFTQGTSQACIRSDRTGVATCNINATLSETDLNSWERTKVAGNVSLADNSTFSLGSKADLTGSITAQESTKVQLNDGSIANLTGLSTTGIFNGANGSTVNLSGVWTTLTN	986
	CP017484 (1f) locus BG572_02680	862	INGNFNLTDNATAQIGFTQGTSQACIRSDRTGVATCNINATLSETDLNSWERTKVAGNVSLADNSTFSLGSKADLTGSITAQESTKVQLNDGSIANLTGLSTTGIFNGANGSTVNLSGVWTTLTN	986
	CP017499 (1i) locus BG576_10160	862	INGNFNLTDNATAQIGFTQGTSQACIRSDRTGVATCNINATLSETDLNSWERTKVAGNVSLADNSTFSLGSKADLTGSITAQESTKVQLNDGSIANLTGLSTTGIFNGANGSTVNLSGVWTTLTN	986
S6 family IgA-specific metalloendopeptidase Genotype 2	CP017538 (2b) locus BG586_01400	813	--	812
	CP017491 (2c) locus BG598_03110	813	--	812
	CP017505 (2d) locus BG605_03105	813	--	812
	CP017552 (2e) locus BG607_03115	813	--	812
Peptidase S6 Genotype 1	CP017495 (1b) locus BG548_10695	699	VNGNFNLSDNATAQVGFTQGKSQSCVR---	725
	CP017510 (1c) locus BG556_10675	699	VNGNFNLSDNATAQVGFTQGKSQSCVR---	725
	CP017502 (1e) locus BG561_10810	699	VNGNFNLSDNATAQVGFTQGKSQSCVR---	725
	CP017484 (1f) locus BG572_10720	699	VNGNFNLSDNATAQVGFTQGKSQSCVR---	725
	CP017499 (1i) locus BG576_02270	699	VNGNFNLSDNATAQVGFTQGKSQSCVR---	725
S6 family IgA-specific metalloendopeptidase Genotype 2	CP017538 (2b) locus BG586_01655	643	YTGDIEHSEKGSGETAEPLPKYNGNAHLIAS--	673
	CP017491 (2c) locus BG598_02860	643	YTGDIEHSEKGSGETAEPLPKYNGNAHLIAS--	673
	CP017505 (2d) locus BG605_02855	643	YTGDIEHSEKGSGETAEPLPKYNGNAHLIAS--	673
	CP017552 (2e) locus BG607_02860	643	YTGDIEHSEKGSGETAEPLPKYNGNAHLIAS--	673

Fig S1 continued

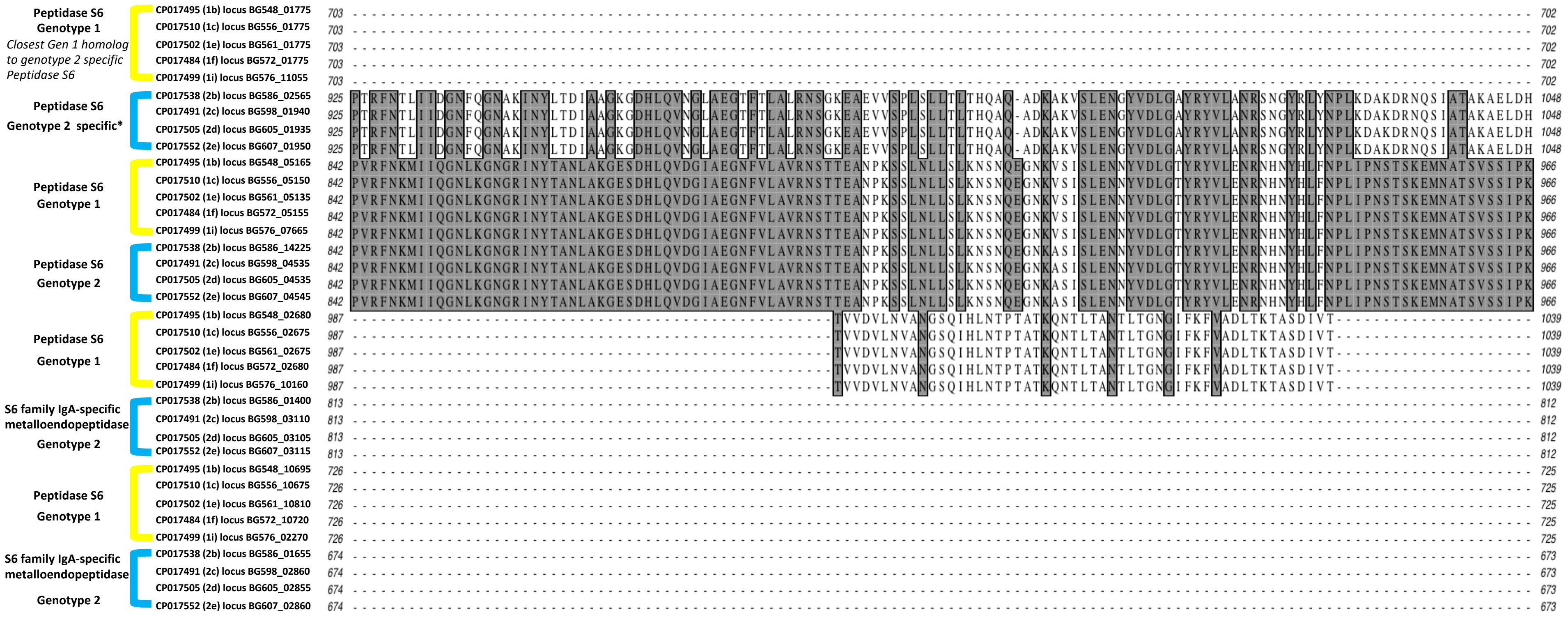

Fig S1 continued

Group	Sequence	Start	Alignment	End
Peptidase S6 Genotype 1 *Closest Gen 1 homolog to genotype 2 specific Peptidase S6*	CP017495 (1b) locus BG548_01775	703	---	702
	CP017510 (1c) locus BG556_01775	703	---	702
	CP017502 (1e) locus BG561_01775	703	---	702
	CP017484 (1f) locus BG572_01775	703	---	702
	CP017499 (1i) locus BG576_11055	703	---	702
Peptidase S6 Genotype 2 specific*	CP017538 (2b) locus BG586_02565	1049	-------AIAEADKQKQEISRLNAEAEKERQAEKNAKQAAANAQSQLSQANSELTRLQQYADYYRRYYPTYYRQIQGQITVAKQKVTQASAALTTAENNAKASAAQIANVEQAVETAQNIAKQVE	1166
	CP017491 (2c) locus BG598_01940	1049	-------AIAEADKQKQEISRLNAEAEKERQAEKNAKQAAANAQSQLSQANSELTRLQQYADYYRRYYPTYYRQIQGQITVAKQKVTQASAALTTAENNAKASAAQIANVEQAVETAQNIAKQVE	1166
	CP017505 (2d) locus BG605_01935	1049	-------AIAEADKQKQEISRLNAEAEKERQAEKNAKQAAANAQSQLSQANSELTRLQQYADYYRRYYPTYYRQIQGQITVAKQKVTQASAALTTAENNAKASAAQIANVEQAVETAQNIAKQVE	1166
	CP017552 (2e) locus BG607_01950	1049	-------AIAEADKQKQEISRLNAEAEKERQAEKNAKQAAANAQSQLSQANSELTRLQQYADYYRRYYPTYYRQIQGQITVAKQKVTQASAALTTAENNAKASAAQIANVEQAVETAQNIAKQVE	1166
Peptidase S6 Genotype 1	CP017495 (1b) locus BG548_05165	967	KESVTNVPTLDKKETEQNLTQLQKDFSAHQLENQKAKQSMINAQSELRRLNSQLNVLQKYVNSRRLGYYT-QQAVLEQISIIQNKIKQTQTIFNDANATVKLTDQKLEEAKLALGSVN-------	1083
	CP017510 (1c) locus BG556_05150	967	KESVTNVPTLDKKETEQNLTQLQKDFSAHQLENQKAKQSMINAQSELRRLNSQLNVLQKYVNSRRLGYYT-QQAVLEQISIIQNKIKQTQTIFNDANATVKLTDQKLEEAKLALGSVN-------	1083
	CP017502 (1e) locus BG561_05135	967	KESVTNVPTLDKKETEQNLTQLQKDFSAHQLENQKAKQSMINAQSELRRLNSQLNVLQKYVNSRRLGYYT-QQAVLEQISIIQNKIKQTQTIFNDANATVKLTDQKLEEAKLALGSVN-------	1083
	CP017484 (1f) locus BG572_05155	967	KESVTNVPTLDKKETEQNLTQLQKDFSAHQLENQKAKQSMINAQSELRRLNSQLNVLQKYVNSRRLGYYT-QQAVLEQISIIQNKIKQTQTIFNDANATVKLTDQKLEEAKLALGSVN-------	1083
	CP017499 (1i) locus BG576_07665	967	KESVTNVPTLDKKETEQNLTQLQKDFSAHQLENQKAKQSMINAQSELRRLNSQLNVLQKYVNSRRLGYYT-QQAVLEQISIIQNKIKQTQTIFNDANATVKLTDQKLEEAKLALGSVN-------	1083
Peptidase S6 Genotype 2	CP017538 (2b) locus BG586_14225	967	KESVTNVPTLDKKETEQNLTQLQKDFSAHQLENQKAKQSMINAQSELRRLNSQLNVLQKYVNSRRLGYYT-QQAVLEQISIIQNKIKQTQTIFNDANATVKLTDQKLEEAKLALGSVN-------	1083
	CP017491 (2c) locus BG598_04535	967	KESVTNVPTLDKKETEQNLTQLQKDFSAHQLENQKAKQSMINAQSELRRLNSQLNVLQKYVNSRRLGYYT-QQAVLEQISIIQNKIKQTQTIFNDANATVKLTDQKLEEAKLALGSVN-------	1083
	CP017505 (2d) locus BG605_04535	967	KESVTNVPTLDKKETEQNLTQLQKDFSAHQLENQKAKQSMINAQSELRRLNSQLNVLQKYVNSRRLGYYT-QQAVLEQISIIQNKIKQTQTIFNDANATVKLTDQKLEEAKLALGSVN-------	1083
	CP017552 (2e) locus BG607_04545	967	KESVTNVPTLDKKETEQNLTQLQKDFSAHQLENQKAKQSMINAQSELRRLNSQLNVLQKYVNSRRLGYYT-QQAVLEQISIIQNKIKQTQTIFNDANATVKLTDQKLEEAKLALGSVN-------	1083
Peptidase S6 Genotype 1	CP017495 (1b) locus BG548_02680	1040	---------IKDTASGQFSIEISTNGSAHSAQKIKLFETTNEEDFSLNLANEVRSGEYRYHLLKEGNVYYLNPEKEEEPKITQKGPSETADPLPEYTGDIEYSEKGPGETADPL-----------	1144
	CP017510 (1c) locus BG556_02675	1040	---------IKDTASGQFSIEISTNGSAHSAQKIKLFETTNEEDFSLNLANEVRSGEYRYHLLKEGNVYYLNPEKEEEPKITQKGPSETADPLPEYTGDIEYSEKGPGETADPL-----------	1144
	CP017502 (1e) locus BG561_02675	1040	---------IKDTASGQFSIEISTNGSAHSAQKIKLFETTNEEDFSLNLANEVRSGEYRYHLLKEGNVYYLNPEKEEEPKITQKGPSETADPLPEYTGDIEYSEKGPGETADPL-----------	1144
	CP017484 (1f) locus BG572_02680	1040	---------IKDTASGQFSIEISTNGSAHSAQKIKLFETTNEEDFSLNLANEVRSGEYRYHLLKEGNVYYLNPEKEEEPKITQKGPSETADPLPEYTGDIEYSEKGPGETADPL-----------	1144
	CP017499 (1i) locus BG576_10160	1040	---------IKDTASGQFSIEISTNGSAHSAQKIKLFETTNEEDFSLNLANEVRSGEYRYHLLKEGNVYYLNPEKEEEPKITQKGPSETADPLPEYTGDIEYSEKGPGETADPL-----------	1144
S6 family IgA-specific metalloendopeptidase Genotype 2	CP017538 (2b) locus BG586_01400	813	---	812
	CP017491 (2c) locus BG598_03110	813	---	812
	CP017505 (2d) locus BG605_03105	813	---	812
	CP017552 (2e) locus BG607_03115	813	---	812
Peptidase S6 Genotype 1	CP017495 (1b) locus BG548_10695	726	---	725
	CP017510 (1c) locus BG556_10675	726	---	725
	CP017502 (1e) locus BG561_10810	726	---	725
	CP017484 (1f) locus BG572_10720	726	---	725
	CP017499 (1i) locus BG576_02270	726	---	725
S6 family IgA-specific metalloendopeptidase Genotype 2	CP017538 (2b) locus BG586_01655	674	---	673
	CP017491 (2c) locus BG598_02860	674	---	673
	CP017505 (2d) locus BG605_02855	674	---	673
	CP017552 (2e) locus BG607_02860	674	---	673

Fig S1 continued

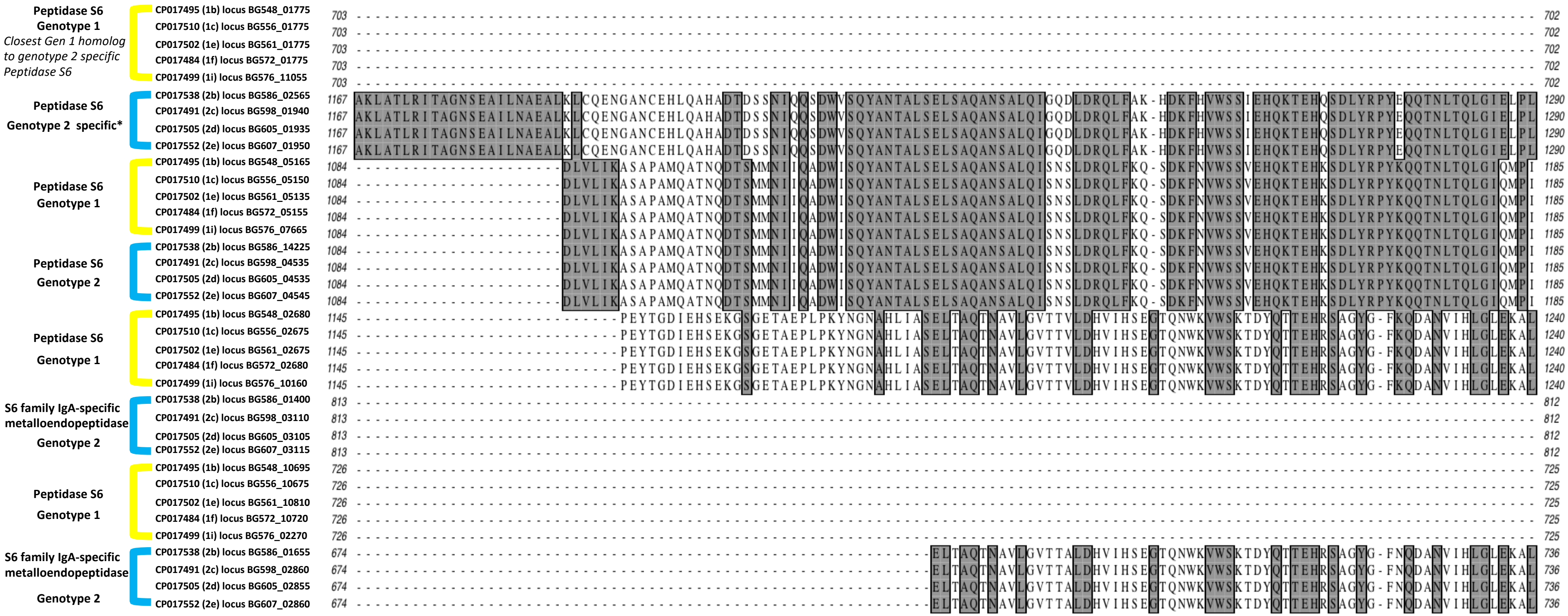

Fig S1 continued

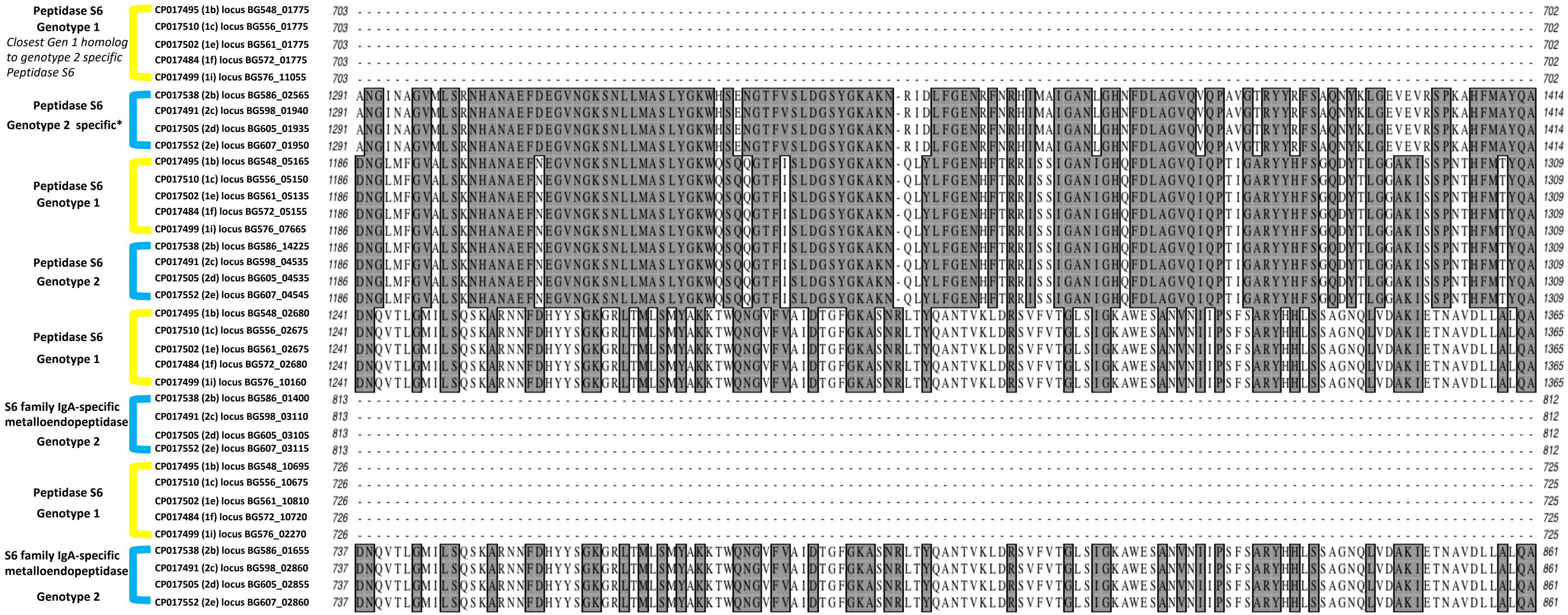

Fig S1 continued

Group	Sequence	Start	Alignment	End
Peptidase S6 Genotype 1 *Closest Gen 1 homolog to genotype 2 specific Peptidase S6*	CP017495 (1b) locus BG548_01775	703		702
	CP017510 (1c) locus BG556_01775	703		702
	CP017502 (1e) locus BG561_01775	703		702
	CP017484 (1f) locus BG572_01775	703		702
	CP017499 (1i) locus BG576_11055	703		702
Peptidase S6 Genotype 2 specific*	CP017538 (2b) locus BG586_02565	1415	GVKVSKAFDLSSWKVEPSLAAHYVDASSKRLSVAVNDNTFSQRFGRYLKTEVGVGVTKGQWQLSSHLGLLKGNEIGKQHFAGFKLGYSW	1503
	CP017491 (2c) locus BG598_01940	1415	GVKVSKAFDLSSWKVEPSLAAHYVDASSKRLSVAVNDNTFSQRFGRYLKTEVGVGVTKGQWQLSSHLGLLKGNEIGKQHFAGFKLGYSW	1503
	CP017505 (2d) locus BG605_01935	1415	GVKVSKAFDLSSWKVEPSLAAHYVDASSKRLSVAVNDNTFSQRFGRYLKTEVGVGVTKGQWQLSSHLGLLKGNEIGKQHFAGFKLGYSW	1503
	CP017552 (2e) locus BG607_01950	1415	GVKVSKAFDLSSWKVEPSLAAHYVDASSKRLSVAVNDNTFSQRFGRYLKTEVGVGVTKGQWQLSSHLGLLKGNEIGKQHFAGFKLGYSW	1503
Peptidase S6 Genotype 1	CP017495 (1b) locus BG548_05165	1310	GLKASKTFSLDDWKVEPSITTHYVDASNKRLAVNVNNNNINQRFGRYLKTEAGIRFEHNQWQFDLHAGFINGNEIKKQHFAGVKLGYSW	1398
	CP017510 (1c) locus BG556_05150	1310	GLKASKTFSLDDWKVEPSITTHYVDASNKRLAVNVNNNNINQRFGRYLKTEAGIRFEHNQWQFDLHAGFINGNEIKKQHFAGVKLGYSW	1398
	CP017502 (1e) locus BG561_05135	1310	GLKASKTFSLDDWKVEPSITTHYVDASNKRLAVNVNNNNINQRFGRYLKTEAGIRFEHNQWQFDLHAGFINGNEIKKQHFAGVKLGYSW	1398
	CP017484 (1f) locus BG572_05155	1310	GLKASKTFSLDDWKVEPSITTHYVDASNKRLAVNVNNNNINQRFGRYLKTEAGIRFEHNQWQFDLHAGFINGNEIKKQHFAGVKLGYSW	1398
	CP017499 (1i) locus BG576_07665	1310	GLKASKTFSLDDWKVEPSITTHYVDASNKRLAVNVNNNNINQRFGRYLKTEAGIRFEHNQWQFDLHAGFINGNEIKKQHFAGVKLGYSW	1398
Peptidase S6 Genotype 2	CP017538 (2b) locus BG586_14225	1310	GLKASKTFSLDDWKVEPSITTHYVDASNKRLAVNVNNNNINQRFGRYLKTEAGIRFEHNQWQFDLHAGFINGNEIKKQHFAGVKLGYSW	1398
	CP017491 (2c) locus BG598_04535	1310	GLKASKTFSLDDWKVEPSITTHYVDASNKRLAVNVNNNNINQRFGRYLKTEAGIRFEHNQWQFDLHAGFINGNEIKKQHFAGVKLGYSW	1398
	CP017505 (2d) locus BG605_04535	1310	GLKASKTFSLDDWKVEPSITTHYVDASNKRLAVNVNNNNINQRFGRYLKTEAGIRFEHNQWQFDLHAGFINGNEIKKQHFAGVKLGYSW	1398
	CP017552 (2e) locus BG607_04545	1310	GLKASKTFSLDDWKVEPSITTHYVDASNKRLAVNVNNNNINQRFGRYLKTEAGIRFEHNQWQFDLHAGFINGNEIKKQHFAGVKLGYSW	1398
Peptidase S6 Genotype 1	CP017495 (1b) locus BG548_02680	1366	GLSINKTLELNGLQIKPEIGSYFVDASHGKLRTRFNNLQIEQQMGRYFKQEAGISANYRNINAGIQAGFLTGNTLNKQRYISFKVTYEW	1454
	CP017510 (1c) locus BG556_02675	1366	GLSINKTLELNGLQIKPEIGSYFVDASHGKLRTRFNNLQIEQQMGRYFKQEAGISANYRNINAGIQAGFLTGNTLNKQRYISFKVTYEW	1454
	CP017502 (1e) locus BG561_02675	1366	GLSINKTLELNGLQIKPEIGSYFVDASHGKLRTRFNNLQIEQQMGRYFKQEAGISANYRNINAGIQAGFLTGNTLNKQRYISFKVTYEW	1454
	CP017484 (1f) locus BG572_02680	1366	GLSINKTLELNGLQIKPEIGSYFVDASHGKLRTRFNNLQIEQQMGRYFKQEAGISANYRNINAGIQAGFLTGNTLNKQRYISFKVTYEW	1454
	CP017499 (1i) locus BG576_10160	1366	GLSINKTLELNGLQIKPEIGSYFVDASHGKLRTRFNNLQIEQQMGRYFKQEAGISANYRNINAGIQAGFLTGNTLNKQRYISFKVTYEW	1454
S6 family IgA-specific metalloendopeptidase Genotype 2	CP017538 (2b) locus BG586_01400	813		812
	CP017491 (2c) locus BG598_03110	813		812
	CP017505 (2d) locus BG605_03105	813		812
	CP017552 (2e) locus BG607_03115	813		812
Peptidase S6 Genotype 1	CP017495 (1b) locus BG548_10695	726		725
	CP017510 (1c) locus BG556_10675	726		725
	CP017502 (1e) locus BG561_10810	726		725
	CP017484 (1f) locus BG572_10720	726		725
	CP017499 (1i) locus BG576_02270	726		725
S6 family IgA-specific metalloendopeptidase Genotype 2	CP017538 (2b) locus BG586_01655	862	GLSINKTLELNGLQIKPEIGSYFVDASHGKLRTRFNNLQIEQQMGRYFKQEAGISANYRNINAGIQAGFLTGNTLNKQRYISFKVTYEW	950
	CP017491 (2c) locus BG598_02860	862	GLSINKTLELNGLQIKPEIGSYFVDASHGKLRTRFNNLQIEQQMGRYFKQEAGISANYRNINAGIQAGFLTGNTLNKQRYISFKVTYEW	950
	CP017505 (2d) locus BG605_02855	862	GLSINKTLELNGLQIKPEIGSYFVDASHGKLRTRFNNLQIEQQMGRYFKQEAGISANYRNINAGIQAGFLTGNTLNKQRYISFKVTYEW	950
	CP017552 (2e) locus BG607_02860	862	GLSINKTLELNGLQIKPEIGSYFVDASHGKLRTRFNNLQIEQQMGRYFKQEAGISANYRNINAGIQAGFLTGNTLNKQRYISFKVTYEW	950
