# Supplementary material for: Differences between predicted outer membrane proteins of genotype 1 and 2 Mannheimia haemolytica
Source: BMC Microbiol. 2020 Aug 12;20:250. doi: 10.1186/s12866-020-01932-2 (PMC7424683; doi:10.1186/s12866-020-01932-2)
Supplement: Supplementary file 7 — Additional file 7: Figure S2. Alignment of all detected isoforms of the peptidase S6 gene specific to genotype 2 M. haemolytica and those encoded by a homologous pseudogene in genotype 1 M. haemolytica. The alignment shows all five isoforms of the peptidase S6 specific to genotype 2 M. haemolytica that were detected, and all four of the peptidase S6 homologs in genotype 1 M. haemolytica which all contained a premature stop codon. Isoform numbers in this figure correspond to the isoform numbers listed in Additional file 1: Table S1. *184 iso 1 is an abbreviation for USDA-ARS-USMARC-184 isoform 1. Areas of 51% chemical identity or greater are indicated with grey boxes. [file 12866_2020_1932_MOESM7_ESM.pdf]

Fig S2

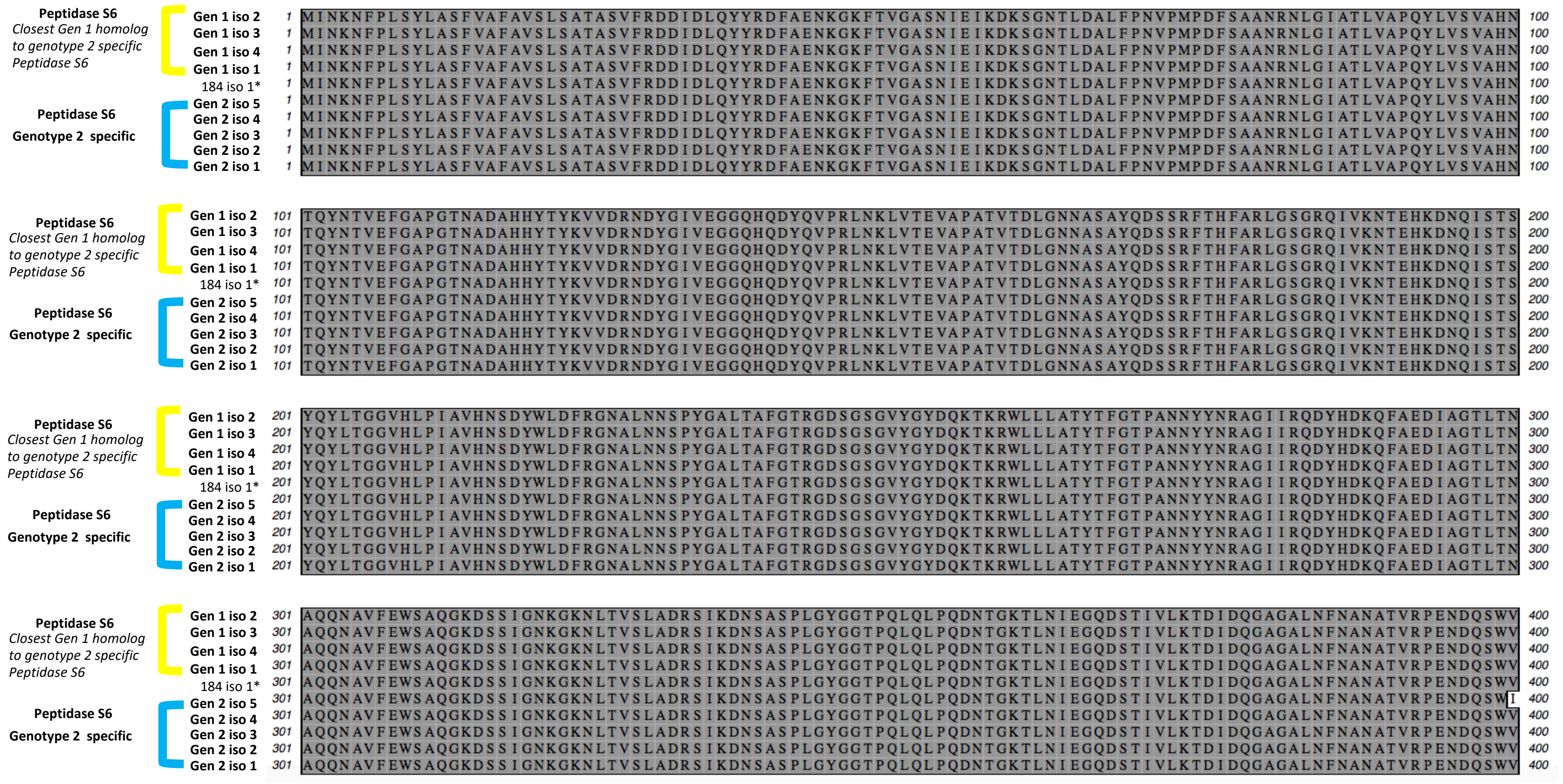

Peptidase S6
Closest Gen 1 homolog to genotype 2 specific Peptidase S6
Peptidase S6
Genotype 2 specific
Gen 1 iso 2 1 MINKNFPLSYLASFVAFAVSLSATASVFRDDIDLQYYRDFAENKGKFTVGASNIEIKDKSGNTLDALFPNVPMPDFSAANRNLGIATLVAPQYLVSVAHN 100
Gen 1 iso 3 1 MINKNFPLSYLASFVAFAVSLSATASVFRDDIDLQYYRDFAENKGKFTVGASNIEIKDKSGNTLDALFPNVPMPDFSAANRNLGIATLVAPQYLVSVAHN 100
Gen 1 iso 4 1 MINKNFPLSYLASFVAFAVSLSATASVFRDDIDLQYYRDFAENKGKFTVGASNIEIKDKSGNTLDALFPNVPMPDFSAANRNLGIATLVAPQYLVSVAHN 100
Gen 1 iso 1 1 MINKNFPLSYLASFVAFAVSLSATASVFRDDIDLQYYRDFAENKGKFTVGASNIEIKDKSGNTLDALFPNVPMPDFSAANRNLGIATLVAPQYLVSVAHN 100
184 iso 1* 1 MINKNFPLSYLASFVAFAVSLSATASVFRDDIDLQYYRDFAENKGKFTVGASNIEIKDKSGNTLDALFPNVPMPDFSAANRNLGIATLVAPQYLVSVAHN 100
Gen 2 iso 5 1 MINKNFPLSYLASFVAFAVSLSATASVFRDDIDLQYYRDFAENKGKFTVGASNIEIKDKSGNTLDALFPNVPMPDFSAANRNLGIATLVAPQYLVSVAHN 100
Gen 2 iso 4 1 MINKNFPLSYLASFVAFAVSLSATASVFRDDIDLQYYRDFAENKGKFTVGASNIEIKDKSGNTLDALFPNVPMPDFSAANRNLGIATLVAPQYLVSVAHN 100
Gen 2 iso 3 1 MINKNFPLSYLASFVAFAVSLSATASVFRDDIDLQYYRDFAENKGKFTVGASNIEIKDKSGNTLDALFPNVPMPDFSAANRNLGIATLVAPQYLVSVAHN 100
Gen 2 iso 2 1 MINKNFPLSYLASFVAFAVSLSATASVFRDDIDLQYYRDFAENKGKFTVGASNIEIKDKSGNTLDALFPNVPMPDFSAANRNLGIATLVAPQYLVSVAHN 100
Gen 2 iso 1 1 MINKNFPLSYLASFVAFAVSLSATASVFRDDIDLQYYRDFAENKGKFTVGASNIEIKDKSGNTLDALFPNVPMPDFSAANRNLGIATLVAPQYLVSVAHN 100
Peptidase S6
Closest Gen 1 homolog to genotype 2 specific Peptidase S6
Peptidase S6
Genotype 2 specific
Gen 1 iso 2 101 TQYNTVEFGAPGTNADAHHYTYKVVDRNDYGIVEGGQHQDYQVPRLNKLVTEVAPATVTDLGNNASAYQDSSRFTHFARLGSGRQIVKNTEHKDNQISTS 200
Gen 1 iso 3 101 TQYNTVEFGAPGTNADAHHYTYKVVDRNDYGIVEGGQHQDYQVPRLNKLVTEVAPATVTDLGNNASAYQDSSRFTHFARLGSGRQIVKNTEHKDNQISTS 200
Gen 1 iso 4 101 TQYNTVEFGAPGTNADAHHYTYKVVDRNDYGIVEGGQHQDYQVPRLNKLVTEVAPATVTDLGNNASAYQDSSRFTHFARLGSGRQIVKNTEHKDNQISTS 200
Gen 1 iso 1 101 TQYNTVEFGAPGTNADAHHYTYKVVDRNDYGIVEGGQHQDYQVPRLNKLVTEVAPATVTDLGNNASAYQDSSRFTHFARLGSGRQIVKNTEHKDNQISTS 200
184 iso 1* 101 TQYNTVEFGAPGTNADAHHYTYKVVDRNDYGIVEGGQHQDYQVPRLNKLVTEVAPATVTDLGNNASAYQDSSRFTHFARLGSGRQIVKNTEHKDNQISTS 200
Gen 2 iso 5 101 TQYNTVEFGAPGTNADAHHYTYKVVDRNDYGIVEGGQHQDYQVPRLNKLVTEVAPATVTDLGNNASAYQDSSRFTHFARLGSGRQIVKNTEHKDNQISTS 200
Gen 2 iso 4 101 TQYNTVEFGAPGTNADAHHYTYKVVDRNDYGIVEGGQHQDYQVPRLNKLVTEVAPATVTDLGNNASAYQDSSRFTHFARLGSGRQIVKNTEHKDNQISTS 200
Gen 2 iso 3 101 TQYNTVEFGAPGTNADAHHYTYKVVDRNDYGIVEGGQHQDYQVPRLNKLVTEVAPATVTDLGNNASAYQDSSRFTHFARLGSGRQIVKNTEHKDNQISTS 200
Gen 2 iso 2 101 TQYNTVEFGAPGTNADAHHYTYKVVDRNDYGIVEGGQHQDYQVPRLNKLVTEVAPATVTDLGNNASAYQDSSRFTHFARLGSGRQIVKNTEHKDNQISTS 200
Gen 2 iso 1 101 TQYNTVEFGAPGTNADAHHYTYKVVDRNDYGIVEGGQHQDYQVPRLNKLVTEVAPATVTDLGNNASAYQDSSRFTHFARLGSGRQIVKNTEHKDNQISTS 200
Peptidase S6
Closest Gen 1 homolog to genotype 2 specific Peptidase S6
Peptidase S6
Genotype 2 specific
Gen 1 iso 2 201 YQYLTGGVHLPIAVHNSDYWLDFRGNALNNSPYGALTAFGTRGDSGSGVYGYDQKTKRWLLLATYTFGTPANNYYNRAGIIRQDYHDKQFAEDIAGTLTN 300
Gen 1 iso 3 201 YQYLTGGVHLPIAVHNSDYWLDFRGNALNNSPYGALTAFGTRGDSGSGVYGYDQKTKRWLLLATYTFGTPANNYYNRAGIIRQDYHDKQFAEDIAGTLTN 300
Gen 1 iso 4 201 YQYLTGGVHLPIAVHNSDYWLDFRGNALNNSPYGALTAFGTRGDSGSGVYGYDQKTKRWLLLATYTFGTPANNYYNRAGIIRQDYHDKQFAEDIAGTLTN 300
Gen 1 iso 1 201 YQYLTGGVHLPIAVHNSDYWLDFRGNALNNSPYGALTAFGTRGDSGSGVYGYDQKTKRWLLLATYTFGTPANNYYNRAGIIRQDYHDKQFAEDIAGTLTN 300
184 iso 1* 201 YQYLTGGVHLPIAVHNSDYWLDFRGNALNNSPYGALTAFGTRGDSGSGVYGYDQKTKRWLLLATYTFGTPANNYYNRAGIIRQDYHDKQFAEDIAGTLTN 300
Gen 2 iso 5 201 YQYLTGGVHLPIAVHNSDYWLDFRGNALNNSPYGALTAFGTRGDSGSGVYGYDQKTKRWLLLATYTFGTPANNYYNRAGIIRQDYHDKQFAEDIAGTLTN 300
Gen 2 iso 4 201 YQYLTGGVHLPIAVHNSDYWLDFRGNALNNSPYGALTAFGTRGDSGSGVYGYDQKTKRWLLLATYTFGTPANNYYNRAGIIRQDYHDKQFAEDIAGTLTN 300
Gen 2 iso 3 201 YQYLTGGVHLPIAVHNSDYWLDFRGNALNNSPYGALTAFGTRGDSGSGVYGYDQKTKRWLLLATYTFGTPANNYYNRAGIIRQDYHDKQFAEDIAGTLTN 300
Gen 2 iso 2 201 YQYLTGGVHLPIAVHNSDYWLDFRGNALNNSPYGALTAFGTRGDSGSGVYGYDQKTKRWLLLATYTFGTPANNYYNRAGIIRQDYHDKQFAEDIAGTLTN 300
Gen 2 iso 1 201 YQYLTGGVHLPIAVHNSDYWLDFRGNALNNSPYGALTAFGTRGDSGSGVYGYDQKTKRWLLLATYTFGTPANNYYNRAGIIRQDYHDKQFAEDIAGTLTN 300
Peptidase S6
Closest Gen 1 homolog to genotype 2 specific Peptidase S6
Peptidase S6
Genotype 2 specific
Gen 1 iso 2 301 AQQNAVFEWSAQGKDSSIGNKGKNLTVSLADRSIKDNSASPLGYGGTPQLQLPQDNTGKTLNIEGQDSTIVLKTDIDQGAGALNFNANATVRPENDQSWV 400
Gen 1 iso 3 301 AQQNAVFEWSAQGKDSSIGNKGKNLTVSLADRSIKDNSASPLGYGGTPQLQLPQDNTGKTLNIEGQDSTIVLKTDIDQGAGALNFNANATVRPENDQSWV 400
Gen 1 iso 4 301 AQQNAVFEWSAQGKDSSIGNKGKNLTVSLADRSIKDNSASPLGYGGTPQLQLPQDNTGKTLNIEGQDSTIVLKTDIDQGAGALNFNANATVRPENDQSWV 400
Gen 1 iso 1 301 AQQNAVFEWSAQGKDSSIGNKGKNLTVSLADRSIKDNSASPLGYGGTPQLQLPQDNTGKTLNIEGQDSTIVLKTDIDQGAGALNFNANATVRPENDQSWV 400
184 iso 1* 301 AQQNAVFEWSAQGKDSSIGNKGKNLTVSLADRSIKDNSASPLGYGGTPQLQLPQDNTGKTLNIEGQDSTIVLKTDIDQGAGALNFNANATVRPENDQSWV 400
Gen 2 iso 5 301 AQQNAVFEWSAQGKDSSIGNKGKNLTVSLADRSIKDNSASPLGYGGTPQLQLPQDNTGKTLNIEGQDSTIVLKTDIDQGAGALNFNANATVRPENDQSWI 400
Gen 2 iso 4 301 AQQNAVFEWSAQGKDSSIGNKGKNLTVSLADRSIKDNSASPLGYGGTPQLQLPQDNTGKTLNIEGQDSTIVLKTDIDQGAGALNFNANATVRPENDQSWV 400
Gen 2 iso 3 301 AQQNAVFEWSAQGKDSSIGNKGKNLTVSLADRSIKDNSASPLGYGGTPQLQLPQDNTGKTLNIEGQDSTIVLKTDIDQGAGALNFNANATVRPENDQSWV 400
Gen 2 iso 2 301 AQQNAVFEWSAQGKDSSIGNKGKNLTVSLADRSIKDNSASPLGYGGTPQLQLPQDNTGKTLNIEGQDSTIVLKTDIDQGAGALNFNANATVRPENDQSWV 400
Gen 2 iso 1 301 AQQNAVFEWSAQGKDSSIGNKGKNLTVSLADRSIKDNSASPLGYGGTPQLQLPQDNTGKTLNIEGQDSTIVLKTDIDQGAGALNFNANATVRPENDQSWV 400

Fig S2 continued

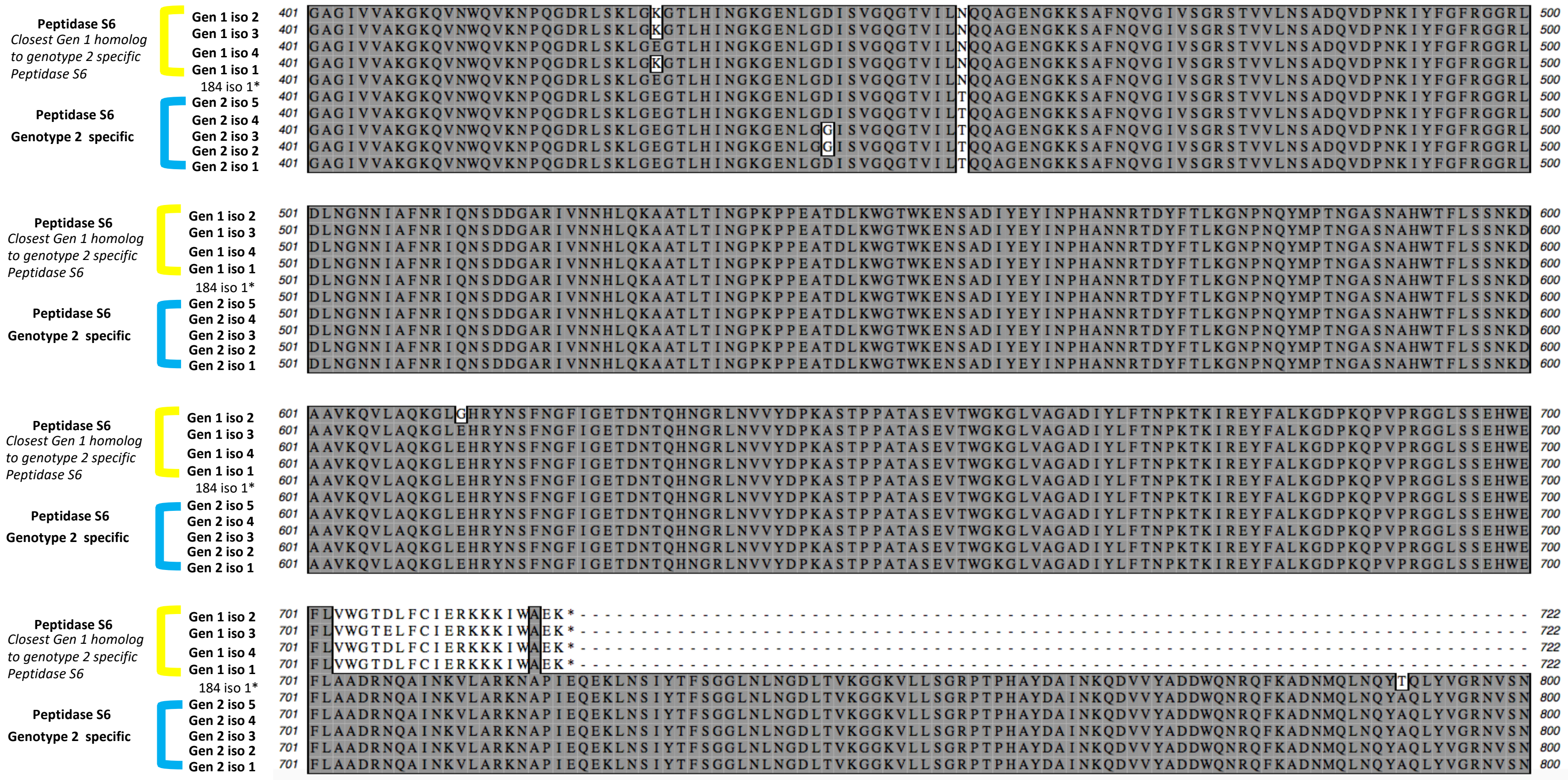

Fig S2 continued

Peptidase S6
Closest Gen 1 homolog to genotype 2 specific Peptidase S6

Peptidase S6
Genotype 2 specific

```
Gen 1 iso 2   723  ----------------------------------------------------------------------------------------------------  722
Gen 1 iso 3   723  ----------------------------------------------------------------------------------------------------  722
Gen 1 iso 4   723  ----------------------------------------------------------------------------------------------------  722
Gen 1 iso 1   723  ----------------------------------------------------------------------------------------------------  722
184 iso 1*    801  LQANLSANDHAQLHLGFINEQTPSCYYSEYTGKTSCDTQAVVSNEIFATLPTTQINGDVSLAEQSQLHIGKANLIGTIQAAATTSIRLANQASWTNTGDS  900
Gen 2 iso 5   801  LQANLSANDHAQLHLGFINEQTPSCYYSEYTGKTSCDTQAVVSNEIFATLPTTQINGDVSLAEQSQLHIGKANLIGTIQAAATTSIRLANQASWTNTGDS  900
Gen 2 iso 4   801  LQANLSANDHAQLHLGFINEQTPSCYYSEYTGKTSCDTQAVVSNEIFATLPTTQINGDVSLAEQSQLHIGKANLIGTIQAAATTSIRLANQASWTNTGDS  900
Gen 2 iso 3   801  LQANLSANGHAQLHLGFINEQTPSCYYSEYTGKTSCDTQAVVSNEIFATLPTTQINGDVSLAEQSQLHIGKANLIGTIQAAATTSIRLANQASWTNTGDS  900
Gen 2 iso 2   801  LQANLSANDHAQLHLGFINEQTPSCYYSEYTGKTSCDTQAVVSNEIFATLPTTQINGDVSLAEQSQLHIGKANLIGTIQAAATTSIRLANQASWTNTGDS  900
Gen 2 iso 1   801  LQANLSANDHAQLHLGFINEQTPSCYYSEYTGKTSCDTQAVVSNEIFATLPTTQINGDVSLAEQSQLHIGKANLIGTIQAAATTSIRLANQASWTNTGDS  900
```

Peptidase S6
Closest Gen 1 homolog to genotype 2 specific Peptidase S6

Peptidase S6
Genotype 2 specific

```
Gen 1 iso 2   723  ----------------------------------------------------------------------------------------------------  722
Gen 1 iso 3   723  ----------------------------------------------------------------------------------------------------  722
Gen 1 iso 4   723  ----------------------------------------------------------------------------------------------------  722
Gen 1 iso 1   723  ----------------------------------------------------------------------------------------------------  722
184 iso 1*    901  RTGNLVAENGSTINLNEKFVTGEIPTRFNTLIIDGNFQGNAKINYLTDIAAGKGDHLQVNGLAEGTFTLALRNSGKEAEVVSPLSLLTLTHQAQADKAKV  1000
Gen 2 iso 5   901  RTGNLVAENGSTINLNEKFVTGEIPTRFNTLIIDGNFQGNAKINYLTDIAAGKGDHLQVNGLAEGTFTLALRNSGKEAEVVSPLSLLTLTHQAQADKAKV  1000
Gen 2 iso 4   901  RTGNLVAENGSTINLNEKFVTGEIPTRFNTLIIDGNFQGNAKINYLTDIAAGKGDHL*VNGLAEGTFTLALRNSGKEAEVVSPLSLLTLTHQAQADKAKV  1000
Gen 2 iso 3   901  RTGNLVAENGSTINLNEKFVTGEIPTRFNTLIIDGNFQGNAKINYLTDIAAGKGDHLQVNGLAEGTFTLALRNSGKEAEVVSPLSLLTLTHQAQADKAKV  1000
Gen 2 iso 2   901  RTGNLVAENGSTINLNEKFVTGEIPTRFNTLIIDGNFQGNAKINYLTDIAAGKGDHLQVNGLAEGTFTLALRNSGKEAEVVSPLSLLTLTHQAQADKAKV  1000
Gen 2 iso 1   901  RTGNLVAENGSTINLNEKFVTGEIPTRFNTLIIDGNFQGNAKINYLTDIAAGKGDHLQVNGLAEGTFTLALRNSGKEAEVVSPLSLLTLTHQAQADKAKV  1000
```

Peptidase S6
Closest Gen 1 homolog to genotype 2 specific Peptidase S6

Peptidase S6
Genotype 2 specific

```
Gen 1 iso 2   723  ----------------------------------------------------------------------------------------------------  722
Gen 1 iso 3   723  ----------------------------------------------------------------------------------------------------  722
Gen 1 iso 4   723  ----------------------------------------------------------------------------------------------------  722
Gen 1 iso 1   723  ----------------------------------------------------------------------------------------------------  722
184 iso 1*    1001 SLENGYVDLGAYRYVLANRSNDYRLYNPLKDAKDRNQSIATAKAELDHAIAEADKQKQEISRLNAEAEKERQAEKNAKQATANAQSQLSQANSELTRLQQ  1100
Gen 2 iso 5   1001 SLENGYVDLGAYRYVLANRSNGYRLYNPLKDAKDRNQSIATAKAELDHAIAEADKQKQEISRLNAEAEKERQAEKNAKQAAANAQSQLSQANSELTRLQQ  1100
Gen 2 iso 4   1001 SLENGYVDLGAYRYVLANRSNGYRLYNPLKDAKDRNQSIATAKAELDHAIAEADKQKQEISRLNAEAEKERQAEKNAKQAAANAQSQLSQANSELTRLQQ  1100
Gen 2 iso 3   1001 SLENGYVDLGAYRYVLANRSNGYRLYNPLKDAKDRNQSIATAKAELDHAIAEADKQKQEISRLNAEAEKERQAEKNAKQAAANAQSQLSQANSELTRLQQ  1100
Gen 2 iso 2   1001 SLENGYVDLGAYRYVLANRSNGYRLYNPLKDAKDRNQSIATAKAELDHAIAEADKQKQEISRLNAEAEKERQAEKNAKQAAANAQSQLSQANSELTRLQQ  1100
Gen 2 iso 1   1001 SLENGYVDLGAYRYVLANRSNGYRLYNPLKDAKDRNQSIATAKAELDHAIAEADKQKQEISRLNAEAEKERQAEKNAKQAAANAQSQLSQANSELTRLQQ  1100
```

Peptidase S6
Closest Gen 1 homolog to genotype 2 specific Peptidase S6

Peptidase S6
Genotype 2 specific

```
Gen 1 iso 2   723  ----------------------------------------------------------------------------------------------------  722
Gen 1 iso 3   723  ----------------------------------------------------------------------------------------------------  722
Gen 1 iso 4   723  ----------------------------------------------------------------------------------------------------  722
Gen 1 iso 1   723  ----------------------------------------------------------------------------------------------------  722
184 iso 1*    1101 YADYYRRYYPTYYRQIQGQITVAKQKVTQASAELTTAENNAKASAAQIANVEQAVETAQNIAKQVEAKLATLRITAGNSEAILNAEALKLCQENGANCEH  1200
Gen 2 iso 5   1101 YADYYRRYYPTYYRQIQGQITVAKQKVTQASAALTTAENNAKASAAQIANVEQAVETAQNIAKQVEAKLATLRITAGNSEAILNAEALKLCQENGANCEH  1200
Gen 2 iso 4   1101 YADYYRRYYPTYYRQIQGQITVAKQKVTQASAALTTAENNAKASAAQIANVEQAVETAQNIAKQVEAKLATLRITAGNSEAILNAEALKLCQENGANCEH  1200
Gen 2 iso 3   1101 YADYYRRYYPTYYRQIQGQITVAKQKVTQASAALTTAENNAKASAAQIANVEQAVETAQNIAKQVEAKLATLRITAGNSEAILNAEALKLCQENGANCEH  1200
Gen 2 iso 2   1101 YADYYRRYYPTYYRQIQGQITVAKQKVTQASAALTTAENNAKASAAQIANVEQAVETAQNIAKQVEAKLATLRITAGNSEAILNAEALKLCQENGANCEH  1200
Gen 2 iso 1   1101 YADYYRRYYPTYYRQIQGQITVAKQKVTQASAALTTAENNAKASAAQIANVEQAVETAQNIAKQVEAKLATLRITAGNSEAILNAEALKLCQENGANCEH  1200
```

Fig S2 continued

Group	Sequence	Start	Alignment	End
**Peptidase S6** *Closest Gen 1 homolog to genotype 2 specific Peptidase S6*	**Gen 1 iso 2**	723	--	722
	**Gen 1 iso 3**	723	--	722
	**Gen 1 iso 4**	723	--	722
	**Gen 1 iso 1**	723	--	722
	184 iso 1*	1201	LQAHADTDSSNIQQSDWVSQYANTALSELSAQANSALQIGQGLDRQLFAKHDKFHVWSSIEHQKTEHQSDLYRPYEQQTNLTQLGIELPLANGINAGVVL	1300
**Peptidase S6 Genotype 2 specific**	**Gen 2 iso 5**	1201	LQAHADTDSSNIQQSDWVSQYANTALSELSAQANSALQIGQDLDRQLFAKHDKFHVWSSIEHQKTEHQSDLYRPYEQQTNLTQLGIELPLANGINAGVML	1300
	**Gen 2 iso 4**	1201	LQAHADTDSSNIQQSDWVSQYANTALSELSAQANSALQIGQDLDRQLFAKHDKFHVWSSIEHQKTEHQSDLYRPYEQQTNLTQLGIELPLANGINAGVML	1300
	**Gen 2 iso 3**	1201	LQAHADTDSSNIQQSDWVSQYANTALSELSAQANSALQIGQDLDRQLFAKHDKFHVWSSIEHQKTEHQSDLYRPYEQQTNLTQLGIELPLANGINAGVML	1300
	**Gen 2 iso 2**	1201	LQAHADTDSSNIQQSDWVSQYANTALSELSAQANSALQIGQDLDRQLFAKHDKFHVWSSIEHQKTEHQSDLYRPYEQQTNLTQLGIELPLANGINAGVML	1300
	**Gen 2 iso 1**	1201	LQAHADTDSSNIQQSDWVSQYANTALSELSAQANSALQIGQDLDRQLFAKHDKFHVWSSIEHQKTEHQSDLYRPYEQQTNLTQLGIELPLANGINAGVML	1300

Group	Sequence	Start	Alignment	End
**Peptidase S6** *Closest Gen 1 homolog to genotype 2 specific Peptidase S6*	**Gen 1 iso 2**	723	--	722
	**Gen 1 iso 3**	723	--	722
	**Gen 1 iso 4**	723	--	722
	**Gen 1 iso 1**	723	--	722
	184 iso 1*	1301	SRNHANAEFDEGVNGKSNLLMASLYGKWHSENGTFVSLDGSYGKAKNRIDLFGENRFNRHIMAIGANLGHNFDLAGVQVQPAVGTRYYRFSAQNYKLGEV	1400
**Peptidase S6 Genotype 2 specific**	**Gen 2 iso 5**	1301	SRNHANAEFDEGVNGKSNLLMASLYGKWHSENGTFVSLDGSYGKAKNRIDLFGENRFNRHIMAIGANLGHNFDLAGVQVQPAVGTRYYRFSAQNYKLGEV	1400
	**Gen 2 iso 4**	1301	SRNHANAEFDEGVNGKSNLLMASLYGKWHSENGTFVSLDGSYGKAKNRIDLFGENRFNRHIMAIGANLGHNFDLAGVQVQPAVGTRYYRFSAQNYKLGEV	1400
	**Gen 2 iso 3**	1301	SRNHANAEFDEGVNGKSNLLMASLYGKWHSENGTFVSLDGSYGKAKNRIDLFGENRFNRHIMAIGANLGHNFDLAGVQVQPAVGTRYYRFSAQNYKLGEV	1400
	**Gen 2 iso 2**	1301	SRNHANAEFDEGVNGKSNLLMASLYGKWHSENGTFVSLDGSYGKAKNRIDLFGENRFNRHIMAIGANLGHNFDLAGVQVQPAVGTRYYRFSAQNYKLGEV	1400
	**Gen 2 iso 1**	1301	SRNHANAEFDEGVNGKSNLLMASLYGKWHSENGTFVSLDGSYGKAKNRIDLFGENRFNRHIMAIGANLGHNFDLAGVQVQPAVGTRYYRFSAQNYKLGEV	1400

Group	Sequence	Start	Alignment	End
**Peptidase S6** *Closest Gen 1 homolog to genotype 2 specific Peptidase S6*	**Gen 1 iso 2**	723	--	722
	**Gen 1 iso 3**	723	--	722
	**Gen 1 iso 4**	723	--	722
	**Gen 1 iso 1**	723	--	722
	184 iso 1*	1401	EVRSPKAHFMAYQAGVKVSKAFDLSSWKVEPSLAAHYVDASSKRLSVVVNDNTFSQRFGRYLKTEVGVGVTKGQWQLSSHLGLLKGNEIGKQHFAGFKLG	1500
**Peptidase S6 Genotype 2 specific**	**Gen 2 iso 5**	1401	EVRSPKAHFMAYQAGVKVSKAFDLSSWKVEPSLAAHYVDASSKRLSVAVNDNTFSQRFGRYLKTEVGVGVTKGQWQLSSHLGLLKGNEIGKQHFAGFKLG	1500
	**Gen 2 iso 4**	1401	EVRSPKAHFMAYQAGVKVSKAFDLSSWKVEPSLAAHYVDASSKRLSVAVNDNTFSQRFGRYLKTEVGVGVTKGQWQLSSHLGLLKGNEIGKQHFAGFKLG	1500
	**Gen 2 iso 3**	1401	EVRSPKAHFMAYQAGVKVSKAFDLSSWKVEPSLAAHYVDASSKRLSVAVNDNTFSQRFGRYLKTEVGVGVTKGQWQLSSHLGLLKGNEIGKQHFAGFKLG	1500
	**Gen 2 iso 2**	1401	EVRSPKAHFMAYQAGVKVSKAFDLSSWKVEPSLAAHYVDASSKRLSVAVNDNTFSQRFGRYLKTEVGVGVTKGQWQLSSHLGLLKGNEIGKQHFAGFKLG	1500
	**Gen 2 iso 1**	1401	EVRSPKAHFMAYQAGVKVSKAFDLSSWKVEPSLAAHYVDASSKRLSVAVNDNTFSQRFGRYLKTEVGVGVTKGQWQLSSHLGLLKGNEIGKQHFAGFKLG	1500

Group	Sequence	Start	Alignment	End
**Peptidase S6** *Closest Gen 1 homolog to genotype 2 specific Peptidase S6*	**Gen 1 iso 2**	723		722
	**Gen 1 iso 3**	723		722
	**Gen 1 iso 4**	723		722
	**Gen 1 iso 1**	723		722
	184 iso 1*	1501	YSW	1503
**Peptidase S6 Genotype 2 specific**	**Gen 2 iso 5**	1501	YSW*	1504
	**Gen 2 iso 4**	1501	YSW*	1504
	**Gen 2 iso 3**	1501	YSW*	1504
	**Gen 2 iso 2**	1501	YSW*	1504
	**Gen 2 iso 1**	1501	YSW*	1504
