# Supplementary material for: Differences between predicted outer membrane proteins of genotype 1 and 2 Mannheimia haemolytica
Source: BMC Microbiol. 2020 Aug 12;20:250. doi: 10.1186/s12866-020-01932-2 (PMC7424683; doi:10.1186/s12866-020-01932-2)

Fig S3

Group	Sequence	Start	Alignment	End
AOMB-BD-CP Genotype 1	CP017495 (1b) locus BG548_08360	1	MKRLRYSYLALILGSALPLSAYADGYLSNDNIRQFDARFNFFLANPLPAPIAKADGRGEAIENLVVKYASDYLNDIYLKEDVIRFRRWQWQDLYTSLDLK	100
	CP017510 (1c) locus BG556_08335	1	MKRLRYSYLALILGSALPLSAYADGYLSNDNIRQFDARFNFFLANPLPAPIAKADGRGEAIENLVVKYASDYLNDIYLKEDVIRFRRWQWQDLYTSLDLK	100
	CP017502 (1e) locus BG561_08395	1	MKRLRYSYLALILGSALPLSAYADGYLSNDNIRQFDARFNFFLANPLPAPIAKADGRGEAIENLVVKYASDYLNDIYLKEDVIRFRRWQWQDLYTSLDLK	100
	CP017484 (1f) locus BG572_08380	1	MKRLRYSYLALILGSALPLSAYADGYLSNDNIRQFDARFNFFLANPLPAPIAKADGRGEAIENLVVKYASDYLNDIYLKEDVIRFRRWQWQDLYTSLDLK	100
	CP017499 (1i) locus BG576_04685	1	MKRLRYSYLALILGSALPLSAYADGYLSNDNIRQFDARFNFFLANPLPAPIAKADGRGEAIENLVVKYASDYLNDIYLKEDVIRFRRWQWQDLYTSLDLK	100
AOMB-BD-CP Genotype 2	CP017538 (2b) locus BG586_08670	1	MKRLRYSYLALILGSALPLSAYADGYLSNDNIRQFDARFNFFLANPLPAPIAKADGRGEAIENLVVKYASDYLNDIYLKEDVIRFRRWQWQDLYTSLDLK	100
	CP017491 (2c) locus BG598_09500	1	MKRLRYSYLALILGSALPLSAYADGYLSNDNIRQFDARFNFFLANPLPAPIAKADGRGEAIENLVVKYASDYLNDIYLKEDVIRFRRWQWQDLYTSLDLK	100
	CP017505 (2d) locus BG605_10030	1	MKRLRYSYLALILGSALPLSAYADGYLSNDNIRQFDARFNFFLANPLPAPIAKADGRGEAIENLVVKYASDYLNDIYLKEDVIRFRRWQWQDLYTSLDLK	100
	CP017552 (2e) locus BG607_09820	1	MKRLRYSYLALILGSALPLSAYADGYLSNDNIRQFDARFNFFLANPLPAPIAKADGRGEAIENLVVKYASDYLNDIYLKEDVIRFRRWQWQDLYTSLDLK	100
AOMB-BD-CP Genotype 1	CP017495 (1b) locus BG548_12060	1		0
	CP017510 (1c) locus BG556_12500	1		0
	CP017502 (1e) locus BG561_11975	1		0
	CP017484 (1f) locus BG572_12085	1		0
	CP017499 (1i) locus BG576_11950	1		0
AOMB-BD-CP Genotype 2	CP017538 (2b) locus BG586_05180	1		0
	CP017491 (2c) locus BG598_12675	1		0
	CP017505 (2d) locus BG605_13210	1		0
	CP017552 (2e) locus BG607_12995	1		0
AOMB-BD-CP Genotype 2 specific*	CP017538 (2b) locus BG586_06725	1		0
	CP017491 (2c) locus BG598_11445	1		0
	CP017505 (2d) locus BG605_11975	1		0
	CP017552 (2e) locus BG607_11765	1		0

Group	Sequence	Start	Alignment	End
AOMB-BD-CP Genotype 1	CP017495 (1b) locus BG548_08360	101	TKQKPDYLNTYRLQAEAYFINKQYQEALSQLDQILRRNPNDVHAMAMSVVASRVLENPNTEAERLLALYQISPSIAQKVQQYLHFTDTMLAASYGSEPQT	200
	CP017510 (1c) locus BG556_08335	101	TKQKPDYLNTYRLQAEAYFINKQYQEALSQLDQILRRNPNDVHAMAMSVVASRVLENPNTEAERLLALYQISPSIAQKVQQYLHFTDTMLAASYGSEPQT	200
	CP017502 (1e) locus BG561_08395	101	TKQKPDYLNTYRLQAEAYFINKQYQEALSQLDQILRRNPNDVHAMAMSVVASRVLENPNTEAERLLALYQISPSIAQKVQQYLHFTDTMLAASYGSEPQT	200
	CP017484 (1f) locus BG572_08380	101	TKQKPDYLNTYRLQAEAYFINKQYQEALSQLDQILRRNPNDVHAMAMSVVASRVLENPNTEAERLLALYQISPSIAQKVQQYLHFTDTMLAASYGSEPQT	200
	CP017499 (1i) locus BG576_04685	101	TKQKPDYLNTYRLQAEAYFINKQYQEALSQLDQILRRNPNDVHAMAMSVVASRVLENPNTEAERLLALYQISPSIAQKVQQYLHFTDTMLAASYGSEPQT	200
AOMB-BD-CP Genotype 2	CP017538 (2b) locus BG586_08670	101	TKQKPDYLNTYRLQAEAYFINKQYQEALSQLDQILRRNPNDVHAMAMSVVASRVLENPNTEAERLLALYQISPSIAQKVQQYLHFTDTMLAASYGSEPQT	200
	CP017491 (2c) locus BG598_09500	101	TKQKPDYLNTYRLQAEAYFINKQYQEALSQLDQILRRNPNDVHAMAMSVVASRVLENPNTEAERLLALYQISPSIAQKVQQYLHFTDTMLAASYGSEPQT	200
	CP017505 (2d) locus BG605_10030	101	TKQKPDYLNTYRLQAEAYFINKQYQEALSQLDQILRRNPNDVHAMAMSVVASRVLENPNTEAERLLALYQISPSIAQKVQQYLHFTDTMLAASYGSEPQT	200
	CP017552 (2e) locus BG607_09820	101	TKQKPDYLNTYRLQAEAYFINKQYQEALSQLDQILRRNPNDVHAMAMSVVASRVLENPNTEAERLLALYQISPSIAQKVQQYLHFTDTMLAASYGSEPQT	200
AOMB-BD-CP Genotype 1	CP017495 (1b) locus BG548_12060	1	--	0
	CP017510 (1c) locus BG556_12500	1	--	0
	CP017502 (1e) locus BG561_11975	1	--	0
	CP017484 (1f) locus BG572_12085	1	--	0
	CP017499 (1i) locus BG576_11950	1	--	0
AOMB-BD-CP Genotype 2	CP017538 (2b) locus BG586_05180	1	--	0
	CP017491 (2c) locus BG598_12675	1	--	0
	CP017505 (2d) locus BG605_13210	1	--	0
	CP017552 (2e) locus BG607_12995	1	--	0
AOMB-BD-CP Genotype 2 specific*	CP017538 (2b) locus BG586_06725	1	--	0
	CP017491 (2c) locus BG598_11445	1	--	0
	CP017505 (2d) locus BG605_11975	1	--	0
	CP017552 (2e) locus BG607_11765	1	--	0

Fig S3 continued

AOMB-BD-CP Genotype 1
CP017495 (1b) locus BG548_08360 201 TMIPDTIAVFGQSPNPDGTPSKGLLQRLEKTKEMAAKFPNAKIILSGGPVRYIYAEADVMKKWLIENGVDESRLLLDDVARDTPGNAVGIIDFMKEYGGR 300
CP017510 (1c) locus BG556_08335 201 TMIPDTIAVFGQSPNPDGTPSKGLLQRLEKTKEMAAKFPNAKIILSGGPVRYIYAEADVMKKWLIENGVDESRLLLDDVARDTPGNAVGIIDFMKEYGGR 300
CP017502 (1e) locus BG561_08395 201 TMIPDTIAVFGQSPNPDGTPSKGLLQRLEKTKEMAAKFPNAKIILSGGPVRYIYAEADVMKKWLIENGVDESRLLLDDVARDTPGNAVGIIDFMKEYGGR 300
CP017484 (1f) locus BG572_08380 201 TMIPDTIAVFGQSPNPDGTPSKGLLQRLEKTKEMAAKFPNAKIILSGGPVRYIYAEADVMKKWLIENGVDESRLLLDDVARDTPGNAVGIIDFMKEYGGR 300
CP017499 (1i) locus BG576_04685 201 TMIPDTIAVFGQSPNPDGTPSKGLLQRLEKTKEMAAKFPNAKIILSGGPVRYIYAEADVMKKWLIENGVDESRLLLDDVARDTPGNAVGIIDFMKEYGGR 300

AOMB-BD-CP Genotype 2
CP017538 (2b) locus BG586_08670 201 TMIPDTIAVFGQSPNPDGTPSKGLLQRLEKTKEMAEKFPNAKIILSGGPVRYIYAEADVMKKWLIENGVDESRLLLDDVARDTPGNAVGIIDFMKEYGGR 300
CP017491 (2c) locus BG598_09500 201 TMIPDTIAVFGQSPNPDGTPSKGLLQRLEKTKEMAEKFPNAKIILSGGPVRYIYAEADVMKKWLIENGVDESRLLLDDVARDTPGNAVGIIDFMKEYGGR 300
CP017505 (2d) locus BG605_10030 201 TMIPDTIAVFGQSPNPDGTPSKGLLQRLEKTKEMAEKFPNAKIILSGGPVRYIYAEADVMKKWLIENGVDESRLLLDDVARDTPGNAVGIIDFMKEYGGR 300
CP017552 (2e) locus BG607_09820 201 TMIPDTIAVFGQSPNPDGTPSKGLLQRLEKTKEMAEKFPNAKIILSGGPVRYIYAEADVMKKWLIENGVDESRLLLDDVARDTPGNAVGIIDFMKEYGGR 300

AOMB-BD-CP Genotype 1
CP017495 (1b) locus BG548_12060 1 -- 0
CP017510 (1c) locus BG556_12500 1 -- 0
CP017502 (1e) locus BG561_11975 1 -- 0
CP017484 (1f) locus BG572_12085 1 -- 0
CP017499 (1i) locus BG576_11950 1 -- 0

AOMB-BD-CP Genotype 2
CP017538 (2b) locus BG586_05180 1 -- 0
CP017491 (2c) locus BG598_12675 1 -- 0
CP017505 (2d) locus BG605_13210 1 -- 0
CP017552 (2e) locus BG607_12995 1 -- 0

AOMB-BD-CP Genotype 2 specific*
CP017538 (2b) locus BG586_06725 1 -- 0
CP017491 (2c) locus BG598_11445 1 -- 0
CP017505 (2d) locus BG605_11975 1 -- 0
CP017552 (2e) locus BG607_11765 1 -- 0

AOMB-BD-CP Genotype 1
CP017495 (1b) locus BG548_08360 301 KVLGIGTILHLPRAMSVLKSYADSIGYELEIDSAGGGSPPNEKNKKGEALYTFVNVARAMGLFTLGDFENLLAKEQAEKEQREALEKAQQNYLEKLVAER 400
CP017510 (1c) locus BG556_08335 301 KVLGIGTILHLPRAMSVLKSYADSIGYELEIDSAGGGSPPNEKNKKGEALYTFVNVARAMGLFTLGDFENLLAKEQAEKEQREALEKAQQNYLEKLVAER 400
CP017502 (1e) locus BG561_08395 301 KVLGIGTILHLPRAMSVLKSYADSIGYELEIDSAGGGSPPNEKNKKGEALYTFVNVARAMGLFTLGDFENLLAKEQAEKEQREALEKAQQNYLEKLVAER 400
CP017484 (1f) locus BG572_08380 301 KVLGIGTILHLPRAMSVLKSYADSIGYELEIDSAGGGSPPNEKNKKGEALYTFVNVARAMGLFTLGDFENLLAKEQAEKEQREALEKAQQNYLEKLVAER 400
CP017499 (1i) locus BG576_04685 301 KVLGIGTILHLPRAMSVLKSYADSIGYELEIDSAGGGSPPNEKNKKGEALYTFVNVARAMGLFTLGDFENLLAKEQAEKEQREALEKAQQNYLEKLVAER 400

AOMB-BD-CP Genotype 2
CP017538 (2b) locus BG586_08670 301 KVLGIGTILHLPRAMSVLKSYADSIGYELEIDSAGGGSPPNEKNKKGEALYTFVNVARAMGLFTLGDFENLLAKEQAEKEQREALEKAQQNYLEKLVAER 400
CP017491 (2c) locus BG598_09500 301 KVLGIGTILHLPRAMSVLKSYADSIGYELEIDSAGGGSPPNEKNKKGEALYTFVNVARAMGLFTLGDFENLLAKEQAEKEQREALEKAQQNYLEKLVAER 400
CP017505 (2d) locus BG605_10030 301 KVLGIGTILHLPRAMSVLKSYADSIGYELEIDSAGGGSPPNEKNKKGEALYTFVNVARAMGLFTLGDFENLLAKEQAEKEQREALEKAQQNYLEKLVAER 400
CP017552 (2e) locus BG607_09820 301 KVLGIGTILHLPRAMSVLKSYADSIGYELEIDSAGGGSPPNEKNKKGEALYTFVNVARAMGLFTLGDFENLLAKEQAEKEQREALEKAQQNYLEKLVAER 400

AOMB-BD-CP Genotype 1
CP017495 (1b) locus BG548_12060 1 --MILGAALNYSKAKVNFDRYGGSS--------------------KADGIGASLYARIGNKHKTPWYLQGRAGYGSVDSDVERHIILADNNASTAKINHR 78
CP017510 (1c) locus BG556_12500 1 --MILGAALNYSKAKVNFDRYGGSS--------------------KADGIGASLYARIGNKHKTPWYLQGRAGYGSVDSDVERHIILADNNASTAKINHR 78
CP017502 (1e) locus BG561_11975 1 --MILGAALNYSKAKVNFDRYGGSS--------------------KADGIGASLYARIGNKHKTPWYLQGRAGYGSVDSDVERHIILADNNASTAKINHR 78
CP017484 (1f) locus BG572_12085 1 --MILGAALNYSKAKVNFDRYGGSS--------------------KADGIGASLYARIGNKHKTPWYLQGRAGYGSVDSDVERHIILADNNASTAKINHR 78
CP017499 (1i) locus BG576_11950 1 --MILGAALNYSKAKVNFDRYGGSS--------------------KADGIGASLYARIGNKHKTPWYLQGRAGYGSVDSDVERHIILADNNASTAKINHR 78

AOMB-BD-CP Genotype 2
CP017538 (2b) locus BG586_05180 1 --MILGAALNYSKAKVNFDRYGGSS--------------------KADGIGASLYARIGNKHKTPWYLQGRAGYGSVDSDVERHIILADNNASTAKINHR 78
CP017491 (2c) locus BG598_12675 1 --MILGAALNYSKAKVNFDRYGGSS--------------------KADGIGASLYARIGNKHKTPWYLQGRAGYGSVDSDVERHIILADNNASTAKINHR 78
CP017505 (2d) locus BG605_13210 1 --MILGAALNYSKAKVNFDRYGGSS--------------------KADGIGASLYARIGNKHKTPWYLQGRAGYGSVDSDVERHIILADNNASTAKINHR 78
CP017552 (2e) locus BG607_12995 1 --MILGAALNYSKAKVNFDRYGGSS--------------------KADGIGASLYARIGNKHKTPWYLQGRAGYGSVDSDVERHIILADNNASTAKINHR 78

AOMB-BD-CP Genotype 2 specific*
CP017538 (2b) locus BG586_06725 1 ----------------MFSFYGKKT--------------------WENGIYVGIDGSIGRANNKLSHQS-------------QTIKLARN---------- 41
CP017491 (2c) locus BG598_11445 1 ----------------MFSFYGKKT--------------------WENGIYVGIDGSIGRANNKLSHQS-------------QTIKLARN---------- 41
CP017505 (2d) locus BG605_11975 1 ----------------MFSFYGKKT--------------------WENGIYVGIDGSIGRANNKLSHQS-------------QTIKLARN---------- 41
CP017552 (2e) locus BG607_11765 1 ----------------MFSFYGKKT--------------------WENGIYVGIDGSIGRANNKLSHQS-------------QTIKLARN---------- 41

Fig S3 continued

Group	Sequence	Start	Alignment	End
AOMB-BD-CP Genotype 1	CP017495 (1b) locus BG548_08360	401	DQAVKNAENSANELEVARQALETAKQAHQQAEEELNKELSKTQEQLKQSTEALSRLIIKQNNPFSEHYVVQLNLMEQATYDLYRRLAEIEHTQDWNLWLS	500
	CP017510 (1c) locus BG556_08335	401	DQAVKNAENSANELEVARQALETAKQAHQQAEEELNKELSKTQEQLKQSTEALSRLIIKQNNPFSEHYVVQLNLMEQATYDLYRRLAEIEHTQDWNLWLS	500
	CP017502 (1e) locus BG561_08395	401	DQAVKNAENSANELEVARQALETAKQAHQQAEEELNKELSKTQEQLKQSTEALSRLIIKQNNPFSEHYVVQLNLMEQATYDLYRRLAEIEHTQDWNLWLS	500
	CP017484 (1f) locus BG572_08380	401	DQAVKNAENSANELEVARQALETAKQAHQQAEEELNKELSKTQEQLKQSTEALSRLIIKQNNPFSEHYVVQLNLMEQATYDLYRRLAEIEHTQDWNLWLS	500
	CP017499 (1i) locus BG576_04685	401	DQAVKNAENSANELEVARQALETAKQAHQQAEEELNKELSKTQEQLKQSTEALSRLIIKQNNPFSEHYVVQLNLMEQATYDLYRRLAEIEHTQDWNLWLS	500
AOMB-BD-CP Genotype 2	CP017538 (2b) locus BG586_08670	401	DQAVKNAENSANELEVARQALETAKQAHQQAEEELNKELSKTQEQLKQSTEALSRLIIKQNNPFSEHYVVQLNLMEQATYDLYRRLAEIEHTQDWNLWLS	500
	CP017491 (2c) locus BG598_09500	401	DQAVKNAENSANELEVARQALETAKQAHQQAEEELNKELSKTQEQLKQSTEALSRLIIKQNNPFSEHYVVQLNLMEQATYDLYRRLAEIEHTQDWNLWLS	500
	CP017505 (2d) locus BG605_10030	401	DQAVKNAENSANELEVARQALETAKQAHQQAEEELNKELSKTQEQLKQSTEALSRLIIKQNNPFSEHYVVQLNLMEQATYDLYRRLAEIEHTQDWNLWLS	500
	CP017552 (2e) locus BG607_09820	401	DQAVKNAENSANELEVARQALETAKQAHQQAEEELNKELSKTQEQLKQSTEALSRLIIKQNNPFSEHYVVQLNLMEQATYDLYRRLAEIEHTQDWNLWLS	500
AOMB-BD-CP Genotype 1	CP017495 (1b) locus BG548_12060	79	DRVFSAYVESGYDFKQGRFALTPFVGFSHDVVRRGAFSE--QNSQFGLTADKATYKQTSGLIGLRTSLEVNLAGMKTTFQGYVNHQKAFN--------RE	168
	CP017510 (1c) locus BG556_12500	79	DRVFSAYVESGYDFKQGRFALTPFVGFSHDVVRRGAFSE--QNSQFGLTADKATYKQTSGLIGLRTSLEVNLAGMKTTFQGYVNHQKAFN--------RE	168
	CP017502 (1e) locus BG561_11975	79	DRVFSAYVESGYDFKQGRFALTPFVGFSHDVVRRGAFSE--QNSQFGLTADKATYKQTSGLIGLRTSLEVNLAGMKTTFQGYVNHQKAFN--------RE	168
	CP017484 (1f) locus BG572_12085	79	DRVFSAYVESGYDFKQGRFALTPFVGFSHDVVRRGAFSE--QNSQFGLTADKATYKQTSGLIGLRTSLEVNLAGMKTTFQGYVNHQKAFN--------RE	168
	CP017499 (1i) locus BG576_11950	79	DRVFSAYVESGYDFKQGRFALTPFVGFSHDVVRRGAFSE--QNSQFGLTADKATYKQTSGLIGLRTSLEVNLAGMKTTFQGYVNHQKAFN--------RE	168
AOMB-BD-CP Genotype 2	CP017538 (2b) locus BG586_05180	79	DRVFSAYVESGYDFKQGRFALTPFVGFSHDVVRRGAFSE--QNSQFGLTADKATYKQTSGLIGLRTSLEVNLAGMKTTFQGYVNHQKAFN--------RE	168
	CP017491 (2c) locus BG598_12675	79	DRVFSAYVESGYDFKQGRFALTPFVGFSHDVVRRGAFSE--QNSQFGLTADKATYKQTSGLIGLRTSLEVNLAGMKTTFQGYVNHQKAFN--------RE	168
	CP017505 (2d) locus BG605_13210	79	DRVFSAYVESGYDFKQGRFALTPFVGFSHDVVRRGAFSE--QNSQFGLTADKATYKQTSGLIGLRTSLEVNLAGMKTTFQGYVNHQKAFN--------RE	168
	CP017552 (2e) locus BG607_12995	79	DRVFSAYVESGYDFKQGRFALTPFVGFSHDVVRRGAFSE--QNSQFGLTADKATYKQTSGLIGLRTSLEVNLAGMKTTFQGYVNHQKAFN--------RE	168
AOMB-BD-CP Genotype 2 specific*	CP017538 (2b) locus BG586_06725	42	--VYSLGLTVGKTLEVASFNLQPSFGMRYHHLTSANAQL--ENTKF--ETDRVDLLAIQAGLALNKTFIFNELKVKPELGSYFVDASHGT--------LK	127
	CP017491 (2c) locus BG598_11445	42	--VYSLGLTVGKTLEVASFNLQPSFGMRYHHLTSANAQL--ENTKF--ETDRVDLLAIQAGLALNKTFIFNELKVKPELGSYFVDASHGT--------LK	127
	CP017505 (2d) locus BG605_11975	42	--VYSLGLTVGKTLEVASFNLQPSFGMRYHHLTSANAQL--ENTKF--ETDRVDLLAIQAGLALNKTFIFNELKVKPELGSYFVDASHGT--------LK	127
	CP017552 (2e) locus BG607_11765	42	--VYSLGLTVGKTLEVASFNLQPSFGMRYHHLTSANAQL--ENTKF--ETDRVDLLAIQAGLALNKTFIFNELKVKPELGSYFVDASHGT--------LK	127

Group	Sequence	Start	Alignment	End
AOMB-BD-CP Genotype 1	CP017495 (1b) locus BG548_08360	501	DSLSKYRLKNRSTEDYHLLHIGLDHQVSHNWSIGGVFGISQGQIENRTHKVKGTSAGIYAGYRPTSGLFWTNFFNYSHFTNQSGVTKRDNNAYSLSSEIG	600
	CP017510 (1c) locus BG556_08335	501	DSLSKYRLKNRSTEDYHLLHIGLDHQVSHNWSIGGVFGISQGQIENRTHKVKGTSAGIYAGYRPTSGLFWTNFFNYSHFTNQSGVTKRDNNAYSLSSEIG	600
	CP017502 (1e) locus BG561_08395	501	DSLSKYRLKNRSTEDYHLLHIGLDHQVSHNWSIGGVFGISQGQIENRTHKVKGTSAGIYAGYRPTSGLFWTNFFNYSHFTNQSGVTKRDNNAYSLSSEIG	600
	CP017484 (1f) locus BG572_08380	501	DSLSKYRLKNRSTEDYHLLHIGLDHQVSHNWSIGGVFGISQGQIENRTHKVKGTSAGIYAGYRPTSGLFWTNFFNYSHFTNQSGVTKRDNNAYSLSSEIG	600
	CP017499 (1i) locus BG576_04685	501	DSLSKYRLKNRSTEDYHLLHIGLDHQVSHNWSIGGVFGISQGQIENRTHKVKGTSAGIYAGYRPTSGLFWTNFFNYSHFTNQSGVTKRDNNAYSLSSEIG	600
AOMB-BD-CP Genotype 2	CP017538 (2b) locus BG586_08670	501	DSLSKYRLKNRSTEDYHLLHIGLDHQVSHNWSIGGVFGISQGQIENRTHKVKGTSAGIYAGYRPTSGLFWTNFFNYSHFTNQSGVTKRDNNAYSLSSEIG	600
	CP017491 (2c) locus BG598_09500	501	DSLSKYRLKNRSTEDYHLLHIGLDHQVSHNWSIGGVFGISQGQIENRTHKVKGTSAGIYAGYRPTSGLFWTNFFNYSHFTNQSGVTKRDNNAYSLSSEIG	600
	CP017505 (2d) locus BG605_10030	501	DSLSKYRLKNRSTEDYHLLHIGLDHQVSHNWSIGGVFGISQGQIENRTHKVKGTSAGIYAGYRPTSGLFWTNFFNYSHFTNQSGVTKRDNNAYSLSSEIG	600
	CP017552 (2e) locus BG607_09820	501	DSLSKYRLKNRSTEDYHLLHIGLDHQVSHNWSIGGVFGISQGQIENRTHKVKGTSAGIYAGYRPTSGLFWTNFFNYSHFTNQSGVTKRDNNAYSLSSEIG	600
AOMB-BD-CP Genotype 1	CP017495 (1b) locus BG548_12060	169	DLSFKASYTGLQDAKFDVKGIGLAKHK--------------------------TWVGIGALAEVN-----------------------KNTSLYVNYDLK	219
	CP017510 (1c) locus BG556_12500	169	DLSFKASYTGLQDAKFDVKGIGLAKHK--------------------------TWVGIGALAEVN-----------------------KNTSLYVNYDLK	219
	CP017502 (1e) locus BG561_11975	169	DLSFKASYTGLQDAKFDVKGIGLAKHK--------------------------TWVGIGALAEVN-----------------------KNTSLYVNYDLK	219
	CP017484 (1f) locus BG572_12085	169	DLSFKASYTGLQDAKFDVKGIGLAKHK--------------------------TWVGIGALAEVN-----------------------KNTSLYVNYDLK	219
	CP017499 (1i) locus BG576_11950	169	DLSFKASYTGLQDAKFDVKGIGLAKHK--------------------------TWVGIGALAEVN-----------------------KNTSLYVNYDLK	219
AOMB-BD-CP Genotype 2	CP017538 (2b) locus BG586_05180	169	DLSFKASYTGLQDAKFDVKGIGLAKHK--------------------------TWVGIGALAEVN-----------------------KNTSLYVNYDLK	219
	CP017491 (2c) locus BG598_12675	169	DLSFKASYTGLQDAKFDVKGIGLAKHK--------------------------TWVGIGALAEVN-----------------------KNTSLYVNYDLK	219
	CP017505 (2d) locus BG605_13210	169	DLSFKASYTGLQDAKFDVKGIGLAKHK--------------------------TWVGIGALAEVN-----------------------KNTSLYVNYDLK	219
	CP017552 (2e) locus BG607_12995	169	DLSFKASYTGLQDAKFDVKGIGLAKHK--------------------------TWVGIGALAEVN-----------------------KNTSLYVNYDLK	219
OMB-BD-CP Genotype 2 specific*	CP017538 (2b) locus BG586_06725	128	TKLNEFSLN-QNIGRYFKQEVGITLHY--------------------------KGVSSSVHAGFT-----------------------KGNTLQE-----	172
	CP017491 (2c) locus BG598_11445	128	TKLNEFSLN-QNIGRYFKQEVGITLHY--------------------------KGVSSSVHAGFT-----------------------KGNTLQE-----	172
	CP017505 (2d) locus BG605_11975	128	TKLNEFSLN-QNIGRYFKQEVGITLHY--------------------------KGVSSSVHAGFT-----------------------KGNTLQE-----	172
	CP017552 (2e) locus BG607_11765	128	TKLNEFSLN-QNIGRYFKQEVGITLHY--------------------------KGVSSSVHAGFT-----------------------KGNTLQE-----	172

Fig S3 continued

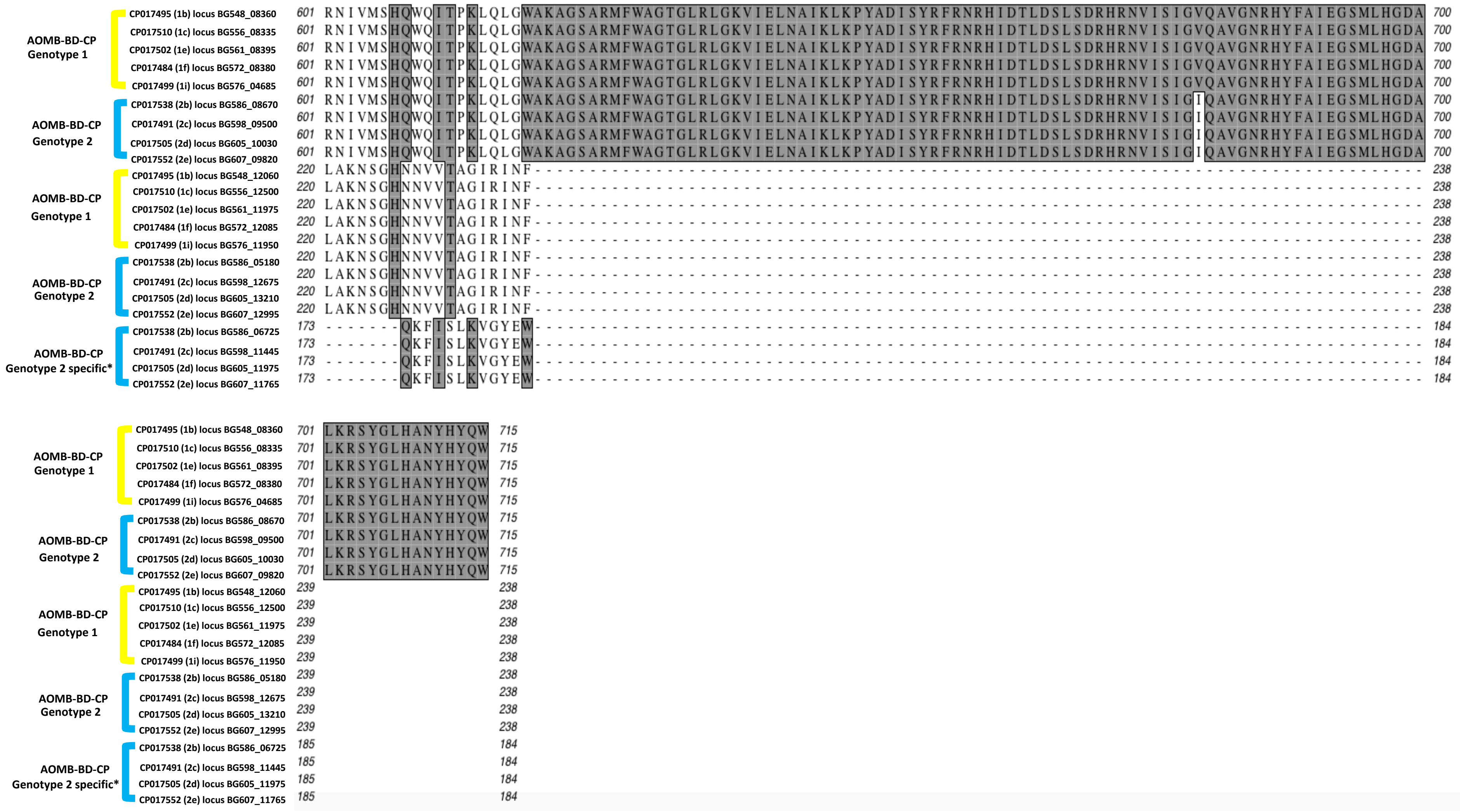

Supplement: Supplementary file 8 — Additional file 8: Figure S3.. Alignment of autotransporter outer membrane beta-barrel domain-containing protein (AOMB-BP-CP) isoforms. The alignment contains all annotated AOMB-BP-CP proteins from five genotype 1 and four genotype 2 M. haemolytica strains that are each of a different subtype. The AOMB-BP-CP proteins flagged by EDGAR as specific to genotype 2 M. haemolytica are indicated with an asterisk. Areas of 51% chemical identity or greater are indicated with grey boxes. [file 12866_2020_1932_MOESM8_ESM.pdf]
