# Supplementary material for: Differences between predicted outer membrane proteins of genotype 1 and 2 Mannheimia haemolytica
Source: BMC Microbiol. 2020 Aug 12;20:250. doi: 10.1186/s12866-020-01932-2 (PMC7424683; doi:10.1186/s12866-020-01932-2)
Supplement: Supplementary file 9 — Additional file 9: Figure S4. Alignment of genotype 2 AOMB-BP-CP proteins flagged as specific to genotype 2 M. haemolytica with genotype 1 peptidase S6 protein and genotype 2 S6 family IgA-specific metalloendopeptidase protein. The alignment contains peptidase S6 protein sequence from five genotype 1 strains of different subtypes, S6 family IgA-specific metalloendopeptidase protein sequence from four genotype 2 strains of different subtypes, and AOMB-BP-CP protein sequence from four genotype 2 strains of different subtypes. The AOMB-BP-CP proteins flagged by EDGAR as specific to genotype 2 M. haemolytica are denoted by an asterisk. The alignment additionally contains translated nucleotides immediately upstream of, in-frame, and concatenated to the AOMB-BP-CP protein sequence for each of the genotype 2 strains representing different subtypes. The black arrow highlights the location of a stop codon in the concatenated sequences. Areas of greater than 51% chemical identity are indicated with grey boxes. [file 12866_2020_1932_MOESM9_ESM.pdf]

Fig S4

Group	Sequence	Start	Alignment	End
Peptidase S6 Genotype 1	CP017495 (1b) locus BG548_02680	1	MKHTFKLSLLATSIAISLSPTLLASIVRGDVDYQYFRDLAENKGQFFVGATNIPVIDKTGKNIGTFLQVTPTKQVEVESNISPKPNVSLSECFDNESLPG	100
	CP017510 (1c) locus BG556_02675	1	MKHTFKLSLLATSIAISLSPTLLASIVRGDVDYQYFRDLAENKGQFFVGATNIPVIDKTGKNIGTFLQVTPTKQVEVESNISPKPNVSLSECFDNESLPG	100
	CP017502 (1e) locus BG561_02675	1	MKHTFKLSLLATSIAISLSPTLLASIVRGDVDYQYFRDLAENKGQFFVGATNIPVIDKTGKNIGTFLQVTPTKQVEVESNISPKPNVSLSECFDNESLPG	100
	CP017484 (1f) locus BG572_02680	1	MKHTFKLSLLATSIAISLSPTLLASIVRGDVDYQYFRDLAENKGQFFVGATNIPVIDKTGKNIGTFLQVTPTKQVEVESNISPKPNVSLSECFDNESLPG	100
	CP017499 (1i) locus BG576_10160	1	MKHTFKLSLLATSIAISLSPTLLASIVRGDVDYQYFRDLAENKGQFFVGATNIPVIDKTGKNIGTFLQVTPTKQVEVESNISPKPNVSLSECFDNESLPG	100
S6 family IgA-specific metalloendopeptidase Genotype 2	CP017538 (2b) locus BG586_06725	1		0
	CP017491 (2c) locus BG598_11445	1		0
	CP017505 (2d) locus BG605_11975	1		0
	CP017552 (2e) locus BG607_11765	1		0
Genotype 2	CP017538 (2b) locus BG586_06725 and upstream	1		0
	CP017491 (2c) locus BG598_11445 and upstream	1		0
	CP017505 (2d) locus BG605_11975 and upstream	1		0
	CP017552 (2e) locus BG607_11765 and upstream	1		0
AOMB-BD-CP Genotype 2 specific*	CP017538 (2b) locus BG586_06725	1		0
	CP017491 (2c) locus BG598_11445	1		0
	CP017505 (2d) locus BG605_11975	1		0
	CP017552 (2e) locus BG607_11765	1		0

Group	Sequence	Start	Alignment	End
Peptidase S6 Genotype 1	CP017495 (1b) locus BG548_02680	101	CYEFLYGGGDEDSNDLPVFETVTGETMSIPMIDFSSITRQTGIATLVNPQYVISVNHNQGYRDIQFGDSSSEKADDHHYNYSVVRRNNYMPDPNKERTDL	200
	CP017510 (1c) locus BG556_02675	101	CYEFLYGGGDEDSNDLPVFETVTGETMSIPMIDFSSITRQTGIATLVNPQYVISVNHNQGYRDIQFGDSSSEKADDHHYNYSVVRRNNYMPDPNKERTDL	200
	CP017502 (1e) locus BG561_02675	101	CYEFLYGGGDEDSNDLPVFETVTGETMSIPMIDFSSITRQTGIATLVNPQYVISVNHNQGYRDIQFGDSSSEKADDHHYNYSVVRRNNYMPDPNKERTDL	200
	CP017484 (1f) locus BG572_02680	101	CYEFLYGGGDEDSNDLPVFETVTGETMSIPMIDFSSITRQTGIATLVNPQYVISVNHNQGYRDIQFGDSSSEKADDHHYNYSVVRRNNYMPDPNKERTDL	200
	CP017499 (1i) locus BG576_10160	101	CYEFLYGGGDEDSNDLPVFETVTGETMSIPMIDFSSITRQTGIATLVNPQYVISVNHNQGYRDIQFGDSSSEKADDHHYNYSVVRRNNYMPDPNKERTDL	200
S6 family IgA-specific metalloendopeptidase Genotype 2	CP017538 (2b) locus BG586_06725	1	---MVQAVSFSHFFAIHYFYFFLHKTKGQCTAK---	30
	CP017491 (2c) locus BG598_11445	1	---MVQAVSFSHFFAIHYFYFFLHKTKGQCTAK---	30
	CP017505 (2d) locus BG605_11975	1	---MVQAVSFSHFFAIHYFYFFLHKTKGQCTAK---	30
	CP017552 (2e) locus BG607_11765	1	---MVQAVSFSHFFAIHYFYFFLHKTKGQCTAK---	30
OMB-BD-CP with extra upstream sequence Genotype 2 specific*	CP017538 (2b) locus BG586_06725 and upstream	1	---	0
	CP017491 (2c) locus BG598_11445 and upstream	1	---	0
	CP017505 (2d) locus BG605_11975 and upstream	1	---	0
	CP017552 (2e) locus BG607_11765 and upstream	1	---	0
AOMB-BD-CP Genotype 2 specific*	CP017538 (2b) locus BG586_06725	1	---	0
	CP017491 (2c) locus BG598_11445	1	---	0
	CP017505 (2d) locus BG605_11975	1	---	0
	CP017552 (2e) locus BG607_11765	1	---	0

Fig S4 continued

Group	Sequence	Start	Alignment	End
Peptidase S6 Genotype 1	CP017495 (1b) locus BG548_02680	201	GKGIEKRERNDEGEKLILDGSGNPTPRWDYHAPRLSKLVTEVAPANEIERTTENVQDFYSVFSDPFIFPMFIRAGSGRQAVINNERDRLNNGRIEVGNGP	300
	CP017510 (1c) locus BG556_02675	201	GKGIEKRERNDEGEKLILDGSGNPTPRWDYHAPRLSKLVTEVAPANEIERTTENVQDFYSVFSDPFIFPMFIRAGSGRQAVINNERDRLNNGRIEVGNGP	300
	CP017502 (1e) locus BG561_02675	201	GKGIEKRERNDEGEKLILDGSGNPTPRWDYHAPRLSKLVTEVAPANEIERTTENVQDFYSVFSDPFIFPMFIRAGSGRQAVINNERDRLNNGRIEVGNGP	300
	CP017484 (1f) locus BG572_02680	201	GKGIEKRERNDEGEKLILDGSGNPTPRWDYHAPRLSKLVTEVAPANEIERTTENVQDFYSVFSDPFIFPMFIRAGSGRQAVINNERDRLNNGRIEVGNGP	300
	CP017499 (1i) locus BG576_10160	201	GKGIEKRERNDEGEKLILDGSGNPTPRWDYHAPRLSKLVTEVAPANEIERTTENVQDFYSVFSDPFIFPMFIRAGSGRQAVINNERDRLNNGRIEVGNGP	300
S6 family IgA-specific metalloendopeptidase Genotype 2	CP017538 (2b) locus BG586_06725	31	--------------------------------PLFSILLFHLFPK--------NSAKFLLFFY--FLVLMVLYSN-------------------EVANGK	69
	CP017491 (2c) locus BG598_11445	31	--------------------------------PLFSILLFHLFPK--------NSAKFLLFFY--FLVLMVLYSN-------------------EVANGK	69
	CP017505 (2d) locus BG605_11975	31	--------------------------------PLFSILLFHLFPK--------NSAKFLLFFY--FLVLMVLYSN-------------------EVANGK	69
	CP017552 (2e) locus BG607_11765	31	--------------------------------PLFSILLFHLFPK--------NSAKFLLFFY--FLVLMVLYSN-------------------EVANGK	69
Genotype 2	CP017538 (2b) locus BG586_06725 and upstream	1	--	0
	CP017491 (2c) locus BG598_11445 and upstream	1	--	0
	CP017505 (2d) locus BG605_11975 and upstream	1	--	0
	CP017552 (2e) locus BG607_11765 and upstream	1	--	0
AOMB-BD-CP Genotype 2 specific*	CP017538 (2b) locus BG586_06725	1	--	0
	CP017491 (2c) locus BG598_11445	1	--	0
	CP017505 (2d) locus BG605_11975	1	--	0
	CP017552 (2e) locus BG607_11765	1	--	0

Group	Sequence	Start	Alignment	End
Peptidase S6 Genotype 1	CP017495 (1b) locus BG548_02680	301	FLTGGSVLPVTNGDPSVPGSDFNSRILVAKTENNTINDVFKDHGYGPLTTLGLPGDSGSALFGYDVRTKKWVVLGVYSDYFSENNTPGGDVYKSYWNYHH	400
	CP017510 (1c) locus BG556_02675	301	FLTGGSVLPVTNGDPSVPGSDFNSRILVAKTENNTINDVFKDHGYGPLTTLGLPGDSGSALFGYDVRTKKWVVLGVYSDYFSENNTPGGDVYKSYWNYHH	400
	CP017502 (1e) locus BG561_02675	301	FLTGGSVLPVTNGDPSVPGSDFNSRILVAKTENNTINDVFKDHGYGPLTTLGLPGDSGSALFGYDVRTKKWVVLGVYSDYFSENNTPGGDVYKSYWNYHH	400
	CP017484 (1f) locus BG572_02680	301	FLTGGSVLPVTNGDPSVPGSDFNSRILVAKTENNTINDVFKDHGYGPLTTLGLPGDSGSALFGYDVRTKKWVVLGVYSDYFSENNTPGGDVYKSYWNYHH	400
	CP017499 (1i) locus BG576_10160	301	FLTGGSVLPVTNGDPSVPGSDFNSRILVAKTENNTINDVFKDHGYGPLTTLGLPGDSGSALFGYDVRTKKWVVLGVYSDYFSENNTPGGDVYKSYWNYHH	400
S6 family IgA-specific metalloendopeptidase Genotype 2	CP017538 (2b) locus BG586_06725	70	LLITG-------------GTNLNGTLSAEGTSEIILSGRPTPHAYDKVANKEVLIEG------------DWINRSFNATIFAAKDN--------------	130
	CP017491 (2c) locus BG598_11445	70	LLITG-------------GTNLNGTLSAEGTSEIILSGRPTPHAYDKVANKEVLIEG------------DWINRSFNATIFAAKDN--------------	130
	CP017505 (2d) locus BG605_11975	70	LLITG-------------GTNLNGTLSAEGTSEIILSGRPTPHAYDKVANKEVLIEG------------DWINRSFNATIFAAKDN--------------	130
	CP017552 (2e) locus BG607_11765	70	LLITG-------------GTNLNGTLSAEGTSEIILSGRPTPHAYDKVANKEVLIEG------------DWINRSFNATIFAAKDN--------------	130
OMB-BD-CP with extra upstream sequence Genotype 2 specific*	CP017538 (2b) locus BG586_06725 and upstream	1	--	0
	CP017491 (2c) locus BG598_11445 and upstream	1	--	0
	CP017505 (2d) locus BG605_11975 and upstream	1	--	0
	CP017552 (2e) locus BG607_11765 and upstream	1	--	0
AOMB-BD-CP Genotype 2 specific*	CP017538 (2b) locus BG586_06725	1	--	0
	CP017491 (2c) locus BG598_11445	1	--	0
	CP017505 (2d) locus BG605_11975	1	--	0
	CP017552 (2e) locus BG607_11765	1	--	0

Fig S4 continued

Group	Sequence	Start	Alignment	End
Peptidase S6 Genotype 1	CP017495 (1b) locus BG548_02680	401	PHYVRALEKENNAGAINANGARLTWTPSGNTSSIVGGNAPLTVNLADTSLPTLQGHNGNTGDNPWNPQLQHGKTFHILGENNTLSLTENINQGAGAIHFH	500
	CP017510 (1c) locus BG556_02675	401	PHYVRALEKENNAGAINANGARLTWTPSGNTSSIVGGNAPLTVNLADTSLPTLQGHNGNTGDNPWNPQLQHGKTFHILGENNTLSLTENINQGAGAIHFH	500
	CP017502 (1e) locus BG561_02675	401	PHYVRALEKENNAGAINANGARLTWTPSGNTSSIVGGNAPLTVNLADTSLPTLQGHNGNTGDNPWNPQLQHGKTFHILGENNTLSLTENINQGAGAIHFH	500
	CP017484 (1f) locus BG572_02680	401	PHYVRALEKENNAGAINANGARLTWTPSGNTSSIVGGNAPLTVNLADTSLPTLQGHNGNTGDNPWNPQLQHGKTFHILGENNTLSLTENINQGAGAIHFH	500
	CP017499 (1i) locus BG576_10160	401	PHYVRALEKENNAGAINANGARLTWTPSGNTSSIVGGNAPLTVNLADTSLPTLQGHNGNTGDNPWNPQLQHGKTFHILGENNTLSLTENINQGAGAIHFH	500
S6 family IgA-specific metalloendopeptidase Genotype 2	CP017538 (2b) locus BG586_06725	131	----GKLEISRNVANINGN-----FNLTDNATAQIG--------------------------------FTQGTSQACIRSDRTGVATCNINATLSETDLN	189
	CP017491 (2c) locus BG598_11445	131	----GKLEISRNVANINGN-----FNLTDNATAQIG--------------------------------FTQGTSQACIRSDRTGVATCNINATLSETDLN	189
	CP017505 (2d) locus BG605_11975	131	----GKLEISRNVANINGN-----FNLTDNATAQIG--------------------------------FTQGTSQACIRSDRTGVATCNINATLSETDLN	189
	CP017552 (2e) locus BG607_11765	131	----GKLEISRNVANINGN-----FNLTDNATAQIG--------------------------------FTQGTSQACIRSDRTGVATCNINATLSETDLN	189
Genotype 2	CP017538 (2b) locus BG586_06725 and upstream	1	--	0
	CP017491 (2c) locus BG598_11445 and upstream	1	--	0
	CP017505 (2d) locus BG605_11975 and upstream	1	--	0
	CP017552 (2e) locus BG607_11765 and upstream	1	--	0
AOMB-BD-CP Genotype 2 specific*	CP017538 (2b) locus BG586_06725	1	--	0
	CP017491 (2c) locus BG598_11445	1	--	0
	CP017505 (2d) locus BG605_11975	1	--	0
	CP017552 (2e) locus BG607_11765	1	--	0

Group	Sequence	Start	Alignment	End
Peptidase S6 Genotype 1	CP017495 (1b) locus BG548_02680	501	GNTTVEGMKAGITWLGAGVDIDKDKNVIWKISNPAGDRLSKIGQGTLTINGKGENKGSVSVGDGTVILNQQADENNKKSAFSELGIVSGRPTVILNSSDQ	600
	CP017510 (1c) locus BG556_02675	501	GNTTVEGMKAGITWLGAGVDIDKDKNVIWKISNPAGDRLSKIGQGTLTINGKGENKGSVSVGDGTVILNQQADENNKKSAFSELGIVSGRPTVILNSSDQ	600
	CP017502 (1e) locus BG561_02675	501	GNTTVEGMKAGITWLGAGVDIDKDKNVIWKISNPAGDRLSKIGQGTLTINGKGENKGSVSVGDGTVILNQQADENNKKSAFSELGIVSGRPTVILNSSDQ	600
	CP017484 (1f) locus BG572_02680	501	GNTTVEGMKAGITWLGAGVDIDKDKNVIWKISNPAGDRLSKIGQGTLTINGKGENKGSVSVGDGTVILNQQADENNKKSAFSELGIVSGRPTVILNSSDQ	600
	CP017499 (1i) locus BG576_10160	501	GNTTVEGMKAGITWLGAGVDIDKDKNVIWKISNPAGDRLSKIGQGTLTINGKGENKGSVSVGDGTVILNQQADENNKKSAFSELGIVSGRPTVILNSSDQ	600
S6 family IgA-specific metalloendopeptidase Genotype 2	CP017538 (2b) locus BG586_06725	190	S---------------------------WERTKVAGN-VSLADNSTFSLGSKADLTGSITAQESTKVQLNDGSIAN-LTGLSTTGIFNG-----------	249
	CP017491 (2c) locus BG598_11445	190	S---------------------------WERTKVAGN-VSLADNSTFSLGSKADLTGSITAQESTKVQLNDGSIAN-LTGLSTTGIFNG-----------	249
	CP017505 (2d) locus BG605_11975	190	S---------------------------WERTKVAGN-VSLADNSTFSLGSKADLTGSITAQESTKVQLNDGSIAN-LTGLSTTGIFNG-----------	249
	CP017552 (2e) locus BG607_11765	190	S---------------------------WERTKVAGN-VSLADNSTFSLGSKADLTGSITAQESTKVQLNDGSIAN-LTGLSTTGIFNG-----------	249
OMB-BD-CP with extra upstream sequence Genotype 2 specific*	CP017538 (2b) locus BG586_06725 and upstream	1	--	0
	CP017491 (2c) locus BG598_11445 and upstream	1	--	0
	CP017505 (2d) locus BG605_11975 and upstream	1	--	0
	CP017552 (2e) locus BG607_11765 and upstream	1	--	0
AOMB-BD-CP Genotype 2 specific*	CP017538 (2b) locus BG586_06725	1	--	0
	CP017491 (2c) locus BG598_11445	1	--	0
	CP017505 (2d) locus BG605_11975	1	--	0
	CP017552 (2e) locus BG607_11765	1	--	0

Fig S4 continued

Group	Sequence	Start	Alignment	End
Peptidase S6 Genotype 1	CP017495 (1b) locus BG548_02680	601	MDPNNIYFGYRGGRLDVNGNDLTFRRIQNSDEGAMVVNHNTAQTANITLTGIVSPTIDQVKVQAITSPDQVSRHLDKDIFTYQRFPHLPTNFIRLKKPLS	700
	CP017510 (1c) locus BG556_02675	601	MDPNNIYFGYRGGRLDVNGNDLTFRRIQNSDEGAMVVNHNTAQTANITLTGIVSPTIDQVKVQAITSPDQVSRHLDKDIFTYQRFPHLPTNFIRLKKPLS	700
	CP017502 (1e) locus BG561_02675	601	MDPNNIYFGYRGGRLDVNGNDLTFRRIQNSDEGAMVVNHNTAQTANITLTGIVSPTIDQVKVQAITSPDQVSRHLDKDIFTYQRFPHLPTNFIRLKKPLS	700
	CP017484 (1f) locus BG572_02680	601	MDPNNIYFGYRGGRLDVNGNDLTFRRIQNSDEGAMVVNHNTAQTANITLTGIVSPTIDQVKVQAITSPDQVSRHLDKDIFTYQRFPHLPTNFIRLKKPLS	700
	CP017499 (1i) locus BG576_10160	601	MDPNNIYFGYRGGRLDVNGNDLTFRRIQNSDEGAMVVNHNTAQTANITLTGIVSPTIDQVKVQAITSPDQVSRHLDKDIFTYQRFPHLPTNFIRLKKPLS	700
S6 family IgA-specific metalloendopeptidase Genotype 2	CP017538 (2b) locus BG586_06725	250	---ANGSTVNLSGVWT-TLTNTVVDVLNVANGSQIHLN------------------------	283
	CP017491 (2c) locus BG598_11445	250	---ANGSTVNLSGVWT-TLTNTVVDVLNVANGSQIHLN------------------------	283
	CP017505 (2d) locus BG605_11975	250	---ANGSTVNLSGVWT-TLTNTVVDVLNVANGSQIHLN------------------------	283
	CP017552 (2e) locus BG607_11765	250	---ANGSTVNLSGVWT-TLTNTVVDVLNVANGSQIHLN------------------------	283
Genotype 2	CP017538 (2b) locus BG586_06725 and upstream	1	--	0
	CP017491 (2c) locus BG598_11445 and upstream	1	--	0
	CP017505 (2d) locus BG605_11975 and upstream	1	--	0
	CP017552 (2e) locus BG607_11765 and upstream	1	--	0
AOMB-BD-CP Genotype 2 specific*	CP017538 (2b) locus BG586_06725	1	--	0
	CP017491 (2c) locus BG598_11445	1	--	0
	CP017505 (2d) locus BG605_11975	1	--	0
	CP017552 (2e) locus BG607_11765	1	--	0

Group	Sequence	Start	Alignment	End
Peptidase S6 Genotype 1	CP017495 (1b) locus BG548_02680	701	VAEITAIFNARKVFIDLDFATGLIRENEYFEVLKVTDESAAKQQILAEKIKAAEGTVQGFNGYFGETDPSKTNGRLNITMNSNEVANGKLLITGGTNLNG	800
	CP017510 (1c) locus BG556_02675	701	VAEITAIFNARKVFIDLDFATGLIRENEYFEVLKVTDESAAKQQILAEKIKAAEGTVQGFNGYFGETDPSKTNGRLNITMNSNEVANGKLLITGGTNLNG	800
	CP017502 (1e) locus BG561_02675	701	VAEITAIFNARKVFIDLDFATGLIRENEYFEVLKVTDESAAKQQILAEKIKAAEGTVQGFNGYFGETDPSKTNGRLNITMNSNEVANGKLLITGGTNLNG	800
	CP017484 (1f) locus BG572_02680	701	VAEITAIFNARKVFIDLDFATGLIRENEYFEVLKVTDESAAKQQILAEKIKAAEGTVQGFNGYFGETDPSKTNGRLNITMNSNEVANGKLLITGGTNLNG	800
	CP017499 (1i) locus BG576_10160	701	VAEITAIFNARKVFIDLDFATGLIRENEYFEVLKVTDESAAKQQILAEKIKAAEGTVQGFNGYFGETDPSKTNGRLNITMNSNEVANGKLLITGGTNLNG	800
S6 family IgA-specific metalloendopeptidase Genotype 2	CP017538 (2b) locus BG586_06725	284	-------------------------------------TPTATKQNTLTANTLTGN------------------GIFKFVADLTKTASDIVTIKD------	322
	CP017491 (2c) locus BG598_11445	284	-------------------------------------TPTATKQNTLTANTLTGN------------------GIFKFVADLTKTASDIVTIKD------	322
	CP017505 (2d) locus BG605_11975	284	-------------------------------------TPTATKQNTLTANTLTGN------------------GIFKFVADLTKTASDIVTIKD------	322
	CP017552 (2e) locus BG607_11765	284	-------------------------------------TPTATKQNTLTANTLTGN------------------GIFKFVADLTKTASDIVTIKD------	322
OMB-BD-CP with extra upstream sequence Genotype 2 specific*	CP017538 (2b) locus BG586_06725 and upstream	1	--	0
	CP017491 (2c) locus BG598_11445 and upstream	1	--	0
	CP017505 (2d) locus BG605_11975 and upstream	1	--	0
	CP017552 (2e) locus BG607_11765 and upstream	1	--	0
AOMB-BD-CP Genotype 2 specific*	CP017538 (2b) locus BG586_06725	1	--	0
	CP017491 (2c) locus BG598_11445	1	--	0
	CP017505 (2d) locus BG605_11975	1	--	0
	CP017552 (2e) locus BG607_11765	1	--	0

Fig S4 continued

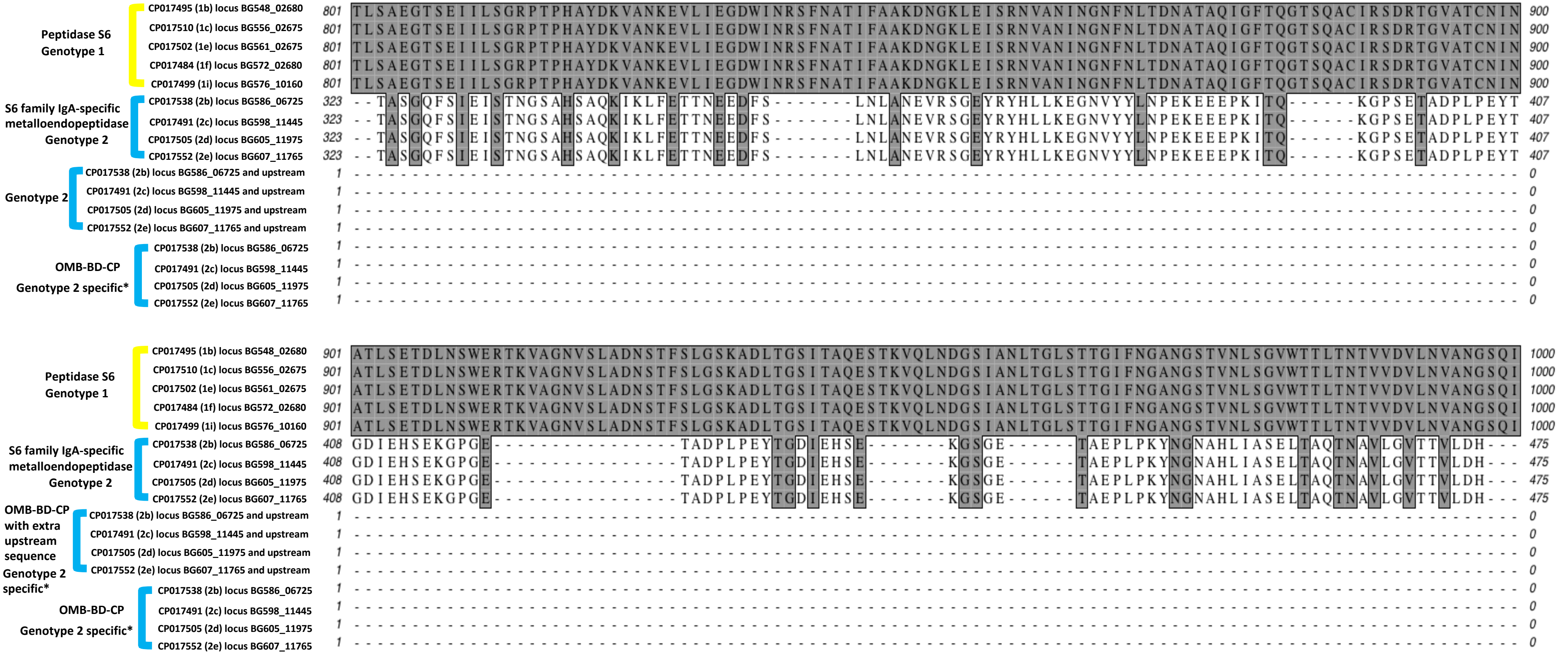

Fig S4 continued

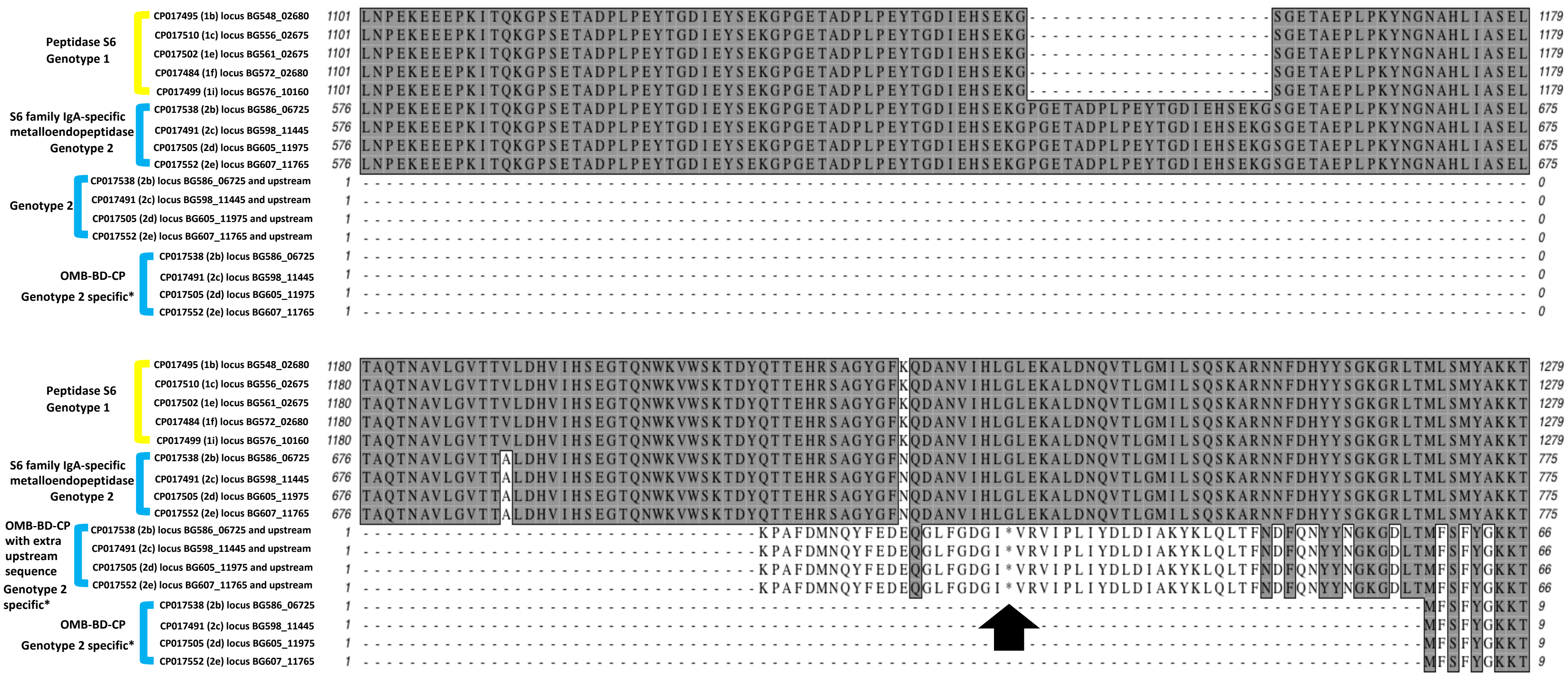

Fig S4 continued

Group	Sequence	Start	Alignment	End
Peptidase S6 Genotype 1	CP017495 (1b) locus BG548_02680	1280	WQNGVFVAIDTGFGKASNRLTYQANTVKLDRSVFVTGLSIGKAWESANVNIIPSFSARYHHLSSAGNQLVDAKIETNAVDLLALQAGLSINKTLELNGLQ	1379
	CP017510 (1c) locus BG556_02675	1280	WQNGVFVAIDTGFGKASNRLTYQANTVKLDRSVFVTGLSIGKAWESANVNIIPSFSARYHHLSSAGNQLVDAKIETNAVDLLALQAGLSINKTLELNGLQ	1379
	CP017502 (1e) locus BG561_02675	1280	WQNGVFVAIDTGFGKASNRLTYQANTVKLDRSVFVTGLSIGKAWESANVNIIPSFSARYHHLSSAGNQLVDAKIETNAVDLLALQAGLSINKTLELNGLQ	1379
	CP017484 (1f) locus BG572_02680	1280	WQNGVFVAIDTGFGKASNRLTYQANTVKLDRSVFVTGLSIGKAWESANVNIIPSFSARYHHLSSAGNQLVDAKIETNAVDLLALQAGLSINKTLELNGLQ	1379
	CP017499 (1i) locus BG576_10160	1280	WQNGVFVAIDTGFGKASNRLTYQANTVKLDRSVFVTGLSIGKAWESANVNIIPSFSARYHHLSSAGNQLVDAKIETNAVDLLALQAGLSINKTLELNGLQ	1379
S6 family IgA-specific metalloendopeptidase Genotype 2	CP017538 (2b) locus BG586_06725	776	WQNGVFVAIDTGFGKASNRLTYQANTVKLDRSVFVTGLSIGKAWESANVNIIPSFSARYHHLSSAGNQLVDAKIETNAVDLLALQAGLSINKTLELNGLQ	875
	CP017491 (2c) locus BG598_11445	776	WQNGVFVAIDTGFGKASNRLTYQANTVKLDRSVFVTGLSIGKAWESANVNIIPSFSARYHHLSSAGNQLVDAKIETNAVDLLALQAGLSINKTLELNGLQ	875
	CP017505 (2d) locus BG605_11975	776	WQNGVFVAIDTGFGKASNRLTYQANTVKLDRSVFVTGLSIGKAWESANVNIIPSFSARYHHLSSAGNQLVDAKIETNAVDLLALQAGLSINKTLELNGLQ	875
	CP017552 (2e) locus BG607_11765	776	WQNGVFVAIDTGFGKASNRLTYQANTVKLDRSVFVTGLSIGKAWESANVNIIPSFSARYHHLSSAGNQLVDAKIETNAVDLLALQAGLSINKTLELNGLQ	875
Genotype 2	CP017538 (2b) locus BG586_06725 and upstream	67	WENGIYVGIDGSIGRANNKLSHQSQTIKLARNVYSLGLTVGKTLEVASFNLQPSFGMRYHHLTSANAQLENTKFETDRVDLLAIQAGLALNKTFIFNELK	166
	CP017491 (2c) locus BG598_11445 and upstream	67	WENGIYVGIDGSIGRANNKLSHQSQTIKLARNVYSLGLTVGKTLEVASFNLQPSFGMRYHHLTSANAQLENTKFETDRVDLLAIQAGLALNKTFIFNELK	166
	CP017505 (2d) locus BG605_11975 and upstream	67	WENGIYVGIDGSIGRANNKLSHQSQTIKLARNVYSLGLTVGKTLEVASFNLQPSFGMRYHHLTSANAQLENTKFETDRVDLLAIQAGLALNKTFIFNELK	166
	CP017552 (2e) locus BG607_11765 and upstream	67	WENGIYVGIDGSIGRANNKLSHQSQTIKLARNVYSLGLTVGKTLEVASFNLQPSFGMRYHHLTSANAQLENTKFETDRVDLLAIQAGLALNKTFIFNELK	166
OMB-BD-CP Genotype 2 specific*	CP017538 (2b) locus BG586_06725	10	WENGIYVGIDGSIGRANNKLSHQSQTIKLARNVYSLGLTVGKTLEVASFNLQPSFGMRYHHLTSANAQLENTKFETDRVDLLAIQAGLALNKTFIFNELK	109
	CP017491 (2c) locus BG598_11445	10	WENGIYVGIDGSIGRANNKLSHQSQTIKLARNVYSLGLTVGKTLEVASFNLQPSFGMRYHHLTSANAQLENTKFETDRVDLLAIQAGLALNKTFIFNELK	109
	CP017505 (2d) locus BG605_11975	10	WENGIYVGIDGSIGRANNKLSHQSQTIKLARNVYSLGLTVGKTLEVASFNLQPSFGMRYHHLTSANAQLENTKFETDRVDLLAIQAGLALNKTFIFNELK	109
	CP017552 (2e) locus BG607_11765	10	WENGIYVGIDGSIGRANNKLSHQSQTIKLARNVYSLGLTVGKTLEVASFNLQPSFGMRYHHLTSANAQLENTKFETDRVDLLAIQAGLALNKTFIFNELK	109

Group	Sequence	Start	Alignment	End
Peptidase S6 Genotype 1	CP017495 (1b) locus BG548_02680	1380	IKPEIGSYFVDASHGKLRTRFNNLQIEQQMGRYFKQEAGISANYRNINAGIQAGFLTGNTLNKQRYISFKVTYEW	1454
	CP017510 (1c) locus BG556_02675	1380	IKPEIGSYFVDASHGKLRTRFNNLQIEQQMGRYFKQEAGISANYRNINAGIQAGFLTGNTLNKQRYISFKVTYEW	1454
	CP017502 (1e) locus BG561_02675	1380	IKPEIGSYFVDASHGKLRTRFNNLQIEQQMGRYFKQEAGISANYRNINAGIQAGFLTGNTLNKQRYISFKVTYEW	1454
	CP017484 (1f) locus BG572_02680	1380	IKPEIGSYFVDASHGKLRTRFNNLQIEQQMGRYFKQEAGISANYRNINAGIQAGFLTGNTLNKQRYISFKVTYEW	1454
	CP017499 (1i) locus BG576_10160	1380	IKPEIGSYFVDASHGKLRTRFNNLQIEQQMGRYFKQEAGISANYRNINAGIQAGFLTGNTLNKQRYISFKVTYEW	1454
S6 family IgA-specific metalloendopeptidase Genotype 2	CP017538 (2b) locus BG586_06725	876	IKPEIGSYFVDASHGKLRTRFNNLQIEQQMGRYFKQEAGISANYRNINAGIQAGFLTGNTLNKQRYISFKVTYEW	950
	CP017491 (2c) locus BG598_11445	876	IKPEIGSYFVDASHGKLRTRFNNLQIEQQMGRYFKQEAGISANYRNINAGIQAGFLTGNTLNKQRYISFKVTYEW	950
	CP017505 (2d) locus BG605_11975	876	IKPEIGSYFVDASHGKLRTRFNNLQIEQQMGRYFKQEAGISANYRNINAGIQAGFLTGNTLNKQRYISFKVTYEW	950
	CP017552 (2e) locus BG607_11765	876	IKPEIGSYFVDASHGKLRTRFNNLQIEQQMGRYFKQEAGISANYRNINAGIQAGFLTGNTLNKQRYISFKVTYEW	950
OMB-BD-CP with extra upstream sequence Genotype 2 specific*	CP017538 (2b) locus BG586_06725 and upstream	167	VKPELGSYFVDASHGTLKTKLNEFSLNQNIGRYFKQEVGITLHYKGVSSSVHAGFTKGNTLQEQKFISLKVGYEW	241
	CP017491 (2c) locus BG598_11445 and upstream	167	VKPELGSYFVDASHGTLKTKLNEFSLNQNIGRYFKQEVGITLHYKGVSSSVHAGFTKGNTLQEQKFISLKVGYEW	241
	CP017505 (2d) locus BG605_11975 and upstream	167	VKPELGSYFVDASHGTLKTKLNEFSLNQNIGRYFKQEVGITLHYKGVSSSVHAGFTKGNTLQEQKFISLKVGYEW	241
	CP017552 (2e) locus BG607_11765 and upstream	167	VKPELGSYFVDASHGTLKTKLNEFSLNQNIGRYFKQEVGITLHYKGVSSSVHAGFTKGNTLQEQKFISLKVGYEW	241
OMB-BD-CP Genotype 2 specific*	CP017538 (2b) locus BG586_06725	110	VKPELGSYFVDASHGTLKTKLNEFSLNQNIGRYFKQEVGITLHYKGVSSSVHAGFTKGNTLQEQKFISLKVGYEW	184
	CP017491 (2c) locus BG598_11445	110	VKPELGSYFVDASHGTLKTKLNEFSLNQNIGRYFKQEVGITLHYKGVSSSVHAGFTKGNTLQEQKFISLKVGYEW	184
	CP017505 (2d) locus BG605_11975	110	VKPELGSYFVDASHGTLKTKLNEFSLNQNIGRYFKQEVGITLHYKGVSSSVHAGFTKGNTLQEQKFISLKVGYEW	184
	CP017552 (2e) locus BG607_11765	110	VKPELGSYFVDASHGTLKTKLNEFSLNQNIGRYFKQEVGITLHYKGVSSSVHAGFTKGNTLQEQKFISLKVGYEW	184
