# Supplementary material for: Differences between predicted outer membrane proteins of genotype 1 and 2 Mannheimia haemolytica
Source: BMC Microbiol. 2020 Aug 12;20:250. doi: 10.1186/s12866-020-01932-2 (PMC7424683; doi:10.1186/s12866-020-01932-2)

Fig S5

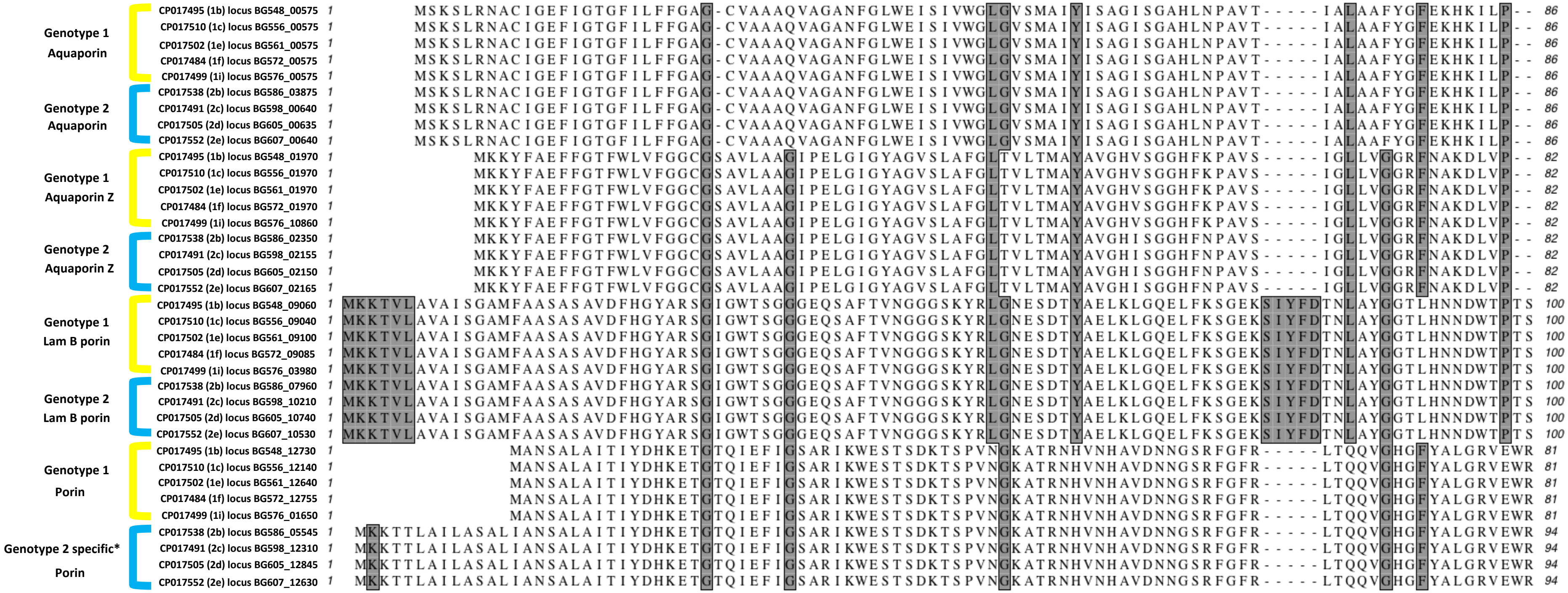
Genotype 1 Aquaporin
CP017495 (1b) locus BG548_00575 1 MSKSLRNACIGEFIGTGFILFFGAG-CVAAAQVAGANFGLWEISIVWGLGVSMAIYISAGISGAHLNPAVT-----IALAAFYGFEKHKILP-- 86
CP017510 (1c) locus BG556_00575 1 MSKSLRNACIGEFIGTGFILFFGAG-CVAAAQVAGANFGLWEISIVWGLGVSMAIYISAGISGAHLNPAVT-----IALAAFYGFEKHKILP-- 86
CP017502 (1e) locus BG561_00575 1 MSKSLRNACIGEFIGTGFILFFGAG-CVAAAQVAGANFGLWEISIVWGLGVSMAIYISAGISGAHLNPAVT-----IALAAFYGFEKHKILP-- 86
CP017484 (1f) locus BG572_00575 1 MSKSLRNACIGEFIGTGFILFFGAG-CVAAAQVAGANFGLWEISIVWGLGVSMAIYISAGISGAHLNPAVT-----IALAAFYGFEKHKILP-- 86
CP017499 (1i) locus BG576_00575 1 MSKSLRNACIGEFIGTGFILFFGAG-CVAAAQVAGANFGLWEISIVWGLGVSMAIYISAGISGAHLNPAVT-----IALAAFYGFEKHKILP-- 86
Genotype 2 Aquaporin
CP017538 (2b) locus BG586_03875 1 MSKSLRNACIGEFIGTGFILFFGAG-CVAAAQVAGANFGLWEISIVWGLGVSMAIYISAGISGAHLNPAVT-----IALAAFYGFEKHKILP-- 86
CP017491 (2c) locus BG598_00640 1 MSKSLRNACIGEFIGTGFILFFGAG-CVAAAQVAGANFGLWEISIVWGLGVSMAIYISAGISGAHLNPAVT-----IALAAFYGFEKHKILP-- 86
CP017505 (2d) locus BG605_00635 1 MSKSLRNACIGEFIGTGFILFFGAG-CVAAAQVAGANFGLWEISIVWGLGVSMAIYISAGISGAHLNPAVT-----IALAAFYGFEKHKILP-- 86
CP017552 (2e) locus BG607_00640 1 MSKSLRNACIGEFIGTGFILFFGAG-CVAAAQVAGANFGLWEISIVWGLGVSMAIYISAGISGAHLNPAVT-----IALAAFYGFEKHKILP-- 86
Genotype 1 Aquaporin Z
CP017495 (1b) locus BG548_01970 1 MKKYFAEFFGTFWLVFGGCGSAVLAAGIPELGIGYAGVSLAFGLTVLTMAYAVGHVSGGHFKPAVS-----IGLLVGGRFNAKDLVP-- 82
CP017510 (1c) locus BG556_01970 1 MKKYFAEFFGTFWLVFGGCGSAVLAAGIPELGIGYAGVSLAFGLTVLTMAYAVGHVSGGHFKPAVS-----IGLLVGGRFNAKDLVP-- 82
CP017502 (1e) locus BG561_01970 1 MKKYFAEFFGTFWLVFGGCGSAVLAAGIPELGIGYAGVSLAFGLTVLTMAYAVGHVSGGHFKPAVS-----IGLLVGGRFNAKDLVP-- 82
CP017484 (1f) locus BG572_01970 1 MKKYFAEFFGTFWLVFGGCGSAVLAAGIPELGIGYAGVSLAFGLTVLTMAYAVGHVSGGHFKPAVS-----IGLLVGGRFNAKDLVP-- 82
CP017499 (1i) locus BG576_10860 1 MKKYFAEFFGTFWLVFGGCGSAVLAAGIPELGIGYAGVSLAFGLTVLTMAYAVGHVSGGHFKPAVS-----IGLLVGGRFNAKDLVP-- 82
Genotype 2 Aquaporin Z
CP017538 (2b) locus BG586_02350 1 MKKYFAEFFGTFWLVFGGCGSAVLAAGIPELGIGYAGVSLAFGLTVLTMAYAVGHISGGHFNPAVS-----IGLLVGGRFNAKDLVP-- 82
CP017491 (2c) locus BG598_02155 1 MKKYFAEFFGTFWLVFGGCGSAVLAAGIPELGIGYAGVSLAFGLTVLTMAYAVGHISGGHFNPAVS-----IGLLVGGRFNAKDLVP-- 82
CP017505 (2d) locus BG605_02150 1 MKKYFAEFFGTFWLVFGGCGSAVLAAGIPELGIGYAGVSLAFGLTVLTMAYAVGHISGGHFNPAVS-----IGLLVGGRFNAKDLVP-- 82
CP017552 (2e) locus BG607_02165 1 MKKYFAEFFGTFWLVFGGCGSAVLAAGIPELGIGYAGVSLAFGLTVLTMAYAVGHISGGHFNPAVS-----IGLLVGGRFNAKDLVP-- 82
Genotype 1 Lam B porin
CP017495 (1b) locus BG548_09060 1 MKKTVLAVAISGAMFAASASAVDFHGYARSGIGWTSGGGEQSAFTVNGGGSKYRLGNESDTYAELKLGQELFKSGEKSIYFDTNLAYGGTLHNNDWTPTS 100
CP017510 (1c) locus BG556_09040 1 MKKTVLAVAISGAMFAASASAVDFHGYARSGIGWTSGGGEQSAFTVNGGGSKYRLGNESDTYAELKLGQELFKSGEKSIYFDTNLAYGGTLHNNDWTPTS 100
CP017502 (1e) locus BG561_09100 1 MKKTVLAVAISGAMFAASASAVDFHGYARSGIGWTSGGGEQSAFTVNGGGSKYRLGNESDTYAELKLGQELFKSGEKSIYFDTNLAYGGTLHNNDWTPTS 100
CP017484 (1f) locus BG572_09085 1 MKKTVLAVAISGAMFAASASAVDFHGYARSGIGWTSGGGEQSAFTVNGGGSKYRLGNESDTYAELKLGQELFKSGEKSIYFDTNLAYGGTLHNNDWTPTS 100
CP017499 (1i) locus BG576_03980 1 MKKTVLAVAISGAMFAASASAVDFHGYARSGIGWTSGGGEQSAFTVNGGGSKYRLGNESDTYAELKLGQELFKSGEKSIYFDTNLAYGGTLHNNDWTPTS 100
Genotype 2 Lam B porin
CP017538 (2b) locus BG586_07960 1 MKKTVLAVAISGAMFAASASAVDFHGYARSGIGWTSGGGEQSAFTVNGGGSKYRLGNESDTYAELKLGQELFKSGEKSIYFDTNLAYGGTLHNNDWTPTS 100
CP017491 (2c) locus BG598_10210 1 MKKTVLAVAISGAMFAASASAVDFHGYARSGIGWTSGGGEQSAFTVNGGGSKYRLGNESDTYAELKLGQELFKSGEKSIYFDTNLAYGGTLHNNDWTPTS 100
CP017505 (2d) locus BG605_10740 1 MKKTVLAVAISGAMFAASASAVDFHGYARSGIGWTSGGGEQSAFTVNGGGSKYRLGNESDTYAELKLGQELFKSGEKSIYFDTNLAYGGTLHNNDWTPTS 100
CP017552 (2e) locus BG607_10530 1 MKKTVLAVAISGAMFAASASAVDFHGYARSGIGWTSGGGEQSAFTVNGGGSKYRLGNESDTYAELKLGQELFKSGEKSIYFDTNLAYGGTLHNNDWTPTS 100
Genotype 1 Porin
CP017495 (1b) locus BG548_12730 1 MANSALAITIYDHKETGTQIEFIGSARIKWESTSDKTSPVNGKATRNHVNHAVDNNGSRFGFR-----LTQQVGHGFYALGRVEWR 81
CP017510 (1c) locus BG556_12140 1 MANSALAITIYDHKETGTQIEFIGSARIKWESTSDKTSPVNGKATRNHVNHAVDNNGSRFGFR-----LTQQVGHGFYALGRVEWR 81
CP017502 (1e) locus BG561_12640 1 MANSALAITIYDHKETGTQIEFIGSARIKWESTSDKTSPVNGKATRNHVNHAVDNNGSRFGFR-----LTQQVGHGFYALGRVEWR 81
CP017484 (1f) locus BG572_12755 1 MANSALAITIYDHKETGTQIEFIGSARIKWESTSDKTSPVNGKATRNHVNHAVDNNGSRFGFR-----LTQQVGHGFYALGRVEWR 81
CP017499 (1i) locus BG576_01650 1 MANSALAITIYDHKETGTQIEFIGSARIKWESTSDKTSPVNGKATRNHVNHAVDNNGSRFGFR-----LTQQVGHGFYALGRVEWR 81
Genotype 2 specific* Porin
CP017538 (2b) locus BG586_05545 1 MKKTTLAILASALIANSALAITIYDHKETGTQIEFIGSARIKWESTSDKTSPVNGKATRNHVNHAVDNNGSRFGFR-----LTQQVGHGFYALGRVEWR 94
CP017491 (2c) locus BG598_12310 1 MKKTTLAILASALIANSALAITIYDHKETGTQIEFIGSARIKWESTSDKTSPVNGKATRNHVNHAVDNNGSRFGFR-----LTQQVGHGFYALGRVEWR 94
CP017505 (2d) locus BG605_12845 1 MKKTTLAILASALIANSALAITIYDHKETGTQIEFIGSARIKWESTSDKTSPVNGKATRNHVNHAVDNNGSRFGFR-----LTQQVGHGFYALGRVEWR 94
CP017552 (2e) locus BG607_12630 1 MKKTTLAILASALIANSALAITIYDHKETGTQIEFIGSARIKWESTSDKTSPVNGKATRNHVNHAVDNNGSRFGFR-----LTQQVGHGFYALGRVEWR 94

Fig S5 continued

Group	Strain / locus	Start	Sequence	End
Genotype 1 Aquaporin	CP017495 (1b) locus BG548_00575	87	-----------YVIAQVAGAFCSVALIYFMYSDLFT------AAEAAQGITRGETVGFAG-VFSTYPNPN------ITLVTAFIVEFVITVVLMSTILAI	162
	CP017510 (1c) locus BG556_00575	87	-----------YVIAQVAGAFCSVALIYFMYSDLFT------AAEAAQGITRGETVGFAG-VFSTYPNPN------ITLVTAFIVEFVITVVLMSTILAI	162
	CP017502 (1e) locus BG561_00575	87	-----------YVIAQVAGAFCSVALIYFMYSDLFT------AAEAAQGITRGETVGFAG-VFSTYPNPN------ITLVTAFIVEFVITVVLMSTILAI	162
	CP017484 (1f) locus BG572_00575	87	-----------YVIAQVAGAFCSVALIYFMYSDLFT------AAEAAQGITRGETVGFAG-VFSTYPNPN------ITLVTAFIVEFVITVVLMSTILAI	162
	CP017499 (1i) locus BG576_00575	87	-----------YVIAQVAGAFCSVALIYFMYSDLFT------AAEAAQGITRGETVGFAG-VFSTYPNPN------ITLVTAFIVEFVITVVLMSTILAI	162
Genotype 2 Aquaporin	CP017538 (2b) locus BG586_03875	87	-----------YVIAQVAGAFCSVALIYFMYSDLFT------AAEAAQGITRGETVGFAG-VFSTYPNPN------ITLVTAFIVEFVITVVLMSTILAI	162
	CP017491 (2c) locus BG598_00640	87	-----------YVIAQVAGAFCSVALIYFMYSDLFT------AAEAAQGITRGETVGFAG-VFSTYPNPN------ITLVTAFIVEFVITVVLMSTILAI	162
	CP017505 (2d) locus BG605_00635	87	-----------YVIAQVAGAFCSVALIYFMYSDLFT------AAEAAQGITRGETVGFAG-VFSTYPNPN------ITLVTAFIVEFVITVVLMSTILAI	162
	CP017552 (2e) locus BG607_00640	87	-----------YVIAQVAGAFCSVALIYFMYSDLFT------AAEAAQGITRGETVGFAG-VFSTYPNPN------ITLVTAFIVEFVITVVLMSTILAI	162
Genotype 1 Aquaporin Z	CP017495 (1b) locus BG548_01970	83	-----------YIIAQVIGAIAAGAVLYTIASGVP---------------TFDATAGFAGNGYGEHSPHG------YSLTAALLIEVVLTAFFLIIIMGA	150
	CP017510 (1c) locus BG556_01970	83	-----------YIIAQVIGAIAAGAVLYTIASGVP---------------TFDATAGFAGNGYGEHSPHG------YSLTAALLIEVVLTAFFLIIIMGA	150
	CP017502 (1e) locus BG561_01970	83	-----------YIIAQVIGAIAAGAVLYTIASGVP---------------TFDATAGFAGNGYGEHSPHG------YSLTAALLIEVVLTAFFLIIIMGA	150
	CP017484 (1f) locus BG572_01970	83	-----------YIIAQVIGAIAAGAVLYTIASGVP---------------TFDATAGFAGNGYGEHSPHG------YSLTAALLIEVVLTAFFLIIIMGA	150
	CP017499 (1i) locus BG576_10860	83	-----------YIIAQVIGAIAAGAVLYTIASGVP---------------TFDATAGFAGNGYGEHSPHG------YSLTAALLIEVVLTAFFLIIIMGA	150
Genotype 2 Aquaporin Z	CP017538 (2b) locus BG586_02350	83	-----------YIIAQVIGAIAAGAVLYTIASGVP---------------TFDATAGFAGNGYGEHSPHG------YSLTAALLIEVVLTAFFLIIIMGA	150
	CP017491 (2c) locus BG598_02155	83	-----------YIIAQVIGAIAAGAVLYTIASGVP---------------TFDATAGFAGNGYGEHSPHG------YSLTAALLIEVVLTAFFLIIIMGA	150
	CP017505 (2d) locus BG605_02150	83	-----------YIIAQVIGAIAAGAVLYTIASGVP---------------TFDATAGFAGNGYGEHSPHG------YSLTAALLIEVVLTAFFLIIIMGA	150
	CP017552 (2e) locus BG607_02165	83	-----------YIIAQVIGAIAAGAVLYTIASGVP---------------TFDATAGFAGNGYGEHSPHG------YSLTAALLIEVVLTAFFLIIIMGA	150
Genotype 1 Lam B porin	CP017495 (1b) locus BG548_09060	101	PALRELNVQFKNFADSLPGATLWAGKRFYQRHDVHMNDFYYWDISGPGAGVENIDLGFGKLSLAVTRDTERGGANTFGYTTVYKLDPSTGKIKLDKQRNK	200
	CP017510 (1c) locus BG556_09040	101	PALRELNVQFKNFADSLPGATLWAGKRFYQRHDVHMNDFYYWDISGPGAGVENIDLGFGKLSLAVTRDTERGGANTFGYTTVYKLDPSTGKIKLDKQRNK	200
	CP017502 (1e) locus BG561_09100	101	PALRELNVQFKNFADSLPGATLWAGKRFYQRHDVHMNDFYYWDISGPGAGVENIDLGFGKLSLAVTRDTERGGANTFGYTTVYKLDPSTGKIKLDKQRNK	200
	CP017484 (1f) locus BG572_09085	101	PALRELNVQFKNFADSLPGATLWAGKRFYQRHDVHMNDFYYWDISGPGAGVENIDLGFGKLSLAVTRDTERGGANTFGYTTVYKLDPSTGKIKLDKQRNK	200
	CP017499 (1i) locus BG576_03980	101	PALRELNVQFKNFADSLPGATLWAGKRFYQRHDVHMNDFYYWDISGPGAGVENIDLGFGKLSLAVTRDTERGGANTFGYTTVYKLDPSTGKIKLDKQRNK	200
Genotype 2 Lam B porin	CP017538 (2b) locus BG586_07960	101	PALRELNVQFKNFADSLPGATLWAGKRFYQRHDVHMNDFYYWDISGPGAGVENIDLGFGKLSLAVTRDTERGGANTFGYTTVYKLDPSTGKIKLDKQRNK	200
	CP017491 (2c) locus BG598_10210	101	PALRELNVQFKNFADSLPGATLWAGKRFYQRHDVHMNDFYYWDISGPGAGVENIDLGFGKLSLAVTRDTERGGANTFGYTTVYKLDPSTGKIKLDKQRNK	200
	CP017505 (2d) locus BG605_10740	101	PALRELNVQFKNFADSLPGATLWAGKRFYQRHDVHMNDFYYWDISGPGAGVENIDLGFGKLSLAVTRDTERGGANTFGYTTVYKLDPSTGKIKLDKQRNK	200
	CP017552 (2e) locus BG607_10530	101	PALRELNVQFKNFADSLPGATLWAGKRFYQRHDVHMNDFYYWDISGPGAGVENIDLGFGKLSLAVTRDTERGGANTFGYTTVYKLDPSTGKIKLDKQRNK	200
Genotype 1 Porin	CP017495 (1b) locus BG548_12730	82	-----------MRGDSSSQHNFDHIYDHQLYAGIG--------HKQYGELTYGNMTNIADEVKQTDLPNT------LSLSDGLLTGSSRRTL*YVYKGIE	156
	CP017510 (1c) locus BG556_12140	82	-----------MRGDSSSQHNFDHIYDHQLYAGIG--------HKQYGELTYGNMTNIADEVKQTDLPNT------LSLSDGLLTGSSRRTL*YVYKGIE	156
	CP017502 (1e) locus BG561_12640	82	-----------MRGDSSSQHNFDHIYDHQLYAGIG--------HKQYGELTYGNMTNIADEVKQTDLPNT------LSLSDGLLTGSSRRTL*YVYKGIE	156
	CP017484 (1f) locus BG572_12755	82	-----------MRGDSSSQHNFDHIYDHQLYAGIG--------HKQYGELTYGNMTNIADEVKQTDLPNT------LSLSDGLLTGSSRRTL*YVYKGIE	156
	CP017499 (1i) locus BG576_01650	82	-----------MRGDSSSQHNFDHIYDHQLYAGIG--------HKQYGELTYGNMTNIADEVKQTDLPNT------LSLSDGLLTGSSRRTL*YVYKGIE	156
enotype 2 specific* Porin	CP017538 (2b) locus BG586_05545	95	-----------MRGDSSSQHNFDHIYDHQLYAGIG--------HKQYGELTYGNMTNIADEVKQTDLPNT------LSLSDGLLTGSSRRTLQYVYKGIE	169
	CP017491 (2c) locus BG598_12310	95	-----------MRGDSSSQHNFDHIYDHQLYAGIG--------HKQYGELTYGNMTNIADEVKQTDLPNT------LSLSDGLLTGSSRRTLQYVYKGIE	169
	CP017505 (2d) locus BG605_12845	95	-----------MRGDSSSQHNFDHIYDHQLYAGIG--------HKQYGELTYGNMTNIADEVKQTDLPNT------LSLSDGLLTGSSRRTLQYVYKGIE	169
	CP017552 (2e) locus BG607_12630	95	-----------MRGDSSSQHNFDHIYDHQLYAGIG--------HKQYGELTYGNMTNIADEVKQTDLPNT------LSLSDGLLTGSSRRTLQYVYKGIE	169

Fig S5 continued

Group	Sequence	Start	Alignment	End
Genotype 1 Aquaporin	CP017495 (1b) locus BG548_00575	163	GDDKNGLPNKALAALLIG----LLIAVIGGATGPLTGFAMNP-ARDFGPKLFAYLAGWGEIALTGGKEIPYFIIPIVAPICGALFG--------------	243
	CP017510 (1c) locus BG556_00575	163	GDDKNGLPNKALAALLIG----LLIAVIGGATGPLTGFAMNP-ARDFGPKLFAYLAGWGEIALTGGKEIPYFIIPIVAPICGALFG--------------	243
	CP017502 (1e) locus BG561_00575	163	GDDKNGLPNKALAALLIG----LLIAVIGGATGPLTGFAMNP-ARDFGPKLFAYLAGWGEIALTGGKEIPYFIIPIVAPICGALFG--------------	243
	CP017484 (1f) locus BG572_00575	163	GDDKNGLPNKALAALLIG----LLIAVIGGATGPLTGFAMNP-ARDFGPKLFAYLAGWGEIALTGGKEIPYFIIPIVAPICGALFG--------------	243
	CP017499 (1i) locus BG576_00575	163	GDDKNGLPNKALAALLIG----LLIAVIGGATGPLTGFAMNP-ARDFGPKLFAYLAGWGEIALTGGKEIPYFIIPIVAPICGALFG--------------	243
Genotype 2 Aquaporin	CP017538 (2b) locus BG586_03875	163	GDDKNGLPNKALAALLIG----LLIAVIGGATGPLTGFAMNP-ARDFGPKLFAYLAGWGEIALTGGKEIPYFIIPIVAPICGALFG--------------	243
	CP017491 (2c) locus BG598_00640	163	GDDKNGLPNKALAALLIG----LLIAVIGGATGPLTGFAMNP-ARDFGPKLFAYLAGWGEIALTGGKEIPYFIIPIVAPICGALFG--------------	243
	CP017505 (2d) locus BG605_00635	163	GDDKNGLPNKALAALLIG----LLIAVIGGATGPLTGFAMNP-ARDFGPKLFAYLAGWGEIALTGGKEIPYFIIPIVAPICGALFG--------------	243
	CP017552 (2e) locus BG607_00640	163	GDDKNGLPNKALAALLIG----LLIAVIGGATGPLTGFAMNP-ARDFGPKLFAYLAGWGEIALTGGKEIPYFIIPIVAPICGALFG--------------	243
Genotype 1 Aquaporin Z	CP017495 (1b) locus BG548_01970	151	TDKR---APAGFAPIAIG----LALTLIHLISIPVTNTSVNP-ARSTGVALFQ-------------GSWAIEQLWLFWVAPIVGAIIG--------------	217
	CP017510 (1c) locus BG556_01970	151	TDKR---APAGFAPIAIG----LALTLIHLISIPVTNTSVNP-ARSTGVALFQ-------------GSWAIEQLWLFWVAPIVGAIIG--------------	217
	CP017502 (1e) locus BG561_01970	151	TDKR---APAGFAPIAIG----LALTLIHLISIPVTNTSVNP-ARSTGVALFQ-------------GSWAIEQLWLFWVAPIVGAIIG--------------	217
	CP017484 (1f) locus BG572_01970	151	TDKR---APAGFAPIAIG----LALTLIHLISIPVTNTSVNP-ARSTGVALFQ-------------GSWAIEQLWLFWVAPIVGAIIG--------------	217
	CP017499 (1i) locus BG576_10860	151	TDKR---APAGFAPIAIG----LALTLIHLISIPVTNTSVNP-ARSTGVALFQ-------------GSWAIEQLWLFWVAPIVGAIIG--------------	217
Genotype 2 Aquaporin Z	CP017538 (2b) locus BG586_02350	151	TDKR---APAGFAPIAIG----LALTLIHLISIPVTNTSVNP-ARSTGVALFQ-------------GSWAIEQLWLFWVAPIVGAIIG--------------	217
	CP017491 (2c) locus BG598_02155	151	TDKR---APAGFAPIAIG----LALTLIHLISIPVTNTSVNP-ARSTGVALFQ-------------GSWAIEQLWLFWVAPIVGAIIG--------------	217
	CP017505 (2d) locus BG605_02150	151	TDKR---APAGFAPIAIG----LALTLIHLISIPVTNTSVNP-ARSTGVALFQ-------------GSWAIEQLWLFWVAPIVGAIIG--------------	217
	CP017552 (2e) locus BG607_02165	151	TDKR---APAGFAPIAIG----LALTLIHLISIPVTNTSVNP-ARSTGVALFQ-------------GSWAIEQLWLFWVAPIVGAIIG--------------	217
Genotype 1 Lam B porin	CP017495 (1b) locus BG548_09060	201	ADVYNDIFDVRLAGIELWKDGSLELGFDYGNAHAKKGAALEKGATKNGYMVTAEYTQGNFFGGFNKFTAQYATDSMTSWNNGHAQGSKADNKGNMLRLIN	300
	CP017510 (1c) locus BG556_09040	201	ADVYNDIFDVRLAGIELWKDGSLELGFDYGNAHAKKGAALEKGATKNGYMVTAEYTQGNFFGGFNKFTAQYATDSMTSWNNGHAQGSKADNKGNMLRLIN	300
	CP017502 (1e) locus BG561_09100	201	ADVYNDIFDVRLAGIELWKDGSLELGFDYGNAHAKKGAALEKGATKNGYMVTAEYTQGNFFGGFNKFTAQYATDSMTSWNNGHAQGSKADNKGNMLRLIN	300
	CP017484 (1f) locus BG572_09085	201	ADVYNDIFDVRLAGIELWKDGSLELGFDYGNAHAKKGAALEKGATKNGYMVTAEYTQGNFFGGFNKFTAQYATDSMTSWNNGHAQGSKADNKGNMLRLIN	300
	CP017499 (1i) locus BG576_03980	201	ADVYNDIFDVRLAGIELWKDGSLELGFDYGNAHAKKGAALEKGATKNGYMVTAEYTQGNFFGGFNKFTAQYATDSMTSWNNGHAQGSKADNKGNMLRLIN	300
Genotype 2 Lam B porin	CP017538 (2b) locus BG586_07960	201	ADVYNDIFDVRLAGIELWKDGSLELGFDYGNAHAKKGAALEKGATKNGYMVTAEYTQGNFFGGFNKFTAQYATDSMTSWNNGHAQGSKADNKGNMLRLIN	300
	CP017491 (2c) locus BG598_10210	201	ADVYNDIFDVRLAGIELWKDGSLELGFDYGNAHAKKGAALEKGATKNGYMVTAEYTQGNFFGGFNKFTAQYATDSMTSWNNGHAQGSKADNKGNMLRLIN	300
	CP017505 (2d) locus BG605_10740	201	ADVYNDIFDVRLAGIELWKDGSLELGFDYGNAHAKKGAALEKGATKNGYMVTAEYTQGNFFGGFNKFTAQYATDSMTSWNNGHAQGSKADNKGNMLRLIN	300
	CP017552 (2e) locus BG607_10530	201	ADVYNDIFDVRLAGIELWKDGSLELGFDYGNAHAKKGAALEKGATKNGYMVTAEYTQGNFFGGFNKFTAQYATDSMTSWNNGHAQGSKADNKGNMLRLIN	300
Genotype 1 Porin	CP017495 (1b) locus BG548_12730	157	GLKLGAFYGGNSQRGNNG----LDLANKRKDIWGAAAIYNYKIDDNQSLKLATGATRERSEQSIGNYERTALAFGTAYTYGKTTLG--------------	238
	CP017510 (1c) locus BG556_12140	157	GLKLGAFYGGNSQRGNNG----LDLANKRKDIWGAAAIYNYKIDDNQSLKLATGATRERSEQSIGNYERTALAFGTAYTYGKTTLG--------------	238
	CP017502 (1e) locus BG561_12640	157	GLKLGAFYGGNSQRGNNG----LDLANKRKDIWGAAAIYNYKIDDNQSLKLATGATRERSEQSIGNYERTALAFGTAYTYGKTTLG--------------	238
	CP017484 (1f) locus BG572_12755	157	GLKLGAFYGGNSQRGNNG----LDLANKRKDIWGAAAIYNYKIDDNQSLKLATGATRERSEQSIGNYERTALAFGTAYTYGKTTLG--------------	238
	CP017499 (1i) locus BG576_01650	157	GLKLGAFYGGNSQRGNNG----LDLANKRKDIWGAAAIYNYKIDDNQSLKLATGATRERSEQSIGNYERTALAFGTAYTYGKTTLG--------------	238
[illegible]enotype 2 specific* Porin	CP017538 (2b) locus BG586_05545	170	GLKLGAFYGGNSQRGNNG----LDLVNKRKDIWGAAAIYNYKIDDNQSLKLATGATRERSEQSIGNYERIALAFGTAYTYGKTTLG--------------	251
	CP017491 (2c) locus BG598_12310	170	GLKLGAFYGGNSQRGNNG----LDLVNKRKDIWGAAAIYNYKIDDNQSLKLATGATRERSEQSIGNYERIALAFGTAYTYGKTTLG--------------	251
	CP017505 (2d) locus BG605_12845	170	GLKLGAFYGGNSQRGNNG----LDLVNKRKDIWGAAAIYNYKIDDNQSLKLATGATRERSEQSIGNYERIALAFGTAYTYGKTTLG--------------	251
	CP017552 (2e) locus BG607_12630	170	GLKLGAFYGGNSQRGNNG----LDLVNKRKDIWGAAAIYNYKIDDNQSLKLATGATRERSEQSIGNYERIALAFGTAYTYGKTTLG--------------	251

Fig S5 continued

Group	Sequence	Start	Alignment	End
Genotype 1 Aquaporin	CP017495 (1b) locus BG548_00575	244	----------AWGYKNLIHNNLPANTQE--	261
	CP017510 (1c) locus BG556_00575	244	----------AWGYKNLIHNNLPANTQE--	261
	CP017502 (1e) locus BG561_00575	244	----------AWGYKNLIHNNLPANTQE--	261
	CP017484 (1f) locus BG572_00575	244	----------AWGYKNLIHNNLPANTQE--	261
	CP017499 (1i) locus BG576_00575	244	----------AWGYKNLIHNNLPANTQE--	261
Genotype 2 Aquaporin	CP017538 (2b) locus BG586_03875	244	----------AWGYKNLIHNNLPANTQE--	261
	CP017491 (2c) locus BG598_00640	244	----------AWGYKNLIHNNLPANTQE--	261
	CP017505 (2d) locus BG605_00635	244	----------AWGYKNLIHNNLPANTQE--	261
	CP017552 (2e) locus BG607_00640	244	----------AWGYKNLIHNNLPANTQE--	261
Genotype 1 Aquaporin Z	CP017495 (1b) locus BG548_01970	218	----------AVVYRFIAEEK---	228
	CP017510 (1c) locus BG556_01970	218	----------AVVYRFIAEEK---	228
	CP017502 (1e) locus BG561_01970	218	----------AVVYRFIAEEK---	228
	CP017484 (1f) locus BG572_01970	218	----------AVVYRFIAEEK---	228
	CP017499 (1i) locus BG576_10860	218	----------AVVYRFIAEEK---	228
Genotype 2 Aquaporin Z	CP017538 (2b) locus BG586_02350	218	----------AVVYRFIAEEK---	228
	CP017491 (2c) locus BG598_02155	218	----------AVVYRFIAEEK---	228
	CP017505 (2d) locus BG605_02150	218	----------AVVYRFIAEEK---	228
	CP017552 (2e) locus BG607_02165	218	----------AVVYRFIAEEK---	228
Genotype 1 Lam B porin	CP017495 (1b) locus BG548_09060	301	QGVVQASDKVEVMYALIYEKTKLDNKQGKTWYSAGIRPMYKWNDTMSTLLEVGYDRIKDQATGKKNDLVKYTVAQQWQAGNSIWARPAIRVFGTYARWND	400
	CP017510 (1c) locus BG556_09040	301	QGVVQASDKVEVMYALIYEKTKLDNKQGKTWYSAGIRPMYKWNDTMSTLLEVGYDRIKDQATGKKNDLVKYTVAQQWQAGNSIWARPAIRVFGTYARWND	400
	CP017502 (1e) locus BG561_09100	301	QGVVQASDKVEVMYALIYEKTKLDNKQGKTWYSAGIRPMYKWNDTMSTLLEVGYDRIKDQATGKKNDLVKYTVAQQWQAGNSIWARPAIRVFGTYARWND	400
	CP017484 (1f) locus BG572_09085	301	QGVVQASDKVEVMYALIYEKTKLDNKQGKTWYSAGIRPMYKWNDTMSTLLEVGYDRIKDQATGKKNDLVKYTVAQQWQAGNSIWARPAIRVFGTYARWND	400
	CP017499 (1i) locus BG576_03980	301	QGVVQASDKVEVMYALIYEKTKLDNKQGKTWYSAGIRPMYKWNDTMSTLLEVGYDRIKDQATGKKNDLVKYTVAQQWQAGNSIWARPAIRVFGTYARWND	400
Genotype 2 Lam B porin	CP017538 (2b) locus BG586_07960	301	QGVVQASDKVEVMYALIYEKTKLDNKQGKTWYSAGIRPMYKWNDTMSTLLEVGYDRIKDQATGKKNDLVKYTVAQQWQAGNSIWARPAIRVFGTYARWND	400
	CP017491 (2c) locus BG598_10210	301	QGVVQASDKVEVMYALIYEKTKLDNKQGKTWYSAGIRPMYKWNDTMSTLLEVGYDRIKDQATGKKNDLVKYTVAQQWQAGNSIWARPAIRVFGTYARWND	400
	CP017505 (2d) locus BG605_10740	301	QGVVQASDKVEVMYALIYEKTKLDNKQGKTWYSAGIRPMYKWNDTMSTLLEVGYDRIKDQATGKKNDLVKYTVAQQWQAGNSIWARPAIRVFGTYARWND	400
	CP017552 (2e) locus BG607_10530	301	QGVVQASDKVEVMYALIYEKTKLDNKQGKTWYSAGIRPMYKWNDTMSTLLEVGYDRIKDQATGKKNDLVKYTVAQQWQAGNSIWARPAIRVFGTYARWND	400
Genotype 1 Porin	CP017495 (1b) locus BG548_12730	239	---------LDLERRETEDQNAIGHKRIEKEVRT--VLLHRLTDQWNAYTMYAYKENKLNAVGLANDSKVKRNQFMLGTEYYIVPKHLKGFVEWQATRAK	327
	CP017510 (1c) locus BG556_12140	239	---------LDLERRETEDQNAIGHKRIEKEVRT--VLLHRLTDQWNAYTMYAYKENKLNAVGLANDSKVKRNQFMLGTEYYIVPKHLKGFVEWQATRAK	327
	CP017502 (1e) locus BG561_12640	239	---------LDLERRETEDQNAIGHKRIEKEVRT--VLLHRLTDQWNAYTMYAYKENKLNAVGLANDSKVKRNQFMLGTEYYIVPKHLKGFVEWQATRAK	327
	CP017484 (1f) locus BG572_12755	239	---------LDLERRETEDQNAIGHKRIEKEVRT--VLLHRLTDQWNAYTMYAYKENKLNAVGLANDSKVKRNQFMLGTEYYIVPKHLKGFVEWQATRAK	327
	CP017499 (1i) locus BG576_01650	239	---------LDLERRETEDQNAIGHKRIEKEVRT--VLLHRLTDQWNAYTMYAYKENKLNAVGLANDSKVKRNQFMLGTEYYIVPKHLKGFVEWQATRAK	327
Genotype 2 specific* Porin	CP017538 (2b) locus BG586_05545	252	---------LDLERRETEDQNAIGHKRIEKEVRT--VLLHRLTDQWNAYTMYAYKENKLNAVGLANDSKVKRNQFMLGTEYYIVPKHLKGFVEWQATRAK	340
	CP017491 (2c) locus BG598_12310	252	---------LDLERRETEDQNAIGHKRIEKEVRT--VLLHRLTDQWNAYTMYAYKENKLNAVGLANDSKVKRNQFMLGTEYYIVPKHLKGFVEWQATRAK	340
	CP017505 (2d) locus BG605_12845	252	---------LDLERRETEDQNAIGHKRIEKEVRT--VLLHRLTDQWNAYTMYAYKENKLNAVGLANDSKVKRNQFMLGTEYYIVPKHLKGFVEWQATRAK	340
	CP017552 (2e) locus BG607_12630	252	---------LDLERRETEDQNAIGHKRIEKEVRT--VLLHRLTDQWNAYTMYAYKENKLNAVGLANDSKVKRNQFMLGTEYYIVPKHLKGFVEWQATRAK	340

Fig S5 continued

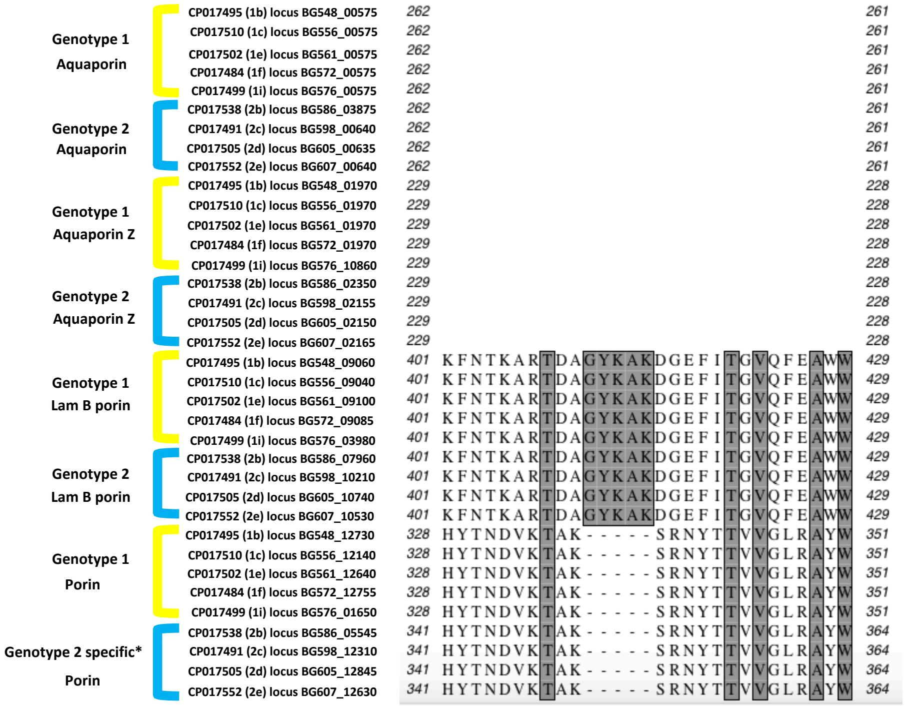

Supplement: Supplementary file 10 — Additional file 10: Figure S5. Alignment of all annotated porin-like proteins in five genotype 1 and four genotype 2 M. haemolytica strains that are each of a different subtype. The proteins flagged by EDGAR as specific to genotype 2 M. haemolytica are indicated within the alignment with an asterisk. The site of premature stop codons within proteins encoded by a pseudogene homolog of the genotype 2 specific porin is highlighted with an arrow. Areas of 51% chemical identity or greater are indicated with grey boxes. [file 12866_2020_1932_MOESM10_ESM.pdf]
