# Supplementary material for: Differences between predicted outer membrane proteins of genotype 1 and 2 Mannheimia haemolytica
Source: BMC Microbiol. 2020 Aug 12;20:250. doi: 10.1186/s12866-020-01932-2 (PMC7424683; doi:10.1186/s12866-020-01932-2)
Supplement: Supplementary file 11 — Additional file 11: Figure S6. Alignment of ligand-gated channel proteins in five genotype 1 and four genotype 2 M. haemolytica strains that are each of a different subtype. Within the alignment, proteins encoded by the ligand-gated channel gene flagged by EDGAR as specific to genotype 2 M. haemolytica are labelled with an asterisk. The stop codon site within the genotype 1 proteins that are encoded by a pseudogene is highlighted with an arrow. Areas of 51% chemical identity or greater are indicated with grey boxes within the alignment. [file 12866_2020_1932_MOESM11_ESM.pdf]

Fig S6

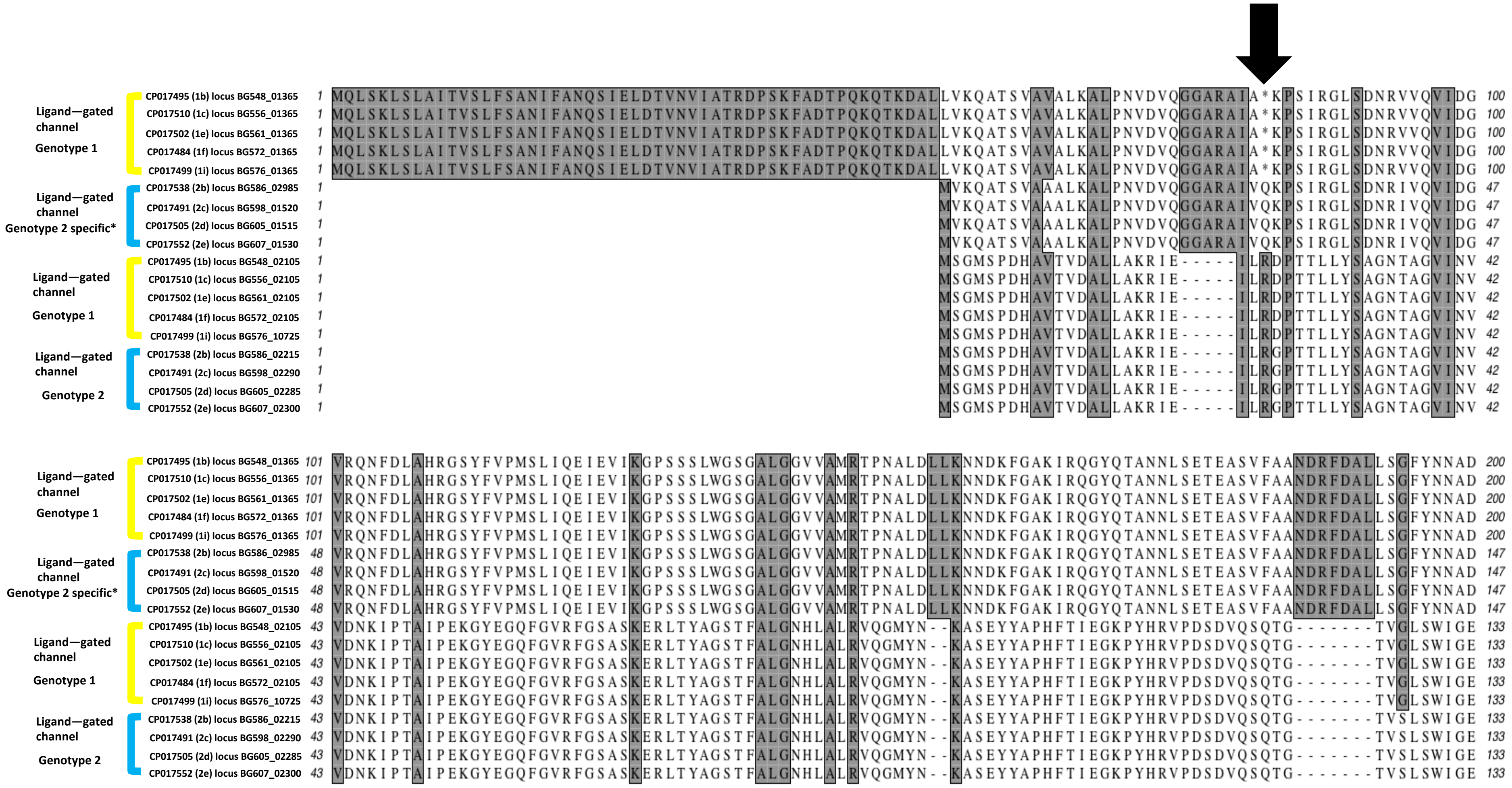

Ligand—gated channel Genotype 1
CP017495 (1b) locus BG548_01365 1 MQLSKLSLAITVSLFSANIFANQSIELDTVNVIATRDPSKFADTPQKQTKDALLVKQATSVAVALKALPNVDVQGGARAIA*KPSIRGLSDNRVVQVIDG 100
CP017510 (1c) locus BG556_01365 1 MQLSKLSLAITVSLFSANIFANQSIELDTVNVIATRDPSKFADTPQKQTKDALLVKQATSVAVALKALPNVDVQGGARAIA*KPSIRGLSDNRVVQVIDG 100
CP017502 (1e) locus BG561_01365 1 MQLSKLSLAITVSLFSANIFANQSIELDTVNVIATRDPSKFADTPQKQTKDALLVKQATSVAVALKALPNVDVQGGARAIA*KPSIRGLSDNRVVQVIDG 100
CP017484 (1f) locus BG572_01365 1 MQLSKLSLAITVSLFSANIFANQSIELDTVNVIATRDPSKFADTPQKQTKDALLVKQATSVAVALKALPNVDVQGGARAIA*KPSIRGLSDNRVVQVIDG 100
CP017499 (1i) locus BG576_01365 1 MQLSKLSLAITVSLFSANIFANQSIELDTVNVIATRDPSKFADTPQKQTKDALLVKQATSVAVALKALPNVDVQGGARAIA*KPSIRGLSDNRVVQVIDG 100
Ligand—gated channel Genotype 2 specific*
CP017538 (2b) locus BG586_02985 1 MVKQATSVAAALKALPNVDVQGGARAIVQKPSIRGLSDNRIVQVIDG 47
CP017491 (2c) locus BG598_01520 1 MVKQATSVAAALKALPNVDVQGGARAIVQKPSIRGLSDNRIVQVIDG 47
CP017505 (2d) locus BG605_01515 1 MVKQATSVAAALKALPNVDVQGGARAIVQKPSIRGLSDNRIVQVIDG 47
CP017552 (2e) locus BG607_01530 1 MVKQATSVAAALKALPNVDVQGGARAIVQKPSIRGLSDNRIVQVIDG 47
Ligand—gated channel Genotype 1
CP017495 (1b) locus BG548_02105 1 MSGMSPDHAVTVDALLAKRIE-----ILRDPTTLLYSAGNTAGVINV 42
CP017510 (1c) locus BG556_02105 1 MSGMSPDHAVTVDALLAKRIE-----ILRDPTTLLYSAGNTAGVINV 42
CP017502 (1e) locus BG561_02105 1 MSGMSPDHAVTVDALLAKRIE-----ILRDPTTLLYSAGNTAGVINV 42
CP017484 (1f) locus BG572_02105 1 MSGMSPDHAVTVDALLAKRIE-----ILRDPTTLLYSAGNTAGVINV 42
CP017499 (1i) locus BG576_10725 1 MSGMSPDHAVTVDALLAKRIE-----ILRDPTTLLYSAGNTAGVINV 42
Ligand—gated channel Genotype 2
CP017538 (2b) locus BG586_02215 1 MSGMSPDHAVTVDALLAKRIE-----ILRGPTTLLYSAGNTAGVINV 42
CP017491 (2c) locus BG598_02290 1 MSGMSPDHAVTVDALLAKRIE-----ILRGPTTLLYSAGNTAGVINV 42
CP017505 (2d) locus BG605_02285 1 MSGMSPDHAVTVDALLAKRIE-----ILRGPTTLLYSAGNTAGVINV 42
CP017552 (2e) locus BG607_02300 1 MSGMSPDHAVTVDALLAKRIE-----ILRGPTTLLYSAGNTAGVINV 42
Ligand—gated channel Genotype 1
CP017495 (1b) locus BG548_01365 101 VRQNFDLAHRGSYFVPMSLIQEIEVIKGPSSSLWGSGALGGVVAMRTPNALDLLKNNDKFGAKIRQGYQTANNLSETEASVFAANDRFDALLSGFYNNAD 200
CP017510 (1c) locus BG556_01365 101 VRQNFDLAHRGSYFVPMSLIQEIEVIKGPSSSLWGSGALGGVVAMRTPNALDLLKNNDKFGAKIRQGYQTANNLSETEASVFAANDRFDALLSGFYNNAD 200
CP017502 (1e) locus BG561_01365 101 VRQNFDLAHRGSYFVPMSLIQEIEVIKGPSSSLWGSGALGGVVAMRTPNALDLLKNNDKFGAKIRQGYQTANNLSETEASVFAANDRFDALLSGFYNNAD 200
CP017484 (1f) locus BG572_01365 101 VRQNFDLAHRGSYFVPMSLIQEIEVIKGPSSSLWGSGALGGVVAMRTPNALDLLKNNDKFGAKIRQGYQTANNLSETEASVFAANDRFDALLSGFYNNAD 200
CP017499 (1i) locus BG576_01365 101 VRQNFDLAHRGSYFVPMSLIQEIEVIKGPSSSLWGSGALGGVVAMRTPNALDLLKNNDKFGAKIRQGYQTANNLSETEASVFAANDRFDALLSGFYNNAD 200
Ligand—gated channel Genotype 2 specific*
CP017538 (2b) locus BG586_02985 48 VRQNFDLAHRGSYFVPMSLIQEIEVIKGPSSSLWGSGALGGVVAMRTPNALDLLKNNDKFGAKIRQGYQTANNLSETEASVFAANDRFDALLSGFYNNAD 147
CP017491 (2c) locus BG598_01520 48 VRQNFDLAHRGSYFVPMSLIQEIEVIKGPSSSLWGSGALGGVVAMRTPNALDLLKNNDKFGAKIRQGYQTANNLSETEASVFAANDRFDALLSGFYNNAD 147
CP017505 (2d) locus BG605_01515 48 VRQNFDLAHRGSYFVPMSLIQEIEVIKGPSSSLWGSGALGGVVAMRTPNALDLLKNNDKFGAKIRQGYQTANNLSETEASVFAANDRFDALLSGFYNNAD 147
CP017552 (2e) locus BG607_01530 48 VRQNFDLAHRGSYFVPMSLIQEIEVIKGPSSSLWGSGALGGVVAMRTPNALDLLKNNDKFGAKIRQGYQTANNLSETEASVFAANDRFDALLSGFYNNAD 147
Ligand—gated channel Genotype 1
CP017495 (1b) locus BG548_02105 43 VDNKIPTAIPEKGYEGQFGVRFGSASKERLTYAGSTFALGNHLALRVQGMYN--KASEYYAPHFTIEGKPYHRVPDSDVQSQTG-------TVGLSWIGE 133
CP017510 (1c) locus BG556_02105 43 VDNKIPTAIPEKGYEGQFGVRFGSASKERLTYAGSTFALGNHLALRVQGMYN--KASEYYAPHFTIEGKPYHRVPDSDVQSQTG-------TVGLSWIGE 133
CP017502 (1e) locus BG561_02105 43 VDNKIPTAIPEKGYEGQFGVRFGSASKERLTYAGSTFALGNHLALRVQGMYN--KASEYYAPHFTIEGKPYHRVPDSDVQSQTG-------TVGLSWIGE 133
CP017484 (1f) locus BG572_02105 43 VDNKIPTAIPEKGYEGQFGVRFGSASKERLTYAGSTFALGNHLALRVQGMYN--KASEYYAPHFTIEGKPYHRVPDSDVQSQTG-------TVGLSWIGE 133
CP017499 (1i) locus BG576_10725 43 VDNKIPTAIPEKGYEGQFGVRFGSASKERLTYAGSTFALGNHLALRVQGMYN--KASEYYAPHFTIEGKPYHRVPDSDVQSQTG-------TVGLSWIGE 133
Ligand—gated channel Genotype 2
CP017538 (2b) locus BG586_02215 43 VDNKIPTAIPEKGYEGQFGVRFGSASKERLTYAGSTFALGNHLALRVQGMYN--KASEYYAPHFTIEGKPYHRVPDSDVQSQTG-------TVSLSWIGE 133
CP017491 (2c) locus BG598_02290 43 VDNKIPTAIPEKGYEGQFGVRFGSASKERLTYAGSTFALGNHLALRVQGMYN--KASEYYAPHFTIEGKPYHRVPDSDVQSQTG-------TVSLSWIGE 133
CP017505 (2d) locus BG605_02285 43 VDNKIPTAIPEKGYEGQFGVRFGSASKERLTYAGSTFALGNHLALRVQGMYN--KASEYYAPHFTIEGKPYHRVPDSDVQSQTG-------TVSLSWIGE 133
CP017552 (2e) locus BG607_02300 43 VDNKIPTAIPEKGYEGQFGVRFGSASKERLTYAGSTFALGNHLALRVQGMYN--KASEYYAPHFTIEGKPYHRVPDSDVQSQTG-------TVSLSWIGE 133

Fig S6 continued

Group	Sequence	Start	Alignment	End
Ligand—gated channel Genotype 1	CP017495 (1b) locus BG548_01365	201	NLRSGEGRELNNTGYKQAGGLVKFGWQLNEANRVELSHRESQFKQTAPSNNDVENQFTTADINAKIAAWHAANP--PSTPNFFAKLAAFYGGLGAQFGSV	298
	CP017510 (1c) locus BG556_01365	201	NLRSGEGRELNNTGYKQAGGLVKFGWQLNEANRVELSHRESQFKQTAPSNNDVENQFTTADINAKIAAWHAANP--PSTPNFFAKLAAFYGGLGAQFGSV	298
	CP017502 (1e) locus BG561_01365	201	NLRSGEGRELNNTGYKQAGGLVKFGWQLNEANRVELSHRESQFKQTAPSNNDVENQFTTADINAKIAAWHAANP--PSTPNFFAKLAAFYGGLGAQFGSV	298
	CP017484 (1f) locus BG572_01365	201	NLRSGEGRELNNTGYKQAGGLVKFGWQLNEANRVELSHRESQFKQTAPSNNDVENQFTTADINAKIAAWHAANP--PSTPNFFAKLAAFYGGLGAQFGSV	298
	CP017499 (1i) locus BG576_01365	201	NLRSGEGRELNNTGYKQAGGLVKFGWQLNEANRVELSHRESQFKQTAPSNNDVENQFTTADINAKIAAWHAANP--PSTPNFFAKLAAFYGGLGAQFGSV	298
Ligand—gated channel Genotype 2 specific*	CP017538 (2b) locus BG586_02985	148	NLRSGEGKELNNTGYKQAGGLVKFGWQINDTHRLELSHRHSQFKQTAPGNNEVENQFTDNDVSAQISAWHAANPGNPSNPVYLAKMLAFYGQLHSSLGSV	247
	CP017491 (2c) locus BG598_01520	148	NLRSGEGKELNNTGYKQAGGLVKFGWQINDTHRLELSHRHSQFKQTAPGNNEVENQFTDNDVSAQISAWHAANPGNPSNPVYLAKMLAFYGQLHSSLGSV	247
	CP017505 (2d) locus BG605_01515	148	NLRSGEGKELNNTGYKQAGGLVKFGWQINDTHRLELSHRHSQFKQTAPGNNEVENQFTDNDVSAQISAWHAANPGNPSNPVYLAKMLAFYGQLHSSLGSV	247
	CP017552 (2e) locus BG607_01530	148	NLRSGEGKELNNTGYKQAGGLVKFGWQINDTHRLELSHRHSQFKQTAPGNNEVENQFTDNDVSAQISAWHAANPGNPSNPVYLAKMLAFYGQLHSSLGSV	247
Ligand—gated channel Genotype 1	CP017495 (1b) locus BG548_02105	134	RGHLGIAYTDRRDKYGLIGHTHKYDHYTISIIRQAVMFAKGYLRFYPHLAEEGDIDYNNPGIRLLHTHIPGGSHYGQDTHEHGKPWIDMHSKRYDIDGSL	233
	CP017510 (1c) locus BG556_02105	134	RGHLGIAYTDRRDKYGLIGHTHKYDHYTISIIRQAVMFAKGYLRFYPHLAEEGDIDYNNPGIRLLHTHIPGGSHYGQDTHEHGKPWIDMHSKRYDIDGSL	233
	CP017502 (1e) locus BG561_02105	134	RGHLGIAYTDRRDKYGLIGHTHKYDHYTISIIRQAVMFAKGYLRFYPHLAEEGDIDYNNPGIRLLHTHIPGGSHYGQDTHEHGKPWIDMHSKRYDIDGSL	233
	CP017484 (1f) locus BG572_02105	134	RGHLGIAYTDRRDKYGLIGHTHKYDHYTISIIRQAVMFAKGYLRFYPHLAEEGDIDYNNPGIRLLHTHIPGGSHYGQDTHEHGKPWIDMHSKRYDIDGSL	233
	CP017499 (1i) locus BG576_10725	134	RGHLGIAYTDRRDKYGLIGHTHKYDHYTISIIRQAVMFAKGYLRFYPHLAEEGDIDYNNPGIRLLHTHIPGGSHYGQDTHEHGKPWIDMHSKRYDIDGSL	233
Ligand—gated channel Genotype 2	CP017538 (2b) locus BG586_02215	134	RGHLGIAYTDRRDKYGLIGHTHKYDHYTISIIRQAVMFAKGYLRFYPHLAEEGDIDYNNPGIRLLHTHIPGGSHYGQDTHEHGKPWIDMHSKRYDIDGSL	233
	CP017491 (2c) locus BG598_02290	134	RGHLGIAYTDRRDKYGLIGHTHKYDHYTISIIRQAVMFAKGYLRFYPHLAEEGDIDYNNPGIRLLHTHIPGGSHYGQDTHEHGKPWIDMHSKRYDIDGSL	233
	CP017505 (2d) locus BG605_02285	134	RGHLGIAYTDRRDKYGLIGHTHKYDHYTISIIRQAVMFAKGYLRFYPHLAEEGDIDYNNPGIRLLHTHIPGGSHYGQDTHEHGKPWIDMHSKRYDIDGSL	233
	CP017552 (2e) locus BG607_02300	134	RGHLGIAYTDRRDKYGLIGHTHKYDHYTISIIRQAVMFAKGYLRFYPHLAEEGDIDYNNPGIRLLHTHIPGGSHYGQDTHEHGKPWIDMHSKRYDIDGSL	233

Group	Sequence	Start	Alignment	End
Ligand—gated channel Genotype 1	CP017495 (1b) locus BG548_01365	299	SYLSDQKINDKSTALNYYFNPSNPYVNAQVTVYQNSTTEKEQRKVSGVEDRTKLTTRGINLRNHSELPWFSLVYGVDYMQDKADTERGTN----NSDARY	394
	CP017510 (1c) locus BG556_01365	299	SYLSDQKINDKSTALNYYFNPSNPYVNAQVTVYQNSTTEKEQRKVSGVEDRTKLTTRGINLRNHSELPWFSLVYGVDYMQDKADTERGTN----NSDARY	394
	CP017502 (1e) locus BG561_01365	299	SYLSDQKINDKSTALNYYFNPSNPYVNAQVTVYQNSTTEKEQRKVSGVEDRTKLTTRGINLRNHSELPWFSLVYGVDYMQDKADTERGTN----NSDARY	394
	CP017484 (1f) locus BG572_01365	299	SYLSDQKINDKSTALNYYFNPSNPYVNAQVTVYQNSTTEKEQRKVSGVEDRTKLTTRGINLRNHSELPWFSLVYGVDYMQDKADTERGTN----NSDARY	394
	CP017499 (1i) locus BG576_01365	299	SYLSDQKINDKSTALNYYFNPSNPYVNAQVTVYQNSTTEKEQRKVSGVEDRTKLTTRGINLRNHSELPWFSLVYGVDYMQDKADTERGTN----NSDARY	394
Ligand—gated channel Genotype 2 specific*	CP017538 (2b) locus BG586_02985	248	SYLSDQKITDKSTSLNYYFNPSNPYLSTQVTLYSNSTTENEHRKISGVEDQTKLSTRGVNIRNHSELSWFSLVYGVDYMLDKVATQRGRN----NTDAQY	343
	CP017491 (2c) locus BG598_01520	248	SYLSDQKITDKSTSLNYYFNPSNPYLSTQVTLYSNSTTENEHRKISGVEDQTKLSTRGVNIRNHSELSWFSLVYGVDYMLDKVATQRGRN----NTDAQY	343
	CP017505 (2d) locus BG605_01515	248	SYLSDQKITDKSTSLNYYFNPSNPYLSTQVTLYSNSTTENEHRKISGVEDQTKLSTRGVNIRNHSELSWFSLVYGVDYMLDKVATQRGRN----NTDAQY	343
	CP017552 (2e) locus BG607_01530	248	SYLSDQKITDKSTSLNYYFNPSNPYLSTQVTLYSNSTTENEHRKISGVEDQTKLSTRGVNIRNHSELSWFSLVYGVDYMLDKVATQRGRN----NTDAQY	343
Ligand—gated channel Genotype 1	CP017495 (1b) locus BG548_02105	234	QNPLPG-FEEAKISANYVDYYHDEKDGKRVENYFKNGKNLRFELVHKEWKGLKGAIGVQYTNQSTSALALEASRAAKVFNKQPLLNNPKTKLWSLFAIE	332
	CP017510 (1c) locus BG556_02105	234	QNPLPG-FEEAKISANYVDYYHDEKDGKRVENYFKNGKNLRFELVHKEWKGLKGAIGVQYTNQSTSALALEASRAAKVFNKQPLLNNPKTKLWSLFAIE	332
	CP017502 (1e) locus BG561_02105	234	QNPLPG-FEEAKISANYVDYYHDEKDGKRVENYFKNGKNLRFELVHKEWKGLKGAIGVQYTNQSTSALALEASRAAKVFNKQPLLNNPKTKLWSLFAIE	332
	CP017484 (1f) locus BG572_02105	234	QNPLPG-FEEAKISANYVDYYHDEKDGKRVENYFKNGKNLRFELVHKEWKGLKGAIGVQYTNQSTSALALEASRAAKVFNKQPLLNNPKTKLWSLFAIE	332
	CP017499 (1i) locus BG576_10725	234	QNPLPG-FEEAKISANYVDYYHDEKDGKRVENYFKNGKNLRFELVHKEWKGLKGAIGVQYTNQSTSALALEASRAAKVFNKQPLLNNPKTKLWSLFAIE	332
Ligand—gated channel Genotype 2	CP017538 (2b) locus BG586_02215	234	QNPLPG-FEEAKISANYVDYYHDEKDGKRVENYFKNGKNLRFELVHKEWKGLKGAIGVQYTNQSTSALALEASRAAKVFNKQPLLNNPKTKLWSLFAIE	332
	CP017491 (2c) locus BG598_02290	234	QNPLPG-FEEAKISANYVDYYHDEKDGKRVENYFKNGKNLRFELVHKEWKGLKGAIGVQYTNQSTSALALEASRAAKVFNKQPLLNNPKTKLWSLFAIE	332
	CP017505 (2d) locus BG605_02285	234	QNPLPG-FEEAKISANYVDYYHDEKDGKRVENYFKNGKNLRFELVHKEWKGLKGAIGVQYTNQSTSALALEASRAAKVFNKQPLLNNPKTKLWSLFAIE	332
	CP017552 (2e) locus BG607_02300	234	QNPLPG-FEEAKISANYVDYYHDEKDGKRVENYFKNGKNLRFELVHKEWKGLKGAIGVQYTNQSTSALALEASRAAKVFNKQPLLNNPKTKLWSLFAIE	332

Fig S6 continued

Group	Sequence	Start	Alignment	End
Ligand—gated channel Genotype 1	CP017495 (1b) locus BG548_01365	395	RSNPYQAKSNTTGAYLIAHIPLWGEKLLFSPSVRYDRFNTSSEAVKYQDSHWSPAAKLTWKATTWLDFTAKYNEAFRAPSMQERFTGGSHFGTQGQGGAP	494
	CP017510 (1c) locus BG556_01365	395	RSNPYQAKSNTTGAYLIAHIPLWGEKLLFSPSVRYDRFNTSSEAVKYQDSHWSPAAKLTWKATTWLDFTAKYNEAFRAPSMQERFTGGSHFGTQGQGGAP	494
	CP017502 (1e) locus BG561_01365	395	RSNPYQAKSNTTGAYLIAHIPLWGEKLLFSPSVRYDRFNTSSEAVKYQDSHWSPAAKLTWKATTWLDFTAKYNEAFRAPSMQERFTGGSHFGTQGQGGAP	494
	CP017484 (1f) locus BG572_01365	395	RSNPYQAKSNTTGAYLIAHIPLWGEKLLFSPSVRYDRFNTSSEAVKYQDSHWSPAAKLTWKATTWLDFTAKYNEAFRAPSMQERFTGGSHFGTQGQGGAP	494
	CP017499 (1i) locus BG576_01365	395	RSNPYQAKSNTTGAYLIAHIPLWGEKLLFSPSVRYDRFNTSSEAVKYQDSHWSPAAKLTWKATTWLDFTAKYNEAFRAPSMQERFTGGSHFGTQGQGGAP	494
Ligand—gated channel Genotype 2 specific*	CP017538 (2b) locus BG586_02985	344	RANPYDAKSKTTGAYLIAHIPLFDEKVVFSPSVRYDRFDTSSETVKYKDSHWSPAAKLTWKATNWLDLTAKYNEAFRAPSMQERFVSGSHFGTTVRG-LP	442
	CP017491 (2c) locus BG598_01520	344	RANPYDAKSKTTGAYLIAHIPLFDEKVVFSPSVRYDRFDTSSETVKYKDSHWSPAAKLTWKATNWLDLTAKYNEAFRAPSMQERFVSGSHFGTTVRG-LP	442
	CP017505 (2d) locus BG605_01515	344	RANPYDAKSKTTGAYLIAHIPLFDEKVVFSPSVRYDRFDTSSETVKYKDSHWSPAAKLTWKATNWLDLTAKYNEAFRAPSMQERFVSGSHFGTTVRG-LP	442
	CP017552 (2e) locus BG607_01530	344	RANPYDAKSKTTGAYLIAHIPLFDEKVVFSPSVRYDRFDTSSETVKYKDSHWSPAAKLTWKATNWLDLTAKYNEAFRAPSMQERFVSGSHFGTTVRG-LP	442
Ligand—gated channel Genotype 1	CP017495 (1b) locus BG548_02105	333	RLNLGDFTFELSGRAERQKIAMDYDVKLIDRWLGFNTPMPNLDPHKDKGYSYSFATHWYFAPNHKLTLNAAHQERL--PNAQELYAHGKHI--------A	422
	CP017510 (1c) locus BG556_02105	333	RLNLGDFTFELSGRAERQKIAMDYDVKLIDRWLGFNTPMPNLDPHKDKGYSYSFATHWYFAPNHKLTLNAAHQERL--PNAQELYAHGKHI--------A	422
	CP017502 (1e) locus BG561_02105	333	RLNLGDFTFELSGRAERQKIAMDYDVKLIDRWLGFNTPMPNLDPHKDKGYSYSFATHWYFAPNHKLTLNAAHQERL--PNAQELYAHGKHI--------A	422
	CP017484 (1f) locus BG572_02105	333	RLNLGDFTFELSGRAERQKIAMDYDVKLIDRWLGFNTPMPNLDPHKDKGYSYSFATHWYFAPNHKLTLNAAHQERL--PNAQELYAHGKHI--------A	422
	CP017499 (1i) locus BG576_10725	333	RLNLGDFTFELSGRAERQKIAMDYDVKLIDRWLGFNTPMPNLDPHKDKGYSYSFATHWYFAPNHKLTLNAAHQERL--PNAQELYAHGKHI--------A	422
Ligand—gated channel Genotype 2	CP017538 (2b) locus BG586_02215	333	RLNLGDFTFELSGRAERQKIAMDYDVKLIDRWLGFNTPMPNLDPHKDKGYSYSFATHWYFAPNHKLTLNAAHQERL--PNAQELYAHGKHI--------A	422
	CP017491 (2c) locus BG598_02290	333	RLNLGDFTFELSGRAERQKIAMDYDVKLIDRWLGFNTPMPNLDPHKDKGYSYSFATHWYFAPNHKLTLNAAHQERL--PNAQELYAHGKHI--------A	422
	CP017505 (2d) locus BG605_02285	333	RLNLGDFTFELSGRAERQKIAMDYDVKLIDRWLGFNTPMPNLDPHKDKGYSYSFATHWYFAPNHKLTLNAAHQERL--PNAQELYAHGKHI--------A	422
	CP017552 (2e) locus BG607_02300	333	RLNLGDFTFELSGRAERQKIAMDYDVKLIDRWLGFNTPMPNLDPHKDKGYSYSFATHWYFAPNHKLTLNAAHQERL--PNAQELYAHGKHI--------A	422

Group	Sequence	Start	Alignment	End
Ligand—gated channel Genotype 1	CP017495 (1b) locus BG548_01365	495	IINIFVQNPNLRPETAKNKEITAHVHFDDLITQGDKFSVEATYFRNDVRDFINLQLYKAN--PASPMEVIPTRSQYRNVTNARLSGVELQTQYQTER---	589
	CP017510 (1c) locus BG556_01365	495	IINIFVQNPNLRPETAKNKEITAHVHFDDLITQGDKFSVEATYFRNDVRDFINLQLYKAN--PASPMEVIPTRSQYRNVTNARLSGVELQTQYQTER---	589
	CP017502 (1e) locus BG561_01365	495	IINIFVQNPNLRPETAKNKEITAHVHFDDLITQGDKFSVEATYFRNDVRDFINLQLYKAN--PASPMEVIPTRSQYRNVTNARLSGVELQTQYQTER---	589
	CP017484 (1f) locus BG572_01365	495	IINIFVQNPNLRPETAKNKEITAHVHFDDLITQGDKFSVEATYFRNDVRDFINLQLYKAN--PASPMEVIPTRSQYRNVTNARLSGVELQTQYQTER---	589
	CP017499 (1i) locus BG576_01365	495	IINIFVQNPNLRPETAKNKEITAHVHFDDLITQGDKFSVEATYFRNDVRDFINLQLYKAN--PASPMEVIPTRSQYRNVTNARLSGVELQTQYQTER---	589
Ligand—gated channel Genotype 2 specific*	CP017538 (2b) locus BG586_02985	443	INNIFLTNPNLRPETAKNKEVTFNVHFDNVISQGDKFNFEATYFRNDVKDLINLQVFKAN--PTSPMEFIPTRSQYQNIANARLSGIELQTRYQTER---	537
	CP017491 (2c) locus BG598_01520	443	INNIFLTNPNLRPETAKNKEVTFNVHFDNVISQGDKFNFEATYFRNDVKDLINLQVFKAN--PTSPMEFIPTRSQYQNIANARLSGIELQTRYQTER---	537
	CP017505 (2d) locus BG605_01515	443	INNIFLTNPNLRPETAKNKEVTFNVHFDNVISQGDKFNFEATYFRNDVKDLINLQVFKAN--PTSPMEFIPTRSQYQNIANARLSGIELQTRYQTER---	537
	CP017552 (2e) locus BG607_01530	443	INNIFLTNPNLRPETAKNKEVTFNVHFDNVISQGDKFNFEATYFRNDVKDLINLQVFKAN--PTSPMEFIPTRSQYQNIANARLSGIELQTRYQTER---	537
Ligand—gated channel Genotype 1	CP017495 (1b) locus BG548_02105	423	LNAFEAGNKNLEKERSNQIELS-------LAYVGDKWDYKLNLYHTRYGNYIYPLTLNDNRGPKSFTDEYNLKVNRYYQGEARFSGAEGEIGYLFTPNYR	515
	CP017510 (1c) locus BG556_02105	423	LNAFEAGNKNLEKERSNQIELS-------LAYVGDKWDYKLNLYHTRYGNYIYPLTLNDNRGPKSFTDEYNLKVNRYYQGEARFSGAEGEIGYLFTPNYR	515
	CP017502 (1e) locus BG561_02105	423	LNAFEAGNKNLEKERSNQIELS-------LAYVGDKWDYKLNLYHTRYGNYIYPLTLNDNRGPKSFTDEYNLKVNRYYQGEARFSGAEGEIGYLFTPNYR	515
	CP017484 (1f) locus BG572_02105	423	LNAFEAGNKNLEKERSNQIELS-------LAYVGDKWDYKLNLYHTRYGNYIYPLTLNDNRGPKSFTDEYNLKVNRYYQGEARFSGAEGEIGYLFTPNYR	515
	CP017499 (1i) locus BG576_10725	423	LNAFEAGNKNLEKERSNQIELS-------LAYVGDKWDYKLNLYHTRYGNYIYPLTLNDNRGPKSFTDEYNLKVNRYYQGEARFSGAEGEIGYLFTPNYR	515
Ligand—gated channel Genotype 2	CP017538 (2b) locus BG586_02215	423	LNAFEAGNKNLKKERSNQIELS-------LAYVGDKWDYKLNLYHTRYGNYIYPLTLNDNRGPKSFTDEYNLKVNRYYQGEARFSGAEGEIGYLFTPNYR	515
	CP017491 (2c) locus BG598_02290	423	LNAFEAGNKNLKKERSNQIELS-------LAYVGDKWDYKLNLYHTRYGNYIYPLTLNDNRGPKSFTDEYNLKVNRYYQGEARFSGAEGEIGYLFTPNYR	515
	CP017505 (2d) locus BG605_02285	423	LNAFEAGNKNLKKERSNQIELS-------LAYVGDKWDYKLNLYHTRYGNYIYPLTLNDNRGPKSFTDEYNLKVNRYYQGEARFSGAEGEIGYLFTPNYR	515
	CP017552 (2e) locus BG607_02300	423	LNAFEAGNKNLKKERSNQIELS-------LAYVGDKWDYKLNLYHTRYGNYIYPLTLNDNRGPKSFTDEYNLKVNRYYQGEARFSGAEGEIGYLFTPNYR	515

Fig S6 continued

```
Ligand—gated channel Genotype 1
CP017495 (1b) locus BG548_01365  590 VTFLANYGSTKGKDKDSGEALSNIAASKIG-FGVNYAVVQDKFTVGANVTRYQAQHRVPKNHGVTYQGYTLTDLHATYAPLKGEWKNLRLDLAVENLFDK 688
CP017510 (1c) locus BG556_01365  590 VTFLANYGSTKGKDKDSGEALSNIAASKIG-FGVNYAVVQDKFTVGANVTRYQAQHRVPKNHGVTYQGYTLTDLHATYAPLKGEWKNLRLDLAVENLFDK 688
CP017502 (1e) locus BG561_01365  590 VTFLANYGSTKGKDKDSGEALSNIAASKIG-FGVNYAVVQDKFTVGANVTRYQAQHRVPKNHGVTYQGYTLTDLHATYAPLKGEWKNLRLDLAVENLFDK 688
CP017484 (1f) locus BG572_01365  590 VTFLANYGSTKGKDKDSGEALSNIAASKIG-FGVNYAVVQDKFTVGANVTRYQAQHRVPKNHGVTYQGYTLTDLHATYAPLKGEWKNLRLDLAVENLFDK 688
CP017499 (1i) locus BG576_01365  590 VTFLANYGSTKGKDKDSGEALSNIAASKIG-FGVNYAVVQDKFTVGANVTRYQAQHRVPKNHGVTYQGYTLTDLHATYAPLKGEWKNLRLDLAVENLFDK 688
Ligand—gated channel Genotype 2 specific*
CP017538 (2b) locus BG586_02985  538 LAVFANYGSTKGKDKDSGEALSNIAASKIG-FGVNYAVVQDKFTVGANVTRYQAQHRVPKNHGVTYQGYTLTDLHATYAPLKGEWKNLRLDVAVENLFDK 636
CP017491 (2c) locus BG598_01520  538 LAVFANYGSTKGKDKDSGEALSNIAASKIG-FGVNYAVVQDKFTVGANVTRYQAQHRVPKNHGVTYQGYTLTDLHATYAPLKGEWKNLRLDVAVENLFDK 636
CP017505 (2d) locus BG605_01515  538 LAVFANYGSTKGKDKDSGEALSNIAASKIG-FGVNYAVVQDKFTVGANVTRYQAQHRVPKNHGVTYQGYTLTDLHATYAPLKGEWKNLRLDVAVENLFDK 636
CP017552 (2e) locus BG607_01530  538 LAVFANYGSTKGKDKDSGEALSNIAASKIG-FGVNYAVVQDKFTVGANVTRYQAQHRVPKNHGVTYQGYTLTDLHATYAPLKGEWKNLRLDVAVENLFDK 636
Ligand—gated channel Genotype 1
CP017495 (1b) locus BG548_02105  516 LAVFGDYVRGKLVNLPNIAMSYNIWTGEVDKWASQPDISAPRIPPLRLGARFNADFNLNWSGMLEYYRVFAQKKVSKYEQVTPGHHQVNLGVTYSNHFNQ 615
CP017510 (1c) locus BG556_02105  516 LAVFGDYVRGKLVNLPNIAMSYNIWTGEVDKWASQPDISAPRIPPLRLGARFNADFNLNWSGMLEYYRVFAQKKVSKYEQVTPGHHQVNLGVTYSNHFNQ 615
CP017502 (1e) locus BG561_02105  516 LAVFGDYVRGKLVNLPNIAMSYNIWTGEVDKWASQPDISAPRIPPLRLGARFNADFNLNWSGMLEYYRVFAQKKVSKYEQVTPGHHQVNLGVTYSNHFNQ 615
CP017484 (1f) locus BG572_02105  516 LAVFGDYVRGKLVNLPNIAMSYNIWTGEVDKWASQPDISAPRIPPLRLGARFNADFNLNWSGMLEYYRVFAQKKVSKYEQVTPGHHQVNLGVTYSNHFNQ 615
CP017499 (1i) locus BG576_10725  516 LAVFGDYVRGKLVNLPNIAMSYNIWTGEVDKWASQPDISAPRIPPLRLGARFNADFNLNWSGMLEYYRVFAQKKVSKYEQVTPGHHQVNLGVTYSNHFNQ 615
Ligand—gated channel Genotype 2
CP017538 (2b) locus BG586_02215  516 LAVFGDYVRGKLVNLPNIAMSYNIWTGEVDKWASQPDISAPRIPPLRLGARFNADFNLNWSGMLEYYRVFAQKKVSKYEQVTPGHHQVNLGVTYSNHFNQ 615
CP017491 (2c) locus BG598_02290  516 LAVFGDYVRGKLVNLPNIAMSYNIWTGEVDKWASQPDISAPRIPPLRLGARFNADFNLNWSGMLEYYRVFAQKKVSKYEQVTPGHHQVNLGVTYSNHFNQ 615
CP017505 (2d) locus BG605_02285  516 LAVFGDYVRGKLVNLPNIAMSYNIWTGEVDKWASQPDISAPRIPPLRLGARFNADFNLNWSGMLEYYRVFAQKKVSKYEQVTPGHHQVNLGVTYSNHFNQ 615
CP017552 (2e) locus BG607_02300  516 LAVFGDYVRGKLVNLPNIAMSYNIWTGEVDKWASQPDISAPRIPPLRLGARFNADFNLNWSGMLEYYRVFAQKKVSKYEQVTPGHHQVNLGVTYSNHFNQ 615
```

```
Ligand—gated channel Genotype 1
CP017495 (1b) locus BG548_01365  689 KYQPAFSLME------------------GSGRNVKLSAAYSF 712
CP017510 (1c) locus BG556_01365  689 KYQPAFSLME------------------GSGRNVKLSAAYSF 712
CP017502 (1e) locus BG561_01365  689 KYQPAFSLME------------------GSGRNVKLSAAYSF 712
CP017484 (1f) locus BG572_01365  689 KYQPAFSLME------------------GSGRNVKLSAAYSF 712
CP017499 (1i) locus BG576_01365  689 KYQPAFSLME------------------GSGRNVKLSAAYSF 712
Ligand—gated channel Genotype 2 specific*
CP017538 (2b) locus BG586_02985  637 KYQPAFSLME------------------GSGRNVKLSAAYSF 660
CP017491 (2c) locus BG598_01520  637 KYQPAFSLME------------------GSGRNVKLSAAYSF 660
CP017505 (2d) locus BG605_01515  637 KYQPAFSLME------------------GSGRNVKLSAAYSF 660
CP017552 (2e) locus BG607_01530  637 KYQPAFSLME------------------GSGRNVKLSAAYSF 660
Ligand—gated channel Genotype 1
CP017495 (1b) locus BG548_02105  616 TEYQVFLKVDNLLNQKMYQHASYLPHIPQMGRNAMLGMNISF 657
CP017510 (1c) locus BG556_02105  616 TEYQVFLKVDNLLNQKMYQHASYLPHIPQMGRNAMLGMNISF 657
CP017502 (1e) locus BG561_02105  616 TEYQVFLKVDNLLNQKMYQHASYLPHIPQMGRNAMLGMNISF 657
CP017484 (1f) locus BG572_02105  616 TEYQVFLKVDNLLNQKMYQHASYLPHIPQMGRNAMLGMNISF 657
CP017499 (1i) locus BG576_10725  616 TEYQVFLKVDNLLNQKMYQHASYLPHIPQMGRNAMLGMNISF 657
Ligand—gated channel Genotype 2
CP017538 (2b) locus BG586_02215  616 TEYQVFLKVDNLLNQKMYQHASYLPHIPQMGRNAMLGMNISF 657
CP017491 (2c) locus BG598_02290  616 TEYQVFLKVDNLLNQKMYQHASYLPHIPQMGRNAMLGMNISF 657
CP017505 (2d) locus BG605_02285  616 TEYQVFLKVDNLLNQKMYQHASYLPHIPQMGRNAMLGMNISF 657
CP017552 (2e) locus BG607_02300  616 TEYQVFLKVDNLLNQKMYQHASYLPHIPQMGRNAMLGMNISF 657
```
