# Supplementary material for: Differences between predicted outer membrane proteins of genotype 1 and 2 Mannheimia haemolytica
Source: BMC Microbiol. 2020 Aug 12;20:250. doi: 10.1186/s12866-020-01932-2 (PMC7424683; doi:10.1186/s12866-020-01932-2)

Fig S7

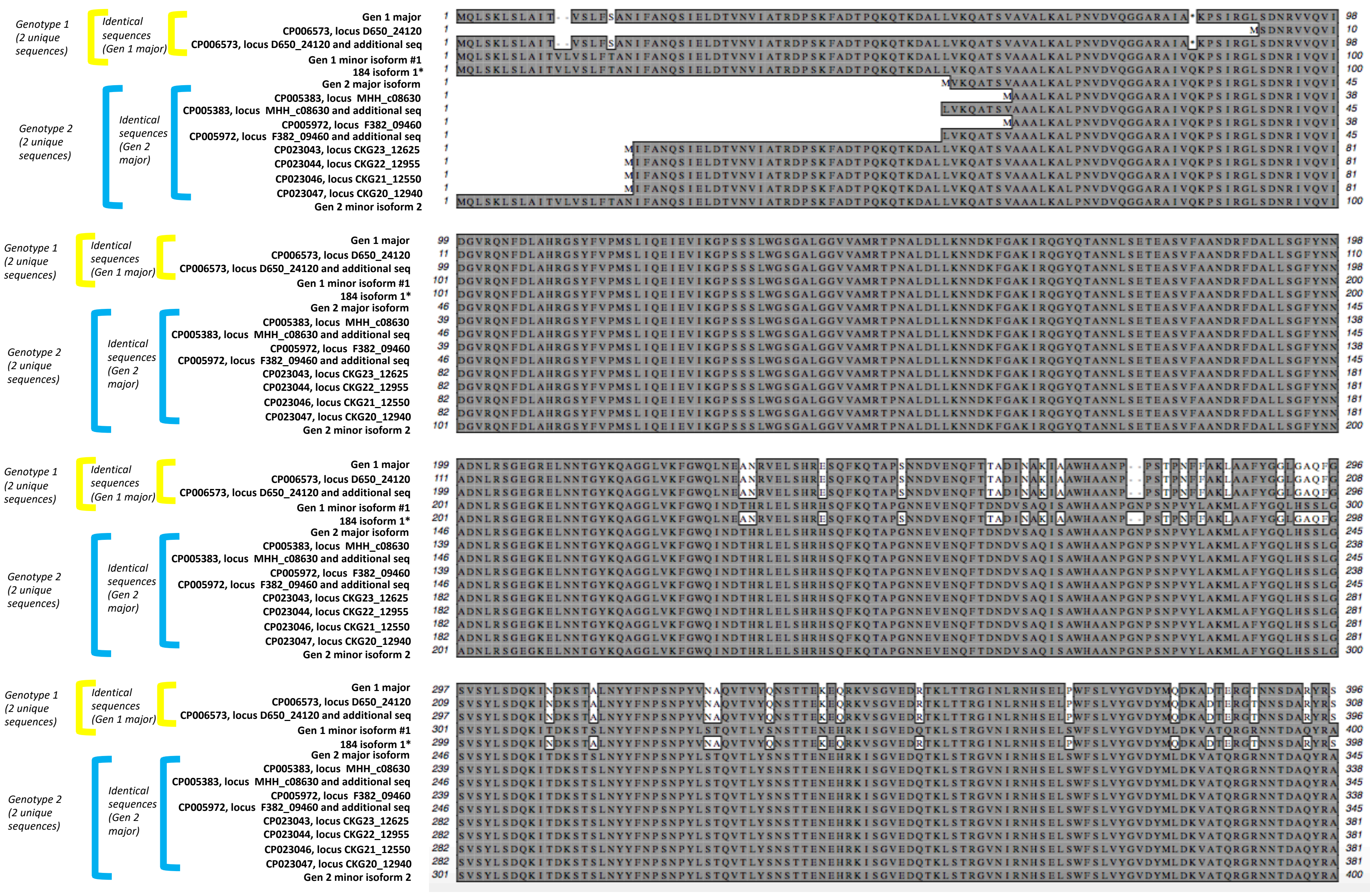

Genotype 1 (2 unique sequences)
Identical sequences (Gen 1 major)
Gen 1 major
CP006573, locus D650_24120
CP006573, locus D650_24120 and additional seq
Gen 1 minor isoform #1
184 isoform 1*
Genotype 2 (2 unique sequences)
Identical sequences (Gen 2 major)
Gen 2 major isoform
CP005383, locus MHH_c08630
CP005383, locus MHH_c08630 and additional seq
CP005972, locus F382_09460
CP005972, locus F382_09460 and additional seq
CP023043, locus CKG23_12625
CP023044, locus CKG22_12955
CP023046, locus CKG21_12550
CP023047, locus CKG20_12940
Gen 2 minor isoform 2

Fig S7 continued

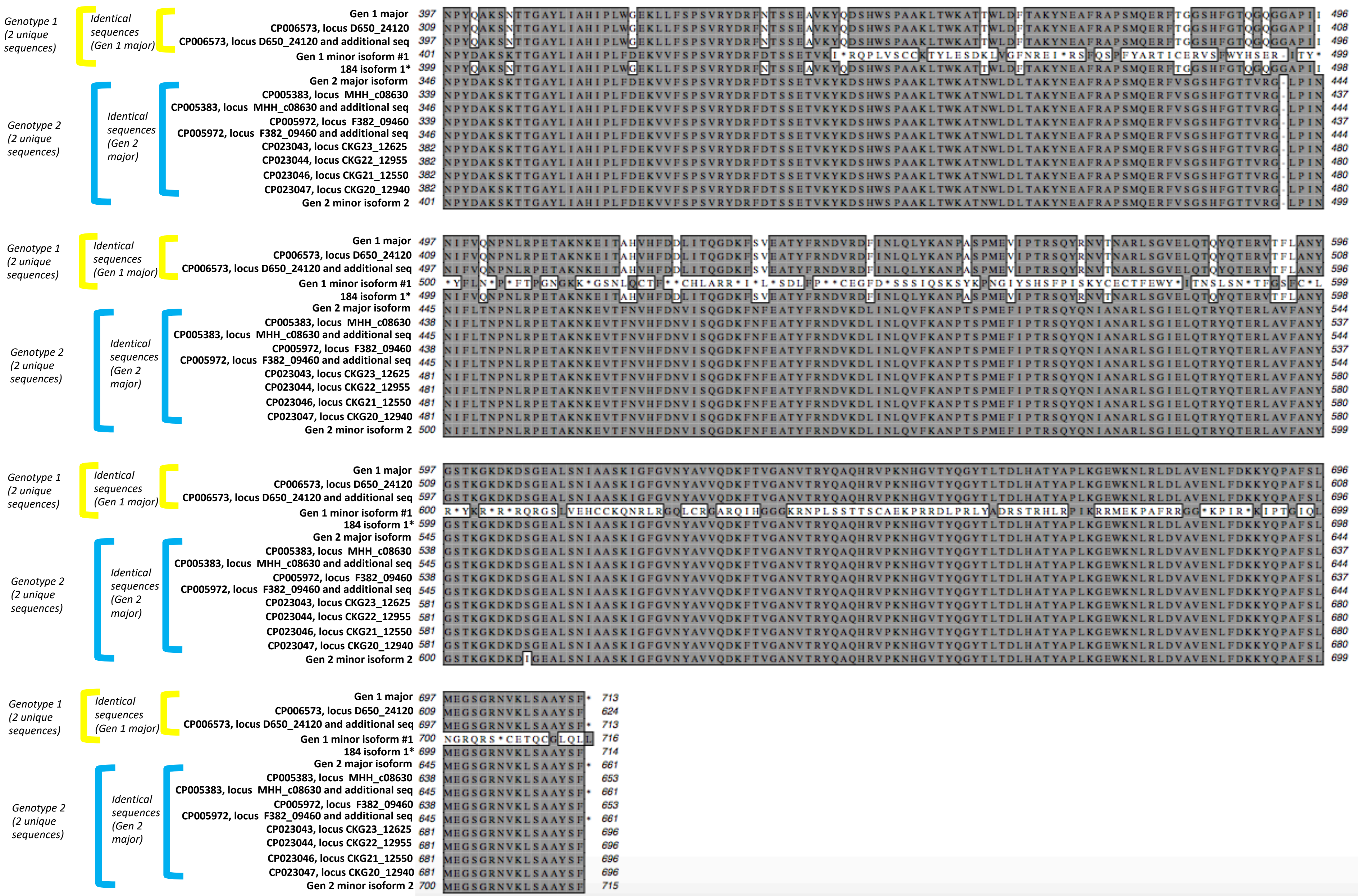

Supplement: Supplementary file 12 — Additional file 12: Figure S7. Alignment of all detected isoforms of the ligand-gated channel specific to genotype 2 M. haemolytica and those of the homologous pseudogene in genotype 1 M. haemolytica. Within the alignment, sequences corresponding to the major isoform of genotype 1 and the major isoform of genotype 2 are highlighted with brackets. *184 isoform 1 is an abbreviation for USDA-ARS-USMARC-184 isoform 1 (ligand-gated channel). Areas of 51% chemical identity or greater are indicated with grey boxes within the alignment. [file 12866_2020_1932_MOESM12_ESM.pdf]
