# Supplementary material for: Differences between predicted outer membrane proteins of genotype 1 and 2 Mannheimia haemolytica
Source: BMC Microbiol. 2020 Aug 12;20:250. doi: 10.1186/s12866-020-01932-2 (PMC7424683; doi:10.1186/s12866-020-01932-2)
Supplement: Supplementary file 13 — Additional file 13: Figure S8. Alignment of genotype 1 pseudogene adhesin B1 and genotype 2 adhesin B isoform sequences. Represented within the alignment are pseudogene B1 adhesin isoforms from five genotype 1 strains that are each of a different genotype, and B adhesin isoforms from four genotype 2 strains that are also each of a different subtype. Areas of 51% chemical identity or greater are indicated with grey boxes. [file 12866_2020_1932_MOESM13_ESM.pdf]

Fig S8

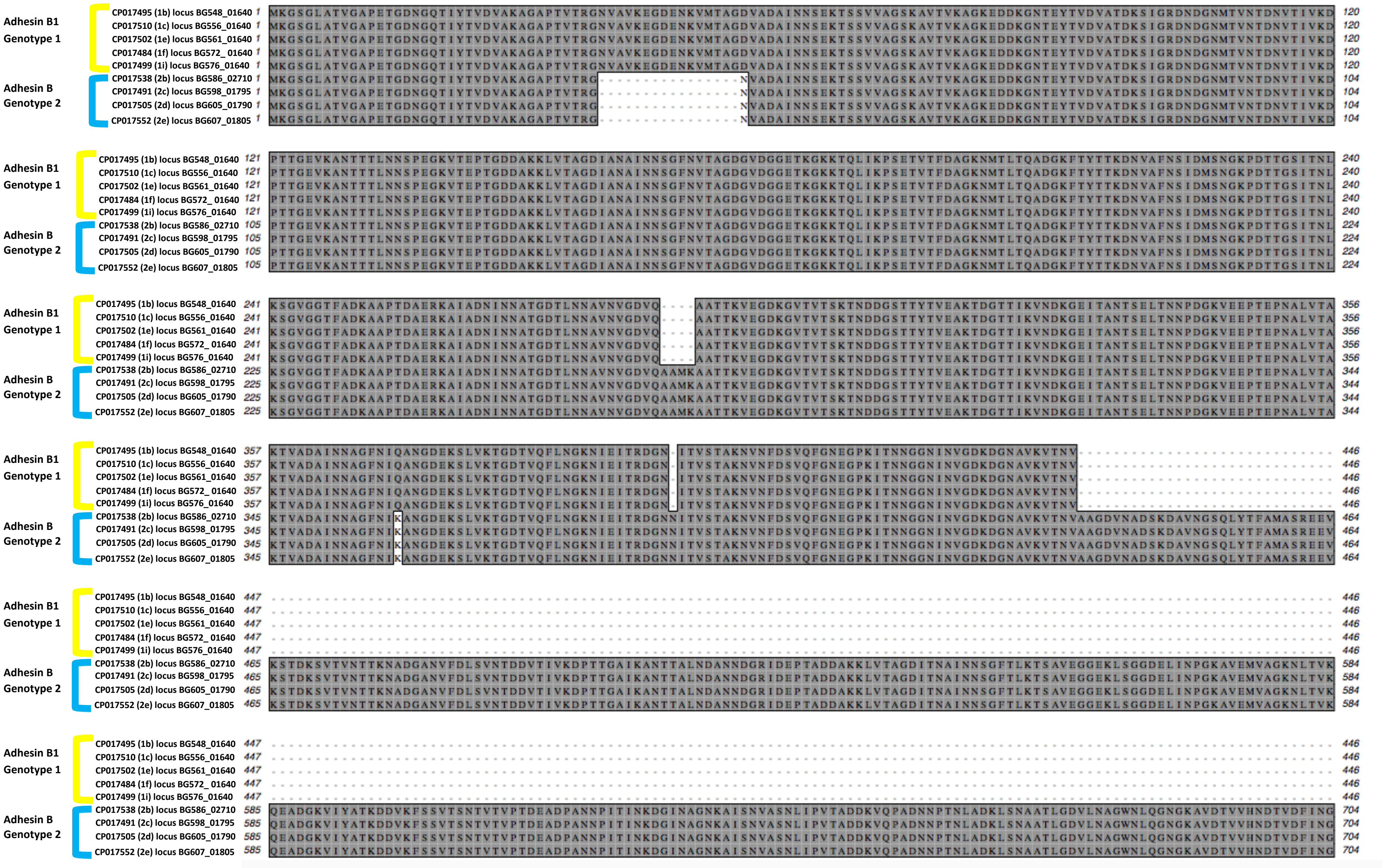
Adhesin B1 Genotype 1
CP017495 (1b) locus BG548_01640
CP017510 (1c) locus BG556_01640
CP017502 (1e) locus BG561_01640
CP017484 (1f) locus BG572_01640
CP017499 (1i) locus BG576_01640
Adhesin B Genotype 2
CP017538 (2b) locus BG586_02710
CP017491 (2c) locus BG598_01795
CP017505 (2d) locus BG605_01790
CP017552 (2e) locus BG607_01805

Fig S8 continued

Group	Sequence	Start	Alignment	End
Adhesin B1 Genotype 1	CP017495 (1b) locus BG548_01640	447		446
	CP017510 (1c) locus BG556_01640	447		446
	CP017502 (1e) locus BG561_01640	447		446
	CP017484 (1f) locus BG572_ 01640	447		446
	CP017499 (1i) locus BG576_01640	447		446
Adhesin B Genotype 2	CP017538 (2b) locus BG586_02710	705	KGTTVTVENKDGKNTIKVDSPIEFVNQDPTDSSTPSNTAKFTGEAPVQLGNVASSVRNEDGSTPEGKDRAEAIKNAEGDKLNNVVNLGDLQAATNAATTKVGGNRGVTITPSTNADGSTT	824
	CP017491 (2c) locus BG598_01795	705	KGTTVTVENKDGKNTIKVDSPIEFVNQDPTDSSTPSNTAKFTGEAPVQLGNVASSVRNEDGSTPEGKDRAEAIKNAEGDKLNNVVNLGDLQAATNAATTKVGGNRGVTITPSTNADGSTT	824
	CP017505 (2d) locus BG605_01790	705	KGTTVTVENKDGKNTIKVDSPIEFVNQDPTDSSTPSNTAKFTGEAPVQLGNVASSVRNEDGSTPEGKDRAEAIKNAEGDKLNNVVNLGDLQAATNAATTKVGGNRGVTITPSTNADGSTT	824
	CP017552 (2e) locus BG607_01805	705	KGTTVTVENKDGKNTIKVDSPIEFVNQDPTDSSTPSNTAKFTGEAPVQLGNVASSVRNEDGSTPEGKDRAEAIKNAEGDKLNNVVNLGDLQAATNAATTKVGGNRGVTITPSTNADGSTT	824

Group	Sequence	Start	Alignment	End
Adhesin B1 Genotype 1	CP017495 (1b) locus BG548_01640	447		446
	CP017510 (1c) locus BG556_01640	447		446
	CP017502 (1e) locus BG561_01640	447		446
	CP017484 (1f) locus BG572_ 01640	447		446
	CP017499 (1i) locus BG576_01640	447		446
Adhesin B Genotype 2	CP017538 (2b) locus BG586_02710	825	YNVEAKTDGTTIKVDNEGNITANTSELGNNEDGTVKAPTQPNALLIAQTVADAVNNAGFNIKSAGNKAAGDQAATKLVKTGEEVVFEAGDNLTVKRDGNQFTFATAKDVSFNSVQFSENG	944
	CP017491 (2c) locus BG598_01795	825	YNVEAKTDGTTIKVDNEGNITANTSELGNNEDGTVKAPTQPNALLIAQTVADAVNNAGFNIKSAGNKAAGDQAATKLVKTGEEVVFEAGDNLTVKRDGNQFTFATAKDVSFNSVQFSENG	944
	CP017505 (2d) locus BG605_01790	825	YNVEAKTDGTTIKVDNEGNITANTSELGNNEDGTVKAPTQPNALLIAQTVADAVNNAGFNIKSAGNKAAGDQAATKLVKTGEEVVFEAGDNLTVKRDGNQFTFATAKDVSFNSVQFSENG	944
	CP017552 (2e) locus BG607_01805	825	YNVEAKTDGTTIKVDNEGNITANTSELGNNEDGTVKAPTQPNALLIAQTVADAVNNAGFNIKSAGNKAAGDQAATKLVKTGEEVVFEAGDNLTVKRDGNQFTFATAKDVSFNSVQFSENG	944

Group	Sequence	Start	Alignment	End
Adhesin B1 Genotype 1	CP017495 (1b) locus BG548_01640	447		446
	CP017510 (1c) locus BG556_01640	447		446
	CP017502 (1e) locus BG561_01640	447		446
	CP017484 (1f) locus BG572_ 01640	447		446
	CP017499 (1i) locus BG576_01640	447		446
Adhesin B Genotype 2	CP017538 (2b) locus BG586_02710	945	PKITNDGDNIKVGDKDGKPTKITNVADGDISPVSTDVINGKQLNNYAKVNGNNIGTDEDGSINIVNGNGTTITSDKAGEVKVNVNITDLTVADNGKINVQDPNGTGSRFVNATTVANAVN	1064
	CP017491 (2c) locus BG598_01795	945	PKITNDGDNIKVGDKDGKPTKITNVADGDISPVSTDVINGKQLNNYAKVNGNNIGTDEDGSINIVNGNGTTITSDKAGEVKVNVNITDLTVADNGKINVQDPNGTGSRFVNATTVANAVN	1064
	CP017505 (2d) locus BG605_01790	945	PKITNDGDNIKVGDKDGKPTKITNVADGDISPVSTDVINGKQLNNYAKVNGNNIGTDEDGSINIVNGNGTTITSDKAGEVKVNVNITDLTVADNGKINVQDPNGTGSRFVNATTVANAVN	1064
	CP017552 (2e) locus BG607_01805	945	PKITNDGDNIKVGDKDGKPTKITNVADGDISPVSTDVINGKQLNNYAKVNGNNIGTDEDGSINIVNGNGTTITSDKAGEVKVNVNITDLTVADNGKINVQDPNGTGSRFVNATTVANAVN	1064

Group	Sequence	Start	Alignment	End
Adhesin B1 Genotype 1	CP017495 (1b) locus BG548_01640	447		446
	CP017510 (1c) locus BG556_01640	447		446
	CP017502 (1e) locus BG561_01640	447		446
	CP017484 (1f) locus BG572_ 01640	447		446
	CP017499 (1i) locus BG576_01640	447		446
Adhesin B Genotype 2	CP017538 (2b) locus BG586_02710	1065	NVSWNVDSKAVGTGAVEGDKAPAKVKAGSTVSVNAGNNIKVTRKGSDVTVAVSDTPEFTSKTGDTLVNNNGVTINNGSAGKAVSLTKDGLNNGGNRITNVKAGEADTDAVNVGQLKGAVN	1184
	CP017491 (2c) locus BG598_01795	1065	NVSWNVDSKAVGTGAVEGDKAPAKVKAGSTVSVNAGNNIKVTRKGSDVTVAVSDTPEFTSKTGDTLVNNNGVTINNGSAGKAVSLTKDGLNNGGNRITNVKAGEADTDAVNVGQLKGAVN	1184
	CP017505 (2d) locus BG605_01790	1065	NVSWNVDSKAVGTGAVEGDKAPAKVKAGSTVSVNAGNNIKVTRKGSDVTVAVSDTPEFTSKTGDTLVNNNGVTINNGSAGKAVSLTKDGLNNGGNRITNVKAGEADTDAVNVGQLKGAVN	1184
	CP017552 (2e) locus BG607_01805	1065	NVSWNVDSKAVGTGAVEGDKAPAKVKAGSTVSVNAGNNIKVTRKGSDVTVAVSDTPEFTSKTGDTLVNNNGVTINNGSAGKAVSLTKDGLNNGGNRITNVKAGEADTDAVNVGQLKGAVN	1184

Group	Sequence	Start	Alignment	End
Adhesin B1 Genotype 1	CP017495 (1b) locus BG548_01640	447		446
	CP017510 (1c) locus BG556_01640	447		446
	CP017502 (1e) locus BG561_01640	447		446
	CP017484 (1f) locus BG572_ 01640	447		446
	CP017499 (1i) locus BG576_01640	447		446
Adhesin B Genotype 2	CP017538 (2b) locus BG586_02710	1185	HLNNKIHRNNREARAGIAGSNAAAALPQVYIPGKSMVAAAGGTFKGENALAVGYSRSSDNGKLILKLQGNANSRGDFGGGVGVGYQW	1271
	CP017491 (2c) locus BG598_01795	1185	HLNNKIHRNNREARAGIAGSNAAAALPQVYIPGKSMVAAAGGTFKGENALAVGYSRSSDNGKLILKLQGNANSRGDFGGGVGVGYQW	1271
	CP017505 (2d) locus BG605_01790	1185	HLNNKIHRNNREARAGIAGSNAAAALPQVYIPGKSMVAAAGGTFKGENALAVGYSRSSDNGKLILKLQGNANSRGDFGGGVGVGYQW	1271
	CP017552 (2e) locus BG607_01805	1185	HLNNKIHRNNREARAGIAGSNAAAALPQVYIPGKSMVAAAGGTFKGENALAVGYSRSSDNGKLILKLQGNANSRGDFGGGVGVGYQW	1271
