# Supplementary material for: Differences between predicted outer membrane proteins of genotype 1 and 2 Mannheimia haemolytica
Source: BMC Microbiol. 2020 Aug 12;20:250. doi: 10.1186/s12866-020-01932-2 (PMC7424683; doi:10.1186/s12866-020-01932-2)
Supplement: Supplementary file 14 — Additional file 14: Figure S9. Alignment of pseudogene adhesin B2 isoforms from five genotype 1 strains that are each of a different genotype and adhesin B isoforms from four genotype 2 strains that are also each of a different subtype. The alignment contains pseudogene adhesin B2 isoforms from five genotype 1 strains that are each of a different genotype and adhesin B isoforms from four genotype 2 strains that are also each of a different subtype. Areas of 51% chemical identity or greater are indicated with grey boxes. [file 12866_2020_1932_MOESM14_ESM.pdf]

Fig S9

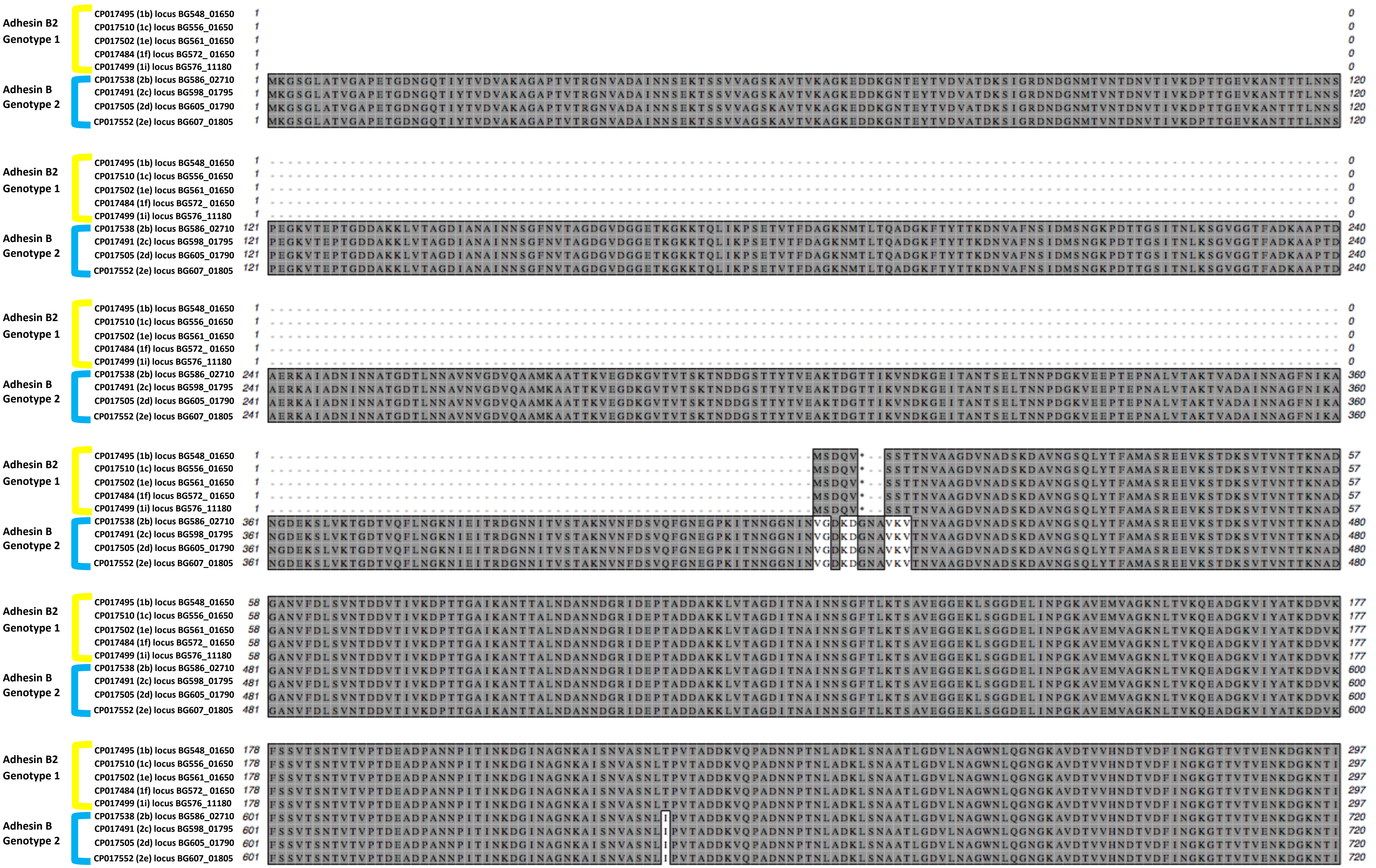
Adhesin B2
Genotype 1
Adhesin B
Genotype 2
CP017495 (1b) locus BG548_01650
CP017510 (1c) locus BG556_01650
CP017502 (1e) locus BG561_01650
CP017484 (1f) locus BG572_01650
CP017499 (1i) locus BG576_11180
CP017538 (2b) locus BG586_02710
CP017491 (2c) locus BG598_01795
CP017505 (2d) locus BG605_01790
CP017552 (2e) locus BG607_01805

Fig S9 continued

Group	Sequence	Start	Alignment	End
Adhesin B2 Genotype 1	CP017495 (1b) locus BG548_01650	298	KVDSPIEFVNQDPTDSSTPSNTAKFTGEAPVQLGNVASSVRNEDGSTPEGKDRAEAIKNAEGDKLNNVVNLGDLQAATNAATTKVGGNRGVTITPSTNADGSTTYNVEAKTDGTTIKVDN	417
	CP017510 (1c) locus BG556_01650	298	KVDSPIEFVNQDPTDSSTPSNTAKFTGEAPVQLGNVASSVRNEDGSTPEGKDRAEAIKNAEGDKLNNVVNLGDLQAATNAATTKVGGNRGVTITPSTNADGSTTYNVEAKTDGTTIKVDN	417
	CP017502 (1e) locus BG561_01650	298	KVDSPIEFVNQDPTDSSTPSNTAKFTGEAPVQLGNVASSVRNEDGSTPEGKDRAEAIKNAEGDKLNNVVNLGDLQAATNAATTKVGGNRGVTITPSTNADGSTTYNVEAKTDGTTIKVDN	417
	CP017484 (1f) locus BG572_ 01650	298	KVDSPIEFVNQDPTDSSTPSNTAKFTGEAPVQLGNVASSVRNEDGSTPEGKDRAEAIKNAEGDKLNNVVNLGDLQAATNAATTKVGGNRGVTITPSTNADGSTTYNVEAKTDGTTIKVDN	417
	CP017499 (1i) locus BG576_11180	298	KVDSPIEFVNQDPTDSSTPSNTAKFTGEAPVQLGNVASSVRNEDGSTPEGKDRAEAIKNAEGDKLNNVVNLGDLQAATNAATTKVGGNRGVTITPSTNADGSTTYNVEAKTDGTTIKVDN	417
Adhesin B Genotype 2	CP017538 (2b) locus BG586_02710	721	KVDSPIEFVNQDPTDSSTPSNTAKFTGEAPVQLGNVASSVRNEDGSTPEGKDRAEAIKNAEGDKLNNVVNLGDLQAATNAATTKVGGNRGVTITPSTNADGSTTYNVEAKTDGTTIKVDN	840
	CP017491 (2c) locus BG598_01795	721	KVDSPIEFVNQDPTDSSTPSNTAKFTGEAPVQLGNVASSVRNEDGSTPEGKDRAEAIKNAEGDKLNNVVNLGDLQAATNAATTKVGGNRGVTITPSTNADGSTTYNVEAKTDGTTIKVDN	840
	CP017505 (2d) locus BG605_01790	721	KVDSPIEFVNQDPTDSSTPSNTAKFTGEAPVQLGNVASSVRNEDGSTPEGKDRAEAIKNAEGDKLNNVVNLGDLQAATNAATTKVGGNRGVTITPSTNADGSTTYNVEAKTDGTTIKVDN	840
	CP017552 (2e) locus BG607_01805	721	KVDSPIEFVNQDPTDSSTPSNTAKFTGEAPVQLGNVASSVRNEDGSTPEGKDRAEAIKNAEGDKLNNVVNLGDLQAATNAATTKVGGNRGVTITPSTNADGSTTYNVEAKTDGTTIKVDN	840

Group	Sequence	Start	Alignment	End
Adhesin B2 Genotype 1	CP017495 (1b) locus BG548_01650	418	EGNITANTSELGNNEDGTVKAPTQPNALVTAQTVADAVNNAGFNIKSAGNKAAGDQAATKLVKTGEEVVFEAGDNLTVKRDGNQFTFATAKDVSFNSVQFSENGPKITNDGDNIKVGDKD	537
	CP017510 (1c) locus BG556_01650	418	EGNITANTSELGNNEDGTVKAPTQPNALVTAQTVADAVNNAGFNIKSAGNKAAGDQAATKLVKTGEEVVFEAGDNLTVKRDGNQFTFATAKDVSFNSVQFSENGPKITNDGDNIKVGDKD	537
	CP017502 (1e) locus BG561_01650	418	EGNITANTSELGNNEDGTVKAPTQPNALVTAQTVADAVNNAGFNIKSAGNKAAGDQAATKLVKTGEEVVFEAGDNLTVKRDGNQFTFATAKDVSFNSVQFSENGPKITNDGDNIKVGDKD	537
	CP017484 (1f) locus BG572_ 01650	418	EGNITANTSELGNNEDGTVKAPTQPNALVTAQTVADAVNNAGFNIKSAGNKAAGDQAATKLVKTGEEVVFEAGDNLTVKRDGNQFTFATAKDVSFNSVQFSENGPKITNDGDNIKVGDKD	537
	CP017499 (1i) locus BG576_11180	418	EGNITANTSELGNNEDGTVKAPTQPNALVTAQTVADAVNNAGFNIKSAGNKAAGDQAATKLVKTGEEVVFEAGDNLTVKRDGNQFTFATAKDVSFNSVQFSENGPKITNDGDNIKVGDKD	537
Adhesin B Genotype 2	CP017538 (2b) locus BG586_02710	841	EGNITANTSELGNNEDGTVKAPTQPNALLIAQTVADAVNNAGFNIKSAGNKAAGDQAATKLVKTGEEVVFEAGDNLTVKRDGNQFTFATAKDVSFNSVQFSENGPKITNDGDNIKVGDKD	960
	CP017491 (2c) locus BG598_01795	841	EGNITANTSELGNNEDGTVKAPTQPNALLIAQTVADAVNNAGFNIKSAGNKAAGDQAATKLVKTGEEVVFEAGDNLTVKRDGNQFTFATAKDVSFNSVQFSENGPKITNDGDNIKVGDKD	960
	CP017505 (2d) locus BG605_01790	841	EGNITANTSELGNNEDGTVKAPTQPNALLIAQTVADAVNNAGFNIKSAGNKAAGDQAATKLVKTGEEVVFEAGDNLTVKRDGNQFTFATAKDVSFNSVQFSENGPKITNDGDNIKVGDKD	960
	CP017552 (2e) locus BG607_01805	841	EGNITANTSELGNNEDGTVKAPTQPNALLIAQTVADAVNNAGFNIKSAGNKAAGDQAATKLVKTGEEVVFEAGDNLTVKRDGNQFTFATAKDVSFNSVQFSENGPKITNDGDNIKVGDKD	960

Group	Sequence	Start	Alignment	End
Adhesin B2 Genotype 1	CP017495 (1b) locus BG548_01650	538	GKPTKITNVADGDISPVSTDVINGKQLNNYAKVNGNNIGTDEDGSINIVNGNGTTITSDKAGEVKVNVNSTDLTVADNGKINVQDPNGTGSHFVNATTVANAVNNVSWNVDSKAVGTGVV	657
	CP017510 (1c) locus BG556_01650	538	GKPTKITNVADGDISPVSTDVINGKQLNNYAKVNGNNIGTDEDGSINIVNGNGTTITSDKAGEVKVNVNSTDLTVADNGKINVQDPNGTGSHFVNATTVANAVNNVSWNVDSKAVGTGVV	657
	CP017502 (1e) locus BG561_01650	538	GKPTKITNVADGDISPVSTDVINGKQLNNYAKVNGNNIGTDEDGSINIVNGNGTTITSDKAGEVKVNVNSTDLTVADNGKINVQDPNGTGSHFVNATTVANAVNNVSWNVDSKAVGTGVV	657
	CP017484 (1f) locus BG572_ 01650	538	GKPTKITNVADGDISPVSTDVINGKQLNNYAKVNGNNIGTDEDGSINIVNGNGTTITSDKAGEVKVNVNSTDLTVADNGKINVQDPNGTGSHFVNATTVANAVNNVSWNVDSKAVGTGVV	657
	CP017499 (1i) locus BG576_11180	538	GKPTKITNVADGDISPVSTDVINGKQLNNYAKVNGNNIGTDEDGSINIVNGNGTTITSDKAGEVKVNVNSTDLTVADNGKINVQDPNGTGSHFVNATTVANAVNNVSWNVDSKAVGTGVV	657
Adhesin B Genotype 2	CP017538 (2b) locus BG586_02710	961	GKPTKITNVADGDISPVSTDVINGKQLNNYAKVNGNNIGTDEDGSINIVNGNGTTITSDKAGEVKVNVNITDLTVADNGKINVQDPNGTGSRFVNATTVANAVNNVSWNVDSKAVGTGAV	1080
	CP017491 (2c) locus BG598_01795	961	GKPTKITNVADGDISPVSTDVINGKQLNNYAKVNGNNIGTDEDGSINIVNGNGTTITSDKAGEVKVNVNITDLTVADNGKINVQDPNGTGSRFVNATTVANAVNNVSWNVDSKAVGTGAV	1080
	CP017505 (2d) locus BG605_01790	961	GKPTKITNVADGDISPVSTDVINGKQLNNYAKVNGNNIGTDEDGSINIVNGNGTTITSDKAGEVKVNVNITDLTVADNGKINVQDPNGTGSRFVNATTVANAVNNVSWNVDSKAVGTGAV	1080
	CP017552 (2e) locus BG607_01805	961	GKPTKITNVADGDISPVSTDVINGKQLNNYAKVNGNNIGTDEDGSINIVNGNGTTITSDKAGEVKVNVNITDLTVADNGKINVQDPNGTGSRFVNATTVANAVNNVSWNVDSKAVGTGAV	1080

Group	Sequence	Start	Alignment	End
Adhesin B2 Genotype 1	CP017495 (1b) locus BG548_01650	658	EGDKAPAKVKAGSTVSVNAGNNIKVTRKGSDVTVAVSDTPEFTSVKTGDTLVNNNGVTINNGSAGKAVSLTKDGLNNGGNRITNVKAGEADTDAVNVGQLKGAVNHLNNKIHRNNREARA	777
	CP017510 (1c) locus BG556_01650	658	EGDKAPAKVKAGSTVSVNAGNNIKVTRKGSDVTVAVSDTPEFTSVKTGDTLVNNNGVTINNGSAGKAVSLTKDGLNNGGNRITNVKAGEADTDAVNVGQLKGAVNHLNNKIHRNNREARA	777
	CP017502 (1e) locus BG561_01650	658	EGDKAPAKVKAGSTVSVNAGNNIKVTRKGSDVTVAVSDTPEFTSVKTGDTLVNNNGVTINNGSAGKAVSLTKDGLNNGGNRITNVKAGEADTDAVNVGQLKGAVNHLNNKIHRNNREARA	777
	CP017484 (1f) locus BG572_ 01650	658	EGDKAPAKVKAGSTVSVNAGNNIKVTRKGSDVTVAVSDTPEFTSVKTGDTLVNNNGVTINNGSAGKAVSLTKDGLNNGGNRITNVKAGEADTDAVNVGQLKGAVNHLNNKIHRNNREARA	777
	CP017499 (1i) locus BG576_11180	658	EGDKAPAKVKAGSTVSVNAGNNIKVTRKGSDVTVAVSDTPEFTSVKTGDTLVNNNGVTINNGSAGKAVSLTKDGLNNGGNRITNVKAGEADTDAVNVGQLKGAVNHLNNKIHRNNREARA	777
Adhesin B Genotype 2	CP017538 (2b) locus BG586_02710	1081	EGDKAPAKVKAGSTVSVNAGNNIKVTRKGSDVTVAVSDTPEFTS-KTGDTLVNNNGVTINNGSAGKAVSLTKDGLNNGGNRITNVKAGEADTDAVNVGQLKGAVNHLNNKIHRNNREARA	1199
	CP017491 (2c) locus BG598_01795	1081	EGDKAPAKVKAGSTVSVNAGNNIKVTRKGSDVTVAVSDTPEFTS-KTGDTLVNNNGVTINNGSAGKAVSLTKDGLNNGGNRITNVKAGEADTDAVNVGQLKGAVNHLNNKIHRNNREARA	1199
	CP017505 (2d) locus BG605_01790	1081	EGDKAPAKVKAGSTVSVNAGNNIKVTRKGSDVTVAVSDTPEFTS-KTGDTLVNNNGVTINNGSAGKAVSLTKDGLNNGGNRITNVKAGEADTDAVNVGQLKGAVNHLNNKIHRNNREARA	1199
	CP017552 (2e) locus BG607_01805	1081	EGDKAPAKVKAGSTVSVNAGNNIKVTRKGSDVTVAVSDTPEFTS-KTGDTLVNNNGVTINNGSAGKAVSLTKDGLNNGGNRITNVKAGEADTDAVNVGQLKGAVNHLNNKIHRNNREARA	1199

Group	Sequence	Start	Alignment	End
Adhesin B2 Genotype 1	CP017495 (1b) locus BG548_01650	778	GIAGSN-AAALPQVYIPGKSMVAAAGGTFKGENALAVGYSRSSDNGKLILKLQGNANSRGDFGGGVGVGYQW*	849
	CP017510 (1c) locus BG556_01650	778	GIAGSN-AAALPQVYIPGKSMVAAAGGTFKGENALAVGYSRSSDNGKLILKLQGNANSRGDFGGGVGVGYQW*	849
	CP017502 (1e) locus BG561_01650	778	GIAGSN-AAALPQVYIPGKSMVAAAGGTFKGENALAVGYSRSSDNGKLILKLQGNANSRGDFGGGVGVGYQW*	849
	CP017484 (1f) locus BG572_ 01650	778	GIAGSN-AAALPQVYIPGKSMVAAAGGTFKGENALAVGYSRSSDNGKLILKLQGNANSRGDFGGGVGVGYQW*	849
	CP017499 (1i) locus BG576_11180	778	GIAGSN-AAALPQVYIPGKSMVAAAGGTFKGENALAVGYSRSSDNGKLILKLQGNANSRGDFGGGVGVGYQW*	849
Adhesin B Genotype 2	CP017538 (2b) locus BG586_02710	1200	GIAGSNAAAALPQVYIPGKSMVAAAGGTFKGENALAVGYSRSSDNGKLILKLQGNANSRGDFGGGVGVGYQW	1271
	CP017491 (2c) locus BG598_01795	1200	GIAGSNAAAALPQVYIPGKSMVAAAGGTFKGENALAVGYSRSSDNGKLILKLQGNANSRGDFGGGVGVGYQW	1271
	CP017505 (2d) locus BG605_01790	1200	GIAGSNAAAALPQVYIPGKSMVAAAGGTFKGENALAVGYSRSSDNGKLILKLQGNANSRGDFGGGVGVGYQW	1271
	CP017552 (2e) locus BG607_01805	1200	GIAGSNAAAALPQVYIPGKSMVAAAGGTFKGENALAVGYSRSSDNGKLILKLQGNANSRGDFGGGVGVGYQW	1271
