# Supplementary material for: Differences between predicted outer membrane proteins of genotype 1 and 2 Mannheimia haemolytica
Source: BMC Microbiol. 2020 Aug 12;20:250. doi: 10.1186/s12866-020-01932-2 (PMC7424683; doi:10.1186/s12866-020-01932-2)

Fig S10

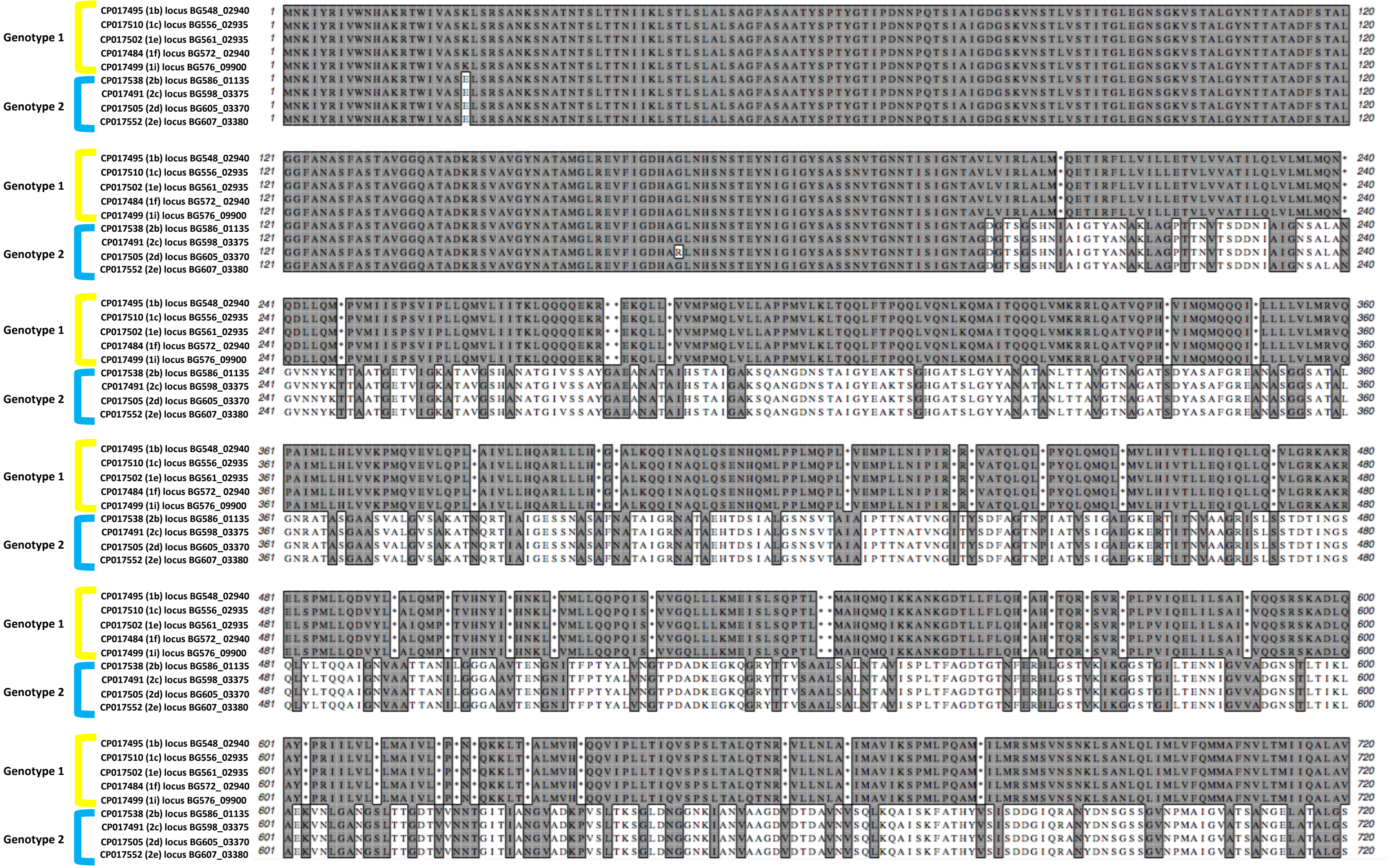
Genotype 1
CP017495 (1b) locus BG548_02940
CP017510 (1c) locus BG556_02935
CP017502 (1e) locus BG561_02935
CP017484 (1f) locus BG572_02940
CP017499 (1i) locus BG576_09900
Genotype 2
CP017538 (2b) locus BG586_01135
CP017491 (2c) locus BG598_03375
CP017505 (2d) locus BG605_03370
CP017552 (2e) locus BG607_03380

Fig S10 continued

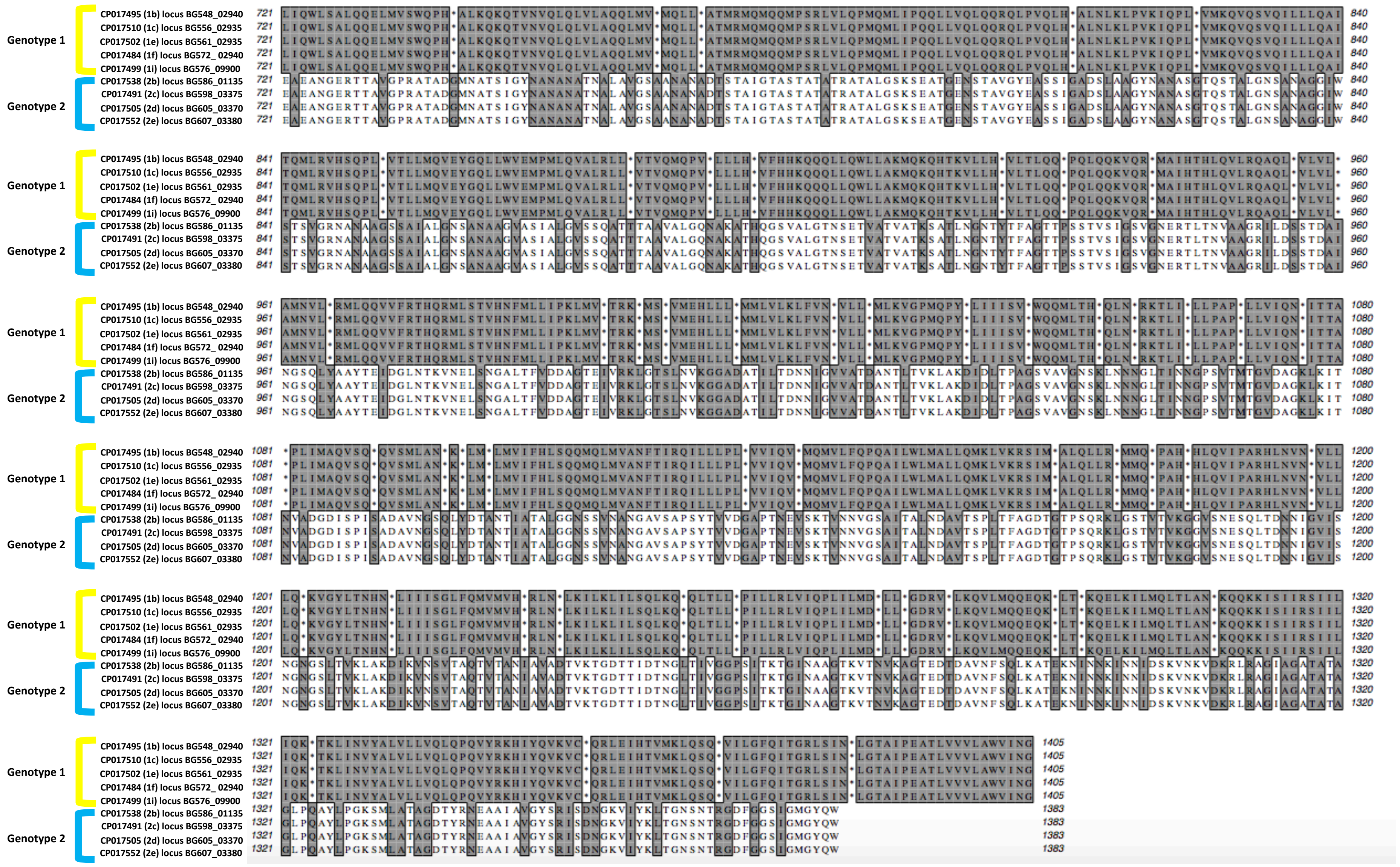

Supplement: Supplementary file 15 — Additional file 15: Figure S10. Alignment adhesin D isoforms from five genotype 1 strains and four genotype 2 strains that are each of a different subtype. The alignment contains adhesin D isoforms from five genotype 1 strains and four genotype 2 strains that are each of a different subtype. Areas of 51% chemical identity or greater are indicated with grey boxes. [file 12866_2020_1932_MOESM15_ESM.pdf]
