# Supplementary material for: Differences between predicted outer membrane proteins of genotype 1 and 2 Mannheimia haemolytica
Source: BMC Microbiol. 2020 Aug 12;20:250. doi: 10.1186/s12866-020-01932-2 (PMC7424683; doi:10.1186/s12866-020-01932-2)

Fig S11

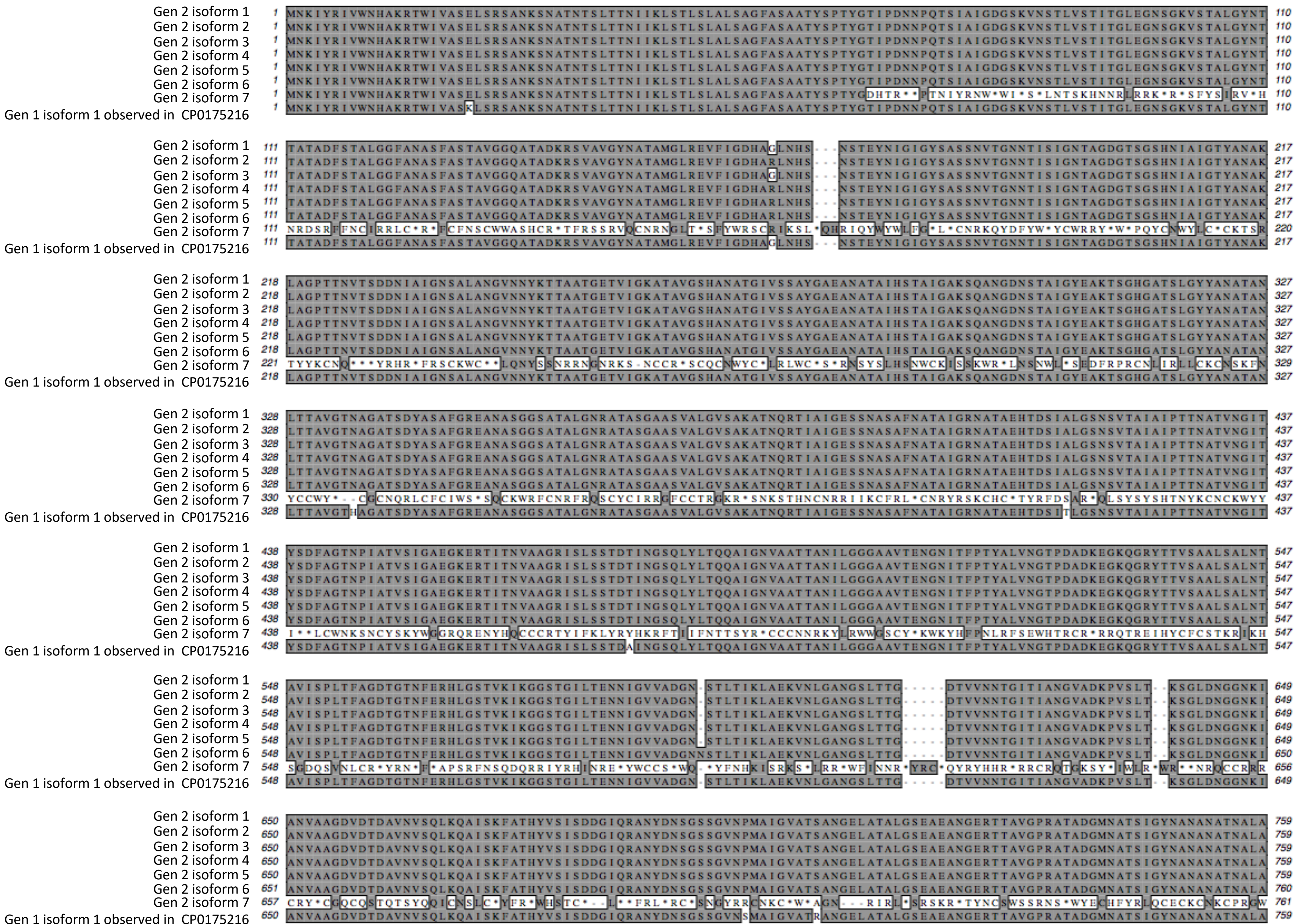
Gen 2 isoform 1
Gen 2 isoform 2
Gen 2 isoform 3
Gen 2 isoform 4
Gen 2 isoform 5
Gen 2 isoform 6
Gen 2 isoform 7
Gen 1 isoform 1 observed in CP0175216

Fig S11 continued

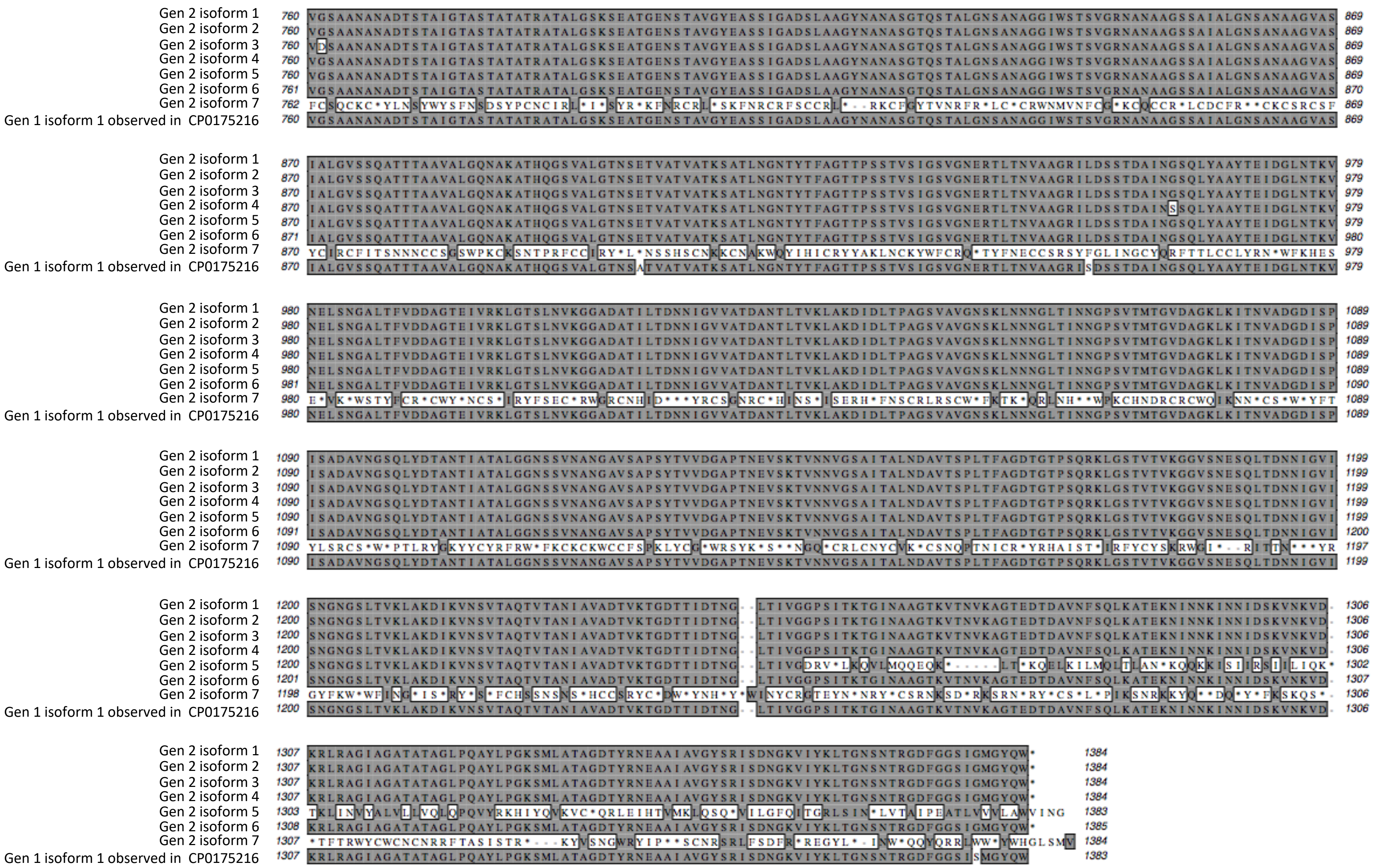

Supplement: Supplementary file 16 — Additional file 16: Figure S11. Alignment of adhesin D isoforms observed in genotype 2 M. haemolytica strains and one genotype 1 strain. The alignment contains adhesin D isoforms observed in genotype 2 M. haemolytica strains and one genotype 1 strain. Areas of 51% chemical identity or greater are indicated with grey boxes. [file 12866_2020_1932_MOESM16_ESM.pdf]
