# Supplementary material for: Differences between predicted outer membrane proteins of genotype 1 and 2 Mannheimia haemolytica
Source: BMC Microbiol. 2020 Aug 12;20:250. doi: 10.1186/s12866-020-01932-2 (PMC7424683; doi:10.1186/s12866-020-01932-2)
Supplement: Supplementary file 17 — Additional file 17: Figure S12. Alignment of adhesin B isoforms observed in genotype 2 M. haemolytica. The alignment contains adhesin B isoforms observed in genotype 2 M. haemolytica. Within it, the arrow points to the site where a single genotype 2 strain (GenBank# CP017521) has a stop codon consistent with genotype 1 strains. Prior to the stop codon, the adhesin B sequence for GenBank# CP017521 is an exact match with Gen 2 isoform 1. Areas of 51% chemical identity or greater are indicated with grey boxes. [file 12866_2020_1932_MOESM17_ESM.pdf]

Fig S12

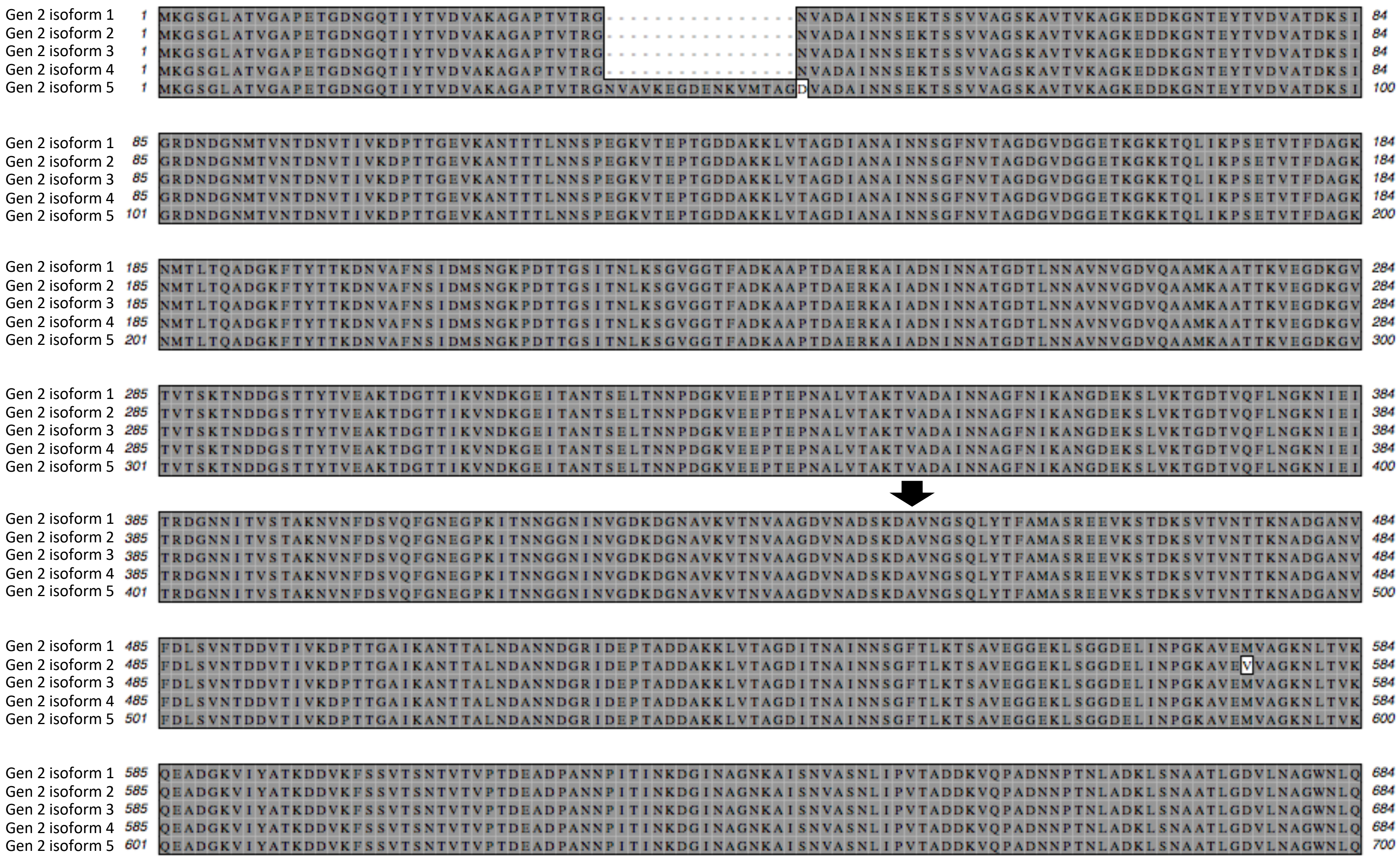

Gen 2 isoform 1 1 MKGSGLATVGAPETGDNGQTIYTVDVAKAGAPTVTRG................NVADAINNSEKTSSVVAGSKAVTVKAGKEDDKGNTEYTVDVATDKSI 84
Gen 2 isoform 2 1 MKGSGLATVGAPETGDNGQTIYTVDVAKAGAPTVTRG................NVADAINNSEKTSSVVAGSKAVTVKAGKEDDKGNTEYTVDVATDKSI 84
Gen 2 isoform 3 1 MKGSGLATVGAPETGDNGQTIYTVDVAKAGAPTVTRG................NVADAINNSEKTSSVVAGSKAVTVKAGKEDDKGNTEYTVDVATDKSI 84
Gen 2 isoform 4 1 MKGSGLATVGAPETGDNGQTIYTVDVAKAGAPTVTRG................NVADAINNSEKTSSVVAGSKAVTVKAGKEDDKGNTEYTVDVATDKSI 84
Gen 2 isoform 5 1 MKGSGLATVGAPETGDNGQTIYTVDVAKAGAPTVTRGNVAVKEGDENKVMTAGDVADAINNSEKTSSVVAGSKAVTVKAGKEDDKGNTEYTVDVATDKSI 100
Gen 2 isoform 1 85 GRDNDGNMTVNTDNVTIVKDPTTGEVKANTTTLNNSPEGKVTEPTGDDAKKLVTAGDIANAINNSGFNVTAGDGVDGGETKGKKTQLIKPSETVTFDAGK 184
Gen 2 isoform 2 85 GRDNDGNMTVNTDNVTIVKDPTTGEVKANTTTLNNSPEGKVTEPTGDDAKKLVTAGDIANAINNSGFNVTAGDGVDGGETKGKKTQLIKPSETVTFDAGK 184
Gen 2 isoform 3 85 GRDNDGNMTVNTDNVTIVKDPTTGEVKANTTTLNNSPEGKVTEPTGDDAKKLVTAGDIANAINNSGFNVTAGDGVDGGETKGKKTQLIKPSETVTFDAGK 184
Gen 2 isoform 4 85 GRDNDGNMTVNTDNVTIVKDPTTGEVKANTTTLNNSPEGKVTEPTGDDAKKLVTAGDIANAINNSGFNVTAGDGVDGGETKGKKTQLIKPSETVTFDAGK 184
Gen 2 isoform 5 101 GRDNDGNMTVNTDNVTIVKDPTTGEVKANTTTLNNSPEGKVTEPTGDDAKKLVTAGDIANAINNSGFNVTAGDGVDGGETKGKKTQLIKPSETVTFDAGK 200
Gen 2 isoform 1 185 NMTLTQADGKFTYTTKDNVAFNSIDMSNGKPDTTGSITNLKSGVGGTFADKAAPTDAERKAIADNINNATGDTLNNAVNVGDVQAAMKAATTKVEGDKGV 284
Gen 2 isoform 2 185 NMTLTQADGKFTYTTKDNVAFNSIDMSNGKPDTTGSITNLKSGVGGTFADKAAPTDAERKAIADNINNATGDTLNNAVNVGDVQAAMKAATTKVEGDKGV 284
Gen 2 isoform 3 185 NMTLTQADGKFTYTTKDNVAFNSIDMSNGKPDTTGSITNLKSGVGGTFADKAAPTDAERKAIADNINNATGDTLNNAVNVGDVQAAMKAATTKVEGDKGV 284
Gen 2 isoform 4 185 NMTLTQADGKFTYTTKDNVAFNSIDMSNGKPDTTGSITNLKSGVGGTFADKAAPTDAERKAIADNINNATGDTLNNAVNVGDVQAAMKAATTKVEGDKGV 284
Gen 2 isoform 5 201 NMTLTQADGKFTYTTKDNVAFNSIDMSNGKPDTTGSITNLKSGVGGTFADKAAPTDAERKAIADNINNATGDTLNNAVNVGDVQAAMKAATTKVEGDKGV 300
Gen 2 isoform 1 285 TVTSKTNDDGSTTYTVEAKTDGTTIKVNDKGEITANTSELTNNPDGKVEEPTEPNALVTAKTVADAINNAGFNIKANGDEKSLVKTGDTVQFLNGKNIEI 384
Gen 2 isoform 2 285 TVTSKTNDDGSTTYTVEAKTDGTTIKVNDKGEITANTSELTNNPDGKVEEPTEPNALVTAKTVADAINNAGFNIKANGDEKSLVKTGDTVQFLNGKNIEI 384
Gen 2 isoform 3 285 TVTSKTNDDGSTTYTVEAKTDGTTIKVNDKGEITANTSELTNNPDGKVEEPTEPNALVTAKTVADAINNAGFNIKANGDEKSLVKTGDTVQFLNGKNIEI 384
Gen 2 isoform 4 285 TVTSKTNDDGSTTYTVEAKTDGTTIKVNDKGEITANTSELTNNPDGKVEEPTEPNALVTAKTVADAINNAGFNIKANGDEKSLVKTGDTVQFLNGKNIEI 384
Gen 2 isoform 5 301 TVTSKTNDDGSTTYTVEAKTDGTTIKVNDKGEITANTSELTNNPDGKVEEPTEPNALVTAKTVADAINNAGFNIKANGDEKSLVKTGDTVQFLNGKNIEI 400
Gen 2 isoform 1 385 TRDGNNITVSTAKNVNFDSVQFGNEGPKITNNGGNINVGDKDGNAVKVTNVAAGDVNADSKDAVNGSQLYTFAMASREEVKSTDKSVTVNTTKNADGANV 484
Gen 2 isoform 2 385 TRDGNNITVSTAKNVNFDSVQFGNEGPKITNNGGNINVGDKDGNAVKVTNVAAGDVNADSKDAVNGSQLYTFAMASREEVKSTDKSVTVNTTKNADGANV 484
Gen 2 isoform 3 385 TRDGNNITVSTAKNVNFDSVQFGNEGPKITNNGGNINVGDKDGNAVKVTNVAAGDVNADSKDAVNGSQLYTFAMASREEVKSTDKSVTVNTTKNADGANV 484
Gen 2 isoform 4 385 TRDGNNITVSTAKNVNFDSVQFGNEGPKITNNGGNINVGDKDGNAVKVTNVAAGDVNADSKDAVNGSQLYTFAMASREEVKSTDKSVTVNTTKNADGANV 484
Gen 2 isoform 5 401 TRDGNNITVSTAKNVNFDSVQFGNEGPKITNNGGNINVGDKDGNAVKVTNVAAGDVNADSKDAVNGSQLYTFAMASREEVKSTDKSVTVNTTKNADGANV 500
Gen 2 isoform 1 485 FDLSVNTDDVTIVKDPTTGAIKANTTALNDANNDGRIDEPTADDAKKLVTAGDITNAINNSGFTLKTSAVEGGEKLSGGDELINPGKAVEMVAGKNLTVK 584
Gen 2 isoform 2 485 FDLSVNTDDVTIVKDPTTGAIKANTTALNDANNDGRIDEPTADDAKKLVTAGDITNAINNSGFTLKTSAVEGGEKLSGGDELINPGKAVEVVAGKNLTVK 584
Gen 2 isoform 3 485 FDLSVNTDDVTIVKDPTTGAIKANTTALNDANNDGRIDEPTADDAKKLVTAGDITNAINNSGFTLKTSAVEGGEKLSGGDELINPGKAVEMVAGKNLTVK 584
Gen 2 isoform 4 485 FDLSVNTDDVTIVKDPTTGAIKANTTALNDANNDGRIDEPTADDAKKLVTAGDITNAINNSGFTLKTSAVEGGEKLSGGDELINPGKAVEMVAGKNLTVK 584
Gen 2 isoform 5 501 FDLSVNTDDVTIVKDPTTGAIKANTTALNDANNDGRIDEPTADDAKKLVTAGDITNAINNSGFTLKTSAVEGGEKLSGGDELINPGKAVEMVAGKNLTVK 600
Gen 2 isoform 1 585 QEADGKVIYATKDDVKFSSVTSNTVTVPTDEADPANNPITINKDGINAGNKAISNVASNLIPVTADDKVQPADNNPTNLADKLSNAATLGDVLNAGWNLQ 684
Gen 2 isoform 2 585 QEADGKVIYATKDDVKFSSVTSNTVTVPTDEADPANNPITINKDGINAGNKAISNVASNLIPVTADDKVQPADNNPTNLADKLSNAATLGDVLNAGWNLQ 684
Gen 2 isoform 3 585 QEADGKVIYATKDDVKFSSVTSNTVTVPTDEADPANNPITINKDGINAGNKAISNVASNLIPVTADDKVQPADNNPTNLADKLSNAATLGDVLNAGWNLQ 684
Gen 2 isoform 4 585 QEADGKVIYATKDDVKFSSVTSNTVTVPTDEADPANNPITINKDGINAGNKAISNVASNLIPVTADDKVQPADNNPTNLADKLSNAATLGDVLNAGWNLQ 684
Gen 2 isoform 5 601 QEADGKVIYATKDDVKFSSVTSNTVTVPTDEADPANNPITINKDGINAGNKAISNVASNLIPVTADDKVQPADNNPTNLADKLSNAATLGDVLNAGWNLQ 700

Fig S12 continued

```
Gen 2 isoform 1   685 GNGKAVDTVVHNDTVDFINGKGTTVTVENKDGKNTIKVDSPIEFVNQDPTDSSTPSNTAKFTGEAPVQLGNVASSVRNEDGSTPEGKDRAEAIKNAEGDK 784
Gen 2 isoform 2   685 GNGKAVDTVVHNDTVDFINGKGTTVTVENKDGKNTIKVDSPIEFVNQDPTDSSTPSNTAKFTGEAPVQLGNVASSVRNEDGSTPEGKDRAEAIKNAEGDK 784
Gen 2 isoform 3   685 GNGKAVDTVVHNDTVDFINGKGTTVTVENKDGKNTIKVDSPIEFVNQDPTDSSTPSNTAKFTGEAPVQLGNVASSVRNEDGSTPEGKDRAEAIKNAEGDK 784
Gen 2 isoform 4   685 GNGKAVDTVVHNDTVDFINGKGTTVTVENKDGKNTIKVDSPIEFVNQDPTDSSTPSNTAKFTGEAPVQLGNVASSVRNEDGSTPEGKDRAEAIKNAEGDK 784
Gen 2 isoform 5   701 GNGKAVDTVVHNDTVDFINGKGTTVTVENKDGKNTIKVDSPIEFVNQDPTDSSTPSNTAKFTGEAPVQLGNVASSVRNEDGSTPEGKDRAEAIKNAEGDK 800

Gen 2 isoform 1   785 LNNVVNLGDLQAATNAATTKVGGNRGVTITPSTNADGSTTYNVEAKTDGTTIKVDNEGNITANTSELGNNEDGTVKAPTQPNALLIAQTVADAVNNAGFN 884
Gen 2 isoform 2   785 LNNVVNLGDLQAATNAATTKVGGNRGVTITPSTNADGSTTYNVEAKTDGTTIKVDNEGNITANTSELGNNEDGTVKAPTQPNALLIAQTVADAVNNAGFN 884
Gen 2 isoform 3   785 LNNVVNLGDLQAATNAATTKVGGNRGVTITPSTNADGSTTYNVEAKTDGTTIKVDNEGNITANTSELGNNEDGTVKAPTQPNALLIAQTVADAVNNAGFN 884
Gen 2 isoform 4   785 LNNVVNLGDLQAATNAATTKVGGNRGVTITPSTNADGSTTYNVEAKTDGTTIKVDNEGNITANTSELGNNEDGTVKAPTQPNALLIAQTVADAVNNAGFN 884
Gen 2 isoform 5   801 LNNVVNLGDLQAATNAATTKVGGNRGVTITPSTNADGSTTYNVEAKTDGTTIKVDNEGNITANTSELGNNEDGTVKAPTQPNALLIAQTVADAVNNAGFN 900

Gen 2 isoform 1   885 IKSAGNKAAGDQAATKLVKTGEEVVFEAGDNLTVKRDGNQFTFATAKDVSFNSVQFSENGPKITNDGDNIKVGDKDGKPTKITNVADGDISPVSTDVING 984
Gen 2 isoform 2   885 IKSAGNKAAGDQAATKLVKTGEEVVFEAGDNLTVKRDGNQFTFATAKDVSFNSVQFSENGPKITNDGDNIKVGDKDGKPTKITNVADGDISPVSTDVING 984
Gen 2 isoform 3   885 IKSAGNKAAGDQAATKLVKTGEEVVFEAGDNLTVKRDGNQFTFATAKDVSFNSVQFSENGPKITNDGDNIKVGDKDGKPTKITNVADGDISPVSTDVING 984
Gen 2 isoform 4   885 IKSAGNKAAGDQAATKLVKTGEEVVFEAGDNLTVKRDGNQFTFATAKDVSFNSVQFSENGPKITNDGDNIKVGDKDGKPTKITNVADGDISPVSTDVING 984
Gen 2 isoform 5   901 IKSAGNKAAGDQAATKLVKTGEEVVFEAGDNLTVKRDGNQFTFATAKDVSFNSVQFSENGPKITNDGDNIKVGDKDGKPTKITNVADGDISPVSTDVING 1000

Gen 2 isoform 1   985 KQLNNYAKVNGNNIGTDEDGSINIVNGNGTTITSDKAGEVKVNVNITDLTVADNGKINVQDPNGTGSRFVNATTVANAVNNVSWNVDSKAVGTGAVEGDK 1084
Gen 2 isoform 2   985 KQLNNYAKVNGNNIGTDEDGSINIVNGNGTTITSDKAGEVKVNVNITDLTVADNGKINVQDPNGTGSRFVNATTVANAVNNVSWNVDSKAVGTGAVEGDK 1084
Gen 2 isoform 3   985 KQLNNYAKVNGNNIGTDEDGSINIVNGNGTTITSDKAGEVKVNVNITDLTVADNGKINVQDPNGTGSRFVNATTVANAVNNVSWNVDSKAVGTGAVEGDK 1084
Gen 2 isoform 4   985 KQLNNYAKVNGNNIGTDEDGSINIVNGNGTTITSDKAGEVKVNVNITDLTVADNGKINVQDPNGTGSRFVNATTVANAVNNVSWNVDSKAVGTGAVEGDK 1084
Gen 2 isoform 5  1001 KQLNNYAKVNGNNIGTDEDGSINIVNGNGTTITSDKAGEVKVNVNITDLTVADNGKINVQDPNGTGSRFVNATTVANAVNNVSWNVDSKAVGTGAVEGDK 1100

Gen 2 isoform 1  1085 APAKVKAGSTVSVNAGNNIKVTRKGSDVTVAVSDTPEFTS-KTGDTLVNNNGVTINNGSAGKAVSLTKDGLNNGGNRITNVKAGEADTDAVNVGQLKGAV 1183
Gen 2 isoform 2  1085 APAKVKAGSTVSVNAGNNIKVTRKGSDVTVAVSDTPEFTS-KTGDTLVNNNGVTINNGSAGKAVSLTKDGLNNGGNRITNVKAGEADTDAVNVGQLKGAV 1183
Gen 2 isoform 3  1085 APAKVKAGSTVSVNAGNNIKVTRKGSDVTVAVSDTPEFTS-KTGDTLVNNNGVTINNGSAGKAVSLTKDGLNNGGNRITNVKAGEADTDAVNVGQLKGAV 1183
Gen 2 isoform 4  1085 APAKVKAGSTVSVNAGNNIKVTRKGSDVTVAVSDTPEFTSVKTGDTLVNNNGVTINNGSAGKAVSLTKDGLNNGGNRITNVKAGEADTDAVNVGQLKGAV 1184
Gen 2 isoform 5  1101 APAKVKAGSTVSVNAGNNIKVTRKGSDVTVAVSDTPEFTSVKTGDTLVNNNGVTINNGSAGKAVSLTKDGLNNGGNRITNVKAGEADTDAVNVGQLKGAV 1200

Gen 2 isoform 1  1184 NHLNNKIHRNNREARAGIAGSNAAAALPQVYIPGKSMVAAAGGTFKGENALAVGYSRSSDNGKLILKLQGNANSRGDFGGGVGVGYQW* 1272
Gen 2 isoform 2  1184 NHLNNKIHRNNREARAGIAGSNAAAALPQVYIPGKSMVAAAGGTFKGENALAVGYSRSSDNGKLILKLQGNANSRGDFGGGVGVGYQW* 1272
Gen 2 isoform 3  1184 NHLNNKIHRNNREARAGIAGSN-AAALPQVYIPGKSMVAAAGGTFKGENALAVGYSRSSDNGKLILKLQGNANSRGDFGGGVGVGYQW* 1271
Gen 2 isoform 4  1185 NHLNNKIHRNNREARAGIAGSNAAAALPQVYIPGKSMVAAAGGTFKGENALAVGYSRSSDNGKLILKLQGNANSRGDFGGGVGVGYQW  1272
Gen 2 isoform 5  1201 NHLNNKIHRNNREARAGIAGSNAAAALPQVYIPGKSMVAAAGGTFKGENALAVGYSRSSDNGKLILKLQGNANSRGDFGGGVGVGYQW  1288
```
