# Supplementary material for: Differences between predicted outer membrane proteins of genotype 1 and 2 Mannheimia haemolytica
Source: BMC Microbiol. 2020 Aug 12;20:250. doi: 10.1186/s12866-020-01932-2 (PMC7424683; doi:10.1186/s12866-020-01932-2)

Fig S13

```
Gen 2 isoform 1    1 MNKIFKVIFNQTTQKMEVVSELARSQGKAASSTDKRGTIKLAAASILGGATLISGMAAFMLSATPAEAAVIIDRIEVQQKAPKKVVNGGIDIYTNTSLAT 100
Gen 2 isoform 2    1 MNKIFKVIFNQTTQKMEVVSELARSQGKAASSTDKRGTIKLAAASILGGATLISGMAAFMLSATPAEAAVIIDRIEVQQKAPKKVVNGGIDIYTNTSLAT 100
Gen 2 isoform 3    1 MNKIFKVIFNQTTQKMEVVSELARSQGKAASSTDKRGTIKLAAASILGGATLISGMAAFMLSATPAEAAVIIDRIEVQQKAPKKVVNGGIDIYTNTSLAT 100
Gen 2 isoform 4    1 MNKIFKVIFNQTTQKMEVVSELARSQGKAASSTDKRGTIKLAAASILGGATLISGMAAFMLSATPAEAAVIIDRIEVQQKAPKKVVNGGIDIYTNTSLAT 100
Gen 2 isoform 5    1 MNKIFKVIFNQTTQKMEVVSELARSQGKAASSTDKRGTIKLAAASILGGATLISGMAAFMLSATPAEAAVIIDRIEVQQKAPKKVVNGGIDIYTNTSLAT 100
Gen 2 isoform 6    1 MNKIFKVIFNQTTQKMEVVSELARSQGKAASSTDKRGTIKLAAASILGGATLISGMAAFMLSATPAEAAVIIDRIEVQQKAPKKVVNGGIDIYTNTSLAT 100
Gen 2 isoform 7    1 MNKIFKVIFNQTTQKMEVVSELARSQGKAASSTDKRGTIKLAAASILGGATLISGMAAFMLSATPAEAAVIIDRIEVQQKAPKKVVNGGIDIYTNTSLAT 100
Gen 2 isoform 8    1 MNKIFKVIFNQTTQKMEVVSELARSQGKAASSTDKRGTIKLAAASILGGATLISGMAAFMLSATPAEAAVIIDRIEVQQKAPKKVVNGGIDIYTNTSLAT 100
Gen 2 isoform 9    1 MNKIFKVIFNQTTQKMEVVSELARSQGKAASSTDKRGTIKLAAASILGGATLISGMAAFMLSATPAEAAVIIDRIEVQQKAPKKVVNGGIDIYTNTSLAT 100
Gen 2 isoform 10   1 MNKIFKVIFNQTTQKMEVVSELARSQGKAASSTDKRGTIKLAAASILGGATLISGMAAFMLSATPAEAAVIIDRIEVQQKAPKKVVNGGIDIYTNTSLAT 100
Gen 2 isoform 11   1 MNKIFKVIFNQTTQKMEVVSELARSQGKAASSTDKRGTIKLAAASILGGATLISGMAAFMLSATPAEAAVIIDRIEVQQKAPKKVVNGGIDIYTNTSLAT 100
Gen 2 isoform 12   1 MNKIFKVIFNQTTQKMEVVSELARSQGKAASSTDKRGTIKLAAASILGGATLISGMAAFMLSATPAEAAVIIDRIEVQQKAPKKVVNGGIDIYTNTSLAT 100
Gen 2 isoform 13   1 MNKIFKVIFNQTTQKMEVVSELARSQGKAASSTDKRGTIKLAAASILGGATLISGMAAFMLSATPAEAAVIIDRIEVQQKAPKKVVNGGIDIYTNTSLAT 100
Gen 2 isoform 14   1 MNKIFKVIFNQTTQKMEVVSELARSQGKAASSTDKRGTIKLAAASILGGATLISGMAAFMLSATPAEAAVIIDRIEVQQKAPKKVVNGGIDIYTNTSLAT 100
Gen 2 isoform 15   1 MNKIFKVIFNQTTQKMEVVSELARSQGKAASSTDKRGTIKLAAASILGGATLISGMAAFMLSATPAEAAVIIDRIEVQQKAPKKVVNGGIDIYTNTSLAT 100
Gen 2 isoform 16   1 MNKIFKVIFNQTTQKMEVVSELARSQGKAASSTDKRGTIKLAAASILGGATLISGMAAFMLSATPAEAAVIIDRIEVQQKAPKKVVNGGIDIYTNTSLAT 100
Gen 2 isoform 17   1 MNKIFKVIFNQTTQKMEVVSELARSQGKAASSTDKRGTIKLAAASILGGATLISGMAAFMLSATPAEAAVIIDRIEVQQKAPKKVVNGGIDIYTNTSLAT 100
Gen 2 isoform 18   1 MNKIFKVIFNQTTQKMEVVSELARSQGKAASSTDKRGTIKLAAASILGGATLISGMAAFMLSATPAEAAVIIDRIEVQQKAPKKVVNGGIDIYTNTSLAT 100
Gen 2 isoform 19   1 MNKIFKVIFNQTTQKMEVVSELARSQGKAASSTDKRGTIKLAAASILGGATLISGMAAFMLSATPAEAAVIIDRIEVQQKAPKKVVNGGIDIYTNTSLAT 100
Gen 2 isoform 20   1 MNKIFKVIFNQTTQKMEVVSELARSQGKAASSTDKRGTIKLAAASILGGATLISGMAAFMLSATPAEAAVIIDRIEVQQKAPKKVVNGGIDIYTNTSLAT 100
Gen 2 isoform 21   1 MNKIFKVIFNQTTQKMEVVSELARSQGKAASSTDKRGTIKLAAASILGGATLISGMAAFMLSATPAEAAVIIDRIEVQQKAPKKVVNGGIDIYTNTSLAT 100
Gen 2 isoform 22   1 MNKIFKVIFNQTTQKMEVVSELARSQGKAASSTDKRGTIKLAAASILGGATLISGMAAFMLSATPAEAAVIIDRIEVQQKAPKKVVNGGIDIYTNTSLAT 100

Gen 2 isoform 1  101 DSITNPYNYFNPGSLSYADRTKLQESNNSVNKEKDSRLLYKSNTTSAIVIGHNAVALDNNRKSRGKPKASGIAIGDYSYSGGGLSIAIGSFSRTASIGSV 200
Gen 2 isoform 2  101 DSITNPYNYFNPGSLSYADRTKLQESNNSVNKEKDSRLLYKSNTTSAIVIGHNAVALDNNRKSRGKPKASGIAIGDYSYSGGGLSIAIGSFSRTASIGSV 200
Gen 2 isoform 3  101 DSITNLYNYFNPGSLSYADRTKLQESNNSVNKEKDSRLLYKSNTTSAIVIGHNAVALDNNRKSRGKPKASGIAIGDYSYSGGGLSIAIGSFSRTASIGSV 200
Gen 2 isoform 4  101 DSITNPYNYFNPGSLSYADRTKLQESNNSVNKEKDSRLLYKSNTTSAIVIGHNAVALDNNRKSRGKPKASGIAIGDYSYSGGGLSIAIGSFSRTASIGSV 200
Gen 2 isoform 5  101 DSITNPYNYFNPGSLSYADRTKLQESNNSVNKEKDSRLLYKSNTTSAIVIGHNAVALDNNRKSRGKPKASGIAIGDYSYSGGGLSIAIGSFSRTASIGSV 200
Gen 2 isoform 6  101 DSITNPYNYFNPGSLSYADRTKLQESNNSVNKEKDSRLLYKSNTTSAIVIGHNAVALDNNRKSRGKPKASGIAIGDYSYSGGGLSIAIGSFSRTASIGSV 200
Gen 2 isoform 7  101 DSITNPYNYFNPGSLSYADRTKLQESNNSVNKEKDSRLLYKSNTTSAIVIGHNAVALDNNRKSRGKPKASGIAIGDYSYSGGGLSIAIGSFSRTASIGSV 200
Gen 2 isoform 8  101 DSITNPYNYFNPGSLSYADRTKLQESNNSVNKEKDSRLLYKSNTTSAIVIGHNAVALDNNRKSRGKPKASGIAIGDYSYSGGGLSIAIGSFSRTASIGSV 200
Gen 2 isoform 9  101 DSITNPYNYFNPGSLSYADRTKLQESNNSVNKEKDSRLLYKSNTTSAIVIGHNAVALDNNRKSRGKPKASGIAIGDYSYSGGGLSIAIGSFSRTASIGSV 200
Gen 2 isoform 10 101 DSITNPYNYFNPGSLSYADRTKLQESNNSVNKEKDSRLLYKSNTTSAIVIGHNAVALDNNRKSRGKPKASGIAIGDYSYSGGGLSIAIGSFSRTASIGSV 200
Gen 2 isoform 11 101 DSITNPYNYFNPGSLSYADRTKLQESNNSVNKEKDSRLLYKSNTTSAIVIGHNAVALDNNRKSRGKPKASGIAIGDYSYSGGGLSIAIGSFSRTASIGSV 200
Gen 2 isoform 12 101 DSITNPYNYFNPGSLSYADRTKLQESNNSVNKEKDSRLLYKSNTTSAIVIGHNAVALDNNRKSRGKPKASGIAIGDYSYSGGGLSIAIGSFSRTASIGSV 200
Gen 2 isoform 13 101 DSITNPYNYFNPGSLSYADRTKLQESNNSVNKEKDSRLLYKSNTTSAIVIGHNAVALDNNRKSRGKPKASGIAIGDYSYSGGGLSIAIGSFSRTASIGSV 200
Gen 2 isoform 14 101 DSITNPYNYFNPGSLSYADRTKLQESNNSVNKEKDSRLLYKSNTTSAIVIGHNAVALDNNRKSRGKPKASGIAIGDYSYSGGGLSIAIGSFSRTASIGSV 200
Gen 2 isoform 15 101 DSITNPYNYFNPGSLSYADRTKLQESNNSVNKEKDSRLLYKSNTTSAIVIGHNAVALDNNRKSRGKPKASGIAIGDYSYSGGGLSIAIGSFSRTASIGSV 200
Gen 2 isoform 16 101 DSITNPYNYFNPGSLSYADRTKLQESNNSVNKEKDSRLLYKSNTTSAIVIGHNAVALDNNRKSRGKPKASGIAIGDYSYSGGGLSIAIGSFSRTASIGSV 200
Gen 2 isoform 17 101 DSITNPYNYFNPGSLSYADRTKLQESNNSVNKEKDSRLLYKSNTTSAIVIGHNAVALDNNRKSRGKPKASGIAIGDYSYSGGGLSIAIGSFSRTASIGSV 200
Gen 2 isoform 18 101 DSITNPYNYFNPGSLSYADRTKLQESNNSVNKEKDSRLLYKSNTTSAIVIGHNAVALDNNRKSREKPKASGIAIGDYSYSGGGLSIAIGSFSRTASIGSV 200
Gen 2 isoform 19 101 DSITNPYNYFNPGSLSYADRTKLQESNNSVNKEKDSRLLYKSNTTSAIVIGHNAVALDNNRKSRGKPKASGIAIGDYSYSGGGLSIAIGSFSRTASIGSV 200
Gen 2 isoform 20 101 DSITNPYNYFNPGSLSYADRTKLQESNNSVNKEKDSRLLYKSNTTSAIVIGHNAVALDNNRKSRGKPKASGIAIGDYSYSGGGLSIAIGSFSRTASIGSV 200
Gen 2 isoform 21 101 DSITNPYNYFNPGSLSYADRTKLQESNNSVNKEKDSRLLYKSNTTSAIVIGHNAVALDNNRKSRGKPKASGIAIGDYSYSGGGLSIAIGSFSRTASIGSV 200
Gen 2 isoform 22 101 DSITNPYNYFNPGSLSYADRTKLQESNNSVNKEKDSRLLYKSNTTSAIVIGHNAVALDNNRKSRGKPKASGIAIGDYSYSGGGLSIAIGSFSRTASIGSV 200

Gen 2 isoform 1  201 ALGTSARAEGFNSFAAMR------QSAAIADYSTAIGSTSWANATASFAIGASATALGDRSFAIGSGTPQTVDGSYGHENRRSIYDGQNNTLAFGTDAFS 294
Gen 2 isoform 2  201 ALGTSARAEGFNSFAAMR------QSAAIADYSTAIGSTSWANATASFAIGASATALGDRSFAIGSGTPQTVDGSYGHENRRSIYDGQNNTLAFGTDAFS 294
Gen 2 isoform 3  201 ALGTSARAEGFNSFAAMR------QSAAIADYSTAIGSTSWANATASFAIGASATALGDRSFAIGSGTPQTVDGSYGHENRRSIYDGQNNTLAFGTDAFS 294
Gen 2 isoform 4  201 ALGTSARAEGFNSFAAMR------QSAAIADYSTAIGSTSWANATASFAIGASATALGDRSFAIGSGTPQTVDGSYGHENRRSIYDGQNNTLAFGTDAFS 294
Gen 2 isoform 5  201 ALGTSARAEGFNSFAAMR------QSAAIADYSTAIGSTSWANATASFAIGASATALGDRSFAIGSGTPQTVDGSYGHENRRSIYDGQNNTLAFGTDAFS 294
Gen 2 isoform 6  201 ALGTSARAEGFNSFAAMR------QSAAIADYSTAIGSTSWANATASFAIGASATALGDRSFAIGSGTPQTVDGSYGHENRRSIYDGQNNTLAFGTDAFS 294
Gen 2 isoform 7  201 ALGTSARAEGFNSFAAMR------QSAAIADYSTAIGSTSWANATASFAIGASATALGDRSFAIGSGTPQTVDGSYGHENRRSIYDGQNNTLAFGTDAFS 294
Gen 2 isoform 8  201 ALGTSARAEGFNSFAAMR------QSAAIADYSTAIGSTSWANATASFAIGASATALGDRSFAIGSGTPQTVDGSYGHENRRSIYDGQNNTLAFGTDAFS 294
Gen 2 isoform 9  201 ALGTSARAEGFNSFAAMR------QSAAIADYSTAIGSTSWANATASFAIGASATALGDRSFAIGSGTPQTVDGSYGHENRRSIYDGQNNTLAFGTDAFS 294
Gen 2 isoform 10 201 ALGTSARAEGFNSFAAMR------QSAAIADYSTAIGSTSWANATASFAIGASATALGDRSFAIGSGTPQTVDGSYGHENRRSIYDGQNNTLAFGTDAFS 294
Gen 2 isoform 11 201 ALGTSARAEGFNSFAAMR------QSAAIADYSTAIGSTSWANATASFAIGASATALGDRSFAIGSGTPQTVDGSYGHENRRSIYDGQNNTLAFGTDAFS 294
Gen 2 isoform 12 201 ALGTSARAEGFNSFAAMR------QSAAIADYSTAIGSTSWANATASFAIGASATALGDRSFAIGSGTPQTVDGSYGHENRRSIYDGQNNTLAFGTDAFS 294
Gen 2 isoform 13 201 ALGTSARAEGFNSFAAMR------QSAAIADYSTAIGSTSWANATASFAIGASATALGDRSFAIGSGTPQTVDGSYGHENRRSIYDGQNNTLAFGTDAFS 294
Gen 2 isoform 14 201 ALGTSARAEGFNSFAAMR------QSAAIADYSTAIGSTSWANATASFAIGASATALGDRSFAIGSGTPQTVDGSYGHENRRSIYDGQNNTLAFGTDAFS 294
Gen 2 isoform 15 201 ALGTSARAEGFNSFAAMR------QSAAIADYSTAIGSTSWANATASFAIGASATALGDRSFAIGSGTPQTVDGSYGHENRRSIYDGQNNTLAFGTDAFS 294
Gen 2 isoform 16 201 ALGTSARAEGFNSFAAMR------QSAAIADYSTAIGSTSWANATASFAIGASATALGDRSFAIGSGTPQTVDGSYGHENRRSIYDGQNNTLAFGTDAFS 294
Gen 2 isoform 17 201 ALGTSARAEGFNSFAAMR------QSAAIADYSTAIGSTSWANATASFAIGASATALGDRSFAIGSGTPQTVDGSYGHENRRSIYDGQNNTLAFGTDAFS 294
Gen 2 isoform 18 201 ALGTSARAEGFNSFAAMR------QSAAIADYSTAIGSTSWANATASFAIGASATALGDRSFAIGSGTPQTVDGSYGHENRRSIYDGQNNTLAFGTDAFS 294
Gen 2 isoform 19 201 ALGTSARAEGFNSFAAMRQSAAMRQSAAIADYSTAIGSTSWANATASFAIGASATALGDRSFAIGSGTPQTVDGSYGHENRRSIYDGQNNTLAFGTDAFS 300
Gen 2 isoform 20 201 ALGTSARAEGFNSFAAMR------QSAAIADYSTAIGSTSWANATASFAIGASATALGDRSFAIGSGTPQTVDGSYGHENRRSIYDGQNNTLAFGTDAFS 294
Gen 2 isoform 21 201 ALGTSARAEGFNSFAAMR------QSAAIADYSTAIGSTSWANATASFAIGASATALGDRSFAIGSGTPQTVDGSYGHENRRSIYDGQNNTLAFGTDAFS 294
Gen 2 isoform 22 201 ALGTSARAEGFNSFAAMR------QSAAIADYSTAIGSTSWANATASFAIGASATALGDRSFAIGSGTPQTVDGSYGHENRRSIYDGQNNTLAFGTDAFS 294
```

Fig S13 continued

Gen 2 isoform 1	295	IGTKAKTNGNHSFAIGTNAWSGGFKLENSNISELGQVINPDTAKVANNAFALGTNTRANSNDTLAFSTNASATGDGSIAFGVNSNTTGIGAIAFGNASKA	394
Gen 2 isoform 2	295	IGTKAKTNGNHSFAIGTNAWSGGFKLENSNISELGQVINPDTAKVANNAFALGTNTRANSNDTLAFSTNASATGDGSIAFGVNSNTTGIGAIAFGNASKA	394
Gen 2 isoform 3	295	IGTKAKTNGNHSFAIGTNAWSGGFKLENSNISELGQVINPDTAKVANNAFALGTNTRANSNDTLAFSTNASATGDGSIAFGVNSNTTGIGAIAFGNASKA	394
Gen 2 isoform 4	295	IGTKAKTNGNHSFAIGTNAWSGGFKLENSNISELGQVINPDTAKVANNAFALGTNTRANSNDTLAFSTNASATGDGSIAFGVNSNTTGIGAIAFGNASKA	394
Gen 2 isoform 5	295	IGTKAKTNGNHSFAIGTNAWSGGFKLENSNISELGQVINPDTAKVANNAFALGTNTRANSNDTLAFSTNASATGDGSIAFGVNSNTTGIGAIAFGNASKA	394
Gen 2 isoform 6	295	IGTKAKTNGNHSFAIGTNAWSGGFKLENSNISELGQVINPDTAKVANNAFALGTNTRANSNDTLAFSTNASATGDGSIAFGVNSNTTGIGAIAFGNASKA	394
Gen 2 isoform 7	295	IGTKAKTNGNHSFAIGTNAWSGGFKLENSNISELGQVINPDTAKVANNAFALGTNTRANSNDTLAFSTNASATGDGSIAFGVNSNTTGIGAIAFGNASKA	394
Gen 2 isoform 8	295	IGTKAKTNGNHSFAIGTNAWSGGFKLENSNISELGQVINPDTAKVANNAFALGTNTRANSNDTLAFSTNASATGDGSIAFGVNSNTTGIGAIAFGNASKA	394
Gen 2 isoform 9	295	IGTKAKTNGNHSFAIGTNAWSGGFKLENSNISELGQVINPDTAKVANNAFALGTNTRANSNDTLAFSTNASATGDGSIAFGVNSNTTGIGAIAFGNASKA	394
Gen 2 isoform 10	295	IGTKAKTNGNHSFAIGTNAWSGGFKLENSNISELGQVINPDTAKVANNAFALGTNTRANSNDTLAFSTNASATGDGSIAFGVNSNTTGIGAIAFGNASKA	394
Gen 2 isoform 11	295	IGTKAKTNGNHSFAIGTNAWSGGFKLENSNISELGQVINPDTAKVANNAFALGTNTRANSNDTLAFSTNASATGDGSIAFGVNSNTTGIGAIAFGNASKA	394
Gen 2 isoform 12	295	IGTKAKTNGNHSFAIGTNAWSGGFKLENSNISELGQVINPDTAKVANNAFALGTNTRANSNDTLAFSTNASATGDGSIAFGVNSNTTGIGAIAFGNASKA	394
Gen 2 isoform 13	295	IGTKAKTNGNHSFAIGTNAWSGGFKLENSNISELGQVINPDTAKVANNAFALGTNTRANSNDTLAFSTNASATGDGSIAFGVNSNTTGIGAIAFGNASKA	394
Gen 2 isoform 14	295	IGTKAKTNGNHSFAIGTNAWSGGFKLENSNISELGQVINPDTAKVANNAFALGTNTRANSNDTLAFSTNASATGDGSIAFGVNSNTTGIGAIAFGNASKA	394
Gen 2 isoform 15	295	IGTKAKTNGNHSFAIGTNAWSGGFKLENSNISELGQVINPDTAKVANNAFALGTNTRANSNDTLAFSTNASATGDGSIAFGVNSNTTGIGAIAFGNASKA	394
Gen 2 isoform 16	295	IGTKAKTNGNHSFAIGTNAWSGGFKLENSNISELGQVINPDTAKVANNAFALGTNTRANSNDTLAFSTNASATGDGSIAFGVNSNTTGIGAIAFGNASKA	394
Gen 2 isoform 17	295	IGTKAKTNGNHSFAIGTNAWSGGFKLENSNISELGQVINPDTAKVANNAFALGTNTRANSNDTLAFSTNASATGDGSIAFGVNSNTTGIGAIAFGNASKA	394
Gen 2 isoform 18	295	IGTKAKTNGNHSFAIGTNAWSGGFKLESSNISELGQVINPDTAKVANNAFALGTNTRANSNDTLAFSTNASATGDGSIAFGVNSNTTGIGAIAFGNASKA	394
Gen 2 isoform 19	301	IGTKAKTNGNHSFAIGTNAWSGGFKLENSNISELGQVINPDTAKVANNAFALGTNTRANSNDTLAFSTNASATGDGSIAFGVNSNTTGIGAIAFGNASKA	400
Gen 2 isoform 20	295	IGTKAKTNGNHSFAIGTNAWSGGFKLENSNISELGQVINPDTAKVANNAFALGTNTRANSNDTLAFSTNASATGDGSIAFGVNSNTTGIGAIAFGNASKA	394
Gen 2 isoform 21	295	IGTKAKTNGNHSFAIGTNAWSGGFKLENSNISELGQVINPDTAKVANNAFALGTNTRANSNDTLAFSTNASATGDGSIAFGVNSNTTGIGAIAFGNASKA	394
Gen 2 isoform 22	295	IGTKAKTNGNHSFAIGTNAWSGGFKLENSNISELGQVINPDTAKVANNAFALGTNTRANSNDTLAFSTNASATGDGSIAFGVNSNTTGIGAIAFGNASKA	394

Gen 2 isoform 1	395	WKSNAISIGWNANSHAEKVISIGSHSNASKDGAMAIGDTANSSGNNSVAIGYSANVSKANATAVGTRANITHEGGTAIGFEAKSKGNYATAIAYNSTAKG	494
Gen 2 isoform 2	395	WKSNAISIGWNANSHAEKVISIGSHSNASKDGAMAIGDTANSSGNNSVAIGYSANVSKANATAVGTRANITHEGGTAIGFEAKSKGNYATAIAYNSTAKG	494
Gen 2 isoform 3	395	WKSNAISIGWNANSHAEKVISIGSHSNASKDGAMAIGDTANSSGNNSVAIGYSANVSKANATAVGTRANITHEGGTAIGFEAKSKGNYATAIAYNSTAKG	494
Gen 2 isoform 4	395	WKSNAISIGWNANSHAEKVISIGSHSNASKDGAMAIGDTANSSGNNSVAIGYSANVSKANATAVGTRANITHEGGTAIGFEAKSKGNYATAIAYNSTAKG	494
Gen 2 isoform 5	395	WKSNAISIGWNANSHAEKVISIGSHSNASKDGAMAIGDTANSSGNNSVAIGYSANVSKANATAVGTRANITHEGGTAIGFEAKSKGNYATAIAYNSTAKG	494
Gen 2 isoform 6	395	WKSNAISIGWNANSHAEKVISIGSHSNASKDGAMAIGDTANSSGNNSVAIGYSANVSKANATAVGTRANITHEGGTAIGFEAKSKGNYATAIAYNSTAKG	494
Gen 2 isoform 7	395	WKSNAISIGWNANSHAEKVISIGSHSNASKDGAMAIGDTANSSGNNSVAIGYSANVSKANATAVGTRANITHEGGTAIGFEAKSKGNYATAIAYNSTAKG	494
Gen 2 isoform 8	395	WKSNAISIGWNANSHAEKVISIGSHSNASKDGAMAIGDTANSSGNNSVAIGYSANVSKANATAVGTRANITHEGGTAIGFEAKSKGNYATAIAYNSTAKG	494
Gen 2 isoform 9	395	WKSNAISIGWNANSHAEKVISIGSHSNASKDGAMAIGDTANSSGNNSVAIGYSANVSKANATAVGTRANITHEGGTAIGFEAKSKGNYATAIAYNSTAKG	494
Gen 2 isoform 10	395	WKSNAISIGWNANSHAEKVISIGSHSNASKDGAMAIGDTANSSGNNSVAIGYSANVSKANATAVGTRANITHEGGTAIGFEAKSKGNYATAIAYNSTAKG	494
Gen 2 isoform 11	395	WKSNAISIGWNANSHAEKVISIGSHSNASKDGAMAIGDTANSSGNNSVAIGYSANVSKANATAVGTRANITHEGGTAIGFEAKSKGNYATAIAYNSTAKG	494
Gen 2 isoform 12	395	WKSNAISIGWNANSHAEKVISIGSHSNASKDGAMAIGDTANSSGNNSVAIGYSANVSKANATAVGTRANITHEGGTAIGFEAKSKGNYATAIAYNSTAKG	494
Gen 2 isoform 13	395	WKSNAISIGWNANSHAEKVISIGSHSNASKDGAMAIGDTANSSGNNSVAIGYSANVSKANATAVGTRANITHEGGTAIGFEAKSKGNYATAIAYNSTAKG	494
Gen 2 isoform 14	395	WKSNAISIGWNANSHAEKVISIGSHSNASKDGAMAIGDTANSSGNNSVAIGYSANVSKANATAVGTRANITHEGGTAIGFEAKSKGNYATAIAYNSTAKG	494
Gen 2 isoform 15	395	WKSNAISIGWNANSHAEKVISIGSHSNASKDGAMAIGDTANSSGNNSVAIGYSANVSKANATAVGTRANITHEGGTAIGFEAKSKGNYATAIAYNSTAKG	494
Gen 2 isoform 16	395	WKSNAISIGWNANSHAEKVISIGSHSNASKDGAMAIGDTANSSGNNSVAIGYSANVSKANATAVGTRANITHEGGTAIGFEAKSKGNYATAIAYNSTAKG	494
Gen 2 isoform 17	395	WKSNAISIGWNANSHAEKVISIGSHSNASKDGAMAIGDTANSSGNNSVAIGYSANVSKANATAVGTRANITHEGGTAIGFEAKSKGNYATAIAYNSTAKG	494
Gen 2 isoform 18	395	WKSNAISIGWNANSHAEKVISIGSHSNASKDGAMAIGDTANSSGNNSVAIGYSANVSKANATAVGTRANITHEGGTAIGFEAKSKGNYATAIAYNSTAKG	494
Gen 2 isoform 19	401	WKSNAISIGWNANSHAEKVISIGSHSNASKDGAMAIGDTANSSGNNSVAIGYSANVSKANATAVGTRANITHEGGTAIGFEAKSKGNYATAIAYNSTAKG	500
Gen 2 isoform 20	395	WKSNAISIGWNANSHAEKVISIGSHSNASKDGAMAIGDTANSSGNNSVAIGYSANVSKANATAVGTRANITHEGGTAIGFEAKSKGNYATAIAYNSTAKG	494
Gen 2 isoform 21	395	WKSNAISIGWNANSHAEKVISIGSHSNASKDGAMAIGDTANSSGNNSVAIGYSANVSKANATAVGTRANITHEGGTAIGFEAKSKGNYATAIAYNSTAKG	494
Gen 2 isoform 22	395	WKSNAISIGWNANSHAEKVISIGSHSNASKDGAMAIGDTANSSGNNSVAIGYSANVSKANATAVGTRANITHEGGTAIGFEAKSKGNYATAIAYNSTAKG	494

Gen 2 isoform 1	495	KNSIAAGVNSTVSGIRAAALGYNNTVSGKDTFVLGSNVTVKTKGSVILGSDSTEATATKEDTATVGTLKYSSFAGDENVVDGDYVSVGNATNARQIKFVA	594
Gen 2 isoform 2	495	KNSIAAGVNSTVSGIRAAALGYNNTVSGKDTFVLGSNVTVKTKGSVILGSDSTEATATKEDTATVGTLKYSSFAGDENVVDGDYVSVGNATNARQIKFVA	594
Gen 2 isoform 3	495	KNSIAAGVNSTVSGIRAAALGYNNTVSGKDTFVLGSNVTVKTKGSVILGSDSTEATATKEDTATVGTLKYSSFAGDENVVDGDYVSVGNATNARQIKFVA	594
Gen 2 isoform 4	495	KNSIAAGVNSTVSGIRAAALGYNNTVSGKDTFVLGSNVTVKTKGSVILGSDSTEATATKEDTATVGTLKYSSFAGDENVVDGDYVSVGNATNARQIKFVA	594
Gen 2 isoform 5	495	KNSIAAGVNSTVSGIRAAALGYNNTVSGKDTFVLGSNVTVKTKGSVILGSDSTEATATKEDTATVGTLKYSSFAGDENVVDGDYVSVGNATNARQIKFVA	594
Gen 2 isoform 6	495	KNSIAAGVNSTVSGIRAAALGYNNTVSGKDTFVLGSNVTVKTKGSVILGSDSTEATATKEDTATVGTLKYSSFAGDENVVDGDYVSVGNATNARQIKFVA	594
Gen 2 isoform 7	495	KNSIAAGVNSTVSGIRAAALGYNNTVSGKDTFVLGSNVTVKTKGSVILGSDSTEATATKEDTATVGTLKYSSFAGDENVVDGDYVSVGNATNARQIKFVA	594
Gen 2 isoform 8	495	KNSIAAGVNSTVSGIRAAALGYNNTVSGKDTFVLGSNVTVKTKGSVILGSDSTEATATKEDTATVGTLKYSSFAGDENVVDGDYVSVGNATNARQIKFVA	594
Gen 2 isoform 9	495	KNSIAAGVNSTVSGIRAAALGYNNTVSGKDTFVLGSNVTVKTKGSVILGSDSTEATATKEDTATVGTLKYSSFAGDENVVDGDYVSVGNATNARQIKFVA	594
Gen 2 isoform 10	495	KNSIAAGVNSTVSGIRAAALGYNNTVSGKDTFVLGSNVTVKTKGSVILGSDSTEATATKEDTATVGTLKYSSFAGDENVVDGDYVSVGNATNARQIKFVA	594
Gen 2 isoform 11	495	KNSIAAGVNSTVSGIRAAALGYNNTVSGKDTFVLGSNVTVKTKGSVILGSDSTEATATKEDTATVGTLKYSSFAGDENVVDGDYVSVGNATNARQIKFVA	594
Gen 2 isoform 12	495	KNSIAAGVNSTVSGIRAAALGYNNTVSGKDTFVLGSNVTVKTKGSVILGSDSTEATATKEDTATVGTLKYSSFAGDENVVDGDYVSVGNATNARQIKFVA	594
Gen 2 isoform 13	495	KNSIAAGVNSTVSGIRAAALGYNNTVSGKDTFVLGSNVTVKTKGSVILGSDSTEATATKEDTATVGTLKYSSFAGDENVVDGDYVSVGNATNARQIKFVA	594
Gen 2 isoform 14	495	KNSIAAGVNSTVSGIRAAALGYNNTVSGKDTFVLGSNVTVKTKGSVILGSDSTEATATKEDTATVGTLKYSSFAGDENVVDGDYVSVGNATNARQIKFVA	594
Gen 2 isoform 15	495	KNSIAAGVNSTVSGIRAAALGYNNTVSGKDTFVLGSNVTVKTKGSVILGSDSTEATATKEDTATVGTLKYSSFAGDENVVDGDYVSVGNATNARQIKFVA	594
Gen 2 isoform 16	495	KNSIAAGVNSTVSGIRAAALGYNNTVSGKDTFVLGSNVTVKTKGSVILGSDSTEATATKEDTATVGTLKYSSFAGDENVVDGDYVSVGNATNARQIKFVA	594
Gen 2 isoform 17	495	KNSIAAGVNSTVSGIRAAALGYNNTVSGKDTFVLGSNVTVKTKGSVILGSDSTEATATKEDTATVGTLKYSSFAGDENVVDGDYVSVGNATNARQIKFVA	594
Gen 2 isoform 18	495	KNSIAAGVNSTVSGIRAAALGYNNTVSGKDTFVLGSNVTVKTKGSVILGSDSTEATATKEDTATVGTLKYSSFAGDENVVDGDYVSVGNATNARQIKFVA	594
Gen 2 isoform 19	501	KNSIAAGVNSTVSGIRAAALGYNNTVSGKDTFVLGSNVTVKTKGSVILGSDSTEATATKEDTATVGTLKYSSFAGDENVVDGDYVSVGNATNARQIKFVA	600
Gen 2 isoform 20	495	KNSIAAGVNSTVSGIRAAALGYNNTVSGKDTFVLGSNVTVKTKGSVILGSDSTEATATKEDTATVGTLKYSSFAGDENVVDGDYVSVGNATNARQIKFVA	594
Gen 2 isoform 21	495	KNSIAAGVNSTVSGIRAAALGYNNTVSGKDTFVLGSNVTVKTKGSVILGSDSTEATATKEDTATVGTLKYSSFAGDENVVDGDYVSVGNATNARQIKFVA	594
Gen 2 isoform 22	495	KNSIAAGVNSTVSGIRAAALGYNNTVSGKDTFVLGSNVTVKTKGSVILGSDSTEATATKEDTATVGTLKYSSFAGDENVVDGDYVSVGNATNARQIKFVA	594

Fig S13 continued

```
Gen 2 isoform 1   595 PGKIASNSTDAINGSQLYAVADTLVKTGFNITADHSGLGGGAKEDNVKLGETVKYTSTDKNVVTTVTNNTIDFGLSDNISVGGKPGKDGSVGVKGADGKD 694
Gen 2 isoform 2   595 PGKIASNSTDAINGSQLYAVADTLVKTGFNITADHSGLGGGAKEDNVKLGETVKYTSTDKNVVTTVTNNTIDFGLSDNISVGGKPGKDGSVGVKGADGKD 694
Gen 2 isoform 3   595 PGKIASNSTDAINGSQLYAVADTLVKTGFNITADHSGLGGGAKEDNVKLGETVKYTSTDKNVVTTVTNNTIDFGLSDNISVGGKPGKDGSVGVKGADGKD 694
Gen 2 isoform 4   595 PGKIASNSTDAINGSQLYAVADTLVKTGFNITADHSGLGGGAKEDNVKLGETVKYTSTDKNVVTTVTNNTIDFGLSDNISVGGKPGKDGSVGVKGADGKD 694
Gen 2 isoform 5   595 PGKIASNSTDAINGSQLYAVADTLVKTGFNITADHSGLGGGAKEDNVKLGETVKYTSTDKNVVTTVTNNTIDFGLSDNISVGGKPGKDGSVGVKGADGKD 694
Gen 2 isoform 6   595 PGKIASNSTDAINGSQLYAVADTLVKTGFNITADHSGLGGGAKEDNVKLGETVKYTSTDKNVVTTVTNNTIDFGLSDNISVGGKPGKDGSVGVKGADGKD 694
Gen 2 isoform 7   595 PGKIASNSTDAINGSQLYAVADTLVKTGFNITADHSGLGGGAKEDNVKLGETVKYTSTDKNVVTTVTNNTIDFGLSDNISVGGKPGKDG----------- 683
Gen 2 isoform 8   595 PGKIASNSTDAINGSQLYAVADTLVKTGFNITADHSGLGGGAKEDNVKLGETVKYTSTDKNVVTTVTNNTIDFGLSDNISVGGKPGKDGSVGVKGADGKD 694
Gen 2 isoform 9   595 PGKIASNSTDAINGSQLYAVADTLVKTGFNITADHSGLGGGAKEDNVKLGETVKYTSTDKNVVTTVTNNTIDFGLSDNISVGGKPGKDGSVGVKGADGKD 694
Gen 2 isoform 10  595 PGKIASNSTDAINGSQLYAVADTLVKTGFNITADHSGLGGGAKEDNVKLGETVKYTSTDKNVVTTVTNNTIDFGLSDNISVGGKPGKDGSVGVKGADGKD 694
Gen 2 isoform 11  595 PGKIASNSTDAINGSQLYAVADTLVKTGFNITADHSGLGGGAKEDNVKLGETVKYTSTDKNVVTTVTNNTIDFGLSDNISVGGKPGKDGSVGVKGADGKD 694
Gen 2 isoform 12  595 PGKIASNSTDAINGSQLYAVADTLVKTGFNITADHSGLGGGAKEDNVKLGETVKYTSTDKNVVTTVTNNTIDFGLSDNISVGGKPGKDGSVGVKGADGKD 694
Gen 2 isoform 13  595 PGKIASNSTDAINGSQLYAVADTLVKTGFNITADHSGLGGGAKEDNVKLGETVKYTSTDKNVVTTVTNNTIDFGLSDNISVGGKPGKDGSVGVKGADGKD 694
Gen 2 isoform 14  595 PGKIASNSTDAINGSQLYAVADTLVKTGFNITADHSGLGGGAKEDNVKLGETVKYTSTDKNVVTTVTNNTIDFGLSDNISVGGKPGKDGSVGVKGADGKD 694
Gen 2 isoform 15  595 PGKIASNSTDAINGSQLYAVADTLVKTGFNITADHSGLGGGAKEDNVKLGETVKYTSTDKNVVTTVTNNTIDFGLSDNISVGGKPGKDGSVGVKGADGKD 694
Gen 2 isoform 16  595 PGKIASNSTDAINGSQLYAVADTLVKTGFNITADHSGLGGGAKEDNVKLGETVKYTSTDKNVVTTVTNNTIDFGLSDNISVGGKPGKDGSVGVKGADGKD 694
Gen 2 isoform 17  595 PGKIASNSTDAINGSQLYAVADTLVKTGFNITADHSGLGGGAKEDNVKLGETVKYTSTDKNVVTTVTNNTIDFGLSDNISVGGKPGKDGSVGVKGADGKD 694
Gen 2 isoform 18  595 PGKIASNSTDAINGSQLYAVADTLVKTGFNITADHSGLGGGAKEDNVKLGETVKYTSTDKNVVTTVTNNTIDFGLSDNISVGGKPGKDGSVGVKGADGKD 694
Gen 2 isoform 19  601 PGKIASNSTDAINGSQLYAVADTLVKTGFNITADHSGLGGGAKEDNVKLGETVKYTSTDKNVVTTVTNNTIDFGLSDNISVGGKPGKDGSVGVKGADGKD 700
Gen 2 isoform 20  595 PGKIASNSTDAINGSQLYAVADTLVKTGFNITADHSGLGGGAKEDNVKLGETVKYTSTDKNVVTTVTNNTIDFGLSDNISVGGKPGKDGSVGVKGTDGKD 694
Gen 2 isoform 21  595 PGKIASNSTDAINGSQLYAVADTLVKTGFNITADHSGLGGGAKEDNVKLGETVKYTSTDKNVVTTVTNNTIDFGLSDNISVGGKPGKDGSVGVKGADGKD 694
Gen 2 isoform 22  595 PGKIASNSTDAINGSQLYAVADTLVKTGFNITADHSGLGGGAKEDNVKLGETVKYTSTDKNVVTTVTNNTIDFGLSDNISVGGKPGKDGSVGVKGADGKD 694
```

```
Gen 2 isoform 1   695 GVTIKPEAIVFHGVDGVNGKDGKDGKASIKVEKGAKGLDGNDGKDGESKTRIVYEKPNGDKEEVATLNDGLKFGANDGKVHDAKLNTQVDIKGATTNTDW 794
Gen 2 isoform 2   695 GVTIKPEAIVFHGVDGVNGKDGKDGKASIKVEKGAKGLDGNDGKDGESKTRIVYEKPNGDKEEVATLNDGLKFGANDGKVHDAKLNTQVDIKGATTNTDW 794
Gen 2 isoform 3   695 GVTIKPEAIVFHGVDGVNGKDGKDGKASIKVEKGAKGLDGNDGKDGESKTRIVYEKPNGDKEEVATLNDGLKFGANDGKVHDAKLNTQVDIKGATTNTDW 794
Gen 2 isoform 4   695 GVTIKPEAIVFHGVDGVNGKDG---KASIKVEKGAKGLDGNDGKDGESKTRIVYEKPNGDKEEVATLNDGLKFGANDGKVHDAKLNTQVDIKGATTNTDW 791
Gen 2 isoform 5   695 GVTIKPEAIVFHGVDGVNGKDGKDGKASIKVEKGAKGLDGNDGKDGESKTRIVYEKPNGDKEEVATLNDGLKFGANDGKVHDAKLNTQVDIKGATTNTDW 794
Gen 2 isoform 6   695 GVTIKPEAIVFHGVDGVNGKDGKDGKASIKVEKGAKGLDGNDGKDGESKTRIVYEKPNGDKEEVATLNDGLKFGANDGKVHDAKLNTQVDIKGATTNTDW 794
Gen 2 isoform 7   684 -------------------------KASIKVEKGAKGLDGNDGKDGESKTRIVYEKPNGDKEEVATLNDGLKFGANDGKVHDAKLNTQVDIKGATTNTDW 758
Gen 2 isoform 8   695 GVTIKPEAIVFHGVDGVNGKDGKDGKASIKVEKGAKGLDGNDGKDGESKTRIVYEKPNGDKEEVATLNDGLKFGANDGKVHDAKLNTQVDIKGATTNTDW 794
Gen 2 isoform 9   695 GVTIKPEAIVFHGVDGVNGKDGKDGKASIKVEKGAKGLDGNDGKDGESKTRIVYEKPNGDKEEVATLNDGLKFGANDGKVHDAKLNTQVDIKGATTNTDW 794
Gen 2 isoform 10  695 GVTIKPEAIVFHGVDGVNGKDGKDGKASIKVEKGAKGLDGNDGKDGESKTRIVYEKPNGDKEEVATLNDGLKFGANDGKVHDAKLNTQVDIKGATTNTDW 794
Gen 2 isoform 11  695 GVTIKPEAIVFHGVDGVNGKDGKDGKASIKVEKGAKGLDGNDGKDGESKTRIVYEKPNGDKEEVATLNDGLKFGANDGKVHDAKLNTQVDIKGATTNTDW 794
Gen 2 isoform 12  695 GVTIKPEAIVFHGVDGVNGKDGKDGKASIKVEKGAKGLDGNDGKDGESKTRIVYEKPNGDKEEVATLNDGLKFGANDGKVHDAKLNTQVDIKGATTNTDW 794
Gen 2 isoform 13  695 GVTIKPEAIVFHGVDGVNGKDGKDGKASIKVEKGAKGLDGNDGKDGESKTRIVYEKPNGDKEEVATLNDGLKFGANDGKVHDAKLNTQVDIKGATTNTDW 794
Gen 2 isoform 14  695 GVTIKPEAIVFHGVDGVNGKDGKDGKASIKVEKGAKGLDGNDGKDGESKTRIVYEKPNGDKEEVATLNDGLKFGANDGKVHDAKLNTQVDIKGATTNTDW 794
Gen 2 isoform 15  695 GVTIKPEAIVFHGVDGVNGKDGKDGKASIKVEKGAKGLDGNDGKDGESKTRIVYEKPNGDKEEVATLNDGLKFGANDGKVHDAKLNTQVDIKGATTNTDW 794
Gen 2 isoform 16  695 GVTIKPEAIVFHGVDGVNGKDGKDGKASIKVEKGAKGLDGNDGKDGESKTRIVYEKPNGDKEEVATLNDGLKFGANDGKVHDAKLNTQVDIKGATTNTDW 794
Gen 2 isoform 17  695 GVTIKPEAIVFHGVDGVNGKDGKDGKASIKVEKGAKGLDGNDGKDGESKTRIVYEKPNGDKEEVATLNDGLKFGANDGKVHDAKLNTQVDIKGATTNTDW 794
Gen 2 isoform 18  695 GVTIKPEAIVFHGVDGVNGKDGKDGKASIKVEKGAKGLDGNDGKDGESKTRIVYEKPNGDKEEVATLNDGLKFGANDGKVHDAKLNTQVDIKGATTNTDW 794
Gen 2 isoform 19  701 GVTIKPEAIVFHGVDGVNGKDGKDGKASIKVEKGAKGLDGNDGKDGESKTRIVYEKPNGDKEEVATLNDGLKFGANDGKVHDAKLNTQVDIKGATTNTDW 800
Gen 2 isoform 20  695 GVTIKPEAIVFHGVDGVNGKDGKDGKASIKVEKGAKGLDGNDGKDGESKTRIVYEKPNGDKEEVATLNDGLKFGANDGKVHDAKLNTQVDIKGATTNTDW 794
Gen 2 isoform 21  695 GVTIKPEAIVFHGVDGVNGKDGKDGKASIKVEKGAKGLDGNDGKDGESKTRIVYEKPNGDKEEVATLNDGLKFGANDGKV*------------------- 775
Gen 2 isoform 22  695 GVTIKPEAIVFHGVDGVNGKDGKDGKASIKVEKGAKGLDGNDGKDGESKTRIVYEKPNGDKEEVATLNDGLKFGANDGKVHDAKLNTQVDIKGATTNTDW 794
```

```
Gen 2 isoform 1   795 AKFDAGKNIMTQVVGNTITVALAKDLAGLDSATFGTQGDSTVINKDGVTIAKKDPNDATKSNVTLTEDGLSNGGNQITNVDSGLKGKDGQPVKLTEAAGD 894
Gen 2 isoform 2   795 AKFDAGKNIMTQVVGNTITVALAKDLAGLDSATFGTQGDSTVINKDGVTIAKKDPNDATKSNVTLTEDGLSNGGNQITNVDSGLKGKDGQPVKLTEAAGD 894
Gen 2 isoform 3   795 AKFDAGKNIMTQVVGNTITVALAKDLAGLDSATFGTQGDSTVINKDGVTIAKKDPNDATKSNVTLTEDGLSNGGNQITNVDSGLKGKDGQPVKLTEAAGD 894
Gen 2 isoform 4   792 AKFDAGKNIMTQVVGNTITVALAKDLAGLDSATFGTQGDSTVINKDGVTIAKKDPNDATKSNVTLTEDGLSNGGNQITNVDSGLKGKDGQPVKLTEAAGD 891
Gen 2 isoform 5   795 AKFDAGKNIMTQVVGNTITVALAKDLAGLDSATFGTQGDSTVINKDGVTIAKKDPNDATKSNVTLTEDGLSNGGNQITNVDSGLKGKDGQPVKLTEAAGD 894
Gen 2 isoform 6   795 AKFDAGKNIMTQVVGNTITVALAKDLAGLDSATFGTQGDSTVINKDGVTIAKKDPNDATKSNVTLTEDGLSNGGNQITNVDSGLKGKDGQPVKLTEAAGD 894
Gen 2 isoform 7   759 AKFDAGKNIMTQVVGNTITVALAKDLAGLDSATFGTQGDSTVINKDGVTIAKKDPNDATKSNVTLTEDGLSNGGNQITNVDSGLKGKDGQPVKLTEAAGD 858
Gen 2 isoform 8   795 AKFDAGKNIMTQVVGNTITVALAKDLAGLDSATFGTQGDSTVINKDGVTIAKKDPNDATKSNVTLTEDGLSNGGNQITNVDSGLKGKDGQPVKLTEAAGD 894
Gen 2 isoform 9   795 AKFDAGKNIMTQVVGNTITVALAKDLAGLDSATFGTQGDSTVINKDGVTIAKKDPNDATKSNVTLTEDGLSNGGNQITNVDSGLKGKDGQPVKLTEAAGD 894
Gen 2 isoform 10  795 AKFDAGKNIMTQVVGNTITVALAKDLAGLDSATFGTQGDSTVINKDGVTIAKKDPNDATKSNVTLTEDGLSNGGNQITNVDSGLKGKDGQPVKLTEAAGD 894
Gen 2 isoform 11  795 AKFDAGKNIMTQVVGNTITVALAKDLAGLDSATFGTQGDSTVINKDGVTIAKKDPNDATKSNVTLTEDGLSNGGNQITNVDSGLKGKDGQPVKLTEAAGD 894
Gen 2 isoform 12  795 AKFDAGKNIMTQVVGNTITVALAKDLAGLDSATFGTQGDSTVINKDGVTIAKKDPNDATKSNVTLTEDGLSNGGNQITNVDSGLKGKDGQPVKLTEAAGD 894
Gen 2 isoform 13  795 AKFDAGKNIMTQVVGNTITVALAKDLAGLDSATFGTQGDSTVINKDGVTIAKKDPNDATKSNVTLTEDGLSNGGNQITNVDSGLKGKDGQPVKLTEAAGD 894
Gen 2 isoform 14  795 AKFDAGKNIMTQVVGNTITVALAKDLAGLDSATFGTQGDSTVINKDGVTIAKKDPNDATKSNVTLTEDGLSNGGNQITNVDSGLKGKDGQPVKLTEAAGD 894
Gen 2 isoform 15  795 AKFDAGKNIMTQVVGNTITVALAKDLAGLDSATFGTQGDSTVINKDGVTIAKKDPNDATKSNVTLTEDGLSNGGNQITNVDSGLKGKDGQPVKLTEAAGD 894
Gen 2 isoform 16  795 AKFDAGKNIMTQVVGNTITVALAKDLAGLDSATFGTQGDSTVINKDGVTIAKKDPNDATKSNVTLTEDGLSNGGNQITNVDSGLKGKDGQPVKLTEAAGD 894
Gen 2 isoform 17  795 AKFDAGKNIMTQVVGNTITVALAKDLAGLDSATFGTQGDSTVINKDGVTIAKKDPNDATKSNVTLTEDGLSNGGNQITNVDSGLKGKDGQPVKLTEAAGD 894
Gen 2 isoform 18  795 AKFDAGKNIMTQVVGNTITVALAKDLAGLDSATFGTQGDSTVINKDGVTIAKKDPNDATKSNVTLTEDGLSNGGNQITNVDSGLKGKDGQPVKLTEAAGD 894
Gen 2 isoform 19  801 AKFDAGKNIMTQVVGNTITVALAKDLAGLDSATFGTQGDSTVINKDGVTIAKKDPNDATKSNVTLTEDGLSNGGNQITNVDSGLKGKDGQPVKLTEAAGD 900
Gen 2 isoform 20  795 AKFDAGKNIMTQVVGNTITVALAKDLAGLDSATFGTQGDSTVINKDGVTIAKKDPNDATKSNVTLTEDGLSNGGNQITNVDSGLKGKDGQPVKLTEAAGD 894
Gen 2 isoform 21  776 ---------------------------------------------------------------------------------------------------- 775
Gen 2 isoform 22  795 AKFDAGKNIMTQVVGNTITVALAKDLAGLDSATFGTQGDSTVINKDGVTIAKKDPNDATKSNVTLTEDGLSNGGNQITNVDSGLKGKDGQPVKLTEAAGD 894
```

Fig S13 continued

Gen 2 isoform 1	895	VLNNAVNVGDLKNTVTETVTEVTTDAFGLKDENNKEFKQNLGTTAKIIGDGNVKTNVVEKDGGHALEVSLVNNITVGKDGEPGKVGIAGKDGKDAIALDG	994
Gen 2 isoform 2	895	VLNNAVNVGDLKNTVTETVTEVTTDAFGLKDENNKEFKQNLGTTAKIIGDGNVKTNVVEKDGGHALEVSLVNNITVGKDGEPGKVGIAGKDGKDAIALDG	994
Gen 2 isoform 3	895	VLNNAVNVGDLKNTVTETVTEVTTDAFGLKDENNKEFKQNLGTTAKIIGDGNVKTNVVEKDGGHALEVSLVNNITVGKDGEPGKVGIAGKDGKDAIALDG	994
Gen 2 isoform 4	892	VLNNAVNVGDLKNTVTETVTEVTTDAFGLKDENNKEFKQNLGTTAKIIGDGNVKTNVVEKDGGHALEVSLVNNITVGKDGEPGKVGIAGKDGKDAIALDG	991
Gen 2 isoform 5	895	VLNNAVNVGDLKNTVTETVTEVTTDAFGLKDENNKEFKQNLGTTAKIIGDGNVKTNVVEKDGGHALEVSLVNNITVGKDGEPGKVGIAGKDGKDAIALDG	994
Gen 2 isoform 6	895	VLNNAVNVGDLKNTVTETVTEVTTDAFGLKDENNKEFKQNLGTTAKIIGDGNVKTNVVEKDGGHALEVSLVNNITVGKDGEPGKVGIAGKDGKDAIALDG	994
Gen 2 isoform 7	859	VLNNAVNVGDLKNTVTETVTEVTTDAFGLKDENNKEFKQNLGTTAKIIGDGNVKTNVVEKDGGHALEVSLVNNITVGKDGEPGKVGIAGKDGKDAIALDG	958
Gen 2 isoform 8	895	VLNNAVNVGDLKNTVTETVTEVTTDAFGLKDENNKEFKQNLGTTAKIIGDGNVKTNVVEKDGGHALEVSLVNNITVGKDGEPGKVGIAGKDGKDAIALDG	994
Gen 2 isoform 9	895	VLNNAVNVGDLKNTVTETVTEVTTDAFGLKDENNKEFKQNLGTTAKIIGDGNVKTNVVEKDGGHALEVSLVNNITVGKDGEPGKVGIAGKDGKDAIALDG	994
Gen 2 isoform 10	895	VLNNAVNVGDLKNTVTETVTEVTTDAFGLKDENNKEFKQNLGTTAKIIGDGNVKTNVVEKDGGHALEVSLVNNITVGKDGEPGKVGIAGKDGKDAIALDG	994
Gen 2 isoform 11	895	VLNNAVNVGDLKNTVTETVTEVTTDAFGLKDENNKEFKQNLGVEDQT*--	942
Gen 2 isoform 12	895	VLNNAVNVGDLKNTVTETVTEVTTDAFGLKDENNKEFKQNLGTTAKIIGDGNVKTNVVEKDGGHALEVSLVNNITVGKDGEPGKVGIAGKDGKDAIALDG	994
Gen 2 isoform 13	895	VLNNAVNVGDLKNTVTETVTEVTTDAFGLKDENNKEFKQNLGTTAKIIGDGNVKTNVVEKDGGHALEVSLVNNITVGKDGEPGKVGIAGKDGKDAIALDG	994
Gen 2 isoform 14	895	VLNNAVNVGDLKNTVTETVTEVTTDAFGLKDENNKEFKQNLGTTAKIIGDGNVKTNVVEKDGGHALEVSLVNNITVGKDGEPGKVGIAGKDGKDAIALDG	994
Gen 2 isoform 15	895	VLNNAVNVGDLKNTVTETVTEVTTDAFGLKDENNKEFKQNLGTTAKIIGDGNVKTNVVEKDGGHALEVSLVNNITVGKDGEPGKVGIAGKDGKDAIALDG	994
Gen 2 isoform 16	895	VLNNAVNVGDLKNTVTETVTEVTTDAFGLKDENNKEFKQNLGTTAKIIGDGNVKTNVVEKDGGHALEVSLVNNITVGKDGEPGKVGIAGKDGKDAIALDG	994
Gen 2 isoform 17	895	VLNNAVNVGDLKNTVTETVTEVTTDAFGLKDENNKEFKQNLGTTAKIIGDGNVKTNVVEKDGGHALEVSLVNNITVGKDGEPGKVGIAGKDGKDAIALDG	994
Gen 2 isoform 18	895	VLNNAVNVGDLKNTVTETVTEVTTDAFGLKDENNKEFKQNLGTTAKIIGDGNVKTNVVEKDGGHALEVSLVNNITVGKDGEPGKVGIAGKDGKDAIALDG	994
Gen 2 isoform 19	901	VLNNAVNVGDLKNTVTETVTEVTTDAFGLKDENNKEFKQNLGTTAKIIGDGNVKTNVVEKDGGHALEVSLVNNITVGKDGEPGKVGIAGKDGKDAIALDG	1000
Gen 2 isoform 20	895	VLNNAVNVGDLKNTVTETVTEVTTDAFGLKDENNKEFKQNLGTTAKIIGDGNVKTNVVEKDGGHALEVSLVNNITVGKDGEPGKVGIAGKDGKDAIALDG	994
Gen 2 isoform 21	776	--	775
Gen 2 isoform 22	895	VLNNAVNVGDLKNTVTETVTEVTTDAFGLKDENNKEFKQNLGTTAKIIGDGNVKTNVVEKDGGHALEVSLVNNITVGKDGEPGKVGIAGKDGKDAIALDG	994

Gen 2 isoform 1	995	KDGAITVGRPGKDGIDGTIGVNGKDGSAVVLNGKDGSIGLNGTNGKDGLTIKGTEGKPGLVGKDGLPGKDGVTRIQYQPTDGNGDPIGEPEQVATLNDGL	1094
Gen 2 isoform 2	995	KDGAITVGRPGKDGIDGTIGVNGKDGSAVVLNGKDGSIGLNGTNGKDGLTIKGTEGKPGLVGKDGLPGKDGVTRIQYQPTDGNGDPIGEPEQVATLNDGL	1094
Gen 2 isoform 3	995	KDGAITVGRPGKDGIDGTIGVNGKDGSAVVLNGKDGSIGLNGTNGKDGLTIKGTEGKPGLVGKDGLPGKDGVTRIQYQPTDGNGDPIGEPEQVATLNDGL	1094
Gen 2 isoform 4	992	KDGAITVGRPGKDGIDGTIGVNGKDGSAVVLNGKDGSIGLNGTNGKDGLTIKGTEGKPGLVGKDGLPGKDGVTRIQYQPTDGNGDPIGEPEQVATLNDGL	1091
Gen 2 isoform 5	995	KDGAITVGRPGKDGIDGTIGVNGKDGSAVVLNGKDGSIGLNGTNGKDGLTIKGTEGKPGLVGKDGLPGKDGVTRIQYQPTDGNGDPIGEPEQVATLNDGL	1094
Gen 2 isoform 6	995	KDGAITVGRPGKDGIDGTIGVNGKDGSAVVLNGKDGSIGLNGTNGKDGLTIKGTEGKPGLVGKDGLPGKDGVTRIQYQPTDGNGDPIGEPEQVATLNDGL	1094
Gen 2 isoform 7	959	KDGAITVGRPGKDGIDGTIGVNGKDGSAVVLNGKDGSIGLNGTNGKDGLTIKGTEGKPGLVGKDGLPGKDGVTRIQYQPTDGNGDPIGEPEQVATLNDGL	1058
Gen 2 isoform 8	995	KDGAITVGRPGKDGIDGTIGVNGKDGSAVVLNGKDGSIGLNGTNGKDGLTIKGTEGKPGLVGKDGLPGKDGVTRIQYQPTDGNGDPIGEPEQVATLNDGL	1094
Gen 2 isoform 9	995	KDGAITVGRPGKDGIDGTIGVNGKDGSAVVLNGKDGSIGLNGTNGKDGLTIKGTEGKPGLVGKDGLPGKDGVTRIQYQPTDGNGDPIGEPEQVATLNDGL	1094
Gen 2 isoform 10	995	KDGAITVGRPGKDGIDGTIGVNGKDGSAVVLNGKDGSIGLNGTNGKDGLTIKGTEGKPGLVGKDGLPGKDGVTRIQYQPTDGNGDPIGEPEQVATLNDGL	1094
Gen 2 isoform 11	943	--	942
Gen 2 isoform 12	995	KDGAITVGRPGKDGIDGTIGVNGKDGSAVVLNGKDGSIGLNGTNGKDGLTIKGTEGKPGLVGKD----------------------------------GL	1060
Gen 2 isoform 13	995	KDGAITVGRPGKDGIDGTIGVNGKDGSAVVLNGKDGSIGLNGTNGKDGLTIKGTEGKPGLVGKDGLPGKDGVTRIQYQPTDGNGDPIGEPEQVATLNDGL	1094
Gen 2 isoform 14	995	KDGAITVGRPGKDGIDGTIGVNGKDGSAVVLNGKDGSIGLNGTNGKDGLTIKGTEGKPGLVGKDGLPGKDGVTRIQYQPTDGNGDPIGEPEQVATLNDGL	1094
Gen 2 isoform 15	995	KDGAITVGRPGKDGIDGTIGVNGKDGSAVVLNGKDGSIGLNGTNGKDGLTIKGTEGKPGLVGKDGLPGKDGVTRIQYQPTDGNGDPIGEPEQVATLNDGL	1094
Gen 2 isoform 16	995	KDGAITVGRPGKDGIDGTIGVNGKDGSAVVLNGKDGSIGLNGTNGKDGLTIKGTEGKPGLVGKDGLPGKDGVTRIQYQPTDGNGDPIGEPEQVATLNDGL	1094
Gen 2 isoform 17	995	KDGAITVGRPGKDGIDGTIGVNGKDGSAVVLNGKDGSIGLNGTNGKDGLTIKGTEGKPGLVGKDGLPGKDGVTRIQYQPTDGNGDPIGEPEQVATLNDGL	1094
Gen 2 isoform 18	995	KDGAITVGRPGKDGIDGTIGVNGKDGSAVVLNGKDGSIGLNGTNGKDGLTIKGTEGKPGLVGKDGLPGKDGVTRIQYQPTDGNGDPIGEPEQVATLNDGL	1094
Gen 2 isoform 19	1001	KDGAITVGRPGKDGIDGTIGVNGKDGSAVVLNGKDGSIGLNGTNGKDGLTIKGTEGKPGLVGKDGLPGKDGVTRIQYQPTDGNGDPIGEPEQVATLNDGL	1100
Gen 2 isoform 20	995	KDGAITVGRPGKDGIDGTIGVNGKDGSAVVLNGKDGSIGLNGTNGKDGLTIKGTEGKPGLVGKDGLPGKDGVTRIQYQPTDGNGDPIGEPEQVATLNDGL	1094
Gen 2 isoform 21	776	--	775
Gen 2 isoform 22	995	KDGAITVGRPGKDGIDGTIGVNGKDGSAVVLNGKDGSIGLNGTNGKDGLTIKGTEGKPGLVGKDGLPGKDGVTRIQYQPTDGNGDPIGEPEQVATLNDGL	1094

Gen 2 isoform 1	1095	HFKGDDATLVSKKLNETLEIIGGADKDKLTANNIGVNSKDGKLSVQLAKDLKDLNSATFKPQDGENGQEGKGPTTVVNNNGVTITPKDGTDGKAVKLTED	1194
Gen 2 isoform 2	1095	HFKGDDATLVSKKLNETLEIIGGADKDKLTANNIGVNSKDGKLSVQLAKDLKDLNSATFKPQDGENGQEGKGPTTVVNNNGVTITPKDGTDGKAVKLTED	1194
Gen 2 isoform 3	1095	HFKGDDATLVSKKLNETLEIIGGADKDKLTANNIGVNSKDGKLSVQLAKDLKDLNSATFKPQDGENGQEGKGPTTVVNNNGVTITPKDGTDGKAVKLTED	1194
Gen 2 isoform 4	1092	HFKGDDATLVSKKLNETLEIIGGADKDKLTANNIGVNSKDGKLSVQLAKDLKDLNSATFKPQDGENGQEGKGPTTVVNNNGVTITPKDGTDGKAVKLTED	1191
Gen 2 isoform 5	1095	HFKGDDATLVSKKLNETLEIIGGADKDKLTANNIGVNSKDGKLSVQLAKDLKDLNSATFKPQDGENGQEGKGPTTVVNNNGVTITPKDGTDGKAVKLTED	1194
Gen 2 isoform 6	1095	HFKGDDATLVSKKLNETLEIIGGADKDKLTANNIGVNSKDGKLSVQLAKDLKDLNSATFKPQDGENGQEGKGPTTVVNNNGVTITPKDGTDGKAVKLTED	1194
Gen 2 isoform 7	1059	HFKGDDATLVSKKLNETLEIIGGADKDKLTANNIGVNSKDGKLSVQLAKDLKDLNSATFKPQDGENGQEGKGPTTVVNNNGVTITPKDGTDGKAVKLTED	1158
Gen 2 isoform 8	1095	HFKGDDATLVSKKLNETLEIIGGADKDKLTANNIGVNSKDGKLSVQLAKDLKDLNSATFKPQDGENGQEGKGPTTVVNNNGVTITPKDGTDGKAVKLTED	1194
Gen 2 isoform 9	1095	HFKGDDATLVSKKLNETLEIIGGADKDKLTANNIGVNSKDGKLSVQLAKDLKDLNSATFKPQDGENGQEGKGPTTVVNNNGVTITPKDGTDGKAVKLTED	1194
Gen 2 isoform 10	1095	HFKGDDATLVSKKLNETLEIIGGADKDKLTANNIGVNSKDGKLSVQLAKDLKDLNSATFKPQDGENGQEGKGPTTVVNNNGVTITPKDGTDGKAVKLTED	1194
Gen 2 isoform 11	943	--	942
Gen 2 isoform 12	1061	HFKGDDATLVSKKLNETLEIIGGADKDKLTANNIGVNSKDGKLSVQLAKDLKDLNSATFKPQDGENGQEGKGPTTVVNNNGVTITPKDGTDGKAVKLTED	1160
Gen 2 isoform 13	1095	HFKGDDATLVSKKLNETLEIIGGADKDKLTANNIGVNSKDGKLSVQLAKDLKDLNSATFKPQDGENGQEGKGPTTVVNNNGVTITPKDGTDGKAVKLTED	1194
Gen 2 isoform 14	1095	HFKGDDATLVSKKLNETLEIIGGADKDKLTANNIGVNSKDGKLSVQLAKDLKDLNSATFKPQDGENGQEGKGPTTVVNNNGVTITPKDGTDGKAVKLTED	1194
Gen 2 isoform 15	1095	HFKGDDATLVSKKLNETLEIIGGADKDKLTANNIGVNSKDGKLSVQLAKDLKDLNSATFKPQDGENGQEGKGPTTVVNNNGVTITPKDGTDGKAVKLTED	1194
Gen 2 isoform 16	1095	HFKGDDATLVSKKLNETLEIIGGADKDKLTANNIGVNSKDGKLSVQLAKDLKDLNSATFKPQDGENGQEGKGPTTVVNNNGVTITPKDGTDGKAVKLTED	1194
Gen 2 isoform 17	1095	HFKGDDATLVSKKLNETLEIIGGADKDKLTANNIGVNSKDGKLSVQLAKDLKDLNSATFKPQDGENGQEGKGPTTVVNNNGVTITPKDGTDGKAVKLTED	1194
Gen 2 isoform 18	1095	HFKGDDATLVSKKLNETLEIIGGADKDKLTANNIGVNSKDGKLSVQLAKDLKDLNSATFKPQDGENGQEGKGPTTVVNNNGVTITPKDGTDGKAVKLTED	1194
Gen 2 isoform 19	1101	HFKGDDATLVSKKLNETLEIIGGADKDKLTANNIGVNSKDGKLSVQLAKDLKDLNSATFKPQDGENGQEGKGPTTVVNNNGVTITPKDGTDGKAVKLTED	1200
Gen 2 isoform 20	1095	HFKGDDATLVSKKLNETLEIIGGADKDKLTANNIGVNSKDGKLSVQLAKDLKDLNSATFKPQDGENGQEGKGPTTVVNNNGVTITPKDGTDGKAVKLTED	1194
Gen 2 isoform 21	776	--	775
Gen 2 isoform 22	1095	HFKGDDATLVSKKLNETLEIIGGADKDKLTANNIGVNSKDGKLSVQLAKDLKDLNSATFKPQDGENGQEGKGPTTVVNNNGVTITPKDGTDGKAVKLTED	1194

Fig S13 continued

Sequence	Start	Alignment	End
Gen 2 isoform 1	1195	GLDNGGNQIVNVKSGIDGLAGKDGINGKDGKPITSVNDLTDDQLKEVGTNAANINDVVNASNDIIEKGFSIAADNGETDHVKLGETVKYTSKDKNIITTV	1294
Gen 2 isoform 2	1195	GLDNGGNQIVNVKSGIDGLAGKDGINGKDGKPITSVNDLTDDQLKEVGTNAANINDVVNASNDIIEKGFSIAADNGETDHVKLGETVKYTSKDKNIITTV	1294
Gen 2 isoform 3	1195	GLDNGGNQIVNVKSGIDGLAGKDGINGKDGKPITSVNDLTDDQLKEVGTNAANINDVVNASNDIIEKGFSIAADNGETDHVKLGETVKYTSKDKNIITTV	1294
Gen 2 isoform 4	1192	GLDNGGNQIVNVKSGIDGLAGKDGINGKDGKPITSVNDLTDDQLKEVGTNAANINDVVNASNDIIEKGFSIAADNGETDHVKLGETVKYTSKDKNIITTV	1291
Gen 2 isoform 5	1195	GLDNGGNQIVNVKSGIDGLAGKDGINGKDGKPITSVNDLTDDQLKEVGTNAANINDVVNASNDIIEKGFSIAADNGETDHVKLGETVKYTSKDKNIITTV	1294
Gen 2 isoform 6	1195	GLDNGGNQIVNVKSGIDGLAGKDGINGKDGKPITSVNDLTDDQLKEVGTNAANINDVVNASNDIIEKGFSIAADNGETDHVKLGETVKYTSKDKNIITTV	1294
Gen 2 isoform 7	1159	GLDNGGNQIVNVKSGIDGLAGKDGINGKDGKPITSVNDLTDDQLKEVGTNAANINDVVNASNDIIEKGFSIAADNGETDHVKLGETVKYTSKDKNIITTV	1258
Gen 2 isoform 8	1195	GLDNGGNQIVNVKSGIDGLAGKDGINGKDGKPITSVNDLTDDQLKEVGTNAANINDVVNASNDIIEKGFSIAADNGETDHVKLGETVKYTSKDKNIITTV	1294
Gen 2 isoform 9	1195	GLDNGGNQIVNVKSGIDGLAGKDGINGKDGKPITSVNDLTDDQLKEVGTNAANINDVVNASNDIIEKGFSIAADNGETDHVKLGETVKYTSKDKNIITTV	1294
Gen 2 isoform 10	1195	GLDNGGNQIVNVKSGIDGLAGKDGINGKDGKPITSVNDLTDDQLKEVGTNAANINDVVNASNDIIEKGFSIAADNGETDHVKLGETVKYTSKDKNIITTV	1294
Gen 2 isoform 11	943	....	942
Gen 2 isoform 12	1161	GLDNGGNQIVNVKSGIDGLAGKDGINGKDGKPITSVNDLTDDQLKEVGTNAANINDVVNASNDIIEKGFSIAADNGETDHVKLGETVKYTSKDKNIITTV	1260
Gen 2 isoform 13	1195	GLDNGGNQIVNVKSGIDGLAGKDGINGKDGKPITSVNDLTDDQLKEVGTNAANINDVVNASNDIIEKGFSIAADNGETDHVKLGETVKYTSKDKNIITTV	1294
Gen 2 isoform 14	1195	GLDNGGNQIVNVKSGIDGLAGKDGINGKDGKPITSVNDLTDDQLKEVGTNAANINDVVNASNDIIEKGFSIAADNGETDHVKLGETVKYTSKDKNIITTV	1294
Gen 2 isoform 15	1195	GLDNGGNQIVNVKSGIDGLAGKDGINGKDGKPITSVNDLTDDQLKEVGTNAANINDVVNASNDIIEKGFSIAADNGETDHVKLGETVKYTSKDKNIITTV	1294
Gen 2 isoform 16	1195	GLDNGGNQIVNVKSGIDGLAGKDGINGKDGKPITSVNDLTDDQLKEVGTNAANINDVVNASNDIIEKGFSIAADNGETDHVKLGETVKYTSKDKNIITTV	1294
Gen 2 isoform 17	1195	GLDNGGNQIVNVKSGIDGLAGKDGINGKDGKPITSVNDLTDDQLKEVGTNAANINDVVNASNDIIEKGFSIAADNGETDHVKLGETVKYTSKDKNIITTV	1294
Gen 2 isoform 18	1195	GLDNGGNQIVNVKSGIDGLAGKDGINGKDGKPITSVNDLTDDQLKEVGTNAANINDVVNASNDIIEKGFSIAADNGETDHVKLGETVKYTSKDKNIITTV	1294
Gen 2 isoform 19	1201	GLDNGGNQIVNVKSGIDGLAGKDGINGKDGKPITSVNDLTDDQLKEVGTNAANINDVVNASNDIIEKGFSIAADNGETDHVKLGETVKYTSKDKNIITTV	1300
Gen 2 isoform 20	1195	GLDNGGNQIVNVKSGIDGLAGKDGINGKDGKPITSVNDLTDDQLKEVGTNAANINDVVNASNDIIEKGFSIAADNGETDHVKLGETVKYTSKDKNIITTV	1294
Gen 2 isoform 21	776	....	775
Gen 2 isoform 22	1195	GLDNGGNQIVNVKSGIDGLAGKDGINGKDGKPITSVNDLTDDQLKEVGTNAANINDVVNASNDIIEKGFSIAADNGETDHVKLGETVKYTSKDKNIITTV	1294

Sequence	Start	Alignment	End
Gen 2 isoform 1	1295	SDNVIDFTLNNDFTIGGPGKDGSIGVKGADGTVGVTLNGKDGSIGLTGPKGADGKDGASATISVKDGAKGLDGNDGKDGESKTRIVYEKPDGTTEEVATL	1394
Gen 2 isoform 2	1295	SDNVIDFTLNNDFTIGGPGKDGSIGVKGADGTVGVTLNGKDGSIGLTGPKGADGKDGASATISVKDGAKGLDGNDGKDGESKTRIVYEKPDGTTEEVATL	1394
Gen 2 isoform 3	1295	SDNVIDFTLNNDFTIGGPGKDGSIGVKGADGTVGVTLNGKDGSIGLTGPKGADGKDGASATISVKDGAKGLDGNDGKDGESKTRIVYEKPDGTTEEVATL	1394
Gen 2 isoform 4	1292	SDNVIDFTLNNDFTIGGPGKDGSIGVKGADGTVGVTLNGKDGSIGLTGPKGADGKDGASATISVKDGAKGLDGNDGKDGESKTRIVYEKPDGTTEEVATL	1391
Gen 2 isoform 5	1295	SDNVIDFTLNNDFTIGGPGKDGSIGVKGADGTVGVTLNGKDGSIGLTGPKGADGKD..	1350
Gen 2 isoform 6	1295	SDNVIDFTLNNDFTIGGPGKDGSIGVKGADGTVGVTLNGKDGSIGLTGPKGADGKDGASATISVKDGAKGLDGNDGKDGESKTRIVYEKPDGTTEEVATL	1394
Gen 2 isoform 7	1259	SDNVIDFTLNNDFTIGGPGKDGSIGVKGADGTVGVTLNGKDGSIGLTGPKGADGKDGASATISVKDGAKGLDGNDGKDGESKTRIVYEKPDGTTEEVATL	1358
Gen 2 isoform 8	1295	SDNVIDFTLNNDFTIGGPGKDGSIGVKGADGTVGVTLNGKDGSIGLTGPKGADGKDGASATISVKDGAKGLDGNDGKDGESKTRIVYEKPDGTTEEVATL	1394
Gen 2 isoform 9	1295	SDNVIDFTLNNDFTIGGPGKDGSIGVKGADGTVGVTLNGKDGSIGLTGPKGADGKDGASATISVKDGAKGLDGNDGKDGESKTRIVYEKPDGTTEEVATL	1394
Gen 2 isoform 10	1295	SDNVIDFTLNNDFTIGGPGKDGSIGVKGADGTVGVTLNGKDGSIGLTGPKGADGKDGASATISVKDGAKGLDGNDGKDGESKTRIVYEKPDGTTEEVATL	1394
Gen 2 isoform 11	943	....	942
Gen 2 isoform 12	1261	SDNVIDFTLNNDFTIGGPGKDGSIGVKGADGTVGVTLNGKDGSIGLTGPKGADGKDGASATISVKDGAKGLDGNDGKDGESKTRIVYEKPDGTTEEVATL	1360
Gen 2 isoform 13	1295	SDNVIDFTLNNDFTIGGPGKDGSIGVKGADGTVGVVLNGKDGSIGLTGPKGADGKD..	1350
Gen 2 isoform 14	1295	SDNVIDFTLNNDFTIGGPGKDGSIGVKGADGTVGVTLNGKDGSIGLTGPKGADGKDGASATISVKDGAKGLDGNDGKDGESKTRIVYEKPDGTTEEVATL	1394
Gen 2 isoform 15	1295	SDNVIDFTLNNDFTIGGPGKDGSIGVKGADGTVGVTLNGKDGSIGLTGPKGADGKDGASATISVKDGAKGLDGNDGKDGESKTRIVYEKPDGTTEEVATL	1394
Gen 2 isoform 16	1295	SDNVIDFTLNNDFTIGGPGKDGSIGVKGADGTVGVTLNGKDGSIGLTGPKGADGKDGASATISVKDGAKGLDGNDGKDGESKTRIVYEKPDGTTEEVATL	1394
Gen 2 isoform 17	1295	SDNVIDFTLNNDFTIGGPGKDGSIGVKGADGTVGVTLNGKDGSIGLTGPKGADGKDGASATISVKDGAKGLDGNDGKDGESKTRIVYEKPDGTTEEVATL	1394
Gen 2 isoform 18	1295	SDNVIDFTLNNDFTIGGPGKDGSIGVKGADGTVGVTLNGKDGSIGLTGPKGADGKDGASATISVKDGAKGLDGNDGKDGESKTRIVYEKPDGTTEEVATL	1394
Gen 2 isoform 19	1301	SDNVIDFTLNNDFTIGGPGKDGSIGVKGADGTVGVTLNGKDGSIGLTGPKGADGKDGASATISVKDGAKGLDGNDGKDGESKTRIVYEKPDGTTEEVATL	1400
Gen 2 isoform 20	1295	SDNVIDFTLNNDFTIGGPGKDGSIGVKGADGTVGVTLNGKDGSIGLTGPKGADGKDGASATISVKDGAKGLDGNDGKDGESKTRIVYEKPDGTTEEVATL	1394
Gen 2 isoform 21	776	....	775
Gen 2 isoform 22	1295	SDNVIDFTLNNDFTIGGPGKDGSIGVKGADGTVGVTLNGKDGSIGLTGPKGADGKDGASATISVKDGAKGLDGNDGKDGESKTRIVYEKPDGTTEEVATL	1394

Sequence	Start	Alignment	End
Gen 2 isoform 1	1395	NDGLRFVGDDGKEIVKKLNETLKISGGDTDLTNLSDNNIGVVNNNGALTVKLAKDIDLTKDGSVTIGDTTVNNDGLTIKDGPSVTKAGINAGDKKIINVQ	1494
Gen 2 isoform 2	1395	NDGLRFVGDDGKEIVKKLNETLKISGGDTDLTNLSDNNIGVVNNNGALTVK*AKDIDLTKDGSVTIGDTTVNNDGLTIKDGPSVTKAGINAGDKKIINVQ	1494
Gen 2 isoform 3	1395	NDGLRFVGDDGKEIVKKLNETLKISGGDTDLTNLSDNNIGVVNNNGALTVKLAKDIDLTKDGSVTIGDTTVNNDGLTIKDGPSVTKAGINAGDKKIINVQ	1494
Gen 2 isoform 4	1392	NDGLRFVGDDGKEIVKKLNETLKISGGDTDLTNLSDNNIGVVNNNGALTVKLAKDIDLTKDGSVTIGDTTVNNDGLTIKDGPSVTKAGINAGDKKIINVQ	1491
Gen 2 isoform 5	1351	....	1350
Gen 2 isoform 6	1395	NDGLRFVGDDGKEIVKKLNETLKISGGDTDLTNLSDNNIGVVNNNGALTVKLAKDIDLTKDGSVTIGDTTVNNDGLTIKDGPSVTKAGINAGDKKIINVQ	1494
Gen 2 isoform 7	1359	NDGLRFVGDDGKEIVKKLNETLKISGGDTDLTNLSDNNIGVVNNNGALTVKLAKDIDLTKDGSVTIGDTTVNNDGLTIKDGPSVTKAGINAGDKKIINVQ	1458
Gen 2 isoform 8	1395	NDGLRFVGDDGKEIVKKLNETLKISGGDTDLTNLSDNNIGVVNNNGALTVKLAKDIDLTKDGSVTIGDTTVNNDGLTIKDGPSVTKAGINAGDKKIINVQ	1494
Gen 2 isoform 9	1395	NDGLRFVGDDGKEIVKKLNETLKISGGDTDLTNLSDNNIGVVNNNGALTVKLAKDIDLTKDGSVTIGDTTVNNDGLTIKDGPSVTKAGINAGDKKIINVQ	1494
Gen 2 isoform 10	1395	NDGLRFVGDDGKEIVKKLNETLKISGGDTDLTNLSDNNIGVVNNNGALTVKLAKDIDLTKDGSVTIGDTTVNNDGLTIKDGPSVTKAGINAGDKKIINVQ	1494
Gen 2 isoform 11	943	....	942
Gen 2 isoform 12	1361	NDGLRFVGDDGKEIVKKLNETLKISGGDTDLTNLSDNNIGVVNNNGALTVKLAKDIDLTKDGSVTIGDTTVNNDGLTIKDGPSVTKAGINAGDKKIINVQ	1460
Gen 2 isoform 13	1351	....	1350
Gen 2 isoform 14	1395	NDGLRFVGDDGKEIVKKLNETLKISGGDTDLTNLSDNNIGVVNNNGALTVKLAKDIDLTKDGSVTIGDTTVNNDGLTIKDGPSVTKAGINAGDKKIINVQ	1494
Gen 2 isoform 15	1395	NDGLRFVGDDGKEIVKKLNETLKISGGDTDLTNLSDNNIGVVNNNGALTVKLAKDIDLTKDGSVTIGDTTVNNDGLTIKDGPSVTKAGINAGDKKIINVQ	1494
Gen 2 isoform 16	1395	NDGLRFVGDDGKEIVKKLNETLKISGGDTDLTNLSDNNIGVVNNNGALTVKLAKDIDLTKDGSVTIGDTTVNNDGLTIKDGPSVTKAGINAGDKKIINVQ	1494
Gen 2 isoform 17	1395	NDGLRFVGDDGKEIVKKLNETLKISGGDTDLTNLSDNNIGVVNNNGALTVKLAKDIDLTKDGSVTIGDTTVNNDGLTIKDGPSVTKAGINAGDKKIINVQ	1494
Gen 2 isoform 18	1395	NDGLRFVGDDGKEIVKKLNETLKISGGDTDLTNLSDNNIGVVNNNGALTVKLAKDIDLTKDGSVTIGDTTVNNDGLTIKDGPSVTKAGINAGDKKIINVQ	1494
Gen 2 isoform 19	1401	NDGLRFVGDDGKEIVKKLNETLKISGGDTDLTNLSDNNIGVVNNNGALTVKLAKDIDLTKDGSVTIGDTTVNNDGLTIKDGPSVTKAGINAGDKKIINVQ	1500
Gen 2 isoform 20	1395	NDGLRFVGDDGKEIVKKLNETLKISGGDTDLTNLSDNNIGVVNNNGALTVKLAKDIDLTKDGSVTIGDTTVNNDGLTIKDGPSVTKAGINAGDKKIINVQ	1494
Gen 2 isoform 21	776	....	775
Gen 2 isoform 22	1395	NDGLRFVGDDGKEIVKKLNETLKISGGDTDLTNLSDNNIGVVNNNGALTVK*AKDIDLTKDGSVTIGDTTVNNDGLTIKDGPSVTKAGINAGDKKIINVQ	1494

Fig S13 continued

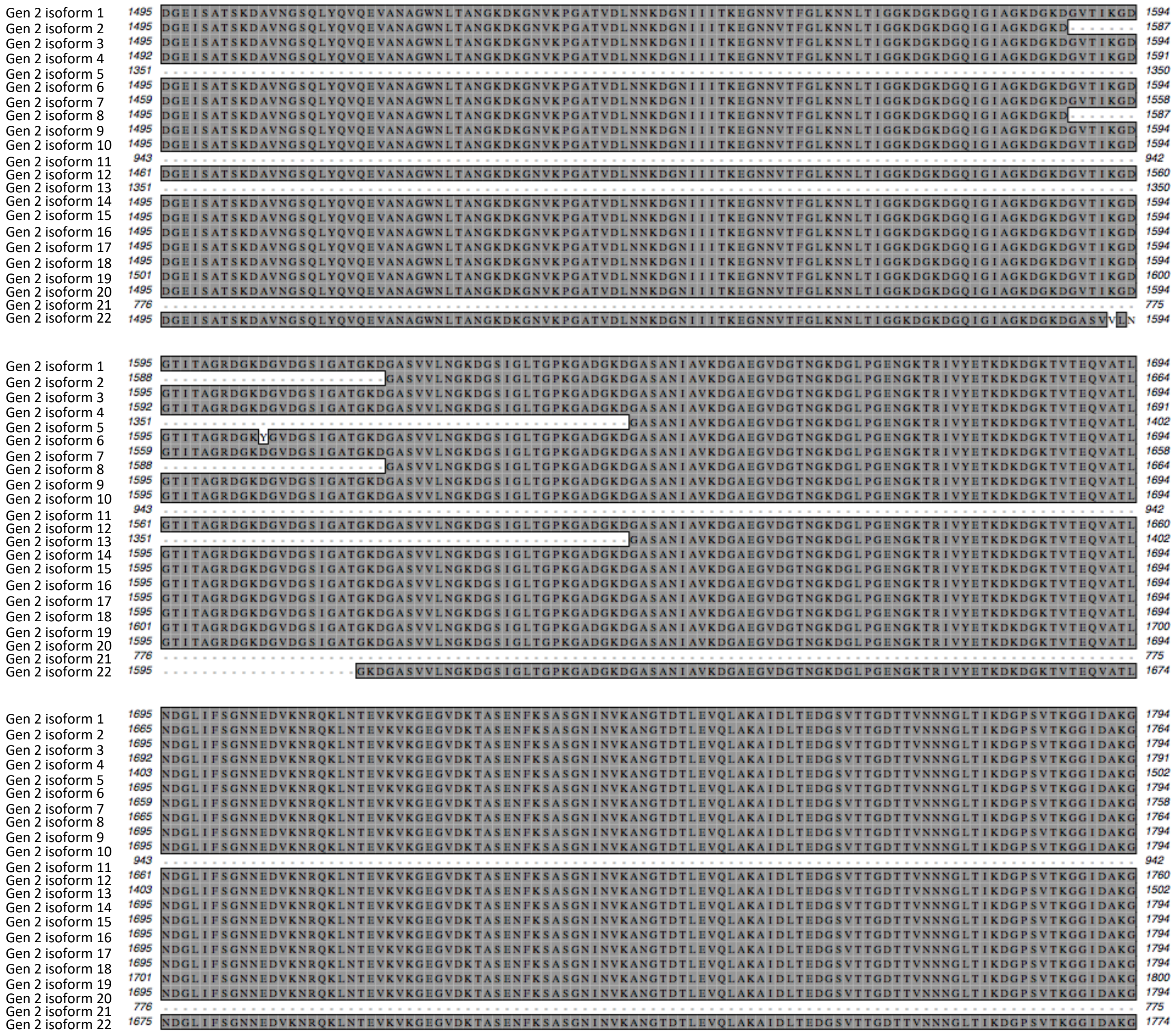

Fig S13 continued

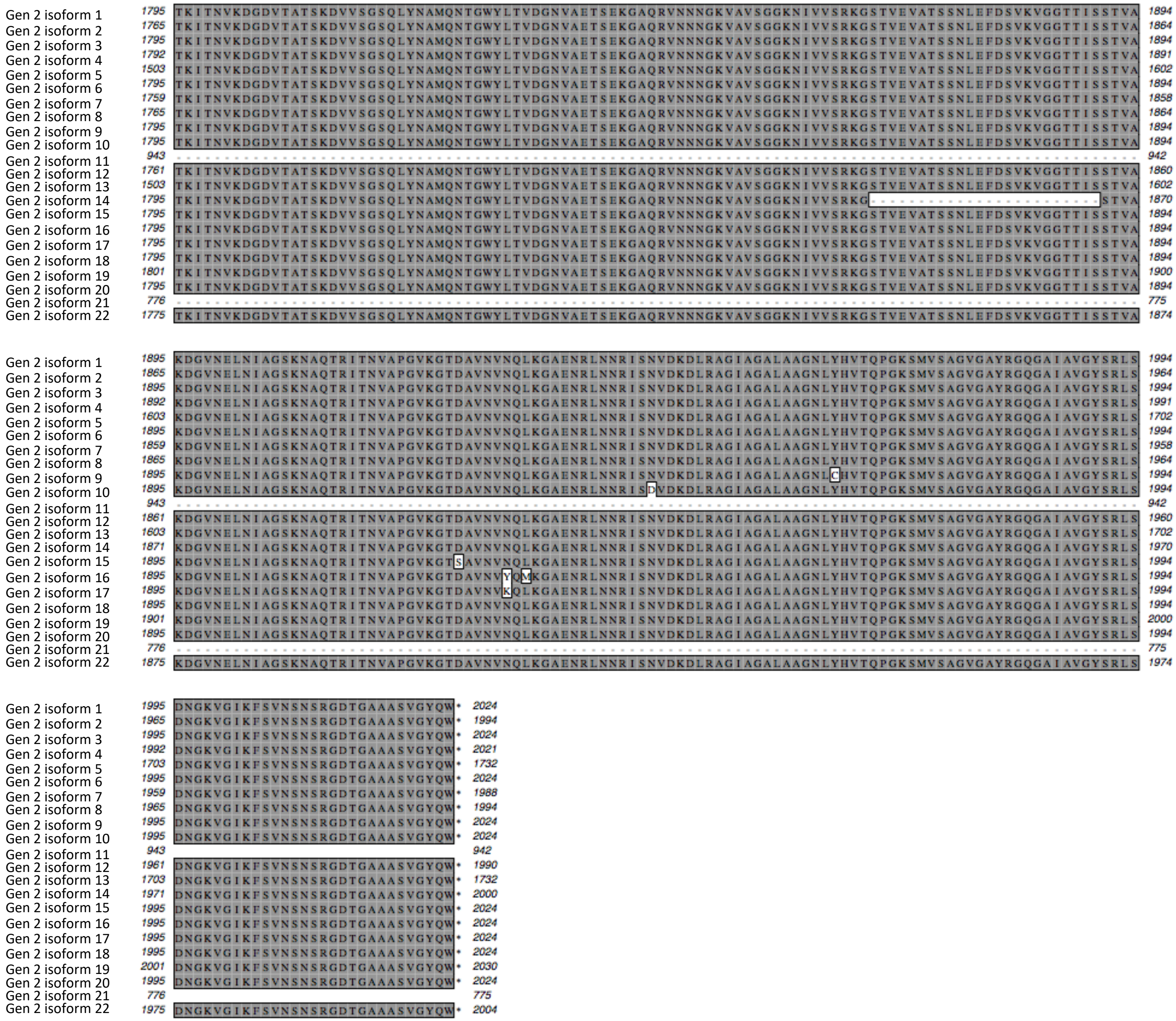

Supplement: Supplementary file 18 — Additional file 18: Figure S13. Alignment of adhesin G isoforms observed in genotype 2 M. haemolytica strains. The alignment contains adhesin G isoforms observed in genotype 2 M. haemolytica strains. Areas of 51% chemical identity or greater are indicated with grey boxes. [file 12866_2020_1932_MOESM18_ESM.pdf]
