# Supplementary material for: Differences between predicted outer membrane proteins of genotype 1 and 2 Mannheimia haemolytica
Source: BMC Microbiol. 2020 Aug 12;20:250. doi: 10.1186/s12866-020-01932-2 (PMC7424683; doi:10.1186/s12866-020-01932-2)

Fig S14

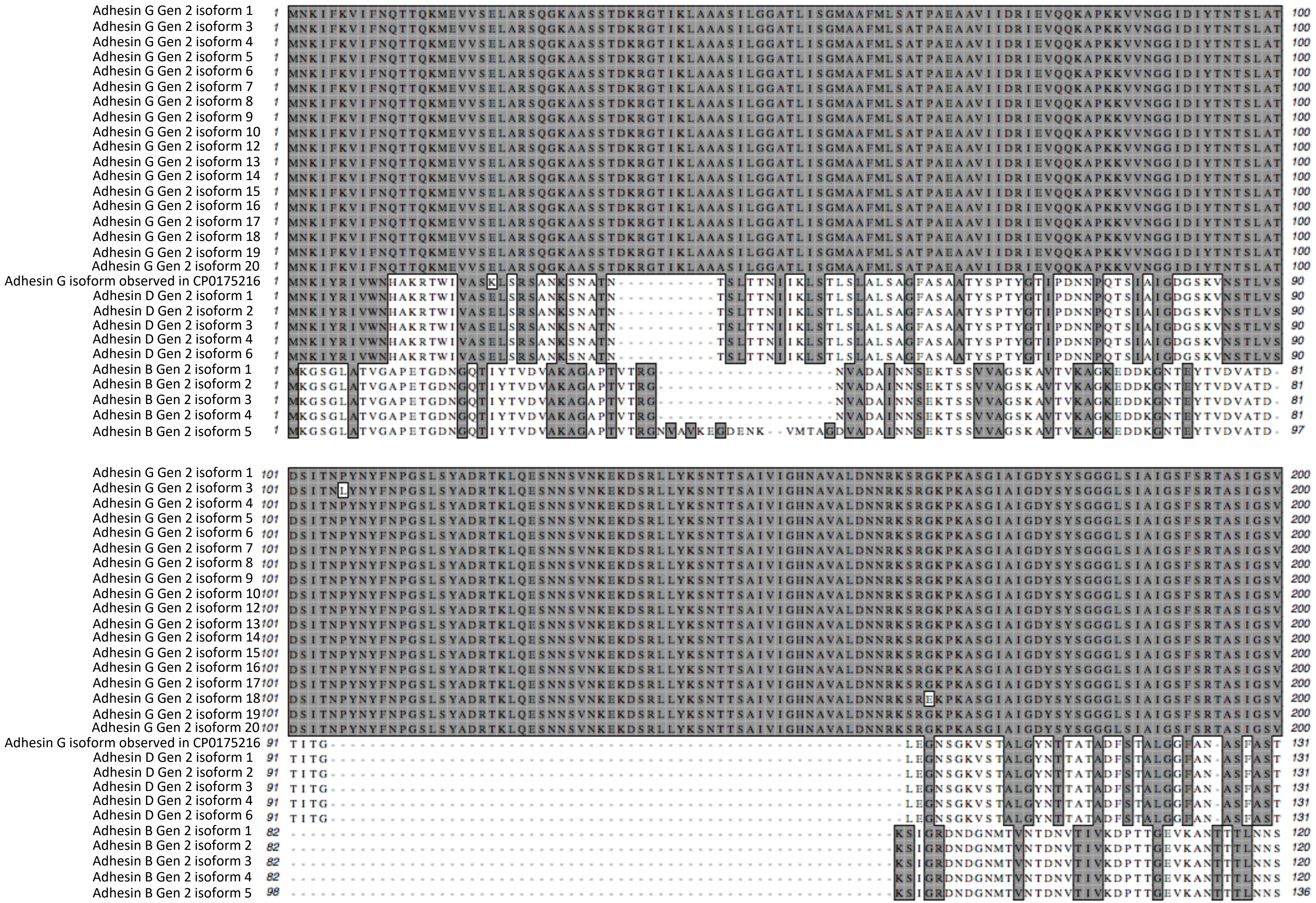
Adhesin G Gen 2 isoform 1 1 MNKIFKVIFNQTTQKMEVVSELARSQGKAASSTDKRGTIKLAAASILGGATLISGMAAFMLSATPAEAAVIIDRIEVQQKAPKKVVNGGIDIYTNTSLAT 100
Adhesin G Gen 2 isoform 3 1 MNKIFKVIFNQTTQKMEVVSELARSQGKAASSTDKRGTIKLAAASILGGATLISGMAAFMLSATPAEAAVIIDRIEVQQKAPKKVVNGGIDIYTNTSLAT 100
Adhesin G Gen 2 isoform 4 1 MNKIFKVIFNQTTQKMEVVSELARSQGKAASSTDKRGTIKLAAASILGGATLISGMAAFMLSATPAEAAVIIDRIEVQQKAPKKVVNGGIDIYTNTSLAT 100
Adhesin G Gen 2 isoform 5 1 MNKIFKVIFNQTTQKMEVVSELARSQGKAASSTDKRGTIKLAAASILGGATLISGMAAFMLSATPAEAAVIIDRIEVQQKAPKKVVNGGIDIYTNTSLAT 100
Adhesin G Gen 2 isoform 6 1 MNKIFKVIFNQTTQKMEVVSELARSQGKAASSTDKRGTIKLAAASILGGATLISGMAAFMLSATPAEAAVIIDRIEVQQKAPKKVVNGGIDIYTNTSLAT 100
Adhesin G Gen 2 isoform 7 1 MNKIFKVIFNQTTQKMEVVSELARSQGKAASSTDKRGTIKLAAASILGGATLISGMAAFMLSATPAEAAVIIDRIEVQQKAPKKVVNGGIDIYTNTSLAT 100
Adhesin G Gen 2 isoform 8 1 MNKIFKVIFNQTTQKMEVVSELARSQGKAASSTDKRGTIKLAAASILGGATLISGMAAFMLSATPAEAAVIIDRIEVQQKAPKKVVNGGIDIYTNTSLAT 100
Adhesin G Gen 2 isoform 9 1 MNKIFKVIFNQTTQKMEVVSELARSQGKAASSTDKRGTIKLAAASILGGATLISGMAAFMLSATPAEAAVIIDRIEVQQKAPKKVVNGGIDIYTNTSLAT 100
Adhesin G Gen 2 isoform 10 1 MNKIFKVIFNQTTQKMEVVSELARSQGKAASSTDKRGTIKLAAASILGGATLISGMAAFMLSATPAEAAVIIDRIEVQQKAPKKVVNGGIDIYTNTSLAT 100
Adhesin G Gen 2 isoform 12 1 MNKIFKVIFNQTTQKMEVVSELARSQGKAASSTDKRGTIKLAAASILGGATLISGMAAFMLSATPAEAAVIIDRIEVQQKAPKKVVNGGIDIYTNTSLAT 100
Adhesin G Gen 2 isoform 13 1 MNKIFKVIFNQTTQKMEVVSELARSQGKAASSTDKRGTIKLAAASILGGATLISGMAAFMLSATPAEAAVIIDRIEVQQKAPKKVVNGGIDIYTNTSLAT 100
Adhesin G Gen 2 isoform 14 1 MNKIFKVIFNQTTQKMEVVSELARSQGKAASSTDKRGTIKLAAASILGGATLISGMAAFMLSATPAEAAVIIDRIEVQQKAPKKVVNGGIDIYTNTSLAT 100
Adhesin G Gen 2 isoform 15 1 MNKIFKVIFNQTTQKMEVVSELARSQGKAASSTDKRGTIKLAAASILGGATLISGMAAFMLSATPAEAAVIIDRIEVQQKAPKKVVNGGIDIYTNTSLAT 100
Adhesin G Gen 2 isoform 16 1 MNKIFKVIFNQTTQKMEVVSELARSQGKAASSTDKRGTIKLAAASILGGATLISGMAAFMLSATPAEAAVIIDRIEVQQKAPKKVVNGGIDIYTNTSLAT 100
Adhesin G Gen 2 isoform 17 1 MNKIFKVIFNQTTQKMEVVSELARSQGKAASSTDKRGTIKLAAASILGGATLISGMAAFMLSATPAEAAVIIDRIEVQQKAPKKVVNGGIDIYTNTSLAT 100
Adhesin G Gen 2 isoform 18 1 MNKIFKVIFNQTTQKMEVVSELARSQGKAASSTDKRGTIKLAAASILGGATLISGMAAFMLSATPAEAAVIIDRIEVQQKAPKKVVNGGIDIYTNTSLAT 100
Adhesin G Gen 2 isoform 19 1 MNKIFKVIFNQTTQKMEVVSELARSQGKAASSTDKRGTIKLAAASILGGATLISGMAAFMLSATPAEAAVIIDRIEVQQKAPKKVVNGGIDIYTNTSLAT 100
Adhesin G Gen 2 isoform 20 1 MNKIFKVIFNQTTQKMEVVSELARSQGKAASSTDKRGTIKLAAASILGGATLISGMAAFMLSATPAEAAVIIDRIEVQQKAPKKVVNGGIDIYTNTSLAT 100
Adhesin G isoform observed in CP0175216 1 MNKIYRIVWNHAKRTWIVASKLSRSANKSNATN----------TSLTTNIIKLSTLSLALSAGFASAATYSPTYGTIPDNNPQTSIAIGDGSKVNSTLVS 90
Adhesin D Gen 2 isoform 1 1 MNKIYRIVWNHAKRTWIVASELSRSANKSNATN----------TSLTTNIIKLSTLSLALSAGFASAATYSPTYGTIPDNNPQTSIAIGDGSKVNSTLVS 90
Adhesin D Gen 2 isoform 2 1 MNKIYRIVWNHAKRTWIVASELSRSANKSNATN----------TSLTTNIIKLSTLSLALSAGFASAATYSPTYGTIPDNNPQTSIAIGDGSKVNSTLVS 90
Adhesin D Gen 2 isoform 3 1 MNKIYRIVWNHAKRTWIVASELSRSANKSNATN----------TSLTTNIIKLSTLSLALSAGFASAATYSPTYGTIPDNNPQTSIAIGDGSKVNSTLVS 90
Adhesin D Gen 2 isoform 4 1 MNKIYRIVWNHAKRTWIVASELSRSANKSNATN----------TSLTTNIIKLSTLSLALSAGFASAATYSPTYGTIPDNNPQTSIAIGDGSKVNSTLVS 90
Adhesin D Gen 2 isoform 6 1 MNKIYRIVWNHAKRTWIVASELSRSANKSNATN----------TSLTTNIIKLSTLSLALSAGFASAATYSPTYGTIPDNNPQTSIAIGDGSKVNSTLVS 90
Adhesin B Gen 2 isoform 1 1 MKGSGLATVGAPETGDNGQTIYTVDVAKAGAPTVTRG------------------NVADAINNSEKTSSVVAGSKAVTVKAGKEDDKGNTEYTVDVATD- 81
Adhesin B Gen 2 isoform 2 1 MKGSGLATVGAPETGDNGQTIYTVDVAKAGAPTVTRG------------------NVADAINNSEKTSSVVAGSKAVTVKAGKEDDKGNTEYTVDVATD- 81
Adhesin B Gen 2 isoform 3 1 MKGSGLATVGAPETGDNGQTIYTVDVAKAGAPTVTRG------------------NVADAINNSEKTSSVVAGSKAVTVKAGKEDDKGNTEYTVDVATD- 81
Adhesin B Gen 2 isoform 4 1 MKGSGLATVGAPETGDNGQTIYTVDVAKAGAPTVTRG------------------NVADAINNSEKTSSVVAGSKAVTVKAGKEDDKGNTEYTVDVATD- 81
Adhesin B Gen 2 isoform 5 1 MKGSGLATVGAPETGDNGQTIYTVDVAKAGAPTVTRGNVAVKEGDENK--VMTAGDVADAINNSEKTSSVVAGSKAVTVKAGKEDDKGNTEYTVDVATD- 97
Adhesin G Gen 2 isoform 1 101 DSITNPYNYFNPGSLSYADRTKLQESNNSVNKEKDSRLLYKSNTTSAIVIGHNAVALDNNRKSRGKPKASGIAIGDYSYSGGGLSIAIGSFSRTASIGSV 200
Adhesin G Gen 2 isoform 3 101 DSITNLYNYFNPGSLSYADRTKLQESNNSVNKEKDSRLLYKSNTTSAIVIGHNAVALDNNRKSRGKPKASGIAIGDYSYSGGGLSIAIGSFSRTASIGSV 200
Adhesin G Gen 2 isoform 4 101 DSITNPYNYFNPGSLSYADRTKLQESNNSVNKEKDSRLLYKSNTTSAIVIGHNAVALDNNRKSRGKPKASGIAIGDYSYSGGGLSIAIGSFSRTASIGSV 200
Adhesin G Gen 2 isoform 5 101 DSITNPYNYFNPGSLSYADRTKLQESNNSVNKEKDSRLLYKSNTTSAIVIGHNAVALDNNRKSRGKPKASGIAIGDYSYSGGGLSIAIGSFSRTASIGSV 200
Adhesin G Gen 2 isoform 6 101 DSITNPYNYFNPGSLSYADRTKLQESNNSVNKEKDSRLLYKSNTTSAIVIGHNAVALDNNRKSRGKPKASGIAIGDYSYSGGGLSIAIGSFSRTASIGSV 200
Adhesin G Gen 2 isoform 7 101 DSITNPYNYFNPGSLSYADRTKLQESNNSVNKEKDSRLLYKSNTTSAIVIGHNAVALDNNRKSRGKPKASGIAIGDYSYSGGGLSIAIGSFSRTASIGSV 200
Adhesin G Gen 2 isoform 8 101 DSITNPYNYFNPGSLSYADRTKLQESNNSVNKEKDSRLLYKSNTTSAIVIGHNAVALDNNRKSRGKPKASGIAIGDYSYSGGGLSIAIGSFSRTASIGSV 200
Adhesin G Gen 2 isoform 9 101 DSITNPYNYFNPGSLSYADRTKLQESNNSVNKEKDSRLLYKSNTTSAIVIGHNAVALDNNRKSRGKPKASGIAIGDYSYSGGGLSIAIGSFSRTASIGSV 200
Adhesin G Gen 2 isoform 10 101 DSITNPYNYFNPGSLSYADRTKLQESNNSVNKEKDSRLLYKSNTTSAIVIGHNAVALDNNRKSRGKPKASGIAIGDYSYSGGGLSIAIGSFSRTASIGSV 200
Adhesin G Gen 2 isoform 12 101 DSITNPYNYFNPGSLSYADRTKLQESNNSVNKEKDSRLLYKSNTTSAIVIGHNAVALDNNRKSRGKPKASGIAIGDYSYSGGGLSIAIGSFSRTASIGSV 200
Adhesin G Gen 2 isoform 13 101 DSITNPYNYFNPGSLSYADRTKLQESNNSVNKEKDSRLLYKSNTTSAIVIGHNAVALDNNRKSRGKPKASGIAIGDYSYSGGGLSIAIGSFSRTASIGSV 200
Adhesin G Gen 2 isoform 14 101 DSITNPYNYFNPGSLSYADRTKLQESNNSVNKEKDSRLLYKSNTTSAIVIGHNAVALDNNRKSRGKPKASGIAIGDYSYSGGGLSIAIGSFSRTASIGSV 200
Adhesin G Gen 2 isoform 15 101 DSITNPYNYFNPGSLSYADRTKLQESNNSVNKEKDSRLLYKSNTTSAIVIGHNAVALDNNRKSRGKPKASGIAIGDYSYSGGGLSIAIGSFSRTASIGSV 200
Adhesin G Gen 2 isoform 16 101 DSITNPYNYFNPGSLSYADRTKLQESNNSVNKEKDSRLLYKSNTTSAIVIGHNAVALDNNRKSRGKPKASGIAIGDYSYSGGGLSIAIGSFSRTASIGSV 200
Adhesin G Gen 2 isoform 17 101 DSITNPYNYFNPGSLSYADRTKLQESNNSVNKEKDSRLLYKSNTTSAIVIGHNAVALDNNRKSRGKPKASGIAIGDYSYSGGGLSIAIGSFSRTASIGSV 200
Adhesin G Gen 2 isoform 18 101 DSITNPYNYFNPGSLSYADRTKLQESNNSVNKEKDSRLLYKSNTTSAIVIGHNAVALDNNRKSREKPKASGIAIGDYSYSGGGLSIAIGSFSRTASIGSV 200
Adhesin G Gen 2 isoform 19 101 DSITNPYNYFNPGSLSYADRTKLQESNNSVNKEKDSRLLYKSNTTSAIVIGHNAVALDNNRKSRGKPKASGIAIGDYSYSGGGLSIAIGSFSRTASIGSV 200
Adhesin G Gen 2 isoform 20 101 DSITNPYNYFNPGSLSYADRTKLQESNNSVNKEKDSRLLYKSNTTSAIVIGHNAVALDNNRKSRGKPKASGIAIGDYSYSGGGLSIAIGSFSRTASIGSV 200
Adhesin G isoform observed in CP0175216 91 TITG---LEGNSGKVSTALGYNTTATADFSTALGGFAN-ASFAST 131
Adhesin D Gen 2 isoform 1 91 TITG---LEGNSGKVSTALGYNTTATADFSTALGGFAN-ASFAST 131
Adhesin D Gen 2 isoform 2 91 TITG---LEGNSGKVSTALGYNTTATADFSTALGGFAN-ASFAST 131
Adhesin D Gen 2 isoform 3 91 TITG---LEGNSGKVSTALGYNTTATADFSTALGGFAN-ASFAST 131
Adhesin D Gen 2 isoform 4 91 TITG---LEGNSGKVSTALGYNTTATADFSTALGGFAN-ASFAST 131
Adhesin D Gen 2 isoform 6 91 TITG---LEGNSGKVSTALGYNTTATADFSTALGGFAN-ASFAST 131
Adhesin B Gen 2 isoform 1 82 --KSIGRDNDGNMTVNTDNVTIVKDPTTGEVKANTTTLNNS 120
Adhesin B Gen 2 isoform 2 82 --KSIGRDNDGNMTVNTDNVTIVKDPTTGEVKANTTTLNNS 120
Adhesin B Gen 2 isoform 3 82 --KSIGRDNDGNMTVNTDNVTIVKDPTTGEVKANTTTLNNS 120
Adhesin B Gen 2 isoform 4 82 --KSIGRDNDGNMTVNTDNVTIVKDPTTGEVKANTTTLNNS 120
Adhesin B Gen 2 isoform 5 98 --KSIGRDNDGNMTVNTDNVTIVKDPTTGEVKANTTTLNNS 136

Fig S14 continued

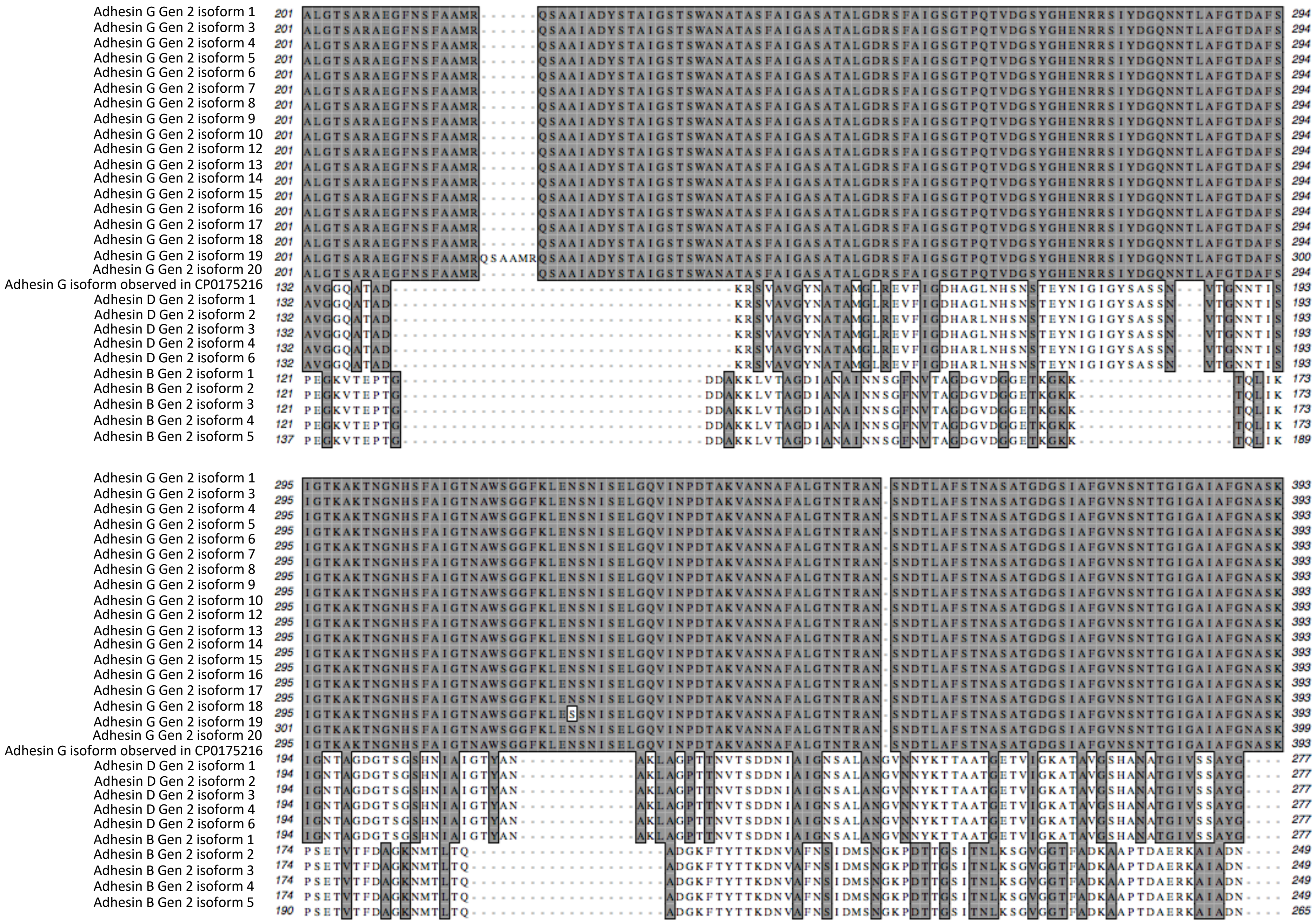

Fig S14 continued

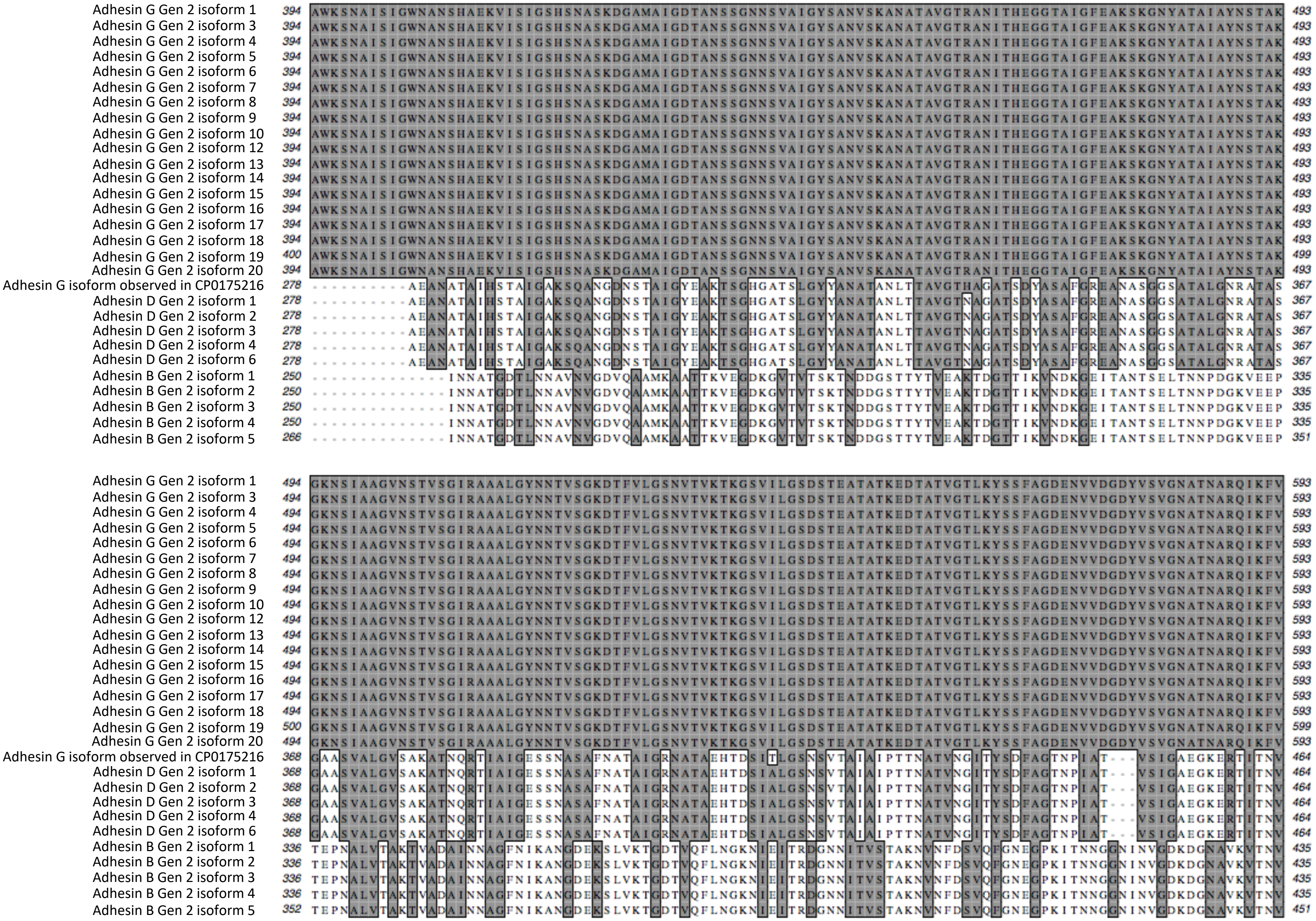

Fig S14 continued

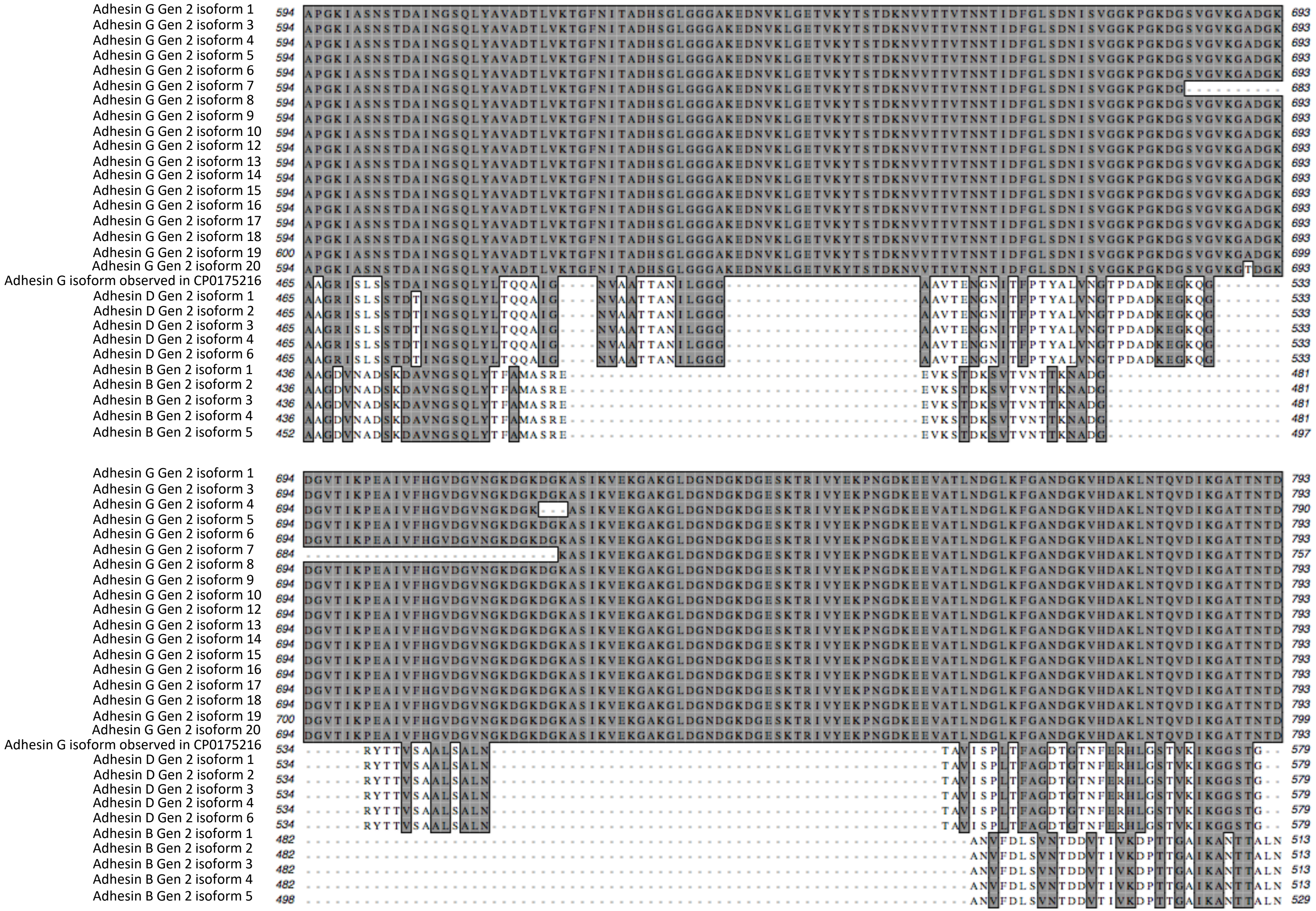

Fig S14 continued

```
Adhesin G Gen 2 isoform 1                  794 WAKFDAGKNIMTQVVGNTITVALAKDLAGLDSATFGTQGDSTVINKDGVTIAKKDPNDATKSNVTLTEDGLSNGGNQITNVDSGLKGKDGQPVKLTEAAG 893
Adhesin G Gen 2 isoform 3                  794 WAKFDAGKNIMTQVVGNTITVALAKDLAGLDSATFGTQGDSTVINKDGVTIAKKDPNDATKSNVTLTEDGLSNGGNQITNVDSGLKGKDGQPVKLTEAAG 893
Adhesin G Gen 2 isoform 4                  791 WAKFDAGKNIMTQVVGNTITVALAKDLAGLDSATFGTQGDSTVINKDGVTIAKKDPNDATKSNVTLTEDGLSNGGNQITNVDSGLKGKDGQPVKLTEAAG 890
Adhesin G Gen 2 isoform 5                  794 WAKFDAGKNIMTQVVGNTITVALAKDLAGLDSATFGTQGDSTVINKDGVTIAKKDPNDATKSNVTLTEDGLSNGGNQITNVDSGLKGKDGQPVKLTEAAG 893
Adhesin G Gen 2 isoform 6                  794 WAKFDAGKNIMTQVVGNTITVALAKDLAGLDSATFGTQGDSTVINKDGVTIAKKDPNDATKSNVTLTEDGLSNGGNQITNVDSGLKGKDGQPVKLTEAAG 893
Adhesin G Gen 2 isoform 7                  758 WAKFDAGKNIMTQVVGNTITVALAKDLAGLDSATFGTQGDSTVINKDGVTIAKKDPNDATKSNVTLTEDGLSNGGNQITNVDSGLKGKDGQPVKLTEAAG 857
Adhesin G Gen 2 isoform 8                  794 WAKFDAGKNIMTQVVGNTITVALAKDLAGLDSATFGTQGDSTVINKDGVTIAKKDPNDATKSNVTLTEDGLSNGGNQITNVDSGLKGKDGQPVKLTEAAG 893
Adhesin G Gen 2 isoform 9                  794 WAKFDAGKNIMTQVVGNTITVALAKDLAGLDSATFGTQGDSTVINKDGVTIAKKDPNDATKSNVTLTEDGLSNGGNQITNVDSGLKGKDGQPVKLTEAAG 893
Adhesin G Gen 2 isoform 10                 794 WAKFDAGKNIMTQVVGNTITVALAKDLAGLDSATFGTQGDSTVINKDGVTIAKKDPNDATKSNVTLTEDGLSNGGNQITNVDSGLKGKDGQPVKLTEAAG 893
Adhesin G Gen 2 isoform 12                 794 WAKFDAGKNIMTQVVGNTITVALAKDLAGLDSATFGTQGDSTVINKDGVTIAKKDPNDATKSNVTLTEDGLSNGGNQITNVDSGLKGKDGQPVKLTEAAG 893
Adhesin G Gen 2 isoform 13                 794 WAKFDAGKNIMTQVVGNTITVALAKDLAGLDSATFGTQGDSTVINKDGVTIAKKDPNDATKSNVTLTEDGLSNGGNQITNVDSGLKGKDGQPVKLTEAAG 893
Adhesin G Gen 2 isoform 14                 794 WAKFDAGKNIMTQVVGNTITVALAKDLAGLDSATFGTQGDSTVINKDGVTIAKKDPNDATKSNVTLTEDGLSNGGNQITNVDSGLKGKDGQPVKLTEAAG 893
Adhesin G Gen 2 isoform 15                 794 WAKFDAGKNIMTQVVGNTITVALAKDLAGLDSATFGTQGDSTVINKDGVTIAKKDPNDATKSNVTLTEDGLSNGGNQITNVDSGLKGKDGQPVKLTEAAG 893
Adhesin G Gen 2 isoform 16                 794 WAKFDAGKNIMTQVVGNTITVALAKDLAGLDSATFGTQGDSTVINKDGVTIAKKDPNDATKSNVTLTEDGLSNGGNQITNVDSGLKGKDGQPVKLTEAAG 893
Adhesin G Gen 2 isoform 17                 794 WAKFDAGKNIMTQVVGNTITVALAKDLAGLDSATFGTQGDSTVINKDGVTIAKKDPNDATKSNVTLTEDGLSNGGNQITNVDSGLKGKDGQPVKLTEAAG 893
Adhesin G Gen 2 isoform 18                 794 WAKFDAGKNIMTQVVGNTITVALAKDLAGLDSATFGTQGDSTVINKDGVTIAKKDPNDATKSNVTLTEDGLSNGGNQITNVDSGLKGKDGQPVKLTEAAG 893
Adhesin G Gen 2 isoform 19                 800 WAKFDAGKNIMTQVVGNTITVALAKDLAGLDSATFGTQGDSTVINKDGVTIAKKDPNDATKSNVTLTEDGLSNGGNQITNVDSGLKGKDGQPVKLTEAAG 899
Adhesin G Gen 2 isoform 20                 794 WAKFDAGKNIMTQVVGNTITVALAKDLAGLDSATFGTQGDSTVINKDGVTIAKKDPNDATKSNVTLTEDGLSNGGNQITNVDSGLKGKDGQPVKLTEAAG 893
Adhesin G isoform observed in CP0175216    580 -ILTENNIGVVADGN-STLTIKLAEKVNLGANGSLTTG--DTVVNNTGITIAN----GVADKPVSLTKSGLDNGGNKIANVAA----------------G 655
Adhesin D Gen 2 isoform 1                  580 -ILTENNIGVVADGN-STLTIKLAEKVNLGANGSLTTG--DTVVNNTGITIAN----GVADKPVSLTKSGLDNGGNKIANVAA----------------G 655
Adhesin D Gen 2 isoform 2                  580 -ILTENNIGVVADGN-STLTIKLAEKVNLGANGSLTTG--DTVVNNTGITIAN----GVADKPVSLTKSGLDNGGNKIANVAA----------------G 655
Adhesin D Gen 2 isoform 3                  580 -ILTENNIGVVADGN-STLTIKLAEKVNLGANGSLTTG--DTVVNNTGITIAN----GVADKPVSLTKSGLDNGGNKIANVAA----------------G 655
Adhesin D Gen 2 isoform 4                  580 -ILTENNIGVVADGN-STLTIKLAEKVNLGANGSLTTG--DTVVNNTGITIAN----GVADKPVSLTKSGLDNGGNKIANVAA----------------G 655
Adhesin D Gen 2 isoform 6                  580 -ILTENNIGVVADGNNSTLTIKLAEKVNLGANGSLTTG--DTVVNNTGITIAN----GVADKPVSLTKSGLDNGGNKIANVAA----------------G 656
Adhesin B Gen 2 isoform 1                  514 DANNDG---------------RIDEPTADDAKKLVTAGDITNAINNSGFTLKTS---AVEGGEKLSGGDELINPGKAVEMVAG------KNLTVKQEADG 589
Adhesin B Gen 2 isoform 2                  514 DANNDG---------------RIDEPTADDAKKLVTAGDITNAINNSGFTLKTS---AVEGGEKLSGGDELINPGKAVEMVAG------KNLTVKQEADG 589
Adhesin B Gen 2 isoform 3                  514 DANNDG---------------RIDEPTADDAKKLVTAGDITNAINNSGFTLKTS---AVEGGEKLSGGDELINPGKAVEMVAG------KNLTVKQEADG 589
Adhesin B Gen 2 isoform 4                  514 DANNDG---------------RIDEPTADDAKKLVTAGDITNAINNSGFTLKTS---AVEGGEKLSGGDELINPGKAVEMVAG------KNLTVKQEADG 589
Adhesin B Gen 2 isoform 5                  530 DANNDG---------------RIDEPTADDAKKLVTAGDITNAINNSGFTLKTS---AVEGGEKLSGGDELINPGKAVEMVAG------KNLTVKQEADG 605

Adhesin G Gen 2 isoform 1                  894 DVLNNAVNVGDLKNTVTETVTEVTTDAFGLKDENNKEFKQNLGTTAKIIGDGNVKTNVVEKDGGHALEVSLVNNITVGKDGEPGKVGIAGKDGKDAIALD 993
Adhesin G Gen 2 isoform 3                  894 DVLNNAVNVGDLKNTVTETVTEVTTDAFGLKDENNKEFKQNLGTTAKIIGDGNVKTNVVEKDGGHALEVSLVNNITVGKDGEPGKVGIAGKDGKDAIALD 993
Adhesin G Gen 2 isoform 4                  891 DVLNNAVNVGDLKNTVTETVTEVTTDAFGLKDENNKEFKQNLGTTAKIIGDGNVKTNVVEKDGGHALEVSLVNNITVGKDGEPGKVGIAGKDGKDAIALD 990
Adhesin G Gen 2 isoform 5                  894 DVLNNAVNVGDLKNTVTETVTEVTTDAFGLKDENNKEFKQNLGTTAKIIGDGNVKTNVVEKDGGHALEVSLVNNITVGKDGEPGKVGIAGKDGKDAIALD 993
Adhesin G Gen 2 isoform 6                  894 DVLNNAVNVGDLKNTVTETVTEVTTDAFGLKDENNKEFKQNLGTTAKIIGDGNVKTNVVEKDGGHALEVSLVNNITVGKDGEPGKVGIAGKDGKDAIALD 993
Adhesin G Gen 2 isoform 7                  858 DVLNNAVNVGDLKNTVTETVTEVTTDAFGLKDENNKEFKQNLGTTAKIIGDGNVKTNVVEKDGGHALEVSLVNNITVGKDGEPGKVGIAGKDGKDAIALD 957
Adhesin G Gen 2 isoform 8                  894 DVLNNAVNVGDLKNTVTETVTEVTTDAFGLKDENNKEFKQNLGTTAKIIGDGNVKTNVVEKDGGHALEVSLVNNITVGKDGEPGKVGIAGKDGKDAIALD 993
Adhesin G Gen 2 isoform 9                  894 DVLNNAVNVGDLKNTVTETVTEVTTDAFGLKDENNKEFKQNLGTTAKIIGDGNVKTNVVEKDGGHALEVSLVNNITVGKDGEPGKVGIAGKDGKDAIALD 993
Adhesin G Gen 2 isoform 10                 894 DVLNNAVNVGDLKNTVTETVTEVTTDAFGLKDENNKEFKQNLGTTAKIIGDGNVKTNVVEKDGGHALEVSLVNNITVGKDGEPGKVGIAGKDGKDAIALD 993
Adhesin G Gen 2 isoform 12                 894 DVLNNAVNVGDLKNTVTETVTEVTTDAFGLKDENNKEFKQNLGTTAKIIGDGNVKTNVVEKDGGHALEVSLVNNITVGKDGEPGKVGIAGKDGKDAIALD 993
Adhesin G Gen 2 isoform 13                 894 DVLNNAVNVGDLKNTVTETVTEVTTDAFGLKDENNKEFKQNLGTTAKIIGDGNVKTNVVEKDGGHALEVSLVNNITVGKDGEPGKVGIAGKDGKDAIALD 993
Adhesin G Gen 2 isoform 14                 894 DVLNNAVNVGDLKNTVTETVTEVTTDAFGLKDENNKEFKQNLGTTAKIIGDGNVKTNVVEKDGGHALEVSLVNNITVGKDGEPGKVGIAGKDGKDAIALD 993
Adhesin G Gen 2 isoform 15                 894 DVLNNAVNVGDLKNTVTETVTEVTTDAFGLKDENNKEFKQNLGTTAKIIGDGNVKTNVVEKDGGHALEVSLVNNITVGKDGEPGKVGIAGKDGKDAIALD 993
Adhesin G Gen 2 isoform 16                 894 DVLNNAVNVGDLKNTVTETVTEVTTDAFGLKDENNKEFKQNLGTTAKIIGDGNVKTNVVEKDGGHALEVSLVNNITVGKDGEPGKVGIAGKDGKDAIALD 993
Adhesin G Gen 2 isoform 17                 894 DVLNNAVNVGDLKNTVTETVTEVTTDAFGLKDENNKEFKQNLGTTAKIIGDGNVKTNVVEKDGGHALEVSLVNNITVGKDGEPGKVGIAGKDGKDAIALD 993
Adhesin G Gen 2 isoform 18                 894 DVLNNAVNVGDLKNTVTETVTEVTTDAFGLKDENNKEFKQNLGTTAKIIGDGNVKTNVVEKDGGHALEVSLVNNITVGKDGEPGKVGIAGKDGKDAIALD 993
Adhesin G Gen 2 isoform 19                 900 DVLNNAVNVGDLKNTVTETVTEVTTDAFGLKDENNKEFKQNLGTTAKIIGDGNVKTNVVEKDGGHALEVSLVNNITVGKDGEPGKVGIAGKDGKDAIALD 999
Adhesin G Gen 2 isoform 20                 894 DVLNNAVNVGDLKNTVTETVTEVTTDAFGLKDENNKEFKQNLGTTAKIIGDGNVKTNVVEKDGGHALEVSLVNNITVGKDGEPGKVGIAGKDGKDAIALD 993
Adhesin G isoform observed in CP0175216    656 DVDTDAVNVSQLKQAISKFATHYVSISDDGIQRANYDNSGSSGVNSMAIGVATRANGELATALGSEAEANGERTTAVG---------------------- 733
Adhesin D Gen 2 isoform 1                  656 DVDTDAVNVSQLKQAISKFATHYVSISDDGIQRANYDNSGSSGVNPMAIGVATSANGELATALGSEAEANGERTTAVG---------------------- 733
Adhesin D Gen 2 isoform 2                  656 DVDTDAVNVSQLKQAISKFATHYVSISDDGIQRANYDNSGSSGVNPMAIGVATSANGELATALGSEAEANGERTTAVG---------------------- 733
Adhesin D Gen 2 isoform 3                  656 DVDTDAVNVSQLKQAISKFATHYVSISDDGIQRANYDNSGSSGVNPMAIGVATSANGELATALGSEAEANGERTTAVG---------------------- 733
Adhesin D Gen 2 isoform 4                  656 DVDTDAVNVSQLKQAISKFATHYVSISDDGIQRANYDNSGSSGVNPMAIGVATSANGELATALGSEAEANGERTTAVG---------------------- 733
Adhesin D Gen 2 isoform 6                  657 DVDTDAVNVSQLKQAISKFATHYVSISDDGIQRANYDNSGSSGVNPMAIGVATSANGELATALGSEAEANGERTTAVG---------------------- 734
Adhesin B Gen 2 isoform 1                  590 KVIYATKDDVKFSSVTSNTVTVPTDEADP---ANNPITINKDGINAGNKAISNVASNLIPVTADDKVQPAD----------------------------- 657
Adhesin B Gen 2 isoform 2                  590 KVIYATKDDVKFSSVTSNTVTVPTDEADP---ANNPITINKDGINAGNKAISNVASNLIPVTADDKVQPAD----------------------------- 657
Adhesin B Gen 2 isoform 3                  590 KVIYATKDDVKFSSVTSNTVTVPTDEADP---ANNPITINKDGINAGNKAISNVASNLIPVTADDKVQPAD----------------------------- 657
Adhesin B Gen 2 isoform 4                  590 KVIYATKDDVKFSSVTSNTVTVPTDEADP---ANNPITINKDGINAGNKAISNVASNLIPVTADDKVQPAD----------------------------- 657
Adhesin B Gen 2 isoform 5                  606 KVIYATKDDVKFSSVTSNTVTVPTDEADP---ANNPITINKDGINAGNKAISNVASNLIPVTADDKVQPAD----------------------------- 673
```

Fig S14 continued

```
Adhesin G Gen 2 isoform 1             994  GKDGAITVGRPGKDGIDGTIGVNGKDGSAVVLNGKDGSIGLNGTNGKDGLTIKGTEGKPGLVGKDGLPGKDGVTRIQYQPTDGNGDPIGEPEQVATLNDG  1093
Adhesin G Gen 2 isoform 3             994  GKDGAITVGRPGKDGIDGTIGVNGKDGSAVVLNGKDGSIGLNGTNGKDGLTIKGTEGKPGLVGKDGLPGKDGVTRIQYQPTDGNGDPIGEPEQVATLNDG  1093
Adhesin G Gen 2 isoform 4             991  GKDGAITVGRPGKDGIDGTIGVNGKDGSAVVLNGKDGSIGLNGTNGKDGLTIKGTEGKPGLVGKDGLPGKDGVTRIQYQPTDGNGDPIGEPEQVATLNDG  1090
Adhesin G Gen 2 isoform 5             994  GKDGAITVGRPGKDGIDGTIGVNGKDGSAVVLNGKDGSIGLNGTNGKDGLTIKGTEGKPGLVGKDGLPGKDGVTRIQYQPTDGNGDPIGEPEQVATLNDG  1093
Adhesin G Gen 2 isoform 6             994  GKDGAITVGRPGKDGIDGTIGVNGKDGSAVVLNGKDGSIGLNGTNGKDGLTIKGTEGKPGLVGKDGLPGKDGVTRIQYQPTDGNGDPIGEPEQVATLNDG  1093
Adhesin G Gen 2 isoform 7             958  GKDGAITVGRPGKDGIDGTIGVNGKDGSAVVLNGKDGSIGLNGTNGKDGLTIKGTEGKPGLVGKDGLPGKDGVTRIQYQPTDGNGDPIGEPEQVATLNDG  1057
Adhesin G Gen 2 isoform 8             994  GKDGAITVGRPGKDGIDGTIGVNGKDGSAVVLNGKDGSIGLNGTNGKDGLTIKGTEGKPGLVGKDGLPGKDGVTRIQYQPTDGNGDPIGEPEQVATLNDG  1093
Adhesin G Gen 2 isoform 9             994  GKDGAITVGRPGKDGIDGTIGVNGKDGSAVVLNGKDGSIGLNGTNGKDGLTIKGTEGKPGLVGKDGLPGKDGVTRIQYQPTDGNGDPIGEPEQVATLNDG  1093
Adhesin G Gen 2 isoform 10            994  GKDGAITVGRPGKDGIDGTIGVNGKDGSAVVLNGKDGSIGLNGTNGKDGLTIKGTEGKPGLVGKDGLPGKDGVTRIQYQPTDGNGDPIGEPEQVATLNDG  1093
Adhesin G Gen 2 isoform 12            994  GKDGAITVGRPGKDGIDGTIGVNGKDGSAVVLNGKDGSIGLNGTNGKDGLTIKGTEGKPGLVGKD----------------------------------G  1059
Adhesin G Gen 2 isoform 13            994  GKDGAITVGRPGKDGIDGTIGVNGKDGSAVVLNGKDGSIGLNGTNGKDGLTIKGTEGKPGLVGKDGLPGKDGVTRIQYQPTDGNGDPIGEPEQVATLNDG  1093
Adhesin G Gen 2 isoform 14            994  GKDGAITVGRPGKDGIDGTIGVNGKDGSAVVLNGKDGSIGLNGTNGKDGLTIKGTEGKPGLVGKDGLPGKDGVTRIQYQPTDGNGDPIGEPEQVATLNDG  1093
Adhesin G Gen 2 isoform 15            994  GKDGAITVGRPGKDGIDGTIGVNGKDGSAVVLNGKDGSIGLNGTNGKDGLTIKGTEGKPGLVGKDGLPGKDGVTRIQYQPTDGNGDPIGEPEQVATLNDG  1093
Adhesin G Gen 2 isoform 16            994  GKDGAITVGRPGKDGIDGTIGVNGKDGSAVVLNGKDGSIGLNGTNGKDGLTIKGTEGKPGLVGKDGLPGKDGVTRIQYQPTDGNGDPIGEPEQVATLNDG  1093
Adhesin G Gen 2 isoform 17            994  GKDGAITVGRPGKDGIDGTIGVNGKDGSAVVLNGKDGSIGLNGTNGKDGLTIKGTEGKPGLVGKDGLPGKDGVTRIQYQPTDGNGDPIGEPEQVATLNDG  1093
Adhesin G Gen 2 isoform 18            994  GKDGAITVGRPGKDGIDGTIGVNGKDGSAVVLNGKDGSIGLNGTNGKDGLTIKGTEGKPGLVGKDGLPGKDGVTRIQYQPTDGNGDPIGEPEQVATLNDG  1093
Adhesin G Gen 2 isoform 19           1000  GKDGAITVGRPGKDGIDGTIGVNGKDGSAVVLNGKDGSIGLNGTNGKDGLTIKGTEGKPGLVGKDGLPGKDGVTRIQYQPTDGNGDPIGEPEQVATLNDG  1099
Adhesin G Gen 2 isoform 20            994  GKDGAITVGRPGKDGIDGTIGVNGKDGSAVVLNGKDGSIGLNGTNGKDGLTIKGTEGKPGLVGKDGLPGKDGVTRIQYQPTDGNGDPIGEPEQVATLNDG  1093
Adhesin G isoform observed in CP0175216  734  --------PRATADGMN------------------ATSIGYN----------------------------------------------------------  749
Adhesin D Gen 2 isoform 1             734  --------PRATADGMN------------------ATSIGYN----------------------------------------------------------  749
Adhesin D Gen 2 isoform 2             734  --------PRATADGMN------------------ATSIGYN----------------------------------------------------------  749
Adhesin D Gen 2 isoform 3             734  --------PRATADGMN------------------ATSIGYN----------------------------------------------------------  749
Adhesin D Gen 2 isoform 4             734  --------PRATADGMN------------------ATSIGYN----------------------------------------------------------  749
Adhesin D Gen 2 isoform 6             735  --------PRATADGMN------------------ATSIGYN----------------------------------------------------------  750
Adhesin B Gen 2 isoform 1             658  ----------------------------------------------------------------------------------------------------  657
Adhesin B Gen 2 isoform 2             658  ----------------------------------------------------------------------------------------------------  657
Adhesin B Gen 2 isoform 3             658  ----------------------------------------------------------------------------------------------------  657
Adhesin B Gen 2 isoform 4             658  ----------------------------------------------------------------------------------------------------  657
Adhesin B Gen 2 isoform 5             674  ----------------------------------------------------------------------------------------------------  673

Adhesin G Gen 2 isoform 1            1094  LHFKGDDATLVSKKLNETLEIIGGADKDKLTANNIGVNSKDGKLSVQLAKDLKDLNSATFKPQDGENGQEGKGPTTVVNNNGVTITPKDGTDGKAVKLTE  1193
Adhesin G Gen 2 isoform 3            1094  LHFKGDDATLVSKKLNETLEIIGGADKDKLTANNIGVNSKDGKLSVQLAKDLKDLNSATFKPQDGENGQEGKGPTTVVNNNGVTITPKDGTDGKAVKLTE  1193
Adhesin G Gen 2 isoform 4            1091  LHFKGDDATLVSKKLNETLEIIGGADKDKLTANNIGVNSKDGKLSVQLAKDLKDLNSATFKPQDGENGQEGKGPTTVVNNNGVTITPKDGTDGKAVKLTE  1190
Adhesin G Gen 2 isoform 5            1094  LHFKGDDATLVSKKLNETLEIIGGADKDKLTANNIGVNSKDGKLSVQLAKDLKDLNSATFKPQDGENGQEGKGPTTVVNNNGVTITPKDGTDGKAVKLTE  1193
Adhesin G Gen 2 isoform 6            1094  LHFKGDDATLVSKKLNETLEIIGGADKDKLTANNIGVNSKDGKLSVQLAKDLKDLNSATFKPQDGENGQEGKGPTTVVNNNGVTITPKDGTDGKAVKLTE  1193
Adhesin G Gen 2 isoform 7            1058  LHFKGDDATLVSKKLNETLEIIGGADKDKLTANNIGVNSKDGKLSVQLAKDLKDLNSATFKPQDGENGQEGKGPTTVVNNNGVTITPKDGTDGKAVKLTE  1157
Adhesin G Gen 2 isoform 8            1094  LHFKGDDATLVSKKLNETLEIIGGADKDKLTANNIGVNSKDGKLSVQLAKDLKDLNSATFKPQDGENGQEGKGPTTVVNNNGVTITPKDGTDGKAVKLTE  1193
Adhesin G Gen 2 isoform 9            1094  LHFKGDDATLVSKKLNETLEIIGGADKDKLTANNIGVNSKDGKLSVQLAKDLKDLNSATFKPQDGENGQEGKGPTTVVNNNGVTITPKDGTDGKAVKLTE  1193
Adhesin G Gen 2 isoform 10           1094  LHFKGDDATLVSKKLNETLEIIGGADKDKLTANNIGVNSKDGKLSVQLAKDLKDLNSATFKPQDGENGQEGKGPTTVVNNNGVTITPKDGTDGKAVKLTE  1193
Adhesin G Gen 2 isoform 12           1060  LHFKGDDATLVSKKLNETLEIIGGADKDKLTANNIGVNSKDGKLSVQLAKDLKDLNSATFKPQDGENGQEGKGPTTVVNNNGVTITPKDGTDGKAVKLTE  1159
Adhesin G Gen 2 isoform 13           1094  LHFKGDDATLVSKKLNETLEIIGGADKDKLTANNIGVNSKDGKLSVQLAKDLKDLNSATFKPQDGENGQEGKGPTTVVNNNGVTITPKDGTDGKAVKLTE  1193
Adhesin G Gen 2 isoform 14           1094  LHFKGDDATLVSKKLNETLEIIGGADKDKLTANNIGVNSKDGKLSVQLAKDLKDLNSATFKPQDGENGQEGKGPTTVVNNNGVTITPKDGTDGKAVKLTE  1193
Adhesin G Gen 2 isoform 15           1094  LHFKGDDATLVSKKLNETLEIIGGADKDKLTANNIGVNSKDGKLSVQLAKDLKDLNSATFKPQDGENGQEGKGPTTVVNNNGVTITPKDGTDGKAVKLTE  1193
Adhesin G Gen 2 isoform 16           1094  LHFKGDDATLVSKKLNETLEIIGGADKDKLTANNIGVNSKDGKLSVQLAKDLKDLNSATFKPQDGENGQEGKGPTTVVNNNGVTITPKDGTDGKAVKLTE  1193
Adhesin G Gen 2 isoform 17           1094  LHFKGDDATLVSKKLNETLEIIGGADKDKLTANNIGVNSKDGKLSVQLAKDLKDLNSATFKPQDGENGQEGKGPTTVVNNNGVTITPKDGTDGKAVKLTE  1193
Adhesin G Gen 2 isoform 18           1094  LHFKGDDATLVSKKLNETLEIIGGADKDKLTANNIGVNSKDGKLSVQLAKDLKDLNSATFKPQDGENGQEGKGPTTVVNNNGVTITPKDGTDGKAVKLTE  1193
Adhesin G Gen 2 isoform 19           1100  LHFKGDDATLVSKKLNETLEIIGGADKDKLTANNIGVNSKDGKLSVQLAKDLKDLNSATFKPQDGENGQEGKGPTTVVNNNGVTITPKDGTDGKAVKLTE  1199
Adhesin G Gen 2 isoform 20           1094  LHFKGDDATLVSKKLNETLEIIGGADKDKLTANNIGVNSKDGKLSVQLAKDLKDLNSATFKPQDGENGQEGKGPTTVVNNNGVTITPKDGTDGKAVKLTE  1193
Adhesin G isoform observed in CP0175216  750  ----------ANANATNALAVGSAANANADTSTAIGTAS--------TATATRATALGSKSEATGENSTAVGYEASSIGADSLAAGYNANASGTQSTALG  831
Adhesin D Gen 2 isoform 1             750  ----------ANANATNALAVGSAANANADTSTAIGTAS--------TATATRATALGSKSEATGENSTAVGYEASSIGADSLAAGYNANASGTQSTALG  831
Adhesin D Gen 2 isoform 2             750  ----------ANANATNALAVGSAANANADTSTAIGTAS--------TATATRATALGSKSEATGENSTAVGYEASSIGADSLAAGYNANASGTQSTALG  831
Adhesin D Gen 2 isoform 3             750  ----------ANANATNALAVDSAANANADTSTAIGTAS--------TATATRATALGSKSEATGENSTAVGYEASSIGADSLAAGYNANASGTQSTALG  831
Adhesin D Gen 2 isoform 4             750  ----------ANANATNALAVGSAANANADTSTAIGTAS--------TATATRATALGSKSEATGENSTAVGYEASSIGADSLAAGYNANASGTQSTALG  831
Adhesin D Gen 2 isoform 6             751  ----------ANANATNALAVGSAANANADTSTAIGTAS--------TATATRATALGSKSEATGENSTAVGYEASSIGADSLAAGYNANASGTQSTALG  832
Adhesin B Gen 2 isoform 1             658  -----------NNPTNLADKLSNAATLGDVLNAGWNLQGNGKAVDTVVHNDTVDFINGKGTTVTVENKD---GKNTIKVDSPIEFVNQDPTDSSTPSNTA  743
Adhesin B Gen 2 isoform 2             658  -----------NNPTNLADKLSNAATLGDVLNAGWNLQGNGKAVDTVVHNDTVDFINGKGTTVTVENKD---GKNTIKVDSPIEFVNQDPTDSSTPSNTA  743
Adhesin B Gen 2 isoform 3             658  -----------NNPTNLADKLSNAATLGDVLNAGWNLQGNGKAVDTVVHNDTVDFINGKGTTVTVENKD---GKNTIKVDSPIEFVNQDPTDSSTPSNTA  743
Adhesin B Gen 2 isoform 4             658  -----------NNPTNLADKLSNAATLGDVLNAGWNLQGNGKAVDTVVHNDTVDFINGKGTTVTVENKD---GKNTIKVDSPIEFVNQDPTDSSTPSNTA  743
Adhesin B Gen 2 isoform 5             674  -----------NNPTNLADKLSNAATLGDVLNAGWNLQGNGKAVDTVVHNDTVDFINGKGTTVTVENKD---GKNTIKVDSPIEFVNQDPTDSSTPSNTA  759
```

Fig S14 continued

```
Adhesin G Gen 2 isoform 1                   1194 DGLDNGGNQIVNVKSGIDGLAGKDGINGKDGKPITSVNDLTDDQLKEVGTNAANINDVVNASNDIIEKGFSIAADNGETDHVKLGETVKYTSKDKNIITT 1293
Adhesin G Gen 2 isoform 3                   1194 DGLDNGGNQIVNVKSGIDGLAGKDGINGKDGKPITSVNDLTDDQLKEVGTNAANINDVVNASNDIIEKGFSIAADNGETDHVKLGETVKYTSKDKNIITT 1293
Adhesin G Gen 2 isoform 4                   1191 DGLDNGGNQIVNVKSGIDGLAGKDGINGKDGKPITSVNDLTDDQLKEVGTNAANINDVVNASNDIIEKGFSIAADNGETDHVKLGETVKYTSKDKNIITT 1290
Adhesin G Gen 2 isoform 5                   1194 DGLDNGGNQIVNVKSGIDGLAGKDGINGKDGKPITSVNDLTDDQLKEVGTNAANINDVVNASNDIIEKGFSIAADNGETDHVKLGETVKYTSKDKNIITT 1293
Adhesin G Gen 2 isoform 6                   1194 DGLDNGGNQIVNVKSGIDGLAGKDGINGKDGKPITSVNDLTDDQLKEVGTNAANINDVVNASNDIIEKGFSIAADNGETDHVKLGETVKYTSKDKNIITT 1293
Adhesin G Gen 2 isoform 7                   1158 DGLDNGGNQIVNVKSGIDGLAGKDGINGKDGKPITSVNDLTDDQLKEVGTNAANINDVVNASNDIIEKGFSIAADNGETDHVKLGETVKYTSKDKNIITT 1257
Adhesin G Gen 2 isoform 8                   1194 DGLDNGGNQIVNVKSGIDGLAGKDGINGKDGKPITSVNDLTDDQLKEVGTNAANINDVVNASNDIIEKGFSIAADNGETDHVKLGETVKYTSKDKNIITT 1293
Adhesin G Gen 2 isoform 9                   1194 DGLDNGGNQIVNVKSGIDGLAGKDGINGKDGKPITSVNDLTDDQLKEVGTNAANINDVVNASNDIIEKGFSIAADNGETDHVKLGETVKYTSKDKNIITT 1293
Adhesin G Gen 2 isoform 10                  1194 DGLDNGGNQIVNVKSGIDGLAGKDGINGKDGKPITSVNDLTDDQLKEVGTNAANINDVVNASNDIIEKGFSIAADNGETDHVKLGETVKYTSKDKNIITT 1293
Adhesin G Gen 2 isoform 12                  1160 DGLDNGGNQIVNVKSGIDGLAGKDGINGKDGKPITSVNDLTDDQLKEVGTNAANINDVVNASNDIIEKGFSIAADNGETDHVKLGETVKYTSKDKNIITT 1259
Adhesin G Gen 2 isoform 13                  1194 DGLDNGGNQIVNVKSGIDGLAGKDGINGKDGKPITSVNDLTDDQLKEVGTNAANINDVVNASNDIIEKGFSIAADNGETDHVKLGETVKYTSKDKNIITT 1293
Adhesin G Gen 2 isoform 14                  1194 DGLDNGGNQIVNVKSGIDGLAGKDGINGKDGKPITSVNDLTDDQLKEVGTNAANINDVVNASNDIIEKGFSIAADNGETDHVKLGETVKYTSKDKNIITT 1293
Adhesin G Gen 2 isoform 15                  1194 DGLDNGGNQIVNVKSGIDGLAGKDGINGKDGKPITSVNDLTDDQLKEVGTNAANINDVVNASNDIIEKGFSIAADNGETDHVKLGETVKYTSKDKNIITT 1293
Adhesin G Gen 2 isoform 16                  1194 DGLDNGGNQIVNVKSGIDGLAGKDGINGKDGKPITSVNDLTDDQLKEVGTNAANINDVVNASNDIIEKGFSIAADNGETDHVKLGETVKYTSKDKNIITT 1293
Adhesin G Gen 2 isoform 17                  1194 DGLDNGGNQIVNVKSGIDGLAGKDGINGKDGKPITSVNDLTDDQLKEVGTNAANINDVVNASNDIIEKGFSIAADNGETDHVKLGETVKYTSKDKNIITT 1293
Adhesin G Gen 2 isoform 18                  1194 DGLDNGGNQIVNVKSGIDGLAGKDGINGKDGKPITSVNDLTDDQLKEVGTNAANINDVVNASNDIIEKGFSIAADNGETDHVKLGETVKYTSKDKNIITT 1293
Adhesin G Gen 2 isoform 19                  1200 DGLDNGGNQIVNVKSGIDGLAGKDGINGKDGKPITSVNDLTDDQLKEVGTNAANINDVVNASNDIIEKGFSIAADNGETDHVKLGETVKYTSKDKNIITT 1299
Adhesin G Gen 2 isoform 20                  1194 DGLDNGGNQIVNVKSGIDGLAGKDGINGKDGKPITSVNDLTDDQLKEVGTNAANINDVVNASNDIIEKGFSIAADNGETDHVKLGETVKYTSKDKNIITT 1293
Adhesin G isoform observed in CP0175216      832 NSANAGGIWSTSVGRNANAAGSSAIALGNSANAAG------------VASIALGVSSQATTTAAVALGQNAKATHQGSVALGTNSATVATVATKSATLNG 919
Adhesin D Gen 2 isoform 1                    832 NSANAGGIWSTSVGRNANAAGSSAIALGNSANAAG------------VASIALGVSSQATTTAAVALGQNAKATHQGSVALGTNSETVATVATKSATLNG 919
Adhesin D Gen 2 isoform 2                    832 NSANAGGIWSTSVGRNANAAGSSAIALGNSANAAG------------VASIALGVSSQATTTAAVALGQNAKATHQGSVALGTNSETVATVATKSATLNG 919
Adhesin D Gen 2 isoform 3                    832 NSANAGGIWSTSVGRNANAAGSSAIALGNSANAAG------------VASIALGVSSQATTTAAVALGQNAKATHQGSVALGTNSETVATVATKSATLNG 919
Adhesin D Gen 2 isoform 4                    832 NSANAGGIWSTSVGRNANAAGSSAIALGNSANAAG------------VASIALGVSSQATTTAAVALGQNAKATHQGSVALGTNSETVATVATKSATLNG 919
Adhesin D Gen 2 isoform 6                    833 NSANAGGIWSTSVGRNANAAGSSAIALGNSANAAG------------VASIALGVSSQATTTAAVALGQNAKATHQGSVALGTNSETVATVATKSATLNG 920
Adhesin B Gen 2 isoform 1                    744 KFTGEAPVQLGNVASSVRNEDGSTPEGKDRAEAIK--------------------NAEGDKLNNVVNLGDLQAATNAATTKVGGNRGVTITPS-TNADGS 822
Adhesin B Gen 2 isoform 2                    744 KFTGEAPVQLGNVASSVRNEDGSTPEGKDRAEAIK--------------------NAEGDKLNNVVNLGDLQAATNAATTKVGGNRGVTITPS-TNADGS 822
Adhesin B Gen 2 isoform 3                    744 KFTGEAPVQLGNVASSVRNEDGSTPEGKDRAEAIK--------------------NAEGDKLNNVVNLGDLQAATNAATTKVGGNRGVTITPS-TNADGS 822
Adhesin B Gen 2 isoform 4                    744 KFTGEAPVQLGNVASSVRNEDGSTPEGKDRAEAIK--------------------NAEGDKLNNVVNLGDLQAATNAATTKVGGNRGVTITPS-TNADGS 822
Adhesin B Gen 2 isoform 5                    760 KFTGEAPVQLGNVASSVRNEDGSTPEGKDRAEAIK--------------------NAEGDKLNNVVNLGDLQAATNAATTKVGGNRGVTITPS-TNADGS 838
```

```
Adhesin G Gen 2 isoform 1                   1294 VSDNVIDFTLNNDFTIGGPGKDGSIGVKGADGTVGVTLNGKDGSIGLTGPKGADGKDGASATISVKDGAKGLDGNDGKDGESKTRIVYEKPDGTTEEVAT 1393
Adhesin G Gen 2 isoform 3                   1294 VSDNVIDFTLNNDFTIGGPGKDGSIGVKGADGTVGVTLNGKDGSIGLTGPKGADGKDGASATISVKDGAKGLDGNDGKDGESKTRIVYEKPDGTTEEVAT 1393
Adhesin G Gen 2 isoform 4                   1291 VSDNVIDFTLNNDFTIGGPGKDGSIGVKGADGTVGVTLNGKDGSIGLTGPKGADGKDGASATISVKDGAKGLDGNDGKDGESKTRIVYEKPDGTTEEVAT 1390
Adhesin G Gen 2 isoform 5                   1294 VSDNVIDFTLNNDFTIGGPGKDGSIGVKGADGTVGVTLNGKDGSIGLTGPKGADGKD------------------------------------------- 1350
Adhesin G Gen 2 isoform 6                   1294 VSDNVIDFTLNNDFTIGGPGKDGSIGVKGADGTVGVTLNGKDGSIGLTGPKGADGKDGASATISVKDGAKGLDGNDGKDGESKTRIVYEKPDGTTEEVAT 1393
Adhesin G Gen 2 isoform 7                   1258 VSDNVIDFTLNNDFTIGGPGKDGSIGVKGADGTVGVTLNGKDGSIGLTGPKGADGKDGASATISVKDGAKGLDGNDGKDGESKTRIVYEKPDGTTEEVAT 1357
Adhesin G Gen 2 isoform 8                   1294 VSDNVIDFTLNNDFTIGGPGKDGSIGVKGADGTVGVTLNGKDGSIGLTGPKGADGKDGASATISVKDGAKGLDGNDGKDGESKTRIVYEKPDGTTEEVAT 1393
Adhesin G Gen 2 isoform 9                   1294 VSDNVIDFTLNNDFTIGGPGKDGSIGVKGADGTVGVTLNGKDGSIGLTGPKGADGKDGASATISVKDGAKGLDGNDGKDGESKTRIVYEKPDGTTEEVAT 1393
Adhesin G Gen 2 isoform 10                  1294 VSDNVIDFTLNNDFTIGGPGKDGSIGVKGADGTVGVTLNGKDGSIGLTGPKGADGKDGASATISVKDGAKGLDGNDGKDGESKTRIVYEKPDGTTEEVAT 1393
Adhesin G Gen 2 isoform 12                  1260 VSDNVIDFTLNNDFTIGGPGKDGSIGVKGADGTVGVTLNGKDGSIGLTGPKGADGKDGASATISVKDGAKGLDGNDGKDGESKTRIVYEKPDGTTEEVAT 1359
Adhesin G Gen 2 isoform 13                  1294 VSDNVIDFTLNNDFTIGGPGKDGSIGVKGADGTVGVVLNGKDGSIGLTGPKGADGKD------------------------------------------- 1350
Adhesin G Gen 2 isoform 14                  1294 VSDNVIDFTLNNDFTIGGPGKDGSIGVKGADGTVGVTLNGKDGSIGLTGPKGADGKDGASATISVKDGAKGLDGNDGKDGESKTRIVYEKPDGTTEEVAT 1393
Adhesin G Gen 2 isoform 15                  1294 VSDNVIDFTLNNDFTIGGPGKDGSIGVKGADGTVGVTLNGKDGSIGLTGPKGADGKDGASATISVKDGAKGLDGNDGKDGESKTRIVYEKPDGTTEEVAT 1393
Adhesin G Gen 2 isoform 16                  1294 VSDNVIDFTLNNDFTIGGPGKDGSIGVKGADGTVGVTLNGKDGSIGLTGPKGADGKDGASATISVKDGAKGLDGNDGKDGESKTRIVYEKPDGTTEEVAT 1393
Adhesin G Gen 2 isoform 17                  1294 VSDNVIDFTLNNDFTIGGPGKDGSIGVKGADGTVGVTLNGKDGSIGLTGPKGADGKDGASATISVKDGAKGLDGNDGKDGESKTRIVYEKPDGTTEEVAT 1393
Adhesin G Gen 2 isoform 18                  1294 VSDNVIDFTLNNDFTIGGPGKDGSIGVKGADGTVGVTLNGKDGSIGLTGPKGADGKDGASATISVKDGAKGLDGNDGKDGESKTRIVYEKPDGTTEEVAT 1393
Adhesin G Gen 2 isoform 19                  1300 VSDNVIDFTLNNDFTIGGPGKDGSIGVKGADGTVGVTLNGKDGSIGLTGPKGADGKDGASATISVKDGAKGLDGNDGKDGESKTRIVYEKPDGTTEEVAT 1399
Adhesin G Gen 2 isoform 20                  1294 VSDNVIDFTLNNDFTIGGPGKDGSIGVKGADGTVGVTLNGKDGSIGLTGPKGADGKDGASATISVKDGAKGLDGNDGKDGESKTRIVYEKPDGTTEEVAT 1393
Adhesin G isoform observed in CP0175216      920 NTYTFAGTTPSSTVSIGSVGNERTLTNVAAGRISDSSTDAINGSQLYAAYTEIDGLNTKVNELS------------------------------------ 983
Adhesin D Gen 2 isoform 1                    920 NTYTFAGTTPSSTVSIGSVGNERTLTNVAAGRILDSSTDAINGSQLYAAYTEIDGLNTKVNELS------------------------------------ 983
Adhesin D Gen 2 isoform 2                    920 NTYTFAGTTPSSTVSIGSVGNERTLTNVAAGRILDSSTDAINGSQLYAAYTEIDGLNTKVNELS------------------------------------ 983
Adhesin D Gen 2 isoform 3                    920 NTYTFAGTTPSSTVSIGSVGNERTLTNVAAGRILDSSTDAINGSQLYAAYTEIDGLNTKVNELS------------------------------------ 983
Adhesin D Gen 2 isoform 4                    920 NTYTFAGTTPSSTVSIGSVGNERTLTNVAAGRILDSSTDAINSSQLYAAYTEIDGLNTKVNELS------------------------------------ 983
Adhesin D Gen 2 isoform 6                    921 NTYTFAGTTPSSTVSIGSVGNERTLTNVAAGRILDSSTDAINGSQLYAAYTEIDGLNTKVNELS------------------------------------ 984
Adhesin B Gen 2 isoform 1                    823 TTYNVEAKTDGTTIKVDNEGNITANTSELGNNEDGTVKAPTQPNALLIAQTVADAVNNAGFNIK------------------------------------ 886
Adhesin B Gen 2 isoform 2                    823 TTYNVEAKTDGTTIKVDNEGNITANTSELGNNEDGTVKAPTQPNALLIAQTVADAVNNAGFNIK------------------------------------ 886
Adhesin B Gen 2 isoform 3                    823 TTYNVEAKTDGTTIKVDNEGNITANTSELGNNEDGTVKAPTQPNALLIAQTVADAVNNAGFNIK------------------------------------ 886
Adhesin B Gen 2 isoform 4                    823 TTYNVEAKTDGTTIKVDNEGNITANTSELGNNEDGTVKAPTQPNALLIAQTVADAVNNAGFNIK------------------------------------ 886
Adhesin B Gen 2 isoform 5                    839 TTYNVEAKTDGTTIKVDNEGNITANTSELGNNEDGTVKAPTQPNALLIAQTVADAVNNAGFNIK------------------------------------ 902
```

Fig S14 continued

```
Adhesin G Gen 2 isoform 1                  1394 LNDGLRFVGDDGKEIVKKLNETLKISGGDTDLTNLSDNNIGVVNNNG-ALTVKLAKDIDLTKDGSVTIGDTTVNNDGLTIKDGPSVTKAGINAGDKKIIN 1492
Adhesin G Gen 2 isoform 3                  1394 LNDGLRFVGDDGKEIVKKLNETLKISGGDTDLTNLSDNNIGVVNNNG-ALTVKLAKDIDLTKDGSVTIGDTTVNNDGLTIKDGPSVTKAGINAGDKKIIN 1492
Adhesin G Gen 2 isoform 4                  1391 LNDGLRFVGDDGKEIVKKLNETLKISGGDTDLTNLSDNNIGVVNNNG-ALTVKLAKDIDLTKDGSVTIGDTTVNNDGLTIKDGPSVTKAGINAGDKKIIN 1489
Adhesin G Gen 2 isoform 5                  1351 ---------------------------------------------------------------------------------------------------- 1350
Adhesin G Gen 2 isoform 6                  1394 LNDGLRFVGDDGKEIVKKLNETLKISGGDTDLTNLSDNNIGVVNNNG-ALTVKLAKDIDLTKDGSVTIGDTTVNNDGLTIKDGPSVTKAGINAGDKKIIN 1492
Adhesin G Gen 2 isoform 7                  1358 LNDGLRFVGDDGKEIVKKLNETLKISGGDTDLTNLSDNNIGVVNNNG-ALTVKLAKDIDLTKDGSVTIGDTTVNNDGLTIKDGPSVTKAGINAGDKKIIN 1456
Adhesin G Gen 2 isoform 8                  1394 LNDGLRFVGDDGKEIVKKLNETLKISGGDTDLTNLSDNNIGVVNNNG-ALTVKLAKDIDLTKDGSVTIGDTTVNNDGLTIKDGPSVTKAGINAGDKKIIN 1492
Adhesin G Gen 2 isoform 9                  1394 LNDGLRFVGDDGKEIVKKLNETLKISGGDTDLTNLSDNNIGVVNNNG-ALTVKLAKDIDLTKDGSVTIGDTTVNNDGLTIKDGPSVTKAGINAGDKKIIN 1492
Adhesin G Gen 2 isoform 10                 1394 LNDGLRFVGDDGKEIVKKLNETLKISGGDTDLTNLSDNNIGVVNNNG-ALTVKLAKDIDLTKDGSVTIGDTTVNNDGLTIKDGPSVTKAGINAGDKKIIN 1492
Adhesin G Gen 2 isoform 12                 1360 LNDGLRFVGDDGKEIVKKLNETLKISGGDTDLTNLSDNNIGVVNNNG-ALTVKLAKDIDLTKDGSVTIGDTTVNNDGLTIKDGPSVTKAGINAGDKKIIN 1458
Adhesin G Gen 2 isoform 13                 1351 ---------------------------------------------------------------------------------------------------- 1350
Adhesin G Gen 2 isoform 14                 1394 LNDGLRFVGDDGKEIVKKLNETLKISGGDTDLTNLSDNNIGVVNNNG-ALTVKLAKDIDLTKDGSVTIGDTTVNNDGLTIKDGPSVTKAGINAGDKKIIN 1492
Adhesin G Gen 2 isoform 15                 1394 LNDGLRFVGDDGKEIVKKLNETLKISGGDTDLTNLSDNNIGVVNNNG-ALTVKLAKDIDLTKDGSVTIGDTTVNNDGLTIKDGPSVTKAGINAGDKKIIN 1492
Adhesin G Gen 2 isoform 16                 1394 LNDGLRFVGDDGKEIVKKLNETLKISGGDTDLTNLSDNNIGVVNNNG-ALTVKLAKDIDLTKDGSVTIGDTTVNNDGLTIKDGPSVTKAGINAGDKKIIN 1492
Adhesin G Gen 2 isoform 17                 1394 LNDGLRFVGDDGKEIVKKLNETLKISGGDTDLTNLSDNNIGVVNNNG-ALTVKLAKDIDLTKDGSVTIGDTTVNNDGLTIKDGPSVTKAGINAGDKKIIN 1492
Adhesin G Gen 2 isoform 18                 1394 LNDGLRFVGDDGKEIVKKLNETLKISGGDTDLTNLSDNNIGVVNNNG-ALTVKLAKDIDLTKDGSVTIGDTTVNNDGLTIKDGPSVTKAGINAGDKKIIN 1492
Adhesin G Gen 2 isoform 19                 1400 LNDGLRFVGDDGKEIVKKLNETLKISGGDTDLTNLSDNNIGVVNNNG-ALTVKLAKDIDLTKDGSVTIGDTTVNNDGLTIKDGPSVTKAGINAGDKKIIN 1498
Adhesin G Gen 2 isoform 20                 1394 LNDGLRFVGDDGKEIVKKLNETLKISGGDTDLTNLSDNNIGVVNNNG-ALTVKLAKDIDLTKDGSVTIGDTTVNNDGLTIKDGPSVTKAGINAGDKKIIN 1492
Adhesin G isoform observed in CP0175216     984 -NGALTFVDDAGTEIVRKLGTSLNVKGG-ADATILTDNNIGVVATDANTLTVKLAKDIDLTPAGSVAVGNSKLNNNGLTINNGPSVTMTGVDAGKLKITN 1081
Adhesin D Gen 2 isoform 1                   984 -NGALTFVDDAGTEIVRKLGTSLNVKGG-ADATILTDNNIGVVATDANTLTVKLAKDIDLTPAGSVAVGNSKLNNNGLTINNGPSVTMTGVDAGKLKITN 1081
Adhesin D Gen 2 isoform 2                   984 -NGALTFVDDAGTEIVRKLGTSLNVKGG-ADATILTDNNIGVVATDANTLTVKLAKDIDLTPAGSVAVGNSKLNNNGLTINNGPSVTMTGVDAGKLKITN 1081
Adhesin D Gen 2 isoform 3                   984 -NGALTFVDDAGTEIVRKLGTSLNVKGG-ADATILTDNNIGVVATDANTLTVKLAKDIDLTPAGSVAVGNSKLNNNGLTINNGPSVTMTGVDAGKLKITN 1081
Adhesin D Gen 2 isoform 4                   984 -NGALTFVDDAGTEIVRKLGTSLNVKGG-ADATILTDNNIGVVATDANTLTVKLAKDIDLTPAGSVAVGNSKLNNNGLTINNGPSVTMTGVDAGKLKITN 1081
Adhesin D Gen 2 isoform 6                   985 -NGALTFVDDAGTEIVRKLGTSLNVKGG-ADATILTDNNIGVVATDANTLTVKLAKDIDLTPAGSVAVGNSKLNNNGLTINNGPSVTMTGVDAGKLKITN 1082
Adhesin B Gen 2 isoform 1                   887 -SAGNKAAGDQAATKLVKTGEEVVFEAG----------DNLTVKRDGNQFTFATAKDVSFNSVQFSENGPKITN-------DGDNIKVGDKDGKPTKITN 968
Adhesin B Gen 2 isoform 2                   887 -SAGNKAAGDQAATKLVKTGEEVVFEAG----------DNLTVKRDGNQFTFATAKDVSFNSVQFSENGPKITN-------DGDNIKVGDKDGKPTKITN 968
Adhesin B Gen 2 isoform 3                   887 -SAGNKAAGDQAATKLVKTGEEVVFEAG----------DNLTVKRDGNQFTFATAKDVSFNSVQFSENGPKITN-------DGDNIKVGDKDGKPTKITN 968
Adhesin B Gen 2 isoform 4                   887 -SAGNKAAGDQAATKLVKTGEEVVFEAG----------DNLTVKRDGNQFTFATAKDVSFNSVQFSENGPKITN-------DGDNIKVGDKDGKPTKITN 968
Adhesin B Gen 2 isoform 5                   903 -SAGNKAAGDQAATKLVKTGEEVVFEAG----------DNLTVKRDGNQFTFATAKDVSFNSVQFSENGPKITN-------DGDNIKVGDKDGKPTKITN 984

Adhesin G Gen 2 isoform 1                  1493 VQDGEISATSKDAVNGSQLYQVQEVANAGWNLTANGKDKGNVKPGATVDLNNKDGNIIITKEGNNVTFGLKNNLTIGGKDGKDGQIGIAGKDGKDGVTIK 1592
Adhesin G Gen 2 isoform 3                  1493 VQDGEISATSKDAVNGSQLYQVQEVANAGWNLTANGKDKGNVKPGATVDLNNKDGNIIITKEGNNVTFGLKNNLTIGGKDGKDGQIGIAGKDGKDGVTIK 1592
Adhesin G Gen 2 isoform 4                  1490 VQDGEISATSKDAVNGSQLYQVQEVANAGWNLTANGKDKGNVKPGATVDLNNKDGNIIITKEGNNVTFGLKNNLTIGGKDGKDGQIGIAGKDGKDGVTIK 1589
Adhesin G Gen 2 isoform 5                  1351 ---------------------------------------------------------------------------------------------------- 1350
Adhesin G Gen 2 isoform 6                  1493 VQDGEISATSKDAVNGSQLYQVQEVANAGWNLTANGKDKGNVKPGATVDLNNKDGNIIITKEGNNVTFGLKNNLTIGGKDGKDGQIGIAGKDGKDGVTIK 1592
Adhesin G Gen 2 isoform 7                  1457 VQDGEISATSKDAVNGSQLYQVQEVANAGWNLTANGKDKGNVKPGATVDLNNKDGNIIITKEGNNVTFGLKNNLTIGGKDGKDGQIGIAGKDGKDGVTIK 1556
Adhesin G Gen 2 isoform 8                  1493 VQDGEISATSKDAVNGSQLYQVQEVANAGWNLTANGKDKGNVKPGATVDLNNKDGNIIITKEGNNVTFGLKNNLTIGGKDGKDGQIGIAGKDGKD----- 1587
Adhesin G Gen 2 isoform 9                  1493 VQDGEISATSKDAVNGSQLYQVQEVANAGWNLTANGKDKGNVKPGATVDLNNKDGNIIITKEGNNVTFGLKNNLTIGGKDGKDGQIGIAGKDGKDGVTIK 1592
Adhesin G Gen 2 isoform 10                 1493 VQDGEISATSKDAVNGSQLYQVQEVANAGWNLTANGKDKGNVKPGATVDLNNKDGNIIITKEGNNVTFGLKNNLTIGGKDGKDGQIGIAGKDGKDGVTIK 1592
Adhesin G Gen 2 isoform 12                 1459 VQDGEISATSKDAVNGSQLYQVQEVANAGWNLTANGKDKGNVKPGATVDLNNKDGNIIITKEGNNVTFGLKNNLTIGGKDGKDGQIGIAGKDGKDGVTIK 1558
Adhesin G Gen 2 isoform 13                 1351 ---------------------------------------------------------------------------------------------------- 1350
Adhesin G Gen 2 isoform 14                 1493 VQDGEISATSKDAVNGSQLYQVQEVANAGWNLTANGKDKGNVKPGATVDLNNKDGNIIITKEGNNVTFGLKNNLTIGGKDGKDGQIGIAGKDGKDGVTIK 1592
Adhesin G Gen 2 isoform 15                 1493 VQDGEISATSKDAVNGSQLYQVQEVANAGWNLTANGKDKGNVKPGATVDLNNKDGNIIITKEGNNVTFGLKNNLTIGGKDGKDGQIGIAGKDGKDGVTIK 1592
Adhesin G Gen 2 isoform 16                 1493 VQDGEISATSKDAVNGSQLYQVQEVANAGWNLTANGKDKGNVKPGATVDLNNKDGNIIITKEGNNVTFGLKNNLTIGGKDGKDGQIGIAGKDGKDGVTIK 1592
Adhesin G Gen 2 isoform 17                 1493 VQDGEISATSKDAVNGSQLYQVQEVANAGWNLTANGKDKGNVKPGATVDLNNKDGNIIITKEGNNVTFGLKNNLTIGGKDGKDGQIGIAGKDGKDGVTIK 1592
Adhesin G Gen 2 isoform 18                 1493 VQDGEISATSKDAVNGSQLYQVQEVANAGWNLTANGKDKGNVKPGATVDLNNKDGNIIITKEGNNVTFGLKNNLTIGGKDGKDGQIGIAGKDGKDGVTIK 1592
Adhesin G Gen 2 isoform 19                 1499 VQDGEISATSKDAVNGSQLYQVQEVANAGWNLTANGKDKGNVKPGATVDLNNKDGNIIITKEGNNVTFGLKNNLTIGGKDGKDGQIGIAGKDGKDGVTIK 1598
Adhesin G Gen 2 isoform 20                 1493 VQDGEISATSKDAVNGSQLYQVQEVANAGWNLTANGKDKGNVKPGATVDLNNKDGNIIITKEGNNVTFGLKNNLTIGGKDGKDGQIGIAGKDGKDGVTIK 1592
Adhesin G isoform observed in CP0175216    1082 VADGDISPISADAVNGSQLYDTANTIATALGGNSSVNANGAVSAPSYTVVDG------------------------------------------------ 1133
Adhesin D Gen 2 isoform 1                  1082 VADGDISPISADAVNGSQLYDTANTIATALGGNSSVNANGAVSAPSYTVVDG------------------------------------------------ 1133
Adhesin D Gen 2 isoform 2                  1082 VADGDISPISADAVNGSQLYDTANTIATALGGNSSVNANGAVSAPSYTVVDG------------------------------------------------ 1133
Adhesin D Gen 2 isoform 3                  1082 VADGDISPISADAVNGSQLYDTANTIATALGGNSSVNANGAVSAPSYTVVDG------------------------------------------------ 1133
Adhesin D Gen 2 isoform 4                  1082 VADGDISPISADAVNGSQLYDTANTIATALGGNSSVNANGAVSAPSYTVVDG------------------------------------------------ 1133
Adhesin D Gen 2 isoform 6                  1083 VADGDISPISADAVNGSQLYDTANTIATALGGNSSVNANGAVSAPSYTVVDG------------------------------------------------ 1134
Adhesin B Gen 2 isoform 1                   969 VADGDISPVSTDVINGKQLNNYAKVNGNNIGTDEDGSINIVNGNGTTITSDKAG---------------------------------------------- 1022
Adhesin B Gen 2 isoform 2                   969 VADGDISPVSTDVINGKQLNNYAKVNGNNIGTDEDGSINIVNGNGTTITSDKAG---------------------------------------------- 1022
Adhesin B Gen 2 isoform 3                   969 VADGDISPVSTDVINGKQLNNYAKVNGNNIGTDEDGSINIVNGNGTTITSDKAG---------------------------------------------- 1022
Adhesin B Gen 2 isoform 4                   969 VADGDISPVSTDVINGKQLNNYAKVNGNNIGTDEDGSINIVNGNGTTITSDKAG---------------------------------------------- 1022
Adhesin B Gen 2 isoform 5                   985 VADGDISPVSTDVINGKQLNNYAKVNGNNIGTDEDGSINIVNGNGTTITSDKAG---------------------------------------------- 1038
```

Fig S14 continued

Sequence	Start	Alignment	End
Adhesin G Gen 2 isoform 1	1593	GDGTITAGRDGKDGVDGSIGATGKDGASVVLNGKDGSIGLTGPKGADGKDGASANIAVKDGAEGVDGTNGKDGLPGENGKTRIVYETKDKDGKTVTEQVA	1692
Adhesin G Gen 2 isoform 3	1593	GDGTITAGRDGKDGVDGSIGATGKDGASVVLNGKDGSIGLTGPKGADGKDGASANIAVKDGAEGVDGTNGKDGLPGENGKTRIVYETKDKDGKTVTEQVA	1692
Adhesin G Gen 2 isoform 4	1590	GDGTITAGRDGKDGVDGSIGATGKDGASVVLNGKDGSIGLTGPKGADGKDGASANIAVKDGAEGVDGTNGKDGLPGENGKTRIVYETKDKDGKTVTEQVA	1689
Adhesin G Gen 2 isoform 5	1351	--GASANIAVKDGAEGVDGTNGKDGLPGENGKTRIVYETKDKDGKTVTEQVA	1400
Adhesin G Gen 2 isoform 6	1593	GDGTITAGRDGKYGVDGSIGATGKDGASVVLNGKDGSIGLTGPKGADGKDGASANIAVKDGAEGVDGTNGKDGLPGENGKTRIVYETKDKDGKTVTEQVA	1692
Adhesin G Gen 2 isoform 7	1557	GDGTITAGRDGKDGVDGSIGATGKDGASVVLNGKDGSIGLTGPKGADGKDGASANIAVKDGAEGVDGTNGKDGLPGENGKTRIVYETKDKDGKTVTEQVA	1656
Adhesin G Gen 2 isoform 8	1588	-------------------------GASVVLNGKDGSIGLTGPKGADGKDGASANIAVKDGAEGVDGTNGKDGLPGENGKTRIVYETKDKDGKTVTEQVA	1662
Adhesin G Gen 2 isoform 9	1593	GDGTITAGRDGKDGVDGSIGATGKDGASVVLNGKDGSIGLTGPKGADGKDGASANIAVKDGAEGVDGTNGKDGLPGENGKTRIVYETKDKDGKTVTEQVA	1692
Adhesin G Gen 2 isoform 10	1593	GDGTITAGRDGKDGVDGSIGATGKDGASVVLNGKDGSIGLTGPKGADGKDGASANIAVKDGAEGVDGTNGKDGLPGENGKTRIVYETKDKDGKTVTEQVA	1692
Adhesin G Gen 2 isoform 12	1559	GDGTITAGRDGKDGVDGSIGATGKDGASVVLNGKDGSIGLTGPKGADGKDGASANIAVKDGAEGVDGTNGKDGLPGENGKTRIVYETKDKDGKTVTEQVA	1658
Adhesin G Gen 2 isoform 13	1351	--GASANIAVKDGAEGVDGTNGKDGLPGENGKTRIVYETKDKDGKTVTEQVA	1400
Adhesin G Gen 2 isoform 14	1593	GDGTITAGRDGKDGVDGSIGATGKDGASVVLNGKDGSIGLTGPKGADGKDGASANIAVKDGAEGVDGTNGKDGLPGENGKTRIVYETKDKDGKTVTEQVA	1692
Adhesin G Gen 2 isoform 15	1593	GDGTITAGRDGKDGVDGSIGATGKDGASVVLNGKDGSIGLTGPKGADGKDGASANIAVKDGAEGVDGTNGKDGLPGENGKTRIVYETKDKDGKTVTEQVA	1692
Adhesin G Gen 2 isoform 16	1593	GDGTITAGRDGKDGVDGSIGATGKDGASVVLNGKDGSIGLTGPKGADGKDGASANIAVKDGAEGVDGTNGKDGLPGENGKTRIVYETKDKDGKTVTEQVA	1692
Adhesin G Gen 2 isoform 17	1593	GDGTITAGRDGKDGVDGSIGATGKDGASVVLNGKDGSIGLTGPKGADGKDGASANIAVKDGAEGVDGTNGKDGLPGENGKTRIVYETKDKDGKTVTEQVA	1692
Adhesin G Gen 2 isoform 18	1593	GDGTITAGRDGKDGVDGSIGATGKDGASVVLNGKDGSIGLTGPKGADGKDGASANIAVKDGAEGVDGTNGKDGLPGENGKTRIVYETKDKDGKTVTEQVA	1692
Adhesin G Gen 2 isoform 19	1599	GDGTITAGRDGKDGVDGSIGATGKDGASVVLNGKDGSIGLTGPKGADGKDGASANIAVKDGAEGVDGTNGKDGLPGENGKTRIVYETKDKDGKTVTEQVA	1698
Adhesin G Gen 2 isoform 20	1593	GDGTITAGRDGKDGVDGSIGATGKDGASVVLNGKDGSIGLTGPKGADGKDGASANIAVKDGAEGVDGTNGKDGLPGENGKTRIVYETKDKDGKTVTEQVA	1692
Adhesin G isoform observed in CP0175216	1134	---APTNEVSKTVNNVGSAITALNDAVTSPLTFAGDTGTPSQRKLGSTVTVKGGVSNESQLT	1192
Adhesin D Gen 2 isoform 1	1134	---APTNEVSKTVNNVGSAITALNDAVTSPLTFAGDTGTPSQRKLGSTVTVKGGVSNESQLT	1192
Adhesin D Gen 2 isoform 2	1134	---APTNEVSKTVNNVGSAITALNDAVTSPLTFAGDTGTPSQRKLGSTVTVKGGVSNESQLT	1192
Adhesin D Gen 2 isoform 3	1134	---APTNEVSKTVNNVGSAITALNDAVTSPLTFAGDTGTPSQRKLGSTVTVKGGVSNESQLT	1192
Adhesin D Gen 2 isoform 4	1134	---APTNEVSKTVNNVGSAITALNDAVTSPLTFAGDTGTPSQRKLGSTVTVKGGVSNESQLT	1192
Adhesin D Gen 2 isoform 6	1135	---APTNEVSKTVNNVGSAITALNDAVTSPLTFAGDTGTPSQRKLGSTVTVKGGVSNESQLT	1193
Adhesin B Gen 2 isoform 1	1023	--EVKVNVNITDLTVADNGKINVQDPNGTG--SRFVNATTVANAVNNVSWNV	1070
Adhesin B Gen 2 isoform 2	1023	--EVKVNVNITDLTVADNGKINVQDPNGTG--SRFVNATTVANAVNNVSWNV	1070
Adhesin B Gen 2 isoform 3	1023	--EVKVNVNITDLTVADNGKINVQDPNGTG--SRFVNATTVANAVNNVSWNV	1070
Adhesin B Gen 2 isoform 4	1023	--EVKVNVNITDLTVADNGKINVQDPNGTG--SRFVNATTVANAVNNVSWNV	1070
Adhesin B Gen 2 isoform 5	1039	--EVKVNVNITDLTVADNGKINVQDPNGTG--SRFVNATTVANAVNNVSWNV	1086

Sequence	Start	Alignment	End
Adhesin G Gen 2 isoform 1	1693	TLNDGLIFSGNNEDVKNRQKLNTEVKVKGEGVDKTASENFKSASGNINVKANGTDTLEVQLAKAIDLTEDGSVTTGDTTVNNNGLTIKDGPS----VTK	1787
Adhesin G Gen 2 isoform 3	1693	TLNDGLIFSGNNEDVKNRQKLNTEVKVKGEGVDKTASENFKSASGNINVKANGTDTLEVQLAKAIDLTEDGSVTTGDTTVNNNGLTIKDGPS----VTK	1787
Adhesin G Gen 2 isoform 4	1690	TLNDGLIFSGNNEDVKNRQKLNTEVKVKGEGVDKTASENFKSASGNINVKANGTDTLEVQLAKAIDLTEDGSVTTGDTTVNNNGLTIKDGPS----VTK	1784
Adhesin G Gen 2 isoform 5	1401	TLNDGLIFSGNNEDVKNRQKLNTEVKVKGEGVDKTASENFKSASGNINVKANGTDTLEVQLAKAIDLTEDGSVTTGDTTVNNNGLTIKDGPS----VTK	1495
Adhesin G Gen 2 isoform 6	1693	TLNDGLIFSGNNEDVKNRQKLNTEVKVKGEGVDKTASENFKSASGNINVKANGTDTLEVQLAKAIDLTEDGSVTTGDTTVNNNGLTIKDGPS----VTK	1787
Adhesin G Gen 2 isoform 7	1657	TLNDGLIFSGNNEDVKNRQKLNTEVKVKGEGVDKTASENFKSASGNINVKANGTDTLEVQLAKAIDLTEDGSVTTGDTTVNNNGLTIKDGPS----VTK	1751
Adhesin G Gen 2 isoform 8	1663	TLNDGLIFSGNNEDVKNRQKLNTEVKVKGEGVDKTASENFKSASGNINVKANGTDTLEVQLAKAIDLTEDGSVTTGDTTVNNNGLTIKDGPS----VTK	1757
Adhesin G Gen 2 isoform 9	1693	TLNDGLIFSGNNEDVKNRQKLNTEVKVKGEGVDKTASENFKSASGNINVKANGTDTLEVQLAKAIDLTEDGSVTTGDTTVNNNGLTIKDGPS----VTK	1787
Adhesin G Gen 2 isoform 10	1693	TLNDGLIFSGNNEDVKNRQKLNTEVKVKGEGVDKTASENFKSASGNINVKANGTDTLEVQLAKAIDLTEDGSVTTGDTTVNNNGLTIKDGPS----VTK	1787
Adhesin G Gen 2 isoform 12	1659	TLNDGLIFSGNNEDVKNRQKLNTEVKVKGEGVDKTASENFKSASGNINVKANGTDTLEVQLAKAIDLTEDGSVTTGDTTVNNNGLTIKDGPS----VTK	1753
Adhesin G Gen 2 isoform 13	1401	TLNDGLIFSGNNEDVKNRQKLNTEVKVKGEGVDKTASENFKSASGNINVKANGTDTLEVQLAKAIDLTEDGSVTTGDTTVNNNGLTIKDGPS----VTK	1495
Adhesin G Gen 2 isoform 14	1693	TLNDGLIFSGNNEDVKNRQKLNTEVKVKGEGVDKTASENFKSASGNINVKANGTDTLEVQLAKAIDLTEDGSVTTGDTTVNNNGLTIKDGPS----VTK	1787
Adhesin G Gen 2 isoform 15	1693	TLNDGLIFSGNNEDVKNRQKLNTEVKVKGEGVDKTASENFKSASGNINVKANGTDTLEVQLAKAIDLTEDGSVTTGDTTVNNNGLTIKDGPS----VTK	1787
Adhesin G Gen 2 isoform 16	1693	TLNDGLIFSGNNEDVKNRQKLNTEVKVKGEGVDKTASENFKSASGNINVKANGTDTLEVQLAKAIDLTEDGSVTTGDTTVNNNGLTIKDGPS----VTK	1787
Adhesin G Gen 2 isoform 17	1693	TLNDGLIFSGNNEDVKNRQKLNTEVKVKGEGVDKTASENFKSASGNINVKANGTDTLEVQLAKAIDLTEDGSVTTGDTTVNNNGLTIKDGPS----VTK	1787
Adhesin G Gen 2 isoform 18	1693	TLNDGLIFSGNNEDVKNRQKLNTEVKVKGEGVDKTASENFKSASGNINVKANGTDTLEVQLAKAIDLTEDGSVTTGDTTVNNNGLTIKDGPS----VTK	1787
Adhesin G Gen 2 isoform 19	1699	TLNDGLIFSGNNEDVKNRQKLNTEVKVKGEGVDKTASENFKSASGNINVKANGTDTLEVQLAKAIDLTEDGSVTTGDTTVNNNGLTIKDGPS----VTK	1793
Adhesin G Gen 2 isoform 20	1693	TLNDGLIFSGNNEDVKNRQKLNTEVKVKGEGVDKTASENFKSASGNINVKANGTDTLEVQLAKAIDLTEDGSVTTGDTTVNNNGLTIKDGPS----VTK	1787
Adhesin G isoform observed in CP0175216	1193	DNNIGVISNGNGS---LTVKLAKDIKVNSVTAQTVTAN----------------------------IAVADTVKTGDTTIDTNGLTIVGGPS----ITK	1256
Adhesin D Gen 2 isoform 1	1193	DNNIGVISNGNGS---LTVKLAKDIKVNSVTAQTVTAN----------------------------IAVADTVKTGDTTIDTNGLTIVGGPS----ITK	1256
Adhesin D Gen 2 isoform 2	1193	DNNIGVISNGNGS---LTVKLAKDIKVNSVTAQTVTAN----------------------------IAVADTVKTGDTTIDTNGLTIVGGPS----ITK	1256
Adhesin D Gen 2 isoform 3	1193	DNNIGVISNGNGS---LTVKLAKDIKVNSVTAQTVTAN----------------------------IAVADTVKTGDTTIDTNGLTIVGGPS----ITK	1256
Adhesin D Gen 2 isoform 4	1193	DNNIGVISNGNGS---LTVKLAKDIKVNSVTAQTVTAN----------------------------IAVADTVKTGDTTIDTNGLTIVGGPS----ITK	1256
Adhesin D Gen 2 isoform 6	1194	DNNIGVISNGNGS---LTVKLAKDIKVNSVTAQTVTAN----------------------------IAVADTVKTGDTTIDTNGLTIVGGPS----ITK	1257
Adhesin B Gen 2 isoform 1	1071	DSKAVGTGAVEGDKAPAKVKAGSTVSVNAGNNIKVTRKGSD-----------------VTVAVSDTPEFTS-KTGDTLVNNNGVTINNGSAGKAVSLTK	1151
Adhesin B Gen 2 isoform 2	1071	DSKAVGTGAVEGDKAPAKVKAGSTVSVNAGNNIKVTRKGSD-----------------VTVAVSDTPEFTS-KTGDTLVNNNGVTINNGSAGKAVSLTK	1151
Adhesin B Gen 2 isoform 3	1071	DSKAVGTGAVEGDKAPAKVKAGSTVSVNAGNNIKVTRKGSD-----------------VTVAVSDTPEFTS-KTGDTLVNNNGVTINNGSAGKAVSLTK	1151
Adhesin B Gen 2 isoform 4	1071	DSKAVGTGAVEGDKAPAKVKAGSTVSVNAGNNIKVTRKGSD-----------------VTVAVSDTPEFTSVKTGDTLVNNNGVTINNGSAGKAVSLTK	1152
Adhesin B Gen 2 isoform 5	1087	DSKAVGTGAVEGDKAPAKVKAGSTVSVNAGNNIKVTRKGSD-----------------VTVAVSDTPEFTSVKTGDTLVNNNGVTINNGSAGKAVSLTK	1168

Fig S14 continued

```
Adhesin G Gen 2 isoform 1               1788 GGIDAKGTKITNVKDGDVTATSKDVVSGSQLYNAMQNTGWYLTVDGNVAETSEKGAQRVNNNGKVAVSGGKNIVVSRKGSTVEVATSSNLEFDSVKVGGT 1887
Adhesin G Gen 2 isoform 3               1788 GGIDAKGTKITNVKDGDVTATSKDVVSGSQLYNAMQNTGWYLTVDGNVAETSEKGAQRVNNNGKVAVSGGKNIVVSRKGSTVEVATSSNLEFDSVKVGGT 1887
Adhesin G Gen 2 isoform 4               1785 GGIDAKGTKITNVKDGDVTATSKDVVSGSQLYNAMQNTGWYLTVDGNVAETSEKGAQRVNNNGKVAVSGGKNIVVSRKGSTVEVATSSNLEFDSVKVGGT 1884
Adhesin G Gen 2 isoform 5               1496 GGIDAKGTKITNVKDGDVTATSKDVVSGSQLYNAMQNTGWYLTVDGNVAETSEKGAQRVNNNGKVAVSGGKNIVVSRKGSTVEVATSSNLEFDSVKVGGT 1595
Adhesin G Gen 2 isoform 6               1788 GGIDAKGTKITNVKDGDVTATSKDVVSGSQLYNAMQNTGWYLTVDGNVAETSEKGAQRVNNNGKVAVSGGKNIVVSRKGSTVEVATSSNLEFDSVKVGGT 1887
Adhesin G Gen 2 isoform 7               1752 GGIDAKGTKITNVKDGDVTATSKDVVSGSQLYNAMQNTGWYLTVDGNVAETSEKGAQRVNNNGKVAVSGGKNIVVSRKGSTVEVATSSNLEFDSVKVGGT 1851
Adhesin G Gen 2 isoform 8               1758 GGIDAKGTKITNVKDGDVTATSKDVVSGSQLYNAMQNTGWYLTVDGNVAETSEKGAQRVNNNGKVAVSGGKNIVVSRKGSTVEVATSSNLEFDSVKVGGT 1857
Adhesin G Gen 2 isoform 9               1788 GGIDAKGTKITNVKDGDVTATSKDVVSGSQLYNAMQNTGWYLTVDGNVAETSEKGAQRVNNNGKVAVSGGKNIVVSRKGSTVEVATSSNLEFDSVKVGGT 1887
Adhesin G Gen 2 isoform 10              1788 GGIDAKGTKITNVKDGDVTATSKDVVSGSQLYNAMQNTGWYLTVDGNVAETSEKGAQRVNNNGKVAVSGGKNIVVSRKGSTVEVATSSNLEFDSVKVGGT 1887
Adhesin G Gen 2 isoform 12              1754 GGIDAKGTKITNVKDGDVTATSKDVVSGSQLYNAMQNTGWYLTVDGNVAETSEKGAQRVNNNGKVAVSGGKNIVVSRKGSTVEVATSSNLEFDSVKVGGT 1853
Adhesin G Gen 2 isoform 13              1496 GGIDAKGTKITNVKDGDVTATSKDVVSGSQLYNAMQNTGWYLTVDGNVAETSEKGAQRVNNNGKVAVSGGKNIVVSRKGSTVEVATSSNLEFDSVKVGGT 1595
Adhesin G Gen 2 isoform 14              1788 GGIDAKGTKITNVKDGDVTATSKDVVSGSQLYNAMQNTGWYLTVDGNVAETSEKGAQRVNNNGKVAVSGGKNIVVSRKG..................... 1866
Adhesin G Gen 2 isoform 15              1788 GGIDAKGTKITNVKDGDVTATSKDVVSGSQLYNAMQNTGWYLTVDGNVAETSEKGAQRVNNNGKVAVSGGKNIVVSRKGSTVEVATSSNLEFDSVKVGGT 1887
Adhesin G Gen 2 isoform 16              1788 GGIDAKGTKITNVKDGDVTATSKDVVSGSQLYNAMQNTGWYLTVDGNVAETSEKGAQRVNNNGKVAVSGGKNIVVSRKGSTVEVATSSNLEFDSVKVGGT 1887
Adhesin G Gen 2 isoform 17              1788 GGIDAKGTKITNVKDGDVTATSKDVVSGSQLYNAMQNTGWYLTVDGNVAETSEKGAQRVNNNGKVAVSGGKNIVVSRKGSTVEVATSSNLEFDSVKVGGT 1887
Adhesin G Gen 2 isoform 18              1788 GGIDAKGTKITNVKDGDVTATSKDVVSGSQLYNAMQNTGWYLTVDGNVAETSEKGAQRVNNNGKVAVSGGKNIVVSRKGSTVEVATSSNLEFDSVKVGGT 1887
Adhesin G Gen 2 isoform 19              1794 GGIDAKGTKITNVKDGDVTATSKDVVSGSQLYNAMQNTGWYLTVDGNVAETSEKGAQRVNNNGKVAVSGGKNIVVSRKGSTVEVATSSNLEFDSVKVGGT 1893
Adhesin G Gen 2 isoform 20              1788 GGIDAKGTKITNVKDGDVTATSKDVVSGSQLYNAMQNTGWYLTVDGNVAETSEKGAQRVNNNGKVAVSGGKNIVVSRKGSTVEVATSSNLEFDSVKVGGT 1887
Adhesin G isoform observed in CP0175216  1257 TGINAAGTKVTNVKAG.................................................................................... 1272
Adhesin D Gen 2 isoform 1               1257 TGINAAGTKVTNVKAG.................................................................................... 1272
Adhesin D Gen 2 isoform 2               1257 TGINAAGTKVTNVKAG.................................................................................... 1272
Adhesin D Gen 2 isoform 3               1257 TGINAAGTKVTNVKAG.................................................................................... 1272
Adhesin D Gen 2 isoform 4               1257 TGINAAGTKVTNVKAG.................................................................................... 1272
Adhesin D Gen 2 isoform 6               1258 TGINAAGTKVTNVKAG.................................................................................... 1273
Adhesin B Gen 2 isoform 1               1152 DGLNNGGNRITNVKAG.................................................................................... 1167
Adhesin B Gen 2 isoform 2               1152 DGLNNGGNRITNVKAG.................................................................................... 1167
Adhesin B Gen 2 isoform 3               1152 DGLNNGGNRITNVKAG.................................................................................... 1167
Adhesin B Gen 2 isoform 4               1153 DGLNNGGNRITNVKAG.................................................................................... 1168
Adhesin B Gen 2 isoform 5               1169 DGLNNGGNRITNVKAG.................................................................................... 1184

Adhesin G Gen 2 isoform 1               1888 TISSTVAKDGVNELNIAGSKNAQTRITNVAPGVKGTDAVNVNQLKGAENRLNNRISNVDKDLRAGIAGALAAGNLYHVTQPGKSMVSAGVGAYRGQGAIA 1987
Adhesin G Gen 2 isoform 3               1888 TISSTVAKDGVNELNIAGSKNAQTRITNVAPGVKGTDAVNVNQLKGAENRLNNRISNVDKDLRAGIAGALAAGNLYHVTQPGKSMVSAGVGAYRGQGAIA 1987
Adhesin G Gen 2 isoform 4               1885 TISSTVAKDGVNELNIAGSKNAQTRITNVAPGVKGTDAVNVNQLKGAENRLNNRISNVDKDLRAGIAGALAAGNLYHVTQPGKSMVSAGVGAYRGQGAIA 1984
Adhesin G Gen 2 isoform 5               1596 TISSTVAKDGVNELNIAGSKNAQTRITNVAPGVKGTDAVNVNQLKGAENRLNNRISNVDKDLRAGIAGALAAGNLYHVTQPGKSMVSAGVGAYRGQGAIA 1695
Adhesin G Gen 2 isoform 6               1888 TISSTVAKDGVNELNIAGSKNAQTRITNVAPGVKGTDAVNVNQLKGAENRLNNRISNVDKDLRAGIAGALAAGNLYHVTQPGKSMVSAGVGAYRGQGAIA 1987
Adhesin G Gen 2 isoform 7               1852 TISSTVAKDGVNELNIAGSKNAQTRITNVAPGVKGTDAVNVNQLKGAENRLNNRISNVDKDLRAGIAGALAAGNLYHVTQPGKSMVSAGVGAYRGQGAIA 1951
Adhesin G Gen 2 isoform 8               1858 TISSTVAKDGVNELNIAGSKNAQTRITNVAPGVKGTDAVNVNQLKGAENRLNNRISNVDKDLRAGIAGALAAGNLYHVTQPGKSMVSAGVGAYRGQGAIA 1957
Adhesin G Gen 2 isoform 9               1888 TISSTVAKDGVNELNIAGSKNAQTRITNVAPGVKGTDAVNVNQLKGAENRLNNRISNVDKDLRAGIAGALAAGNLCHVTQPGKSMVSAGVGAYRGQGAIA 1987
Adhesin G Gen 2 isoform 10              1888 TISSTVAKDGVNELNIAGSKNAQTRITNVAPGVKGTDAVNVNQLKGAENRLNNRISDVDKDLRAGIAGALAAGNLYHVTQPGKSMVSAGVGAYRGQGAIA 1987
Adhesin G Gen 2 isoform 12              1854 TISSTVAKDGVNELNIAGSKNAQTRITNVAPGVKGTDAVNVNQLKGAENRLNNRISNVDKDLRAGIAGALAAGNLYHVTQPGKSMVSAGVGAYRGQGAIA 1953
Adhesin G Gen 2 isoform 13              1596 TISSTVAKDGVNELNIAGSKNAQTRITNVAPGVKGTDAVNVNQLKGAENRLNNRISNVDKDLRAGIAGALAAGNLYHVTQPGKSMVSAGVGAYRGQGAIA 1695
Adhesin G Gen 2 isoform 14              1867 ...STVAKDGVNELNIAGSKNAQTRITNVAPGVKGTDAVNVNQLKGAENRLNNRISNVDKDLRAGIAGALAAGNLYHVTQPGKSMVSAGVGAYRGQGAIA 1963
Adhesin G Gen 2 isoform 15              1888 TISSTVAKDGVNELNIAGSKNAQTRITNVAPGVKGTSAVNVNQLKGAENRLNNRISNVDKDLRAGIAGALAAGNLYHVTQPGKSMVSAGVGAYRGQGAIA 1987
Adhesin G Gen 2 isoform 16              1888 TISSTVAKDGVNELNIAGSKNAQTRITNVAPGVKGTDAVNVYQMKGAENRLNNRISNVDKDLRAGIAGALAAGNLYHVTQPGKSMVSAGVGAYRGQGAIA 1987
Adhesin G Gen 2 isoform 17              1888 TISSTVAKDGVNELNIAGSKNAQTRITNVAPGVKGTDAVNVKQLKGAENRLNNRISNVDKDLRAGIAGALAAGNLYHVTQPGKSMVSAGVGAYRGQGAIA 1987
Adhesin G Gen 2 isoform 18              1888 TISSTVAKDGVNELNIAGSKNAQTRITNVAPGVKGTDAVNVNQLKGAENRLNNRISNVDKDLRAGIAGALAAGNLYHVTQPGKSMVSAGVGAYRGQGAIA 1987
Adhesin G Gen 2 isoform 19              1894 TISSTVAKDGVNELNIAGSKNAQTRITNVAPGVKGTDAVNVNQLKGAENRLNNRISNVDKDLRAGIAGALAAGNLYHVTQPGKSMVSAGVGAYRGQGAIA 1993
Adhesin G Gen 2 isoform 20              1888 TISSTVAKDGVNELNIAGSKNAQTRITNVAPGVKGTDAVNVNQLKGAENRLNNRISNVDKDLRAGIAGALAAGNLYHVTQPGKSMVSAGVGAYRGQGAIA 1987
Adhesin G isoform observed in CP0175216  1273 .......TEDTDAVNFSQLKATEKNIN..................NKINNIDSKVNKVDKRLRAGIAGATATAGLPQAYLPGKSMLATAGDTYRNEAAIA 1347
Adhesin D Gen 2 isoform 1               1273 .......TEDTDAVNFSQLKATEKNIN..................NKINNIDSKVNKVDKRLRAGIAGATATAGLPQAYLPGKSMLATAGDTYRNEAAIA 1347
Adhesin D Gen 2 isoform 2               1273 .......TEDTDAVNFSQLKATEKNIN..................NKINNIDSKVNKVDKRLRAGIAGATATAGLPQAYLPGKSMLATAGDTYRNEAAIA 1347
Adhesin D Gen 2 isoform 3               1273 .......TEDTDAVNFSQLKATEKNIN..................NKINNIDSKVNKVDKRLRAGIAGATATAGLPQAYLPGKSMLATAGDTYRNEAAIA 1347
Adhesin D Gen 2 isoform 4               1273 .......TEDTDAVNFSQLKATEKNIN..................NKINNIDSKVNKVDKRLRAGIAGATATAGLPQAYLPGKSMLATAGDTYRNEAAIA 1347
Adhesin D Gen 2 isoform 6               1274 .......TEDTDAVNFSQLKATEKNIN..................NKINNIDSKVNKVDKRLRAGIAGATATAGLPQAYLPGKSMLATAGDTYRNEAAIA 1348
Adhesin B Gen 2 isoform 1               1168 .......EADTDAVNVGQLK.........................GAVNHLNNKIHRNNREARAGIAGSNAAAALPQVYIPGKSMVAAAGGTFKGENALA 1235
Adhesin B Gen 2 isoform 2               1168 .......EADTDAVNVGQLK.........................GAVNHLNNKIHRNNREARAGIAGSNAAAALPQVYIPGKSMVAAAGGTFKGENALA 1235
Adhesin B Gen 2 isoform 3               1168 .......EADTDAVNVGQLK.........................GAVNHLNNKIHRNNREARAGIAGSN-AAALPQVYIPGKSMVAAAGGTFKGENALA 1234
Adhesin B Gen 2 isoform 4               1169 .......EADTDAVNVGQLK.........................GAVNHLNNKIHRNNREARAGIAGSNAAAALPQVYIPGKSMVAAAGGTFKGENALA 1236
Adhesin B Gen 2 isoform 5               1185 .......EADTDAVNVGQLK.........................GAVNHLNNKIHRNNREARAGIAGSNAAAALPQVYIPGKSMVAAAGGTFKGENALA 1252
```

Fig S14 continued

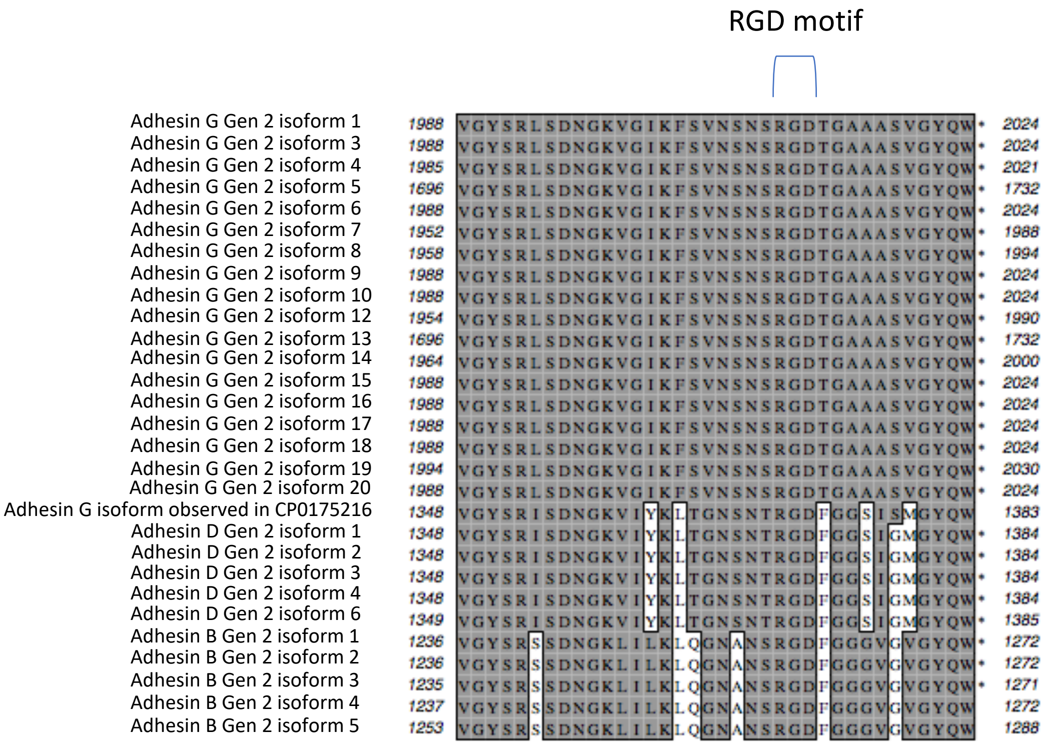

Supplement: Supplementary file 19 — Additional file 19: Figure S14. Alignment of all unique genotype 2 adhesin B, D, and G isoforms observed in this study. The alignment contains all unique genotype 2 adhesin B, D, and G isoforms observed in this study. The one genotype 2 “like” adhesin G isoform observed in a genotype 1 strain (GenBank# CP0175216) is also included in the alignment. The conserved RGD motif is highlighted with a bracket. Areas of 51% chemical identity or greater are indicated with grey boxes. [file 12866_2020_1932_MOESM19_ESM.pdf]
